# Supplementary material for: Macrophage metabolic reprogramming during dietary stress influences adult body size in Drosophila
Source: EMBO Rep. 2025 Sep 9;26(22):5397–430. doi: 10.1038/s44319-025-00574-7 (PMC12635341; doi:10.1038/s44319-025-00574-7)
Supplement: Supplementary file 1 — Appendix [file 44319_2025_574_MOESM1_ESM.pdf]

Appendix for:

"Macrophage metabolic reprogramming during dietary stress influences adult body size in *Drosophila*"

Table of content

Description		Page no. s
Appendix Fig. S1:	Whole-genome transcriptomics of immune cells and larvae exposed to high sugar diet.	2, 3
Appendix Fig. S2:	Steady-state metabolite and isotope flux analysis with $U^{13}C$-pyruvate into TCA metabolites and lactate from immune cells on RF and Ct.HSD.	4, 5
Appendix Fig. S3:	Lipid uptake via Croquemort is required to sustain HSD dietary stress.	6-8
Appendix Fig. S4:	Model for "Macrophage metabolic reprogramming in dietary stress influences adult body size in *Drosophila"*	9-11
Appendix Table S1:	List of final candidate genes identified from *RNAi* screen.	12-15
Appendix Table S2:	GO term enrichment for differentially expressed genes in immune cells following 4 hours HSD (4h.HSD) treatment.	16-17
Appendix Table S3:	GO term enrichment for differentially expressed genes in immune cells following long term HSD (Ct.HSD) treatment.	18,19
Appendix Table S4:	Comparison of GO term categories between immune cells and whole larva.	20
Appendix Table S5:	List of Q1/Q3 parameters and retention time (R_T) of metabolites analysed by LC/MS.	21,22

Appendix Fig. S1

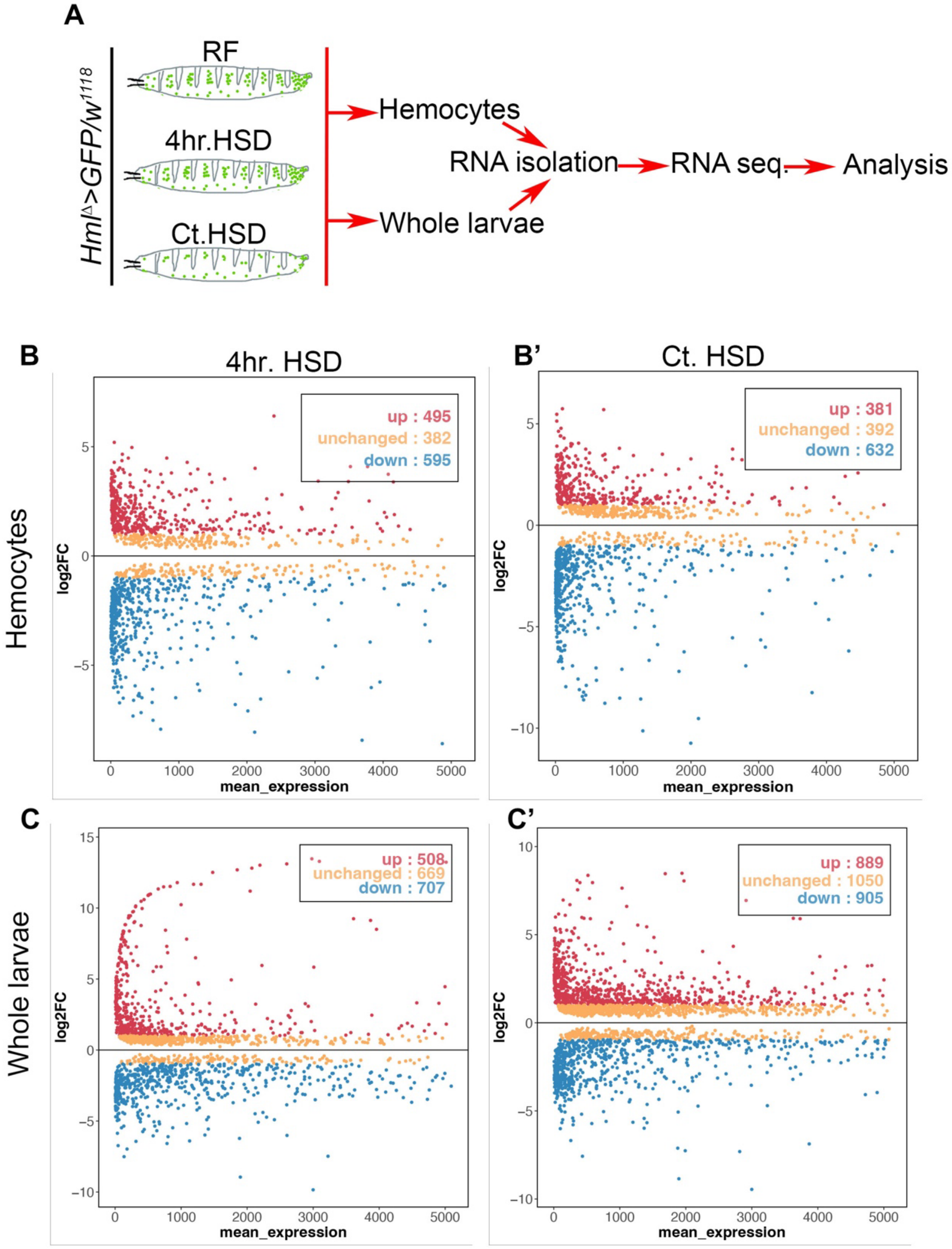

Appendix Figure S1

Whole-genome transcriptomics of immune cells and larvae exposed to high sugar diet

(A) Schematic representation of transcriptomics performed on immune cells and whole larvae in *Hml*$^{\Delta}$*GFP>/w*^{1118} (Control, RF), *Hml*$^{\Delta}$*GFP>/w*^{1118} (4hr.HSD) and *Hml*$^{\Delta}$*GFP>/w*^{1118} (Ct.HSD) dietary conditions. See methods for details.

(B,B') Scatter plots depicting the distribution of differentially expressed genes in immune cells and (C,C') whole larvae fed on 4hr.HSD and Ct.HSD respectively compared to larvae fed on RF.

Appendix Fig. S2

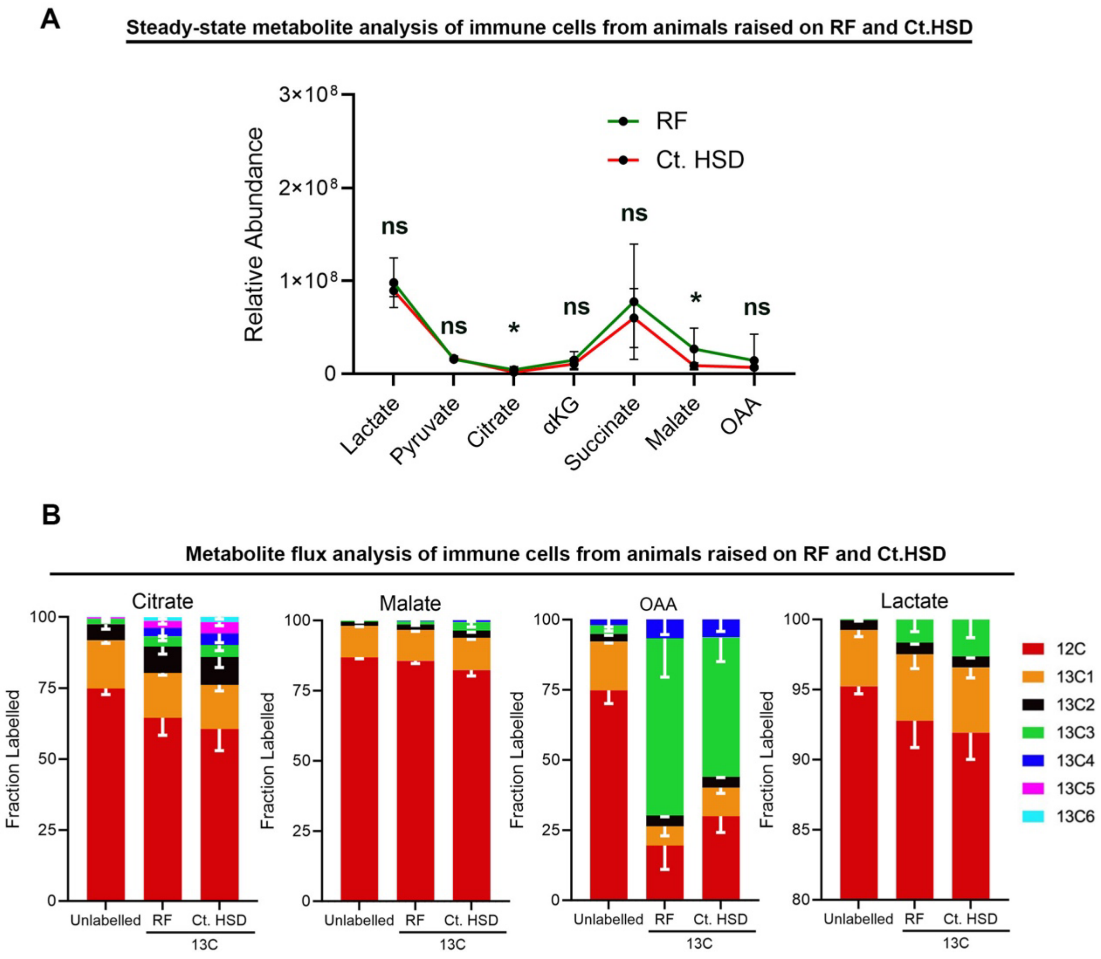

Appendix Figure S2

Steady-state metabolite and isotopic flux analysis with U^{13}C-pyruvate into TCA metabolites and lactate from immune cells on RF and Ct.HSD.

(A) Mass spectrometry analysis of steady-state lactate and TCA metabolites in immune cells between *Hml$^{\Delta}$GFP>/w^{1118}* (Control, RF, n=12) and *Hml$^{\Delta}$GFP>/w^{1118}* (Ct.HSD, n=14) do not show any overall change. Lactate (RF, n=12, Ct.HSD, n=14, P = 0.2956), Pyruvate (RF, n=12, Ct.HSD, n=14, P = 0.4952), Citrate (RF, n=12, Ct.HSD, n=14, P = 0.0316), αKG (RF, n=12, Ct.HSD, n=14, P = 0.1920), Succinate (RF, n=12, Ct.HSD, n=14, P = 0.3898), Malate (RF, n=12, Ct.HSD, n=14, P = 0.0178), OAA (RF, n=12, Ct.HSD, n=14, P = 0.3892).

(B) Distribution of labeled U^{13}C pyruvate in TCA metabolites and lactate in *Hml$^{\Delta}$GFP>/w^{1118}* (Control, RF) and *Hml$^{\Delta}$GFP>/w^{1118}* (Ct.HSD) conditions showing the respective fraction label incorporation from U^{13}C pyruvate in citrate (UN, n=7, RF, n=11 and HSD, n=13) , αKG (UN, n=7, RF, n=12 and HSD, n=14), malate (UN, n=7, RF, n=12 and HSD, n=14), OAA (UN, n=7, RF, n=11 and HSD, n=14) and lactate (UN, n=7, RF, n=11 and HSD, n=14). 13C label incorporation in unlabeled (UN) condition is shown to indicate the natural isotopic abundance.

Data information: "n" is the number of biological replicates analysed. Statistical comparisons were performed against regular food (RF); asterisks mark statistically significant differences (*P < 0.05; **P < 0.01; ***P < 0.001; ****P < 0.0001). RF and Ct.HSD indicate larvae fed regular food diet and constitutive high sugar diet, respectively. The statistical analysis applied for (A) is unpaired t test (Welch's correction).

Appendix Fig. S3

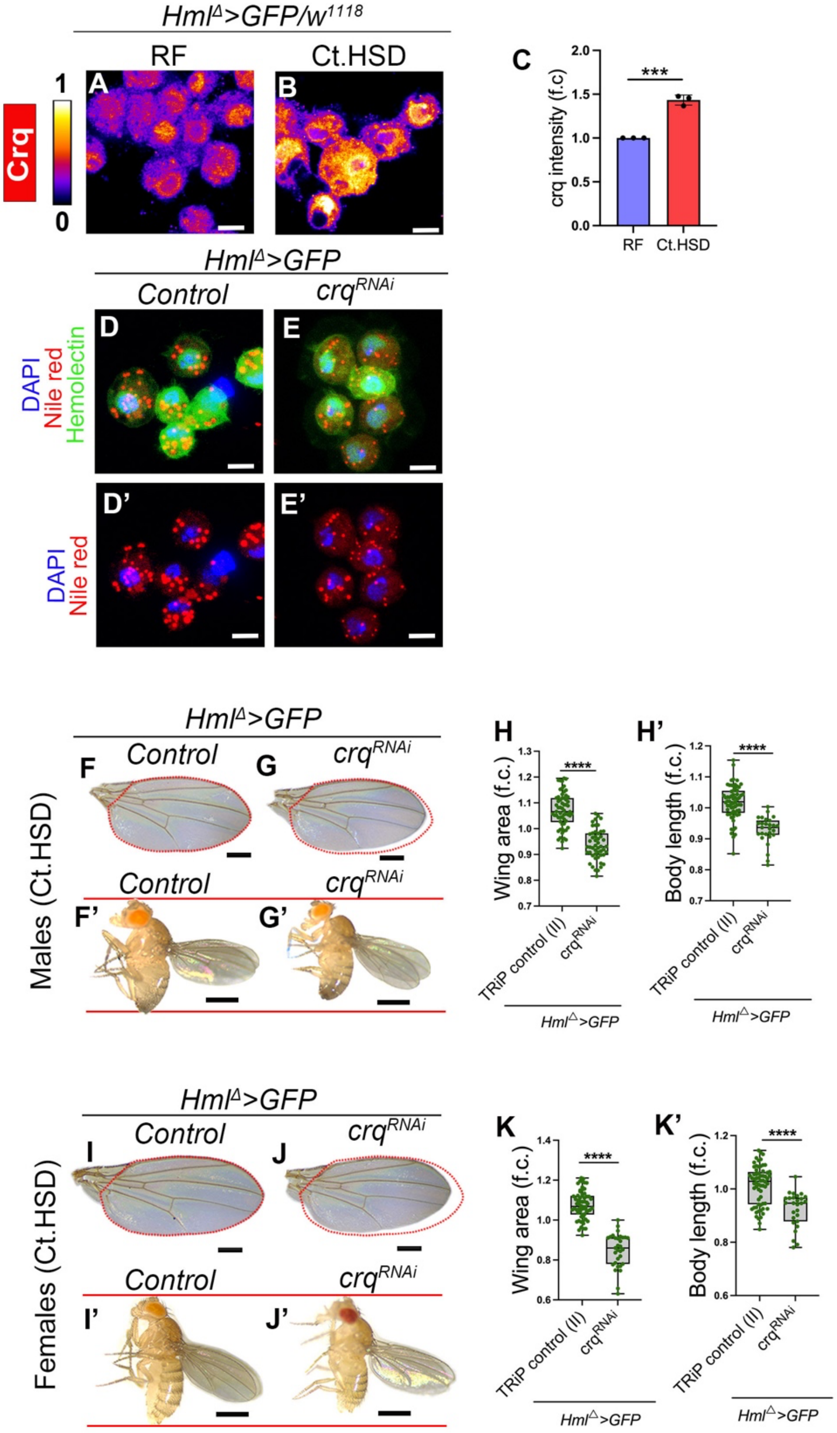

Appendix Figure S3

Lipid uptake via Croquemort is required to sustain HSD dietary stress

(A,B) Representative images of immune cells stained to visualize Croquemort protein expression. Compared to crq protein levels in (A) RF Control (*Hml*$^{\Delta}$*>GFP/w*^{1118}*),* (B) long-term HSD condition (Ct.HSD) the expression of crq is increased dramatically. (C) Relative quantification of crq protein expression, RF (N=3, n=30) and Ct.HSD (N=3, n=30, P =0.0002).

(D-E') Representative images of immune cells on Ct.HSD stained to show lipid droplets (Nile Red, red) and Hemolectin (Hml) marked in green represents immune cell marker. Compared to lipid levels seen in (D,D') HSD *Control* (*Hml*$^{\Delta}$*>GFP/w*^{1118}), (E,E') knockdown of immune cell *crq* function (*Hml*$^{\Delta}$*>GFP/crq*^{RNAi}) leads to decrease in lipid droplets inside the cells.

(F-J') Representative images of adult male-female flies and wings to show size phenotype on Ct.HSD upon manipulating lipid uptake transporter *crq* in immune cells. As compared to (F,F') in males and (I,I') in females HSD *Control* (*Hml*$^{\Delta}$*>GFP/w*^{1118}), (G, G'), (J,J') knockdown of *crq* (*Hml*$^{\Delta}$*>GFP/crq*^{RNAi}) led to reduction in body size and wing area.

(H) Quantification of male wing area in *Hml*$^{\Delta}$*>GFP/crq*^{RNAi} (N=3, n=50, $P < 0.0001$) in comparison to *Hml*$^{\Delta}$*>GFP/TRiP (II) control* (Ct.HSD, N=3, n=62).

(H') Quantification of male body length in *Hml*$^{\Delta}$*>GFP/crq*^{RNAi} (N=3, n=27, $P < 0.0001$) in comparison to *Hml*$^{\Delta}$*>GFP/TRiP (II) control* (Ct.HSD, N=3, n=64).

(K) Quantification of female wing area in *Hml*$^{\Delta}$*>GFP/crq*^{RNAi} (N=3, n=31, $P < 0.0001$) in comparison to *Hml*$^{\Delta}$*>GFP/TRiP (II) control* (Ct.HSD, N=3, n=64).

(K') Quantification of female body length in *Hml*$^{\Delta}$*>GFP/crq*^{RNAi} (N=3, n=27, $P < 0.0001$) in comparison to *Hml*$^{\Delta}$*>GFP/TRiP (II) control* (Ct.HSD, N=3, n=64).

Data information: DNA is stained with DAPI (blue), immune cells are shown in green (*Hml*$^{\Delta}$*>UAS-GFP*). Croquemort (crq) staining is shown in spectral mode in panels (A, B). Nile red staining to mark lipids is shown in red in panels (D-E'). Scale bar: 5μm for immune cells, 0.5mm for flies and 0.25mm for wings. In quantification graph (C) each dot represents an experimental repeat while in graphs (H,H') and (K,K') each dot represents an animal. Except for panel (A-C) where comparisons are with respect to Control on RF, in all other panels comparison for significance is with respective background control on Ct.HSD. Asterisks mark statistically significant differences (*$P < 0.05$; **$P < 0.01$; ***$P < 0.001$; ****$P < 0.0001$). The statistical analysis applied for (c) is unpaired t-test, for other panels (H,H',K,K') Mann-Whitney test. N indicates the number of independent biological replicates, and n refers to the total number of animals analysed. Only right wing from each adult fly was selected for quantification. The differences in wing areas or fly body lengths in panels is indicated with a red dotted line or two horizontal red lines that highlight changes across genotypes. RF and Ct.HSD correspond to regular food and constitutive high sugar diet respectively. In bar graphs data are presented as mean ± SD. Box plots show the median (center line), 25th-75th percentiles (bounds of box), and whiskers extending to the minimum and maximum values; all individual data points are shown.

Appendix Fig. S4

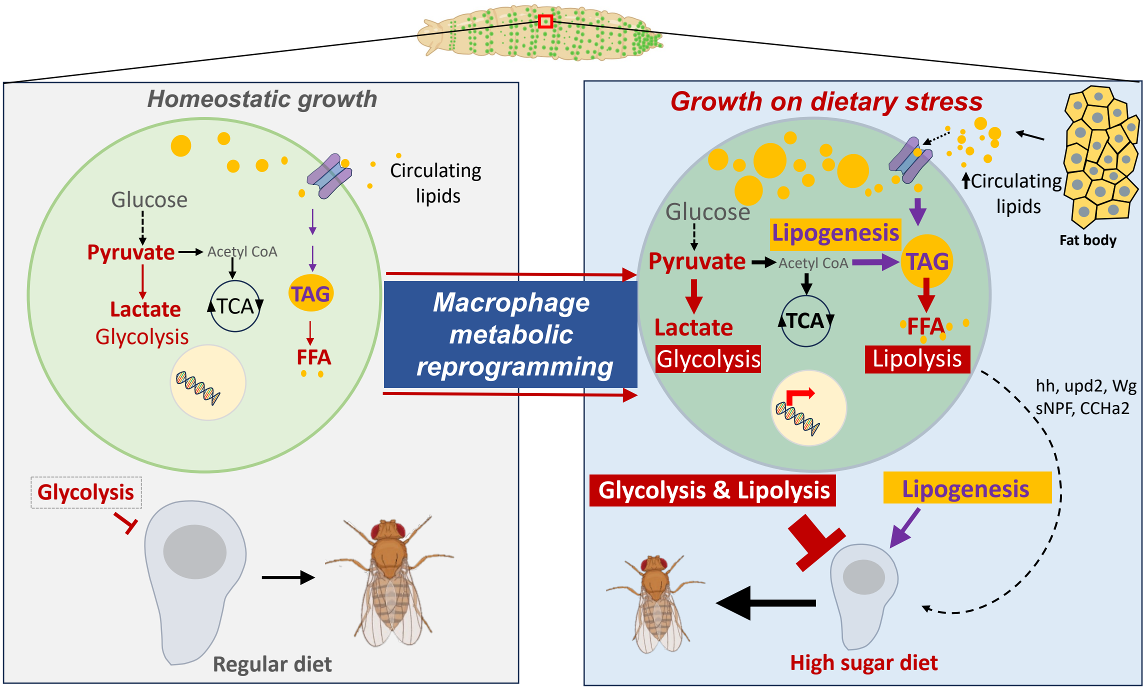

Appendix Fig. S4

Model "Macrophage metabolic reprogramming in dietary stress influences adult body size in *Drosophila*"

Model highlighting high sugar diet (HSD) induced macrophage metabolic reprogramming in *Drosophila* larvae affects adult growth. In regular food condition, macrophages maintain a low glycolytic state that sustains a basal level of systemic repression on growth of imaginal discs that underlies homeostatic growth in regular diet. In HSD, this exposure induces transcriptional rewiring of their metabolic state to activate an oxidative, lipogenic and lipolytic program (red arrow in the nucleus). Metabolically, this leads to increased cycling of pyruvate entry into the TCA cycle and also into lactate. The induction of a lipogenic shift (Purple arrows and yellow box is representation of lipogenic state, lipid droplets shown in yellow) is mediated by activation of *de novo* lipogenesis (ACC dependent), circulating hemolymph lipid uptake (most-likely fat body derived), and elevated TAG synthesis pathway. The resulting TAGs are also broken down through the lipolytic pathway (Free Fatty Acids, FFA) and this overall maintains the levels of TAG in the HSD immune cells. While increased TCA/oxidation (black arrows and circle) does not control growth, the heightened larval immune cell glycolytic capacity and lipolysis mounts an early growth inhibitory impact (red colour, signifies growth inhibitory states) specifically on imaginal disc development (red block arrow), while the lipogenic metabolic induction favors their growth (purple colour, signifies growth promoting states). However, the extent of lipogenesis is not sufficient to oppose the dominant output from catabolic events that leads to overall growth repression, and ultimately, the emerging adult fly size on HSD is small. Mechanistically, these metabolic state changes in blood cells, may engage

systemically using signaling ligands (dashed arrow from immune cells) like hedgehog (hh), unpaired (upd2), Wingless, (wg), CChamide2 (CCHa2), short neuropeptide F (sNPF) etc, which are identified candidates from the screen, but the details remain to be further deciphered.

Appendix Tables

Appendix Table S1: List of final candidate genes identified from RNA*i* screen

<table>
<tr><th>S. No.</th><th>Gene name</th><th>Annotation symbol</th><th>Allele name</th><th>Body size</th></tr>
<tr><td colspan="5">Transcription factor</td></tr>
<tr><td rowspan="2">1</td><td rowspan="2">Ecdysone-induced protein 75B (EIP75B)</td><td rowspan="2">CG8127</td><td>GLC01418</td><td>- -</td></tr>
<tr><td>JF02257</td><td>- -</td></tr>
<tr><td colspan="5">RNA Binding protein</td></tr>
<tr><td rowspan="2">2</td><td rowspan="2">Heterogeneous nuclear ribonucleoprotein at 98DE (Hrb98DE)</td><td rowspan="2">CG9983</td><td>JF01249</td><td>- -</td></tr>
<tr><td>HMS00342</td><td>- -</td></tr>
<tr><td colspan="5">Mitochondrial function</td></tr>
<tr><td rowspan="4">3</td><td rowspan="4">Parkin (park)</td><td rowspan="4">CG10523</td><td>GD5543</td><td>- - -</td></tr>
<tr><td>GD5543</td><td>- - -</td></tr>
<tr><td>JF01200</td><td>- -</td></tr>
<tr><td>HMS01651</td><td>NE</td></tr>
<tr><td colspan="5">Calcium responsive activity</td></tr>
<tr><td rowspan="2">4</td><td rowspan="2">Sarco/endoplasmic reticulum Ca(2+)-ATPase (SERCA)</td><td rowspan="2">CG3725</td><td>GD436</td><td>+ +</td></tr>
<tr><td>JF01948</td><td>+</td></tr>
<tr><td rowspan="2">5</td><td rowspan="2">Calcium/calmodulin-dependent protein kinase II (CaMKII)</td><td rowspan="2">CG18069</td><td>GD9506</td><td>- -</td></tr>
<tr><td>GL00237</td><td>-</td></tr>
<tr><td colspan="5">Autophagy</td></tr>
<tr><td rowspan="2">6</td><td rowspan="2">Autophagy-related 13 (Atg13)</td><td rowspan="2">CG7331</td><td>HMS02028</td><td>+</td></tr>
<tr><td>GD12202</td><td>+</td></tr>
<tr><td rowspan="3">7</td><td rowspan="3">Autophagy-related 7 (Atg7)</td><td rowspan="3">CG5489</td><td>GD11671</td><td>- -</td></tr>
<tr><td>GD11671</td><td>- -</td></tr>
<tr><td>GD11671</td><td>NE</td></tr>
<tr><td colspan="5">Lipid metabolism</td></tr>
<tr><td rowspan="2">8</td><td rowspan="2">Glycerol-3-phosphate acyltransferase 4 (Gpat4)</td><td rowspan="2">CG3209</td><td>KK108132</td><td>- -</td></tr>
<tr><td>GD3566</td><td>- -</td></tr>
</table>

			HMS02556	NE
9	Lipid storage droplet-2 (Lsd-2)	CG9057	***GD14108***	**++**
			HMS00629	**+**
			HMS01292	**+**
10	Oxysterol-binding protein-related protein 8 (Orp8)	CG42668	***HMS01223***	**++**
			KK111315	**+**
			KK108193	NE
11	Brummer (bmm)	CG5295	***JF01946***	**++**
			EY06194	**- -**
			EY06577	**- -**
12	LDL receptor protein 1 (LRP1)	CG33087	***HMS02875***	**- -**
			JF01628	**-**
13	Sugarbabe (sug)	CG3850	***HMC03866***	**- -**
			GD11090	**- -**
			JF02347	NE
**GPCR pathway**				
14	Pyrokinin 2 receptor 1 (PK2-R1)	CG8784	***JF03303***	**++**
			GD685	**+**
15	Leucine-rich repeat-containing G protein-coupled receptor 3 (Lgr3)	CG31096	***JF03217***	**+**
			HMC04196	**+**
16	Muscarinic Acetylcholine Receptor, A-type (mAChR-A)	CG4356	***JF02725***	**- -**
			<u>*GD630*</u>	**-**
**Notch pathway**				
17	Notch (N)	CG3936	***HMS00001***	**- -**
			<u>*GD144*</u>	**- -**
			HMS00009	**++**
			JF01693	NE
18	Kuzbanian (kuz)	CG7147	***UAS-kuz.DN***	**+++**
			VSH330255	**++**
			HMS05424	**-**
**Wnt pathway**				

19	Frizzled (fz)	CG17697	***JF01481***	**- -**
			GD4614	**- -**
20	α-catenin (α-cat)	CG17947	***GL00636***	**- -**
			HMS00317	**- -**
			GD8808	NE
**Hh pathway**				
21	Smoothened (smo)	CG11561	***GL01472***	**++**
			JF02363	**+**
			GD577	NE
**Secreted ligands**				
22	unpaired 2 (upd2)	CG5988	*HMS00901*	**- -**
			HMS00948	**- -**
23	Pigment-dispersing factor (Pdf)	CG6496	***GD17520***	**- -**
			GD17520	**- -**
			GD299	NE
24	CCHamide-2 (CCHa2)	CG14375	***KK111272***	**- -**
			HMC04565	**- -**
25	Insulin-like peptide 4 (Ilp4)	CG6736	***JF01346***	**++**
			HMS02660	**+**
			HMS00547	NE
26	Insulin-like peptide 5 (Ilp5)	CG33273	***HMS00548***	**- -**
			GD16039	**- -**
			JF01347	NE
27	Secreted decoy of InR (Sdr)	CG3837	***HMS02444***	**- -**
			GL00178	**- -**
28	Hedgehog (hh)	CG4637	***GD6242***	**++**
			JF01804	**+**
**Stress response**				
29	Superoxide dismutase 1 (Sod1)	CG11793	***GD7385***	**+**
			GL01016	**+**
**Cytoskeletal dynamics**				

30	Septin 4 (Septin4)	CG9699	***JF01592***	**- - -**
			GL01287	**- - -**
			GD1322	NE

Appendix Table S2: GO term enrichment for differentially expressed genes in immune cells following 4 hours HSD (4h.HSD) treatment

Go category/sub-category	P value	Fold Enrichment	Genes
**Down regulated**			
**Molting cycle**	0.000262	4.165894737	*E, Fz, Lcp1,* ***Ecr, Pk, RK, Sgs1, Sgs3, Sgs4, Sgs5, Sgs7, Sgs8, Eig71Ee, Ple, Hug, Sgs5bis, Spok***
**Biological adhesion**	0.008214	2.500537057	
Cell-cell adhesion			*PlexA, Pollux,* ***Amalgam, Zye, Impl2***
Cell migration			*Mew, Itgaps4, Itgbn*
**Cellular response to ecdysone**	0.025857	7.351578947	***Ecr, Blimp-1, Utx, Hr4, Br, Lkrsdh***
**Response to external stimulus**	0.049943	1.5227936	
JAK-STAT pathway			*Tot A, Tot C, Listericin*
Toll/Imd pathway			*BomS6, Drsl2, PGRP-SC1b, PGRP-SC2, PGRP-SC1a*
Defence response to bacteria			*BomS6, Drsl2, PGRP-SC1b, PGRP-SC2, PGRP-SC1a*
Melanization			*PPO3*
**Up regulated**			
**Small molecule metabolic process**	4.82E-08	2.353927	

Amino acid metabolism			*DAT, Path, Gad1, Faa* ***GLS***
Sugar metabolism			*Slv, Fbp* ***Men-b, Mpc1, LManV***
Lipid metabolism			*Abcd3 , Lpr, ACC, Hydr2* ***Gpdh1, Hydr1, Lipin,, Hmgcr, Pisd***
**Glutathione metabolic process**	0.004684	5.388889	*GstD7, GstZ2, GstE8, GstE5, GstE3, GstE10, Gclc*
**Regulation of cell division**	0.018031	5.173333	*Plcxd* ***Polo, Klu Idgf1, Feo,Tum, Chinmo, Scra***
**Wound healing**	0.021609	3.67931	*Kst, RhOGEF3*

Appendix Table S3: GO term enrichment for differentially expressed genes in immune cells following long term HSD (Ct.HSD) treatment

Go category/sub-category	P value	Fold Enrichment	Genes
**Down regulated**			
**Molting cycle**	0.002848761	3.68184514	*Ddc, Idgf4*<br>***Ecr, Pu, Rk, Sgs1, Sgs3, Sgs4, Sgs5, Sgs7, Sgs8, Eig71Ee, Ple,, Hug, Sgs5bis, Spok***
**Cellular response to ecdysone**	0.02996159	6.903459638	***Ecr, Blimp-1, Utx, Hr4, Br, Lkrsdh***
**Biological adhesion**	0.031825867	2.254190902	
Cell-cell adhesion			***Amalgam, Zye, Impl2***
**Up regulated**			
**Small molecule metabolic process**	0.000120691	2.22701993	
Amino acid metabolism			*Ass*<br>***Gls***
Sugar metabolism			***Men-b, Mpc1, LManV***
Lipid metabolism			*Bgm*<br>***Gpdh1, Hydr1, Lipin, Hmgcr,, Pisd***
**Regulation of cell division**	0.000256117	8.277333333	*Sti, aurb, Incenp*<br>***Polo, Klu, Idgf1, Feo, Tum, Chinmo,, Scra***

**Mitotic sister chromatid segregation**	0.000402545	4.656	*CycB, Ncd, Polo, Rod, Klp67A, Pav, Barr, aurB, Feo, CG15740, Ndc80, Nnf1A, Mink, Cmet, Incenp*
**Fatty acid biosynthetic process**	0.002735695	4.837402597	*Bgm, CG12512, Hydr1, CG18609, CG8534, CG9459, CG30008, fa2h, CG33110, Alc*

Appendix Table S4: Comparison of GO term categories between immune cells and whole larva

GO category/pathways	Hemocytes	p values	Whole larvae	p values
Exposure to 4 hours high sugar diet				
**Biological adhesion**	Down	0.008214	Up	0.00099282
**Response to external stimulus**	Down	0.049943	Down	0.0056554
**Metabolic process**	Up	4.82E-08	Down	0.00012302
**Regulation of cell division**	Up	0.018031	Up	0.02940038
Exposure to constitutive high sugar diet				
**Metabolic process**	Up	0.000120691	Down	0.00146718
**Developmental pathways**	None		Up	5.8869E-26

Appendix Table S5: List of Q1/Q3 parameters and retention time (RT) of metabolites analysed by LC/MS

Metabolite	Q1/Q3	Retention Time (R_T)
Pyruvate	299.1 -> 91.1	6.354
Lactate	196.1 -> 91.1	3.032
Citrate	508.1 -> 91.1	5.329
α-ketoglutaric acid (αKG)	462.1 -> 91.1	6.224
Succinate	329.1 -> 91.1	4.477
Malate	345.1 -> 91.1	4.207
Oxaloacetate (OAA)	448.1 -> 91.1	7.357
Citrate [M+1]	509.1 -> 91.1	5.329
Citrate [M+2]	510.1 -> 91.1	5.329
Citrate [M+3]	511.1 -> 91.1	5.329
Citrate M+4]	512.1 -> 91.1	5.329
Citrate [M+5]	513.1 -> 91.1	5.329
Citrate [M+6]	514.1 -> 91.1	5.329
Lactate [M+1]	197.1 -> 91.1	3.032
Lactate [M+2]	198.1 -> 91.1	3.032
Lactate [M+3]	199.1 -> 91.1	3.032
Malate [M+1]	346.1 -> 91.1	4.207
Malate [M+2]	347.1 -> 91.1	4.207

Malate [M+3]	348.1 -> 91.1	4.207
Malate [M+4]	349.1 -> 91.1	4.207
OAA [M+1]	449.1 -> 91.1	7.357
OAA [M+2]	450.1 -> 91.1	7.357
OAA [M+3]	451.1 -> 91.1	7.357
OAA [M+4]	452.1 -> 91.1	7.357
