# Supplementary figures and images for: Macrophage metabolic reprogramming during dietary stress influences adult body size in Drosophila (part 1 of 2)
Source: EMBO Rep. 2025 Sep 9;26(22):5397–430. doi: 10.1038/s44319-025-00574-7 (PMC12635341; doi:10.1038/s44319-025-00574-7)

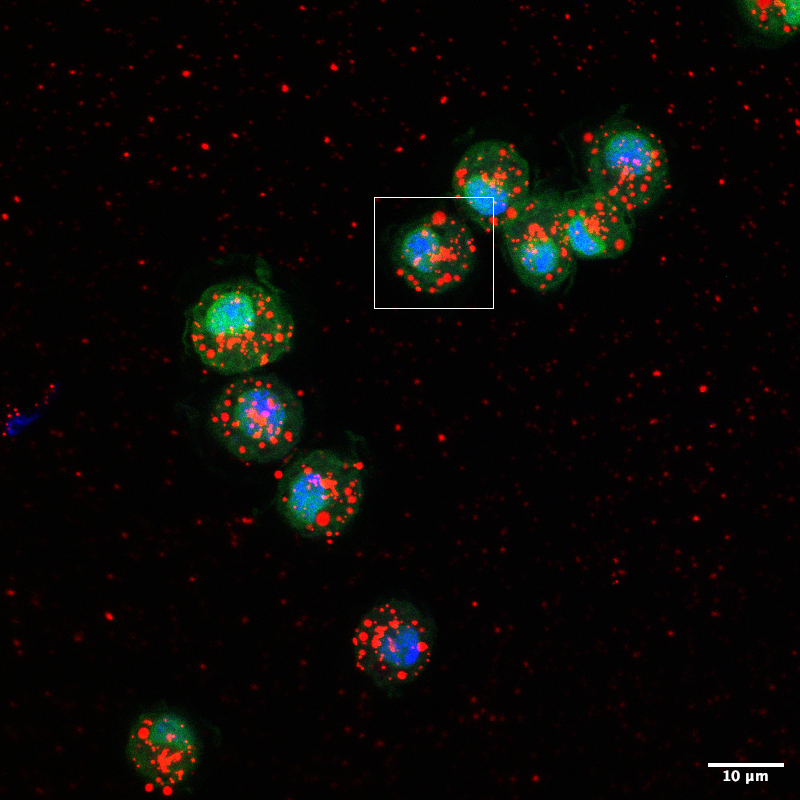

Supplement: Supplementary file 3 — Source data Fig. 1 [file 44319_2025_574_MOESM3_ESM.zip › Fig. 1/Fig. 1 h-h''/4hr.HSD_phagocytosis.tif]

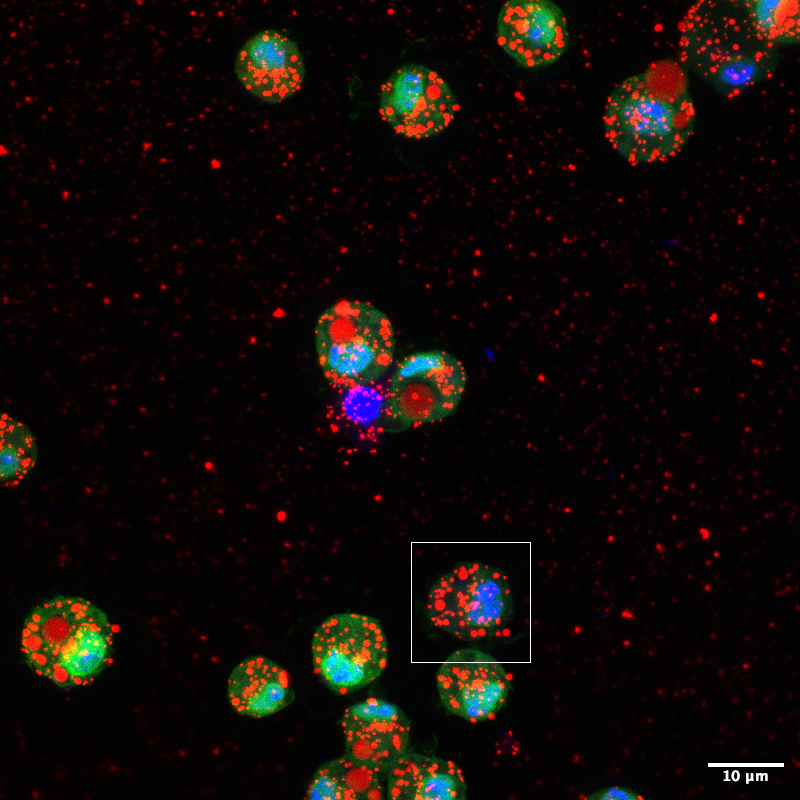

Supplement: Supplementary file 3 — Source data Fig. 1 [file 44319_2025_574_MOESM3_ESM.zip › Fig. 1/Fig. 1 h-h''/RF_phagocytosis.tif]

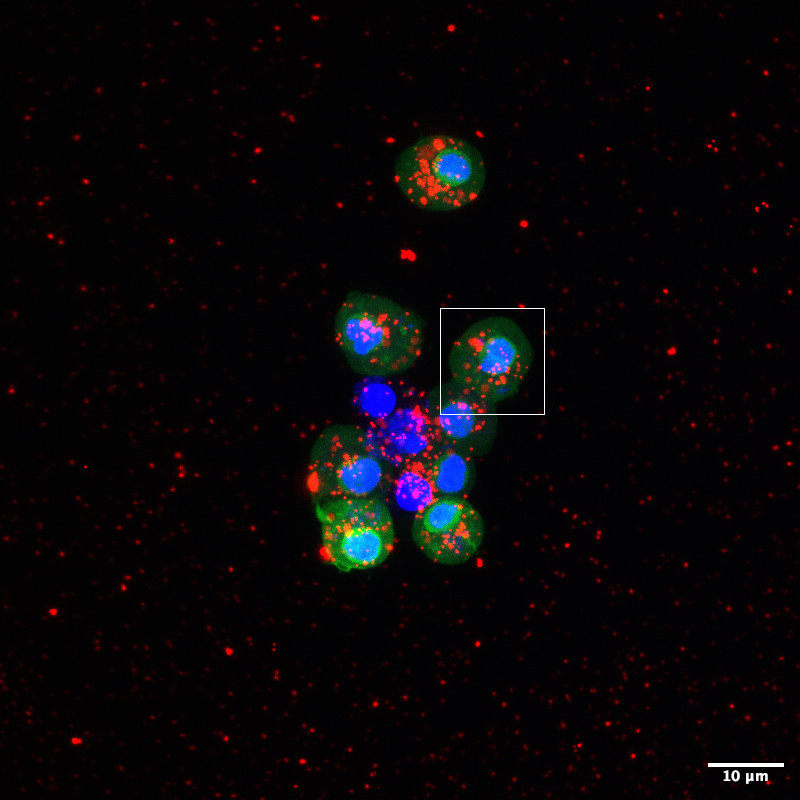

Supplement: Supplementary file 3 — Source data Fig. 1 [file 44319_2025_574_MOESM3_ESM.zip › Fig. 1/Fig. 1 h-h''/Ct.HSD_phagocytosis.tif]

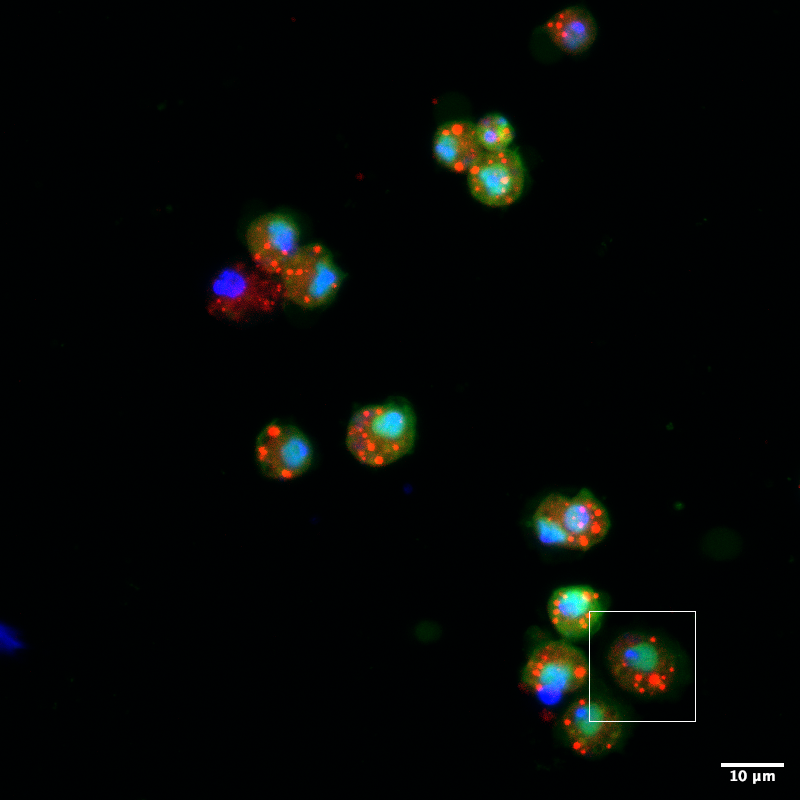

Supplement: Supplementary file 3 — Source data Fig. 1 [file 44319_2025_574_MOESM3_ESM.zip › Fig. 1/Fig. 1 g-g''/4hr.HSD_lipids.tif]

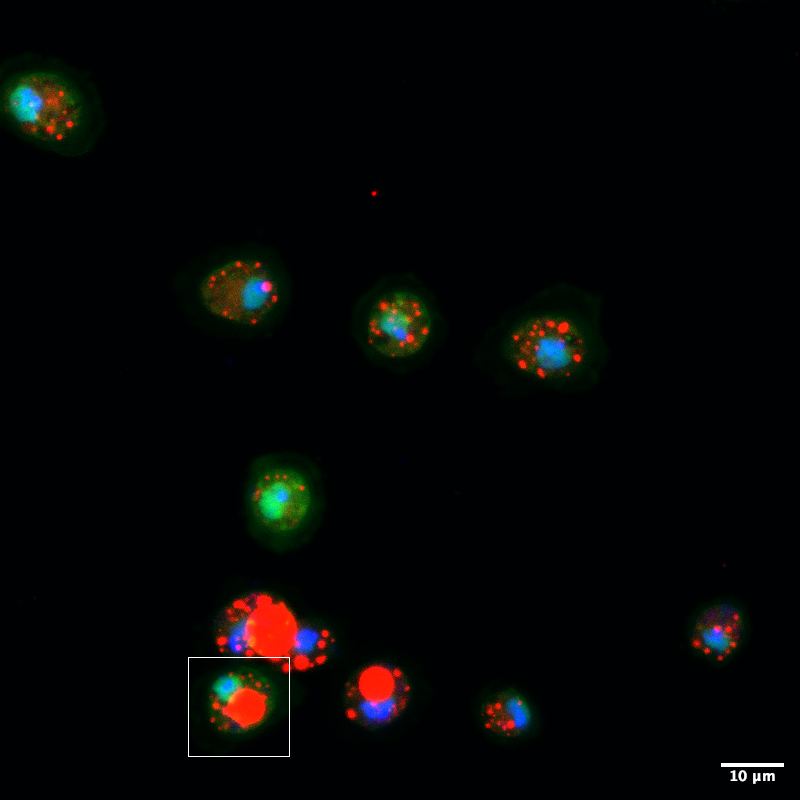

Supplement: Supplementary file 3 — Source data Fig. 1 [file 44319_2025_574_MOESM3_ESM.zip › Fig. 1/Fig. 1 g-g''/Ct.HSD_lipids.tif]

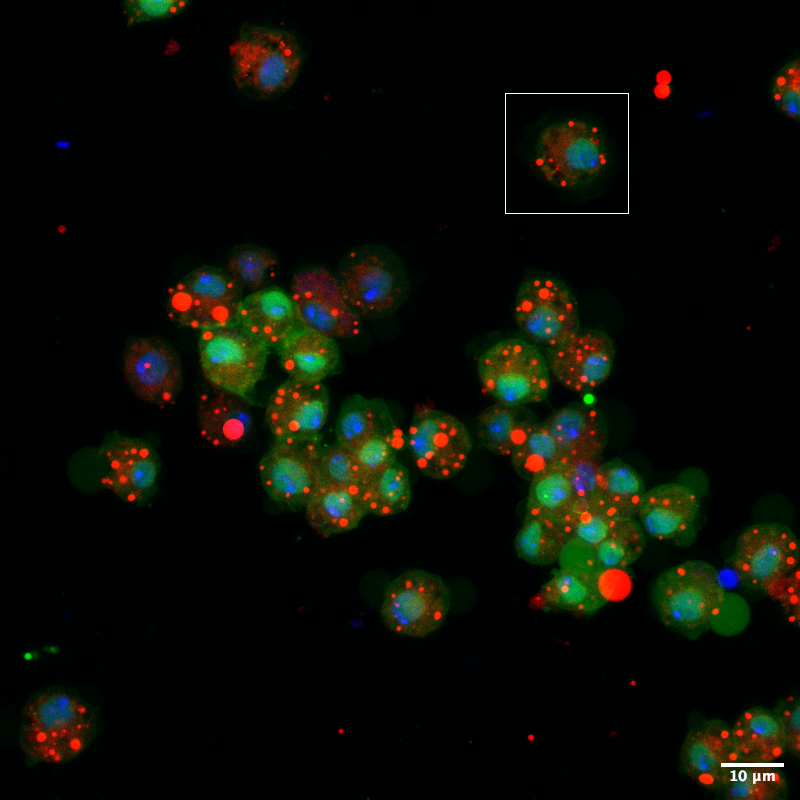

Supplement: Supplementary file 3 — Source data Fig. 1 [file 44319_2025_574_MOESM3_ESM.zip › Fig. 1/Fig. 1 g-g''/RF_lipids.tif]

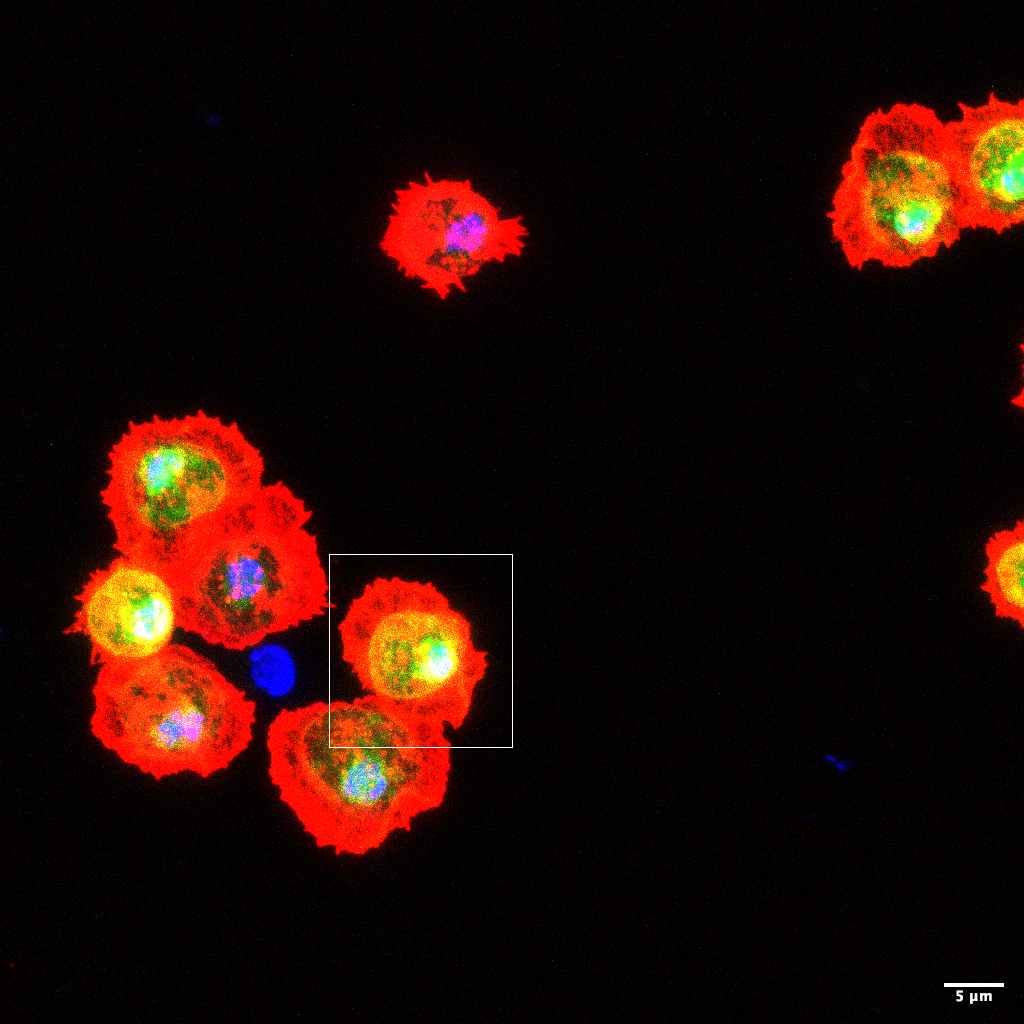

Supplement: Supplementary file 3 — Source data Fig. 1 [file 44319_2025_574_MOESM3_ESM.zip › Fig. 1/Fig. 1 j-j''/Ct.HSD_phalloidin.tif]

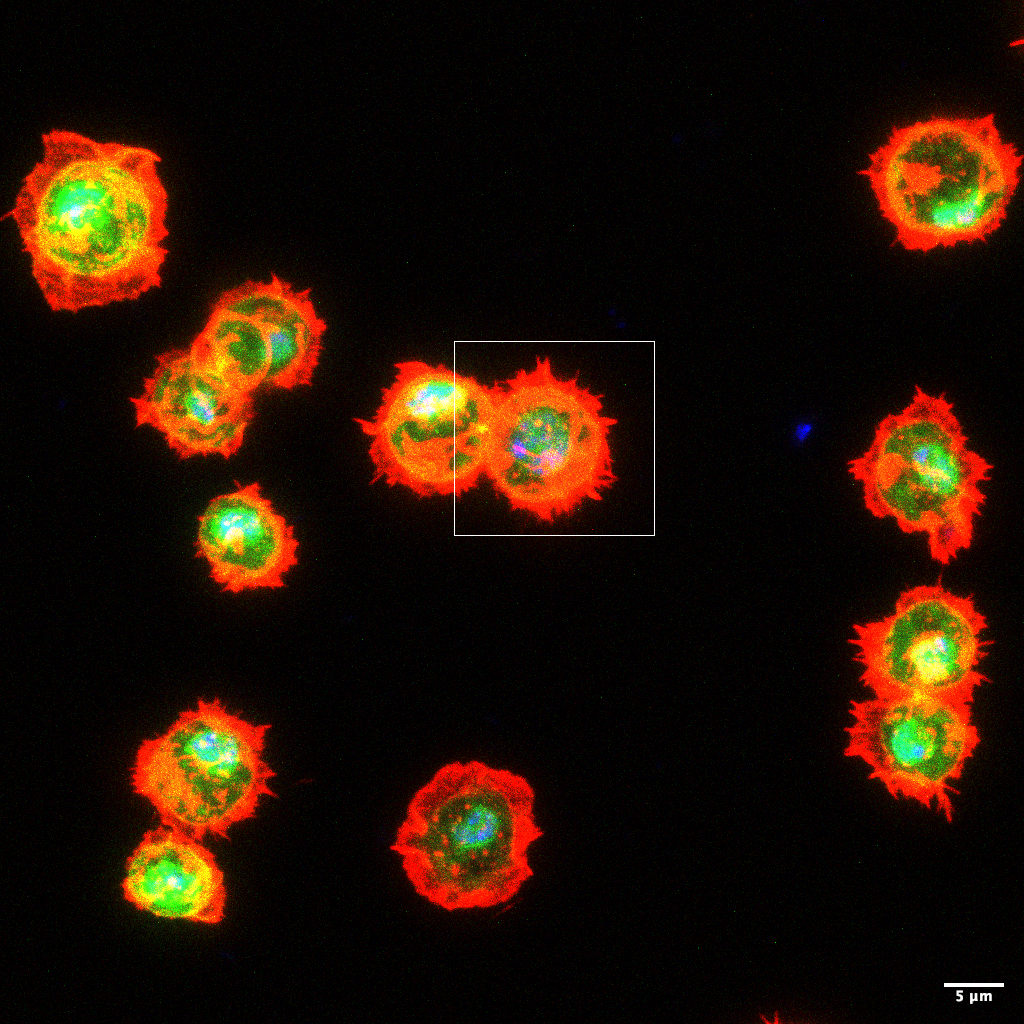

Supplement: Supplementary file 3 — Source data Fig. 1 [file 44319_2025_574_MOESM3_ESM.zip › Fig. 1/Fig. 1 j-j''/4hr.HSD_phalloidin.tif]

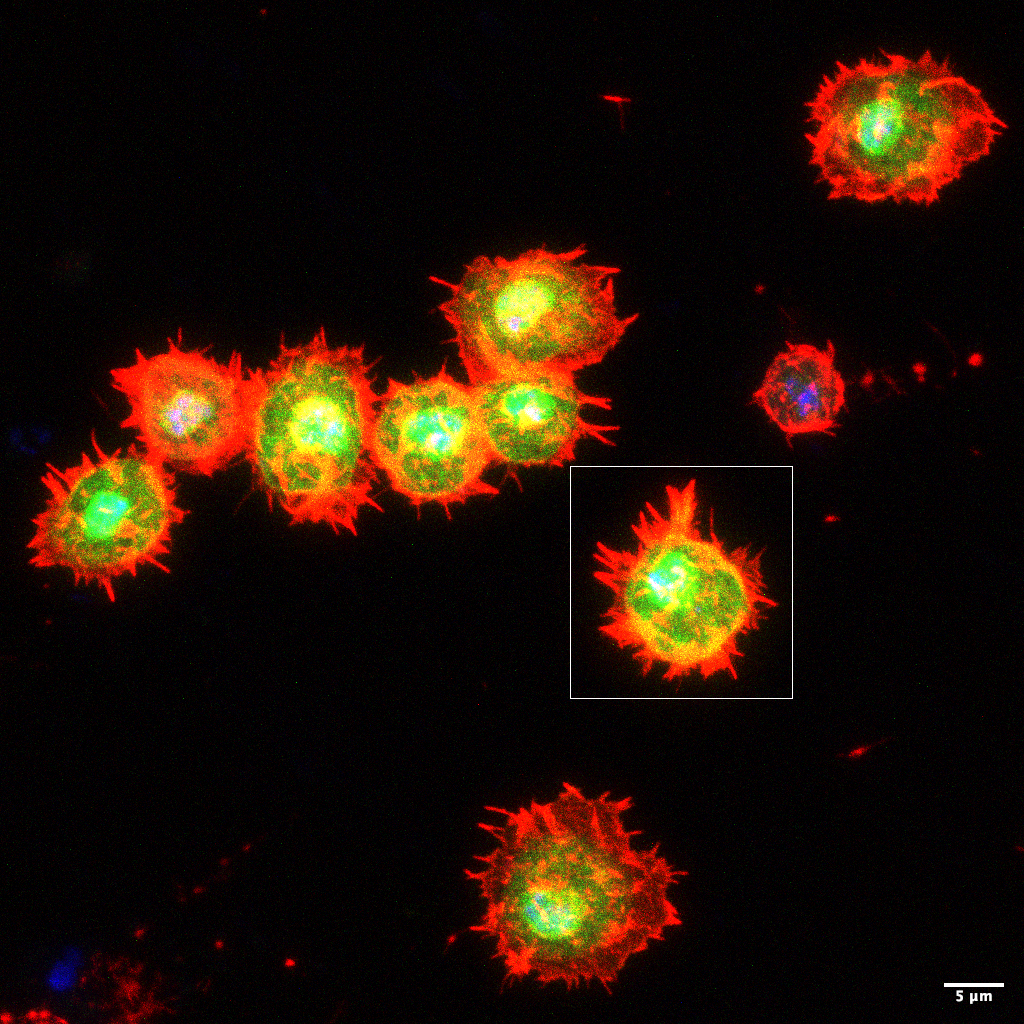

Supplement: Supplementary file 3 — Source data Fig. 1 [file 44319_2025_574_MOESM3_ESM.zip › Fig. 1/Fig. 1 j-j''/RF_phalloidin.tif]

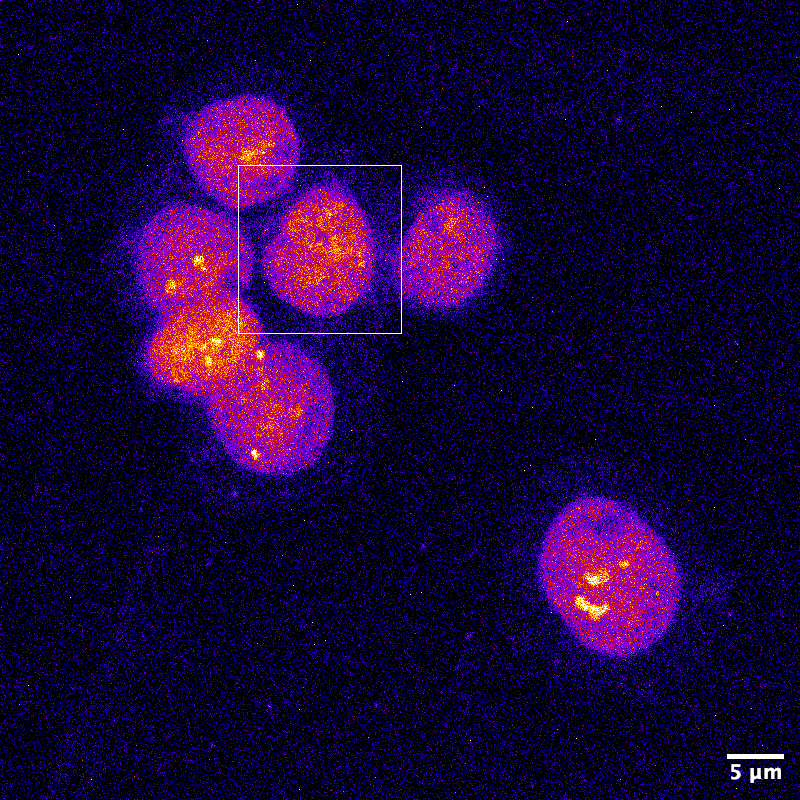

Supplement: Supplementary file 3 — Source data Fig. 1 [file 44319_2025_574_MOESM3_ESM.zip › Fig. 1/Fig. 1 e-e''/4hr.HSD_ROS.tif]

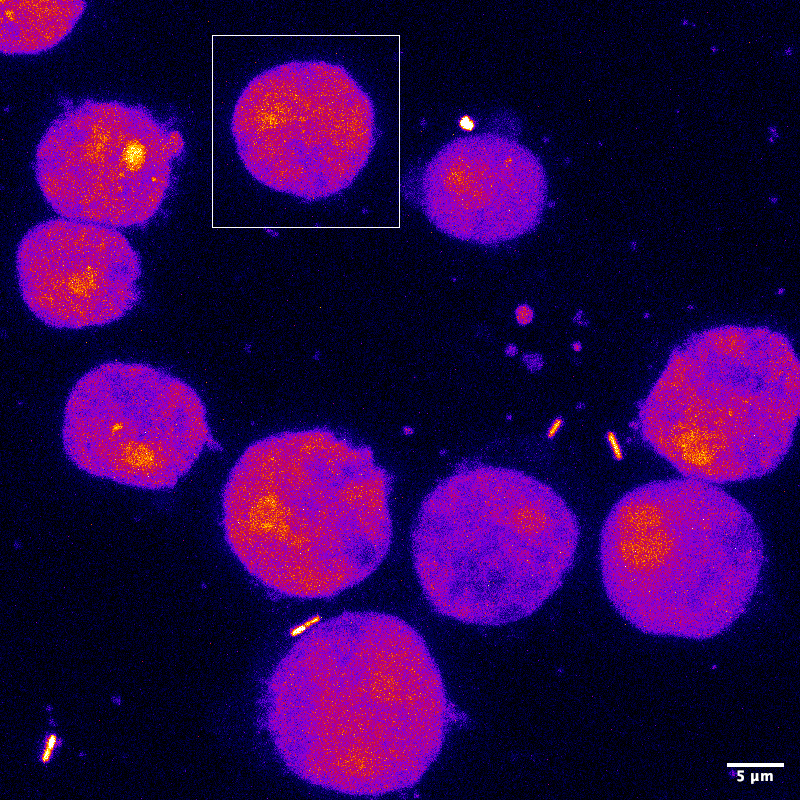

Supplement: Supplementary file 3 — Source data Fig. 1 [file 44319_2025_574_MOESM3_ESM.zip › Fig. 1/Fig. 1 e-e''/RF_ROS.tif]

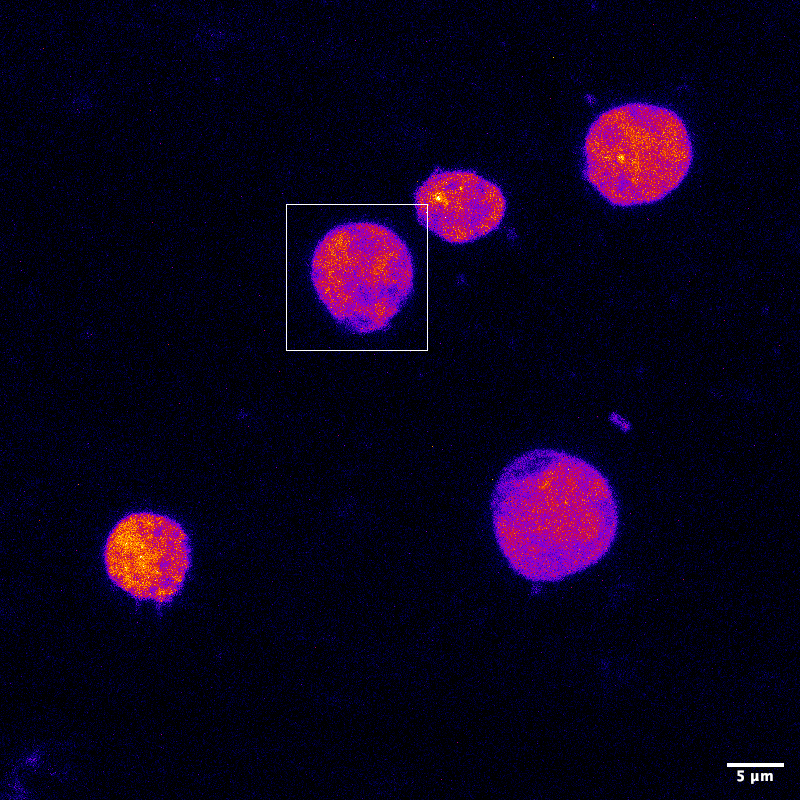

Supplement: Supplementary file 3 — Source data Fig. 1 [file 44319_2025_574_MOESM3_ESM.zip › Fig. 1/Fig. 1 e-e''/Ct.HSD_ROS.tif]

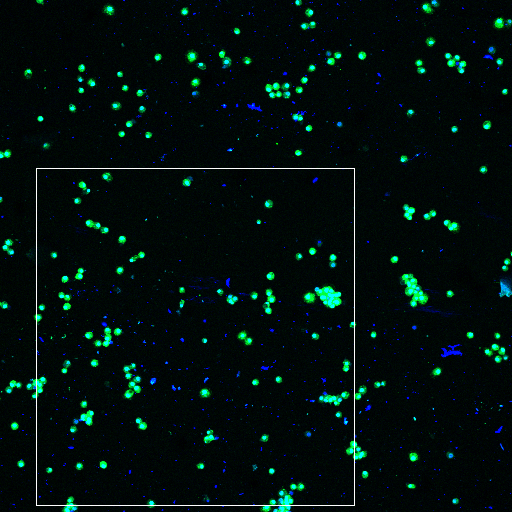

Supplement: Supplementary file 3 — Source data Fig. 1 [file 44319_2025_574_MOESM3_ESM.zip › Fig. 1/Fig. 1 a'-c'/4hr.HSD_bleeds.tif]

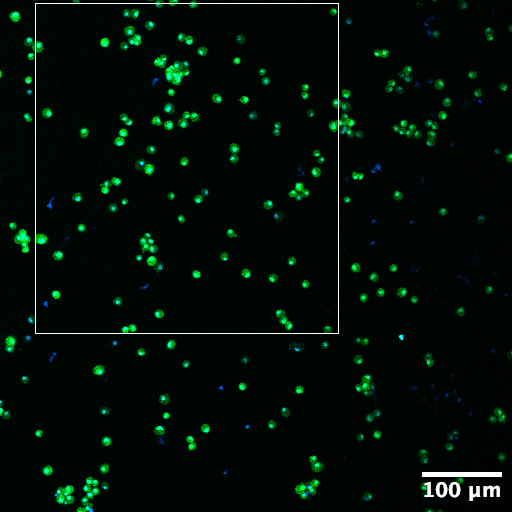

Supplement: Supplementary file 3 — Source data Fig. 1 [file 44319_2025_574_MOESM3_ESM.zip › Fig. 1/Fig. 1 a'-c'/RF_bleeds.tif]

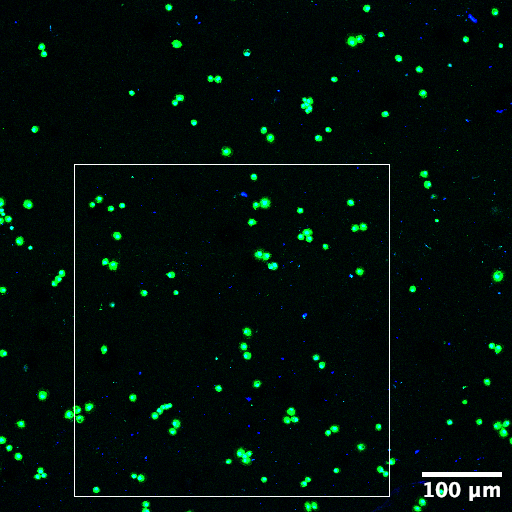

Supplement: Supplementary file 3 — Source data Fig. 1 [file 44319_2025_574_MOESM3_ESM.zip › Fig. 1/Fig. 1 a'-c'/Ct.HSD_bleeds.tif]

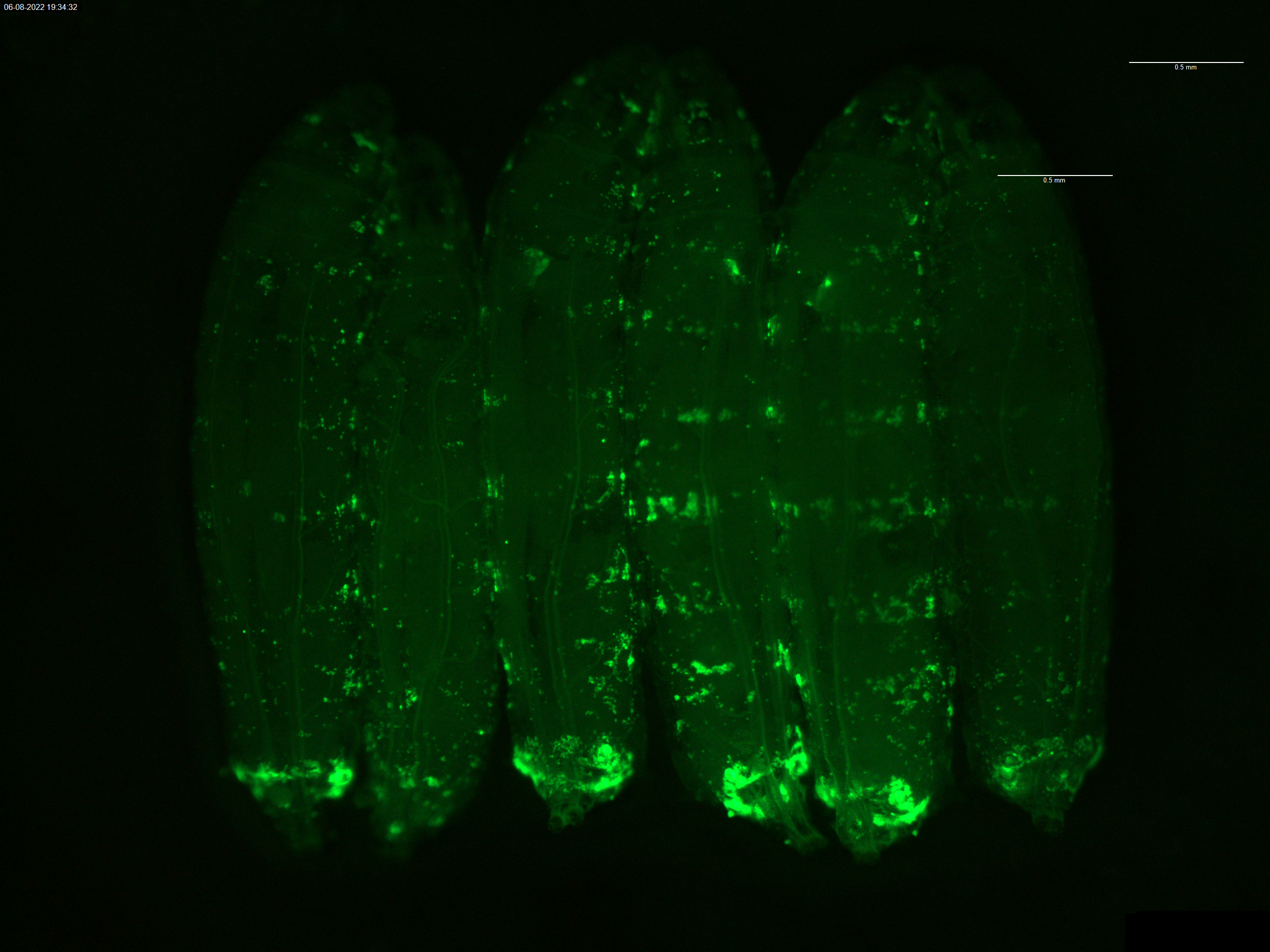

Supplement: Supplementary file 3 — Source data Fig. 1 [file 44319_2025_574_MOESM3_ESM.zip › Fig. 1/Fig. 1 a-c/Ct.HSD_Larvae.tif]

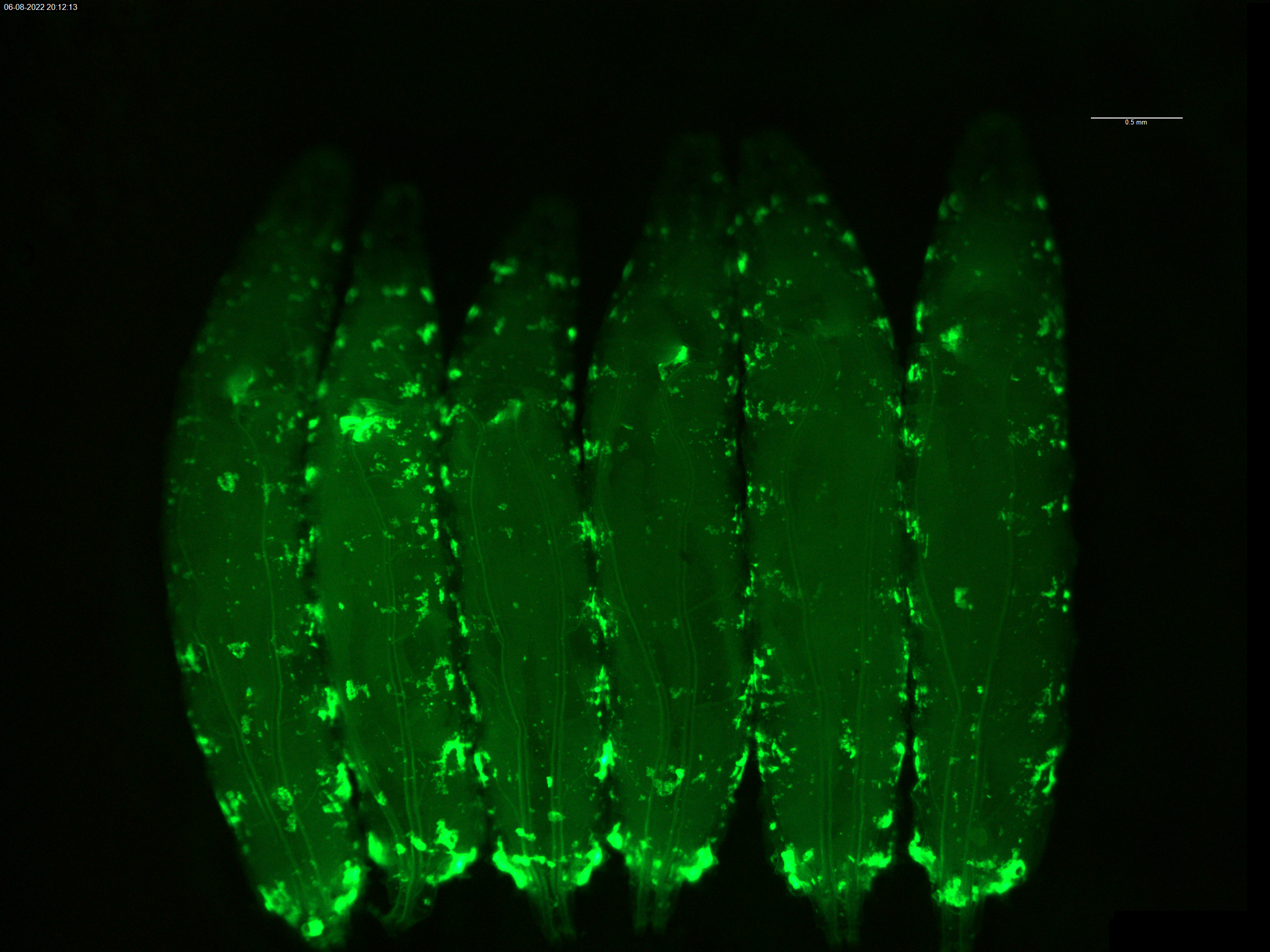

Supplement: Supplementary file 3 — Source data Fig. 1 [file 44319_2025_574_MOESM3_ESM.zip › Fig. 1/Fig. 1 a-c/RF_Larvae.tif]

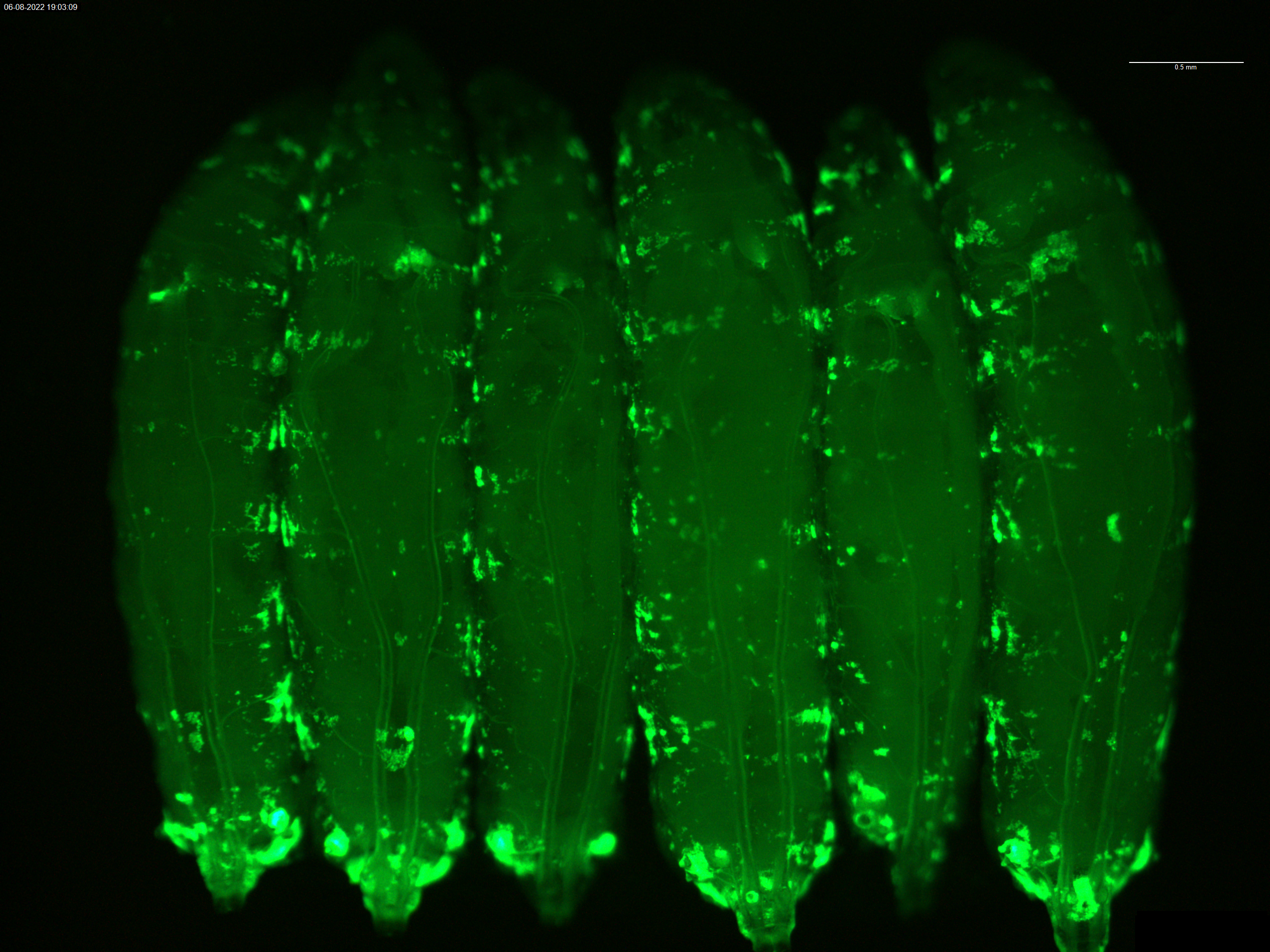

Supplement: Supplementary file 3 — Source data Fig. 1 [file 44319_2025_574_MOESM3_ESM.zip › Fig. 1/Fig. 1 a-c/4hr.HSD_Larvae .tif]

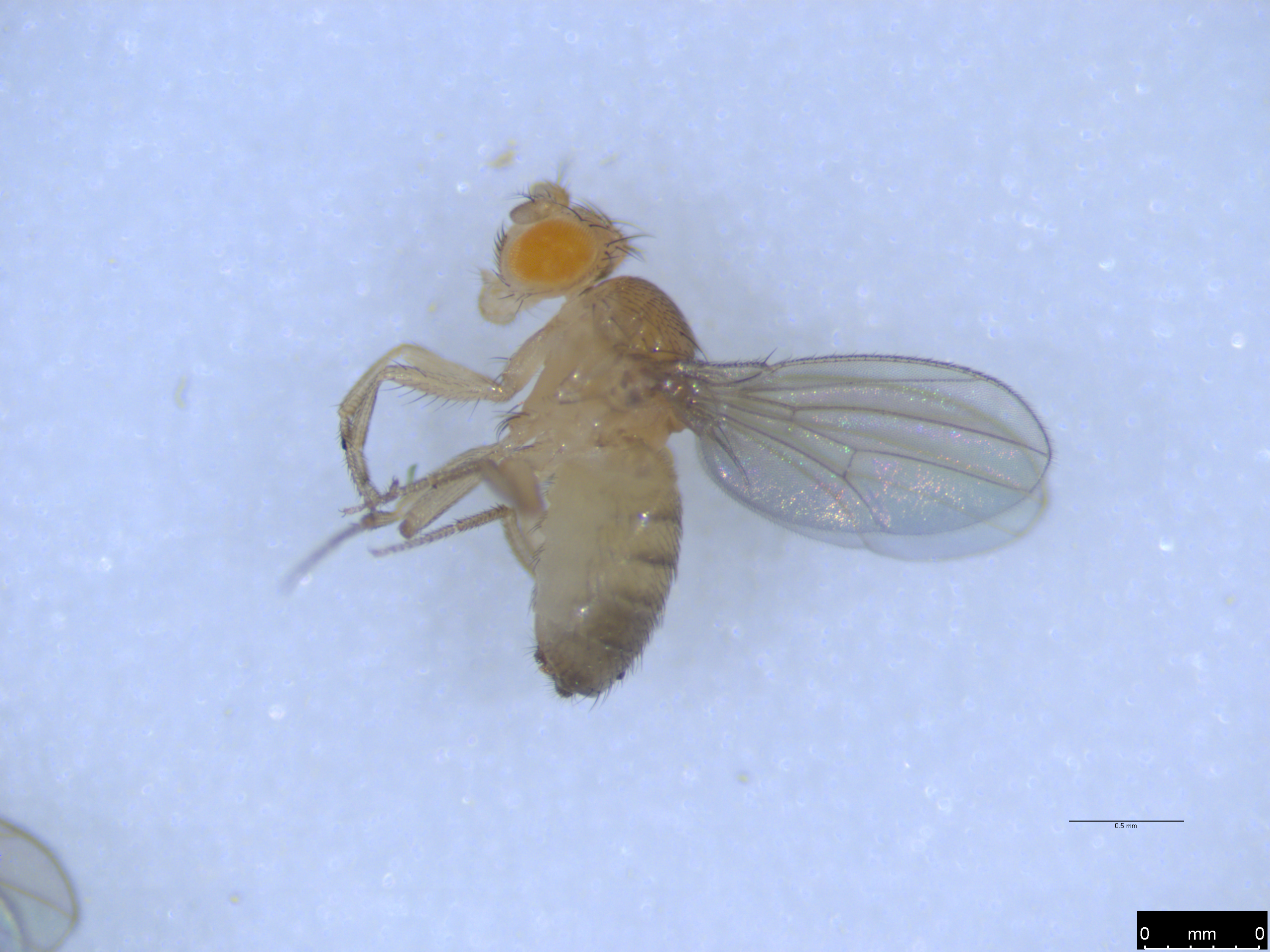

Supplement: Supplementary file 6 — Source data Fig. 4 [file 44319_2025_574_MOESM6_ESM.zip › Fig. 4/c'-f'/LdhRNAi_fly_Ct.HSD.tif]

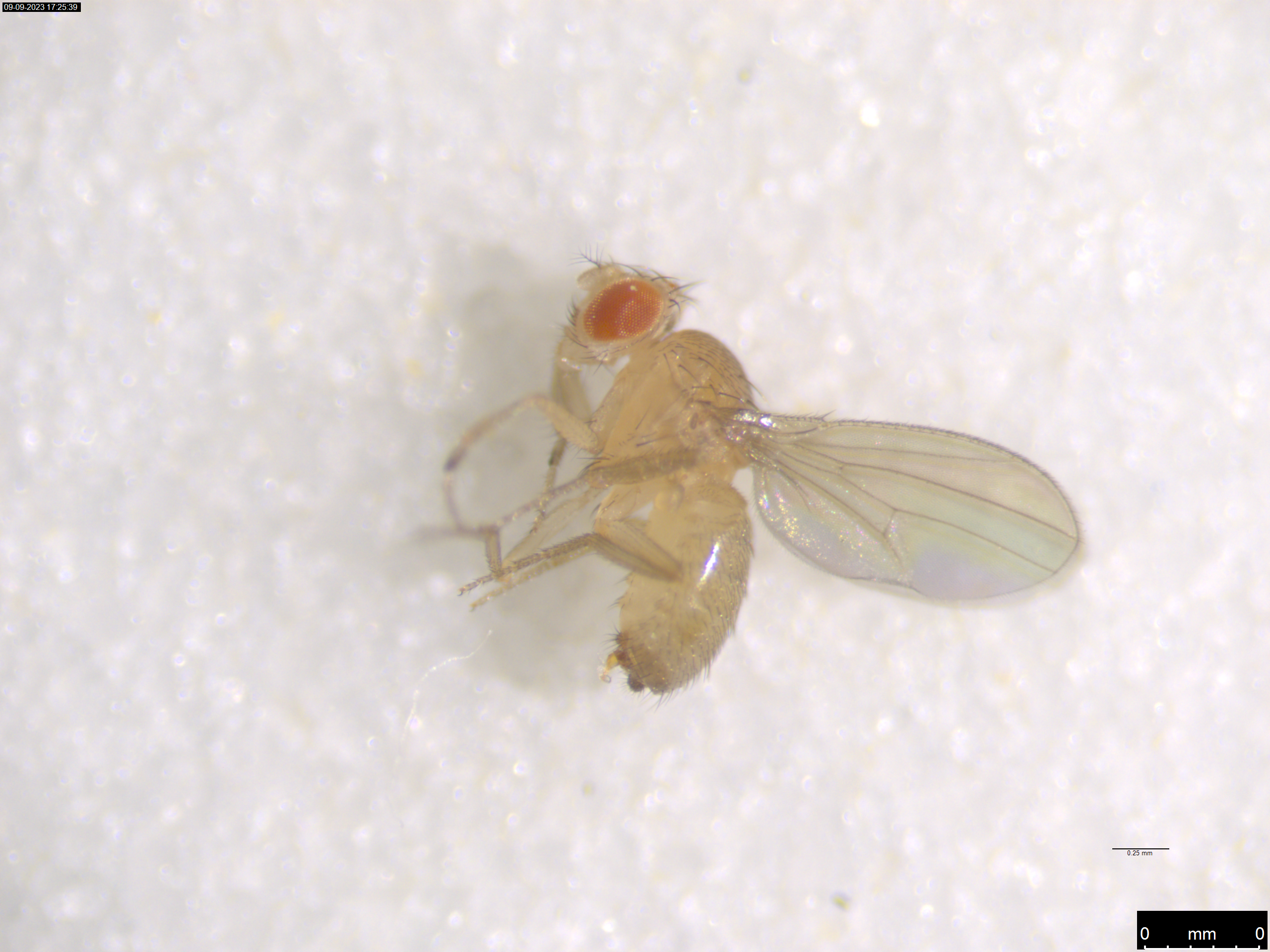

Supplement: Supplementary file 6 — Source data Fig. 4 [file 44319_2025_574_MOESM6_ESM.zip › Fig. 4/c'-f'/UAS-Ldh_fly_Ct.HSD.tif]

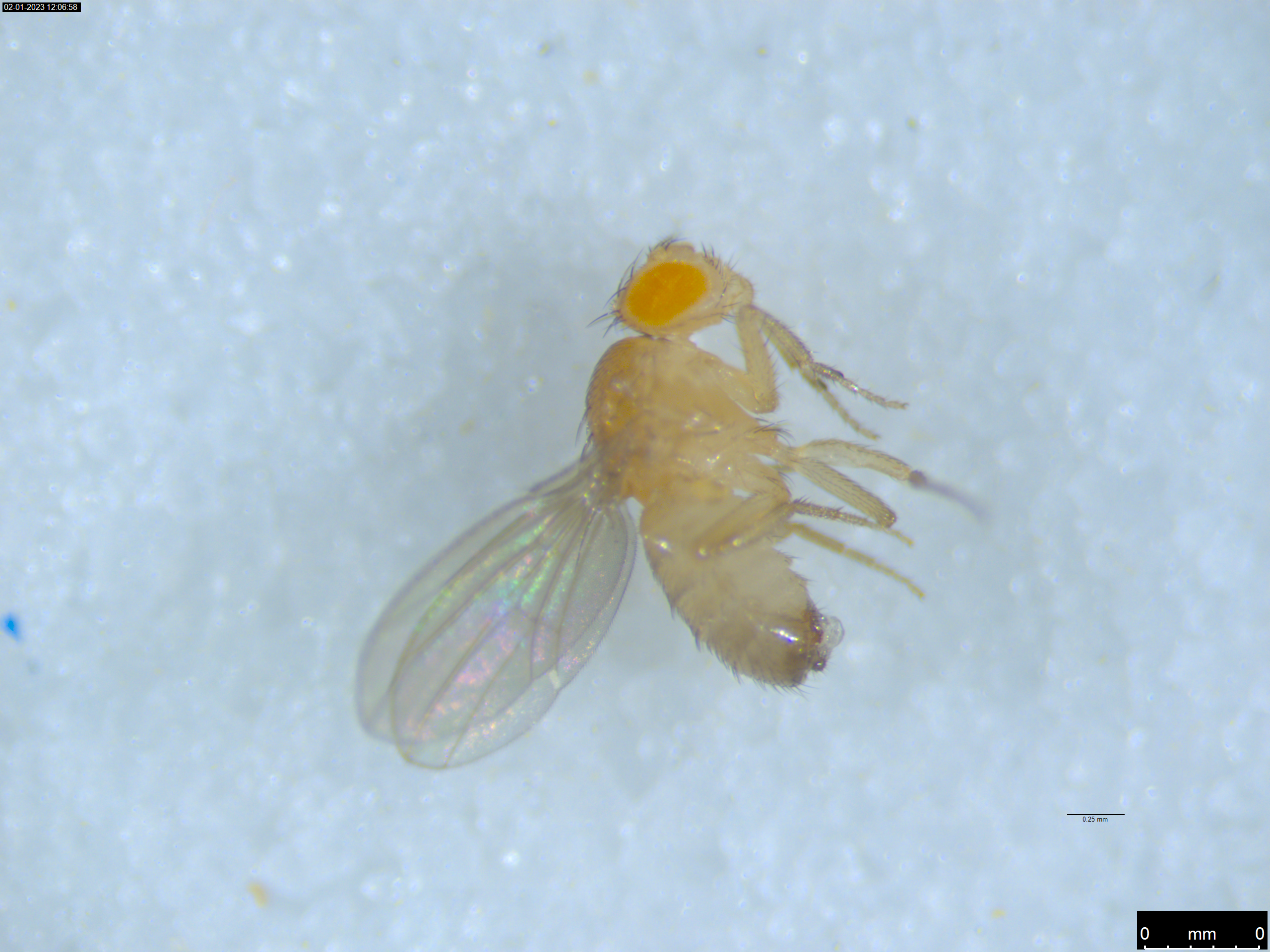

Supplement: Supplementary file 6 — Source data Fig. 4 [file 44319_2025_574_MOESM6_ESM.zip › Fig. 4/c'-f'/Control_fly_Ct.HSD.tif]

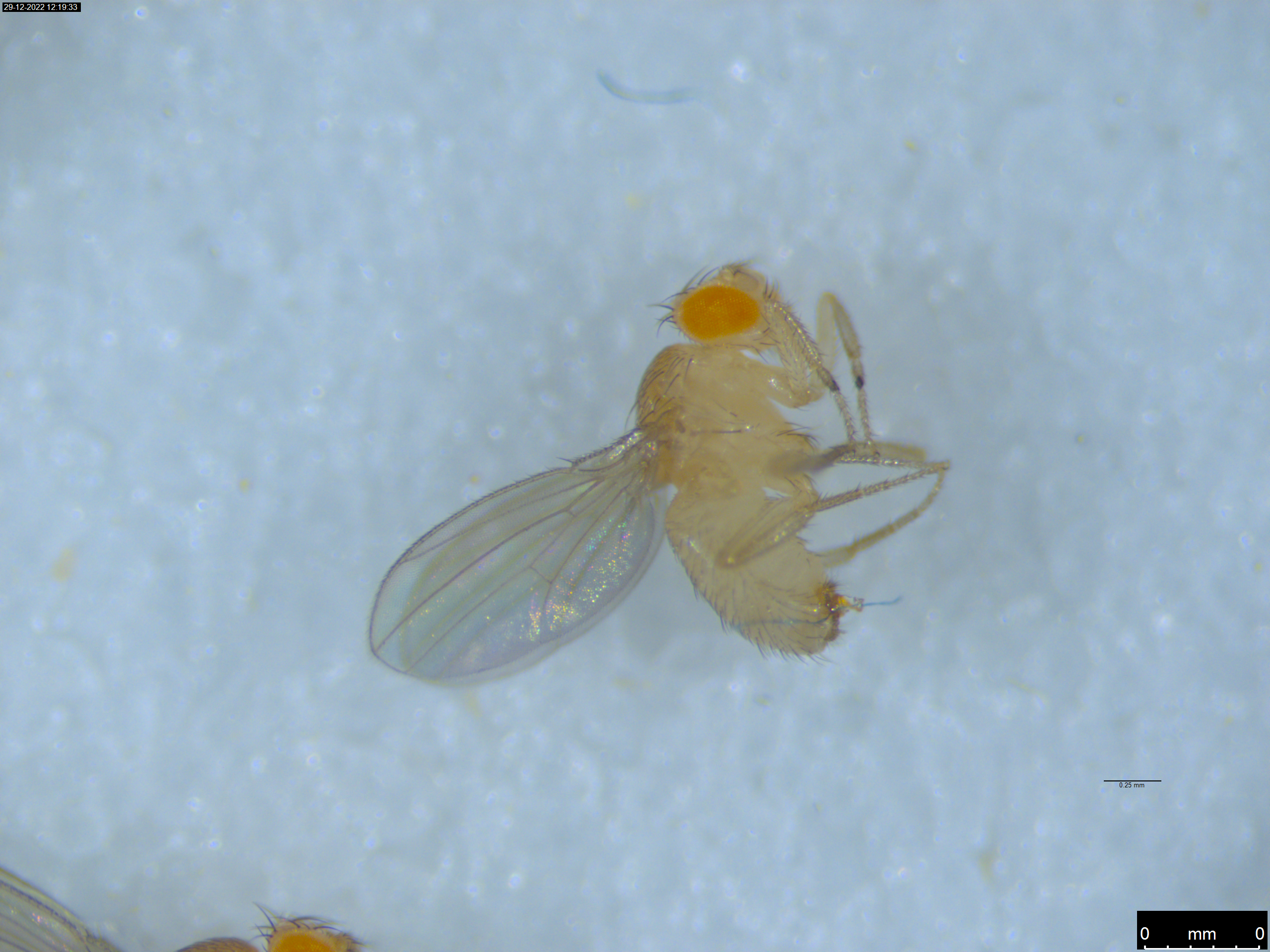

Supplement: Supplementary file 6 — Source data Fig. 4 [file 44319_2025_574_MOESM6_ESM.zip › Fig. 4/c'-f'/PdhaRNAi_fly_Ct.HSD.tif]

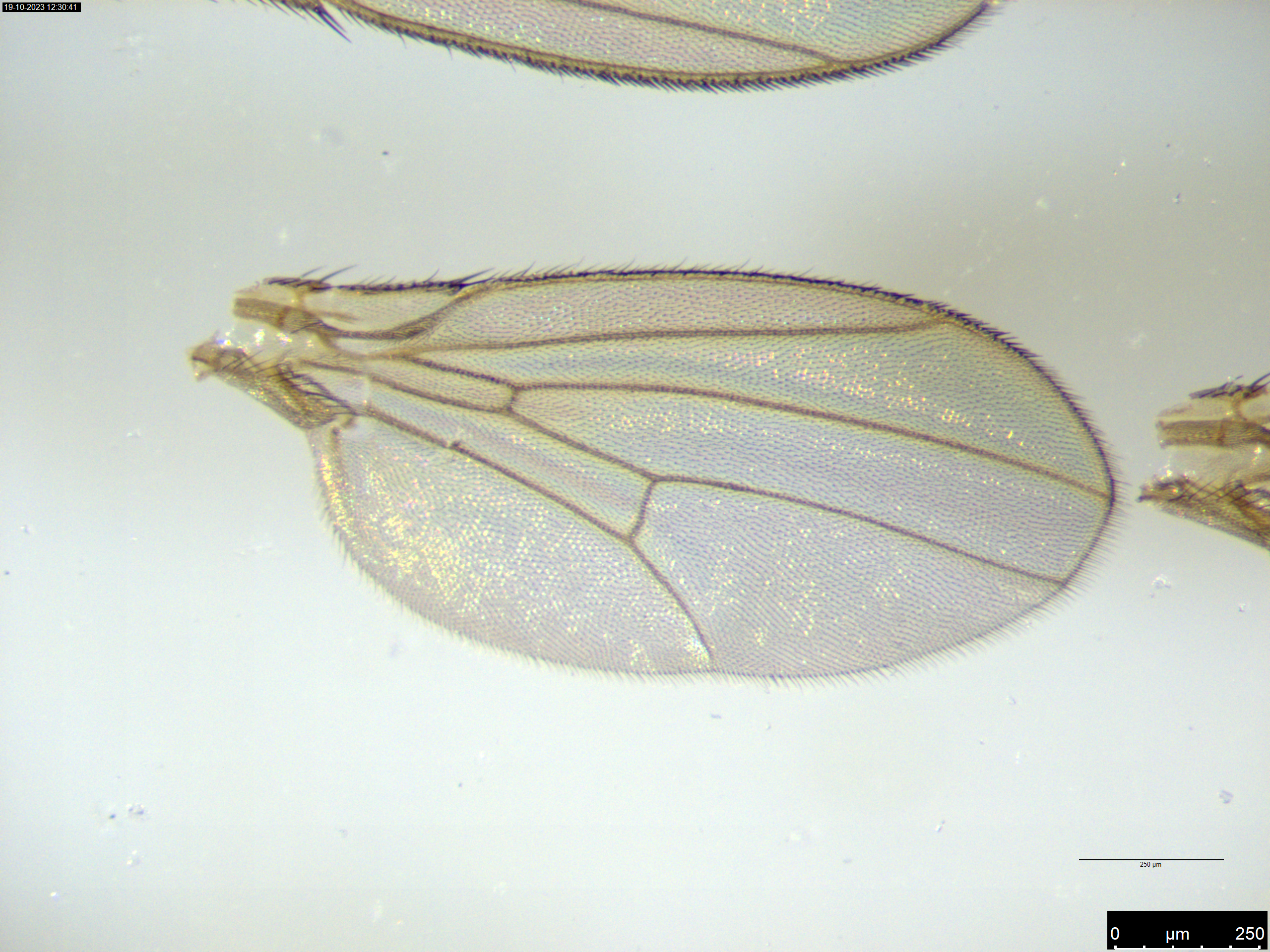

Supplement: Supplementary file 6 — Source data Fig. 4 [file 44319_2025_574_MOESM6_ESM.zip › Fig. 4/c-f/UAS-Ldh_wing_Ct.HSD.tif]

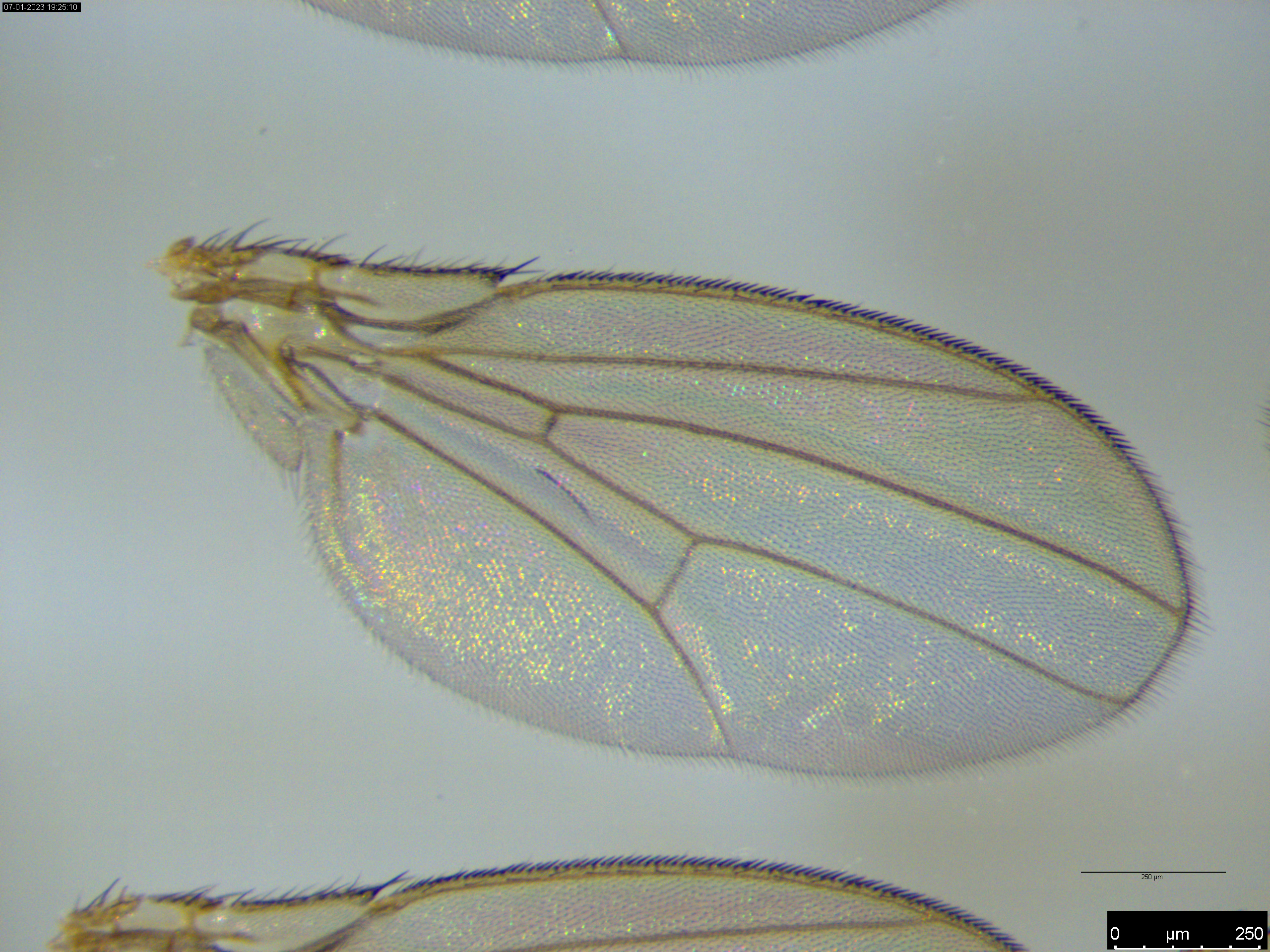

Supplement: Supplementary file 6 — Source data Fig. 4 [file 44319_2025_574_MOESM6_ESM.zip › Fig. 4/c-f/LdhRNAi_wing_Ct.HSD.tif]

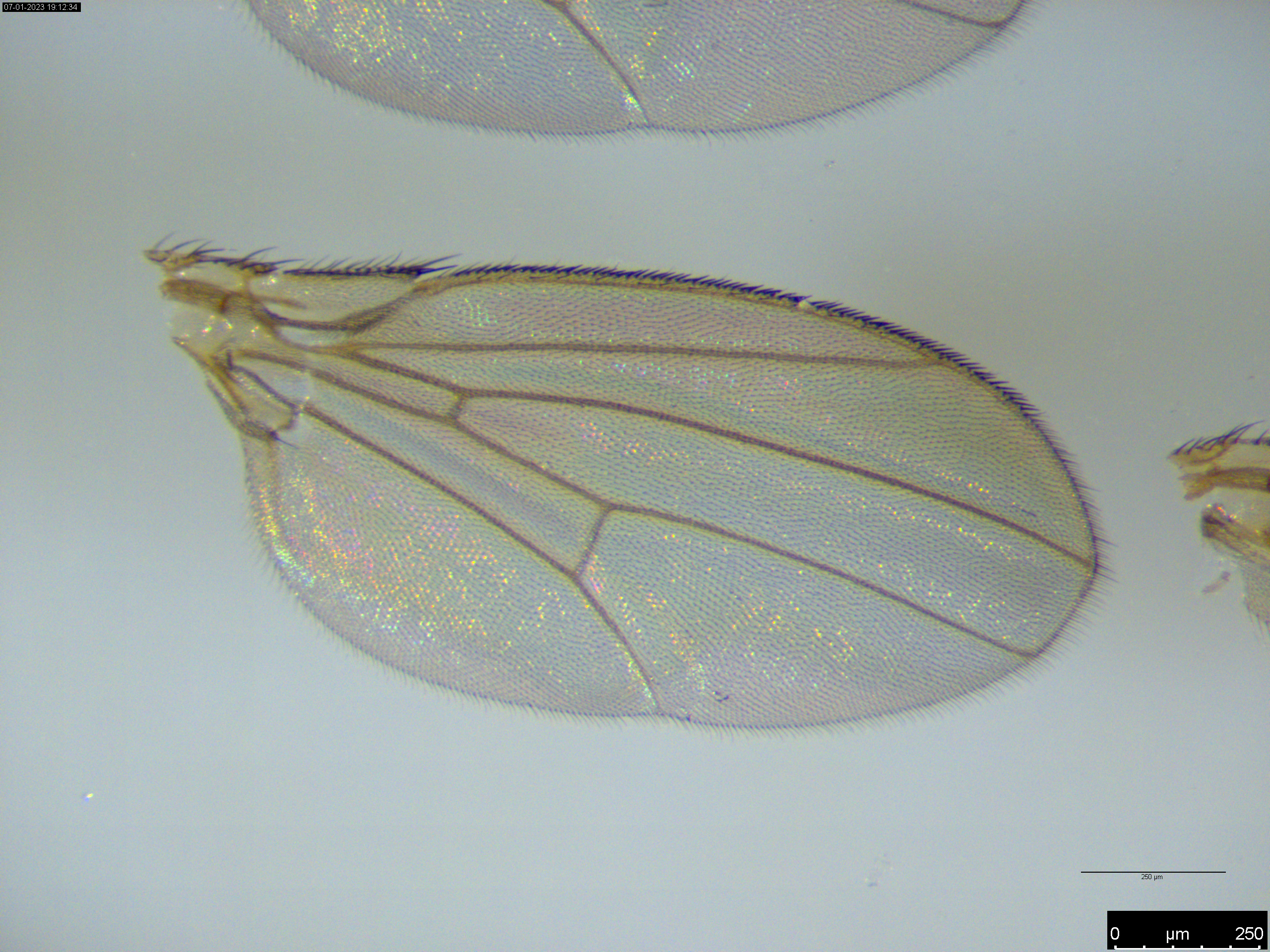

Supplement: Supplementary file 6 — Source data Fig. 4 [file 44319_2025_574_MOESM6_ESM.zip › Fig. 4/c-f/Control_wing_Ct.HSD.tif]

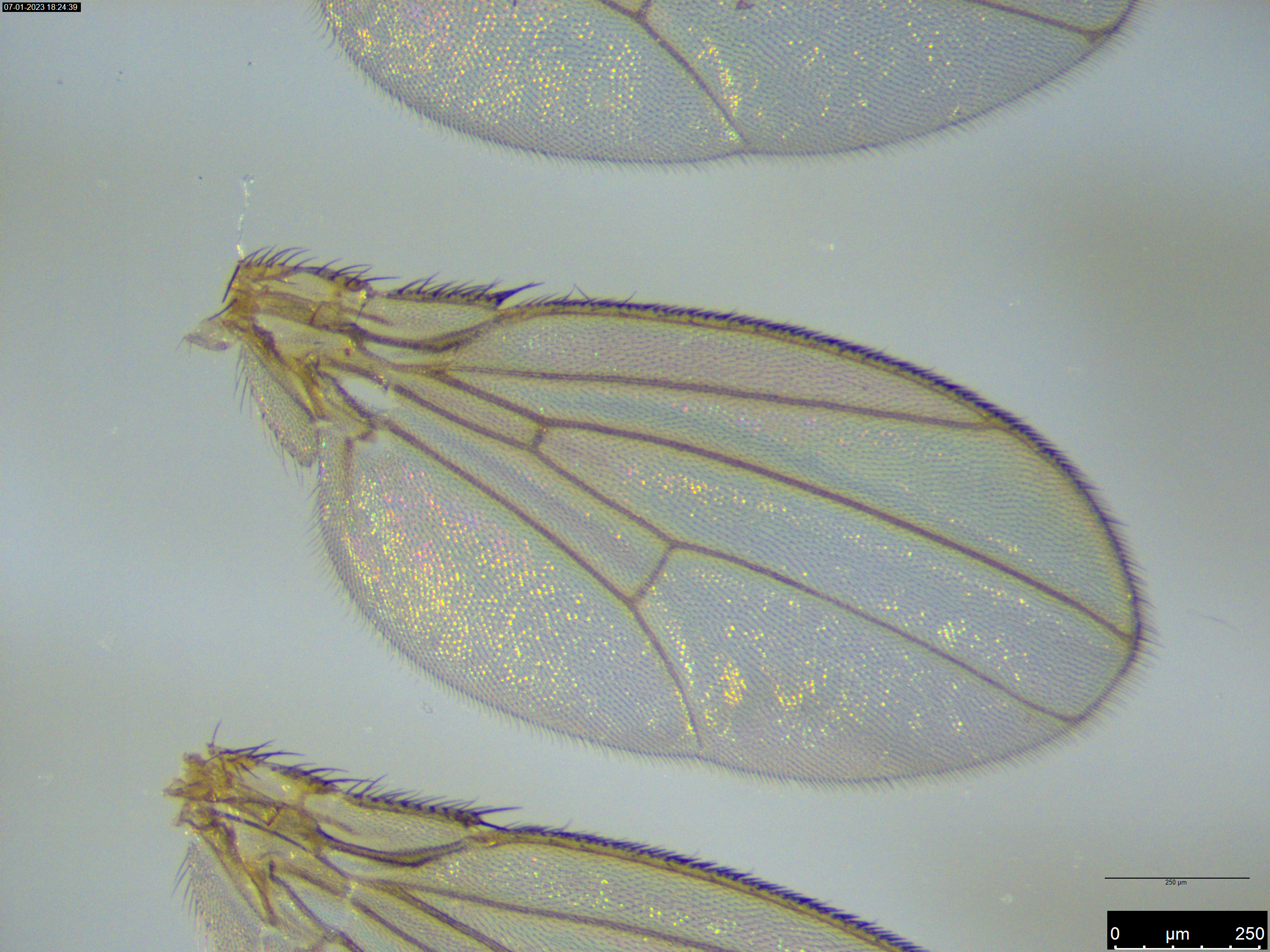

Supplement: Supplementary file 6 — Source data Fig. 4 [file 44319_2025_574_MOESM6_ESM.zip › Fig. 4/c-f/PdhaRNAi_wing_HSD.tif]

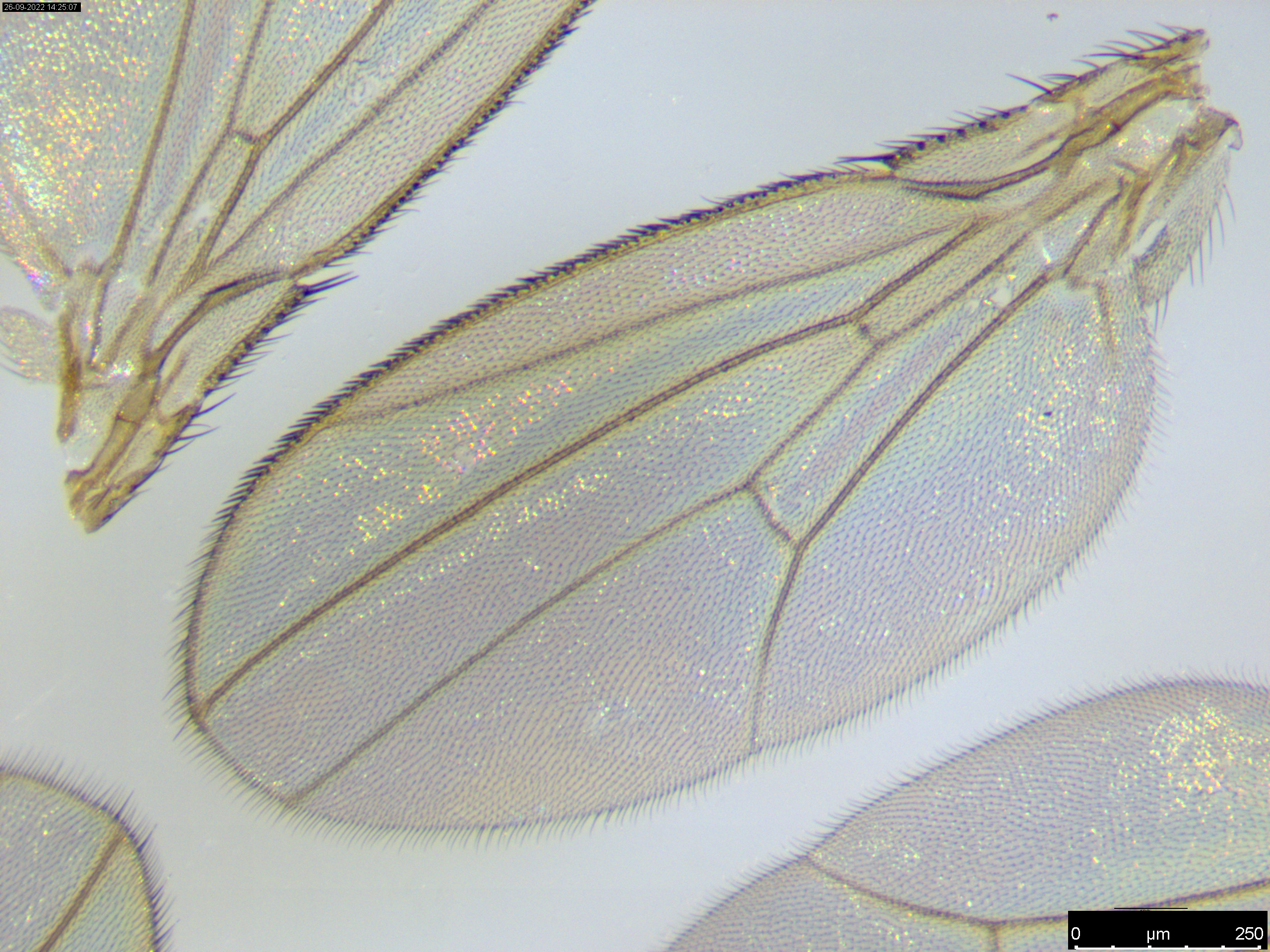

Supplement: Supplementary file 7 — Source data Fig. 5 [file 44319_2025_574_MOESM7_ESM.zip › Fig. 5/Fig. 5 k-o/Gpat4RNAi_wing_HSD_male.tif]

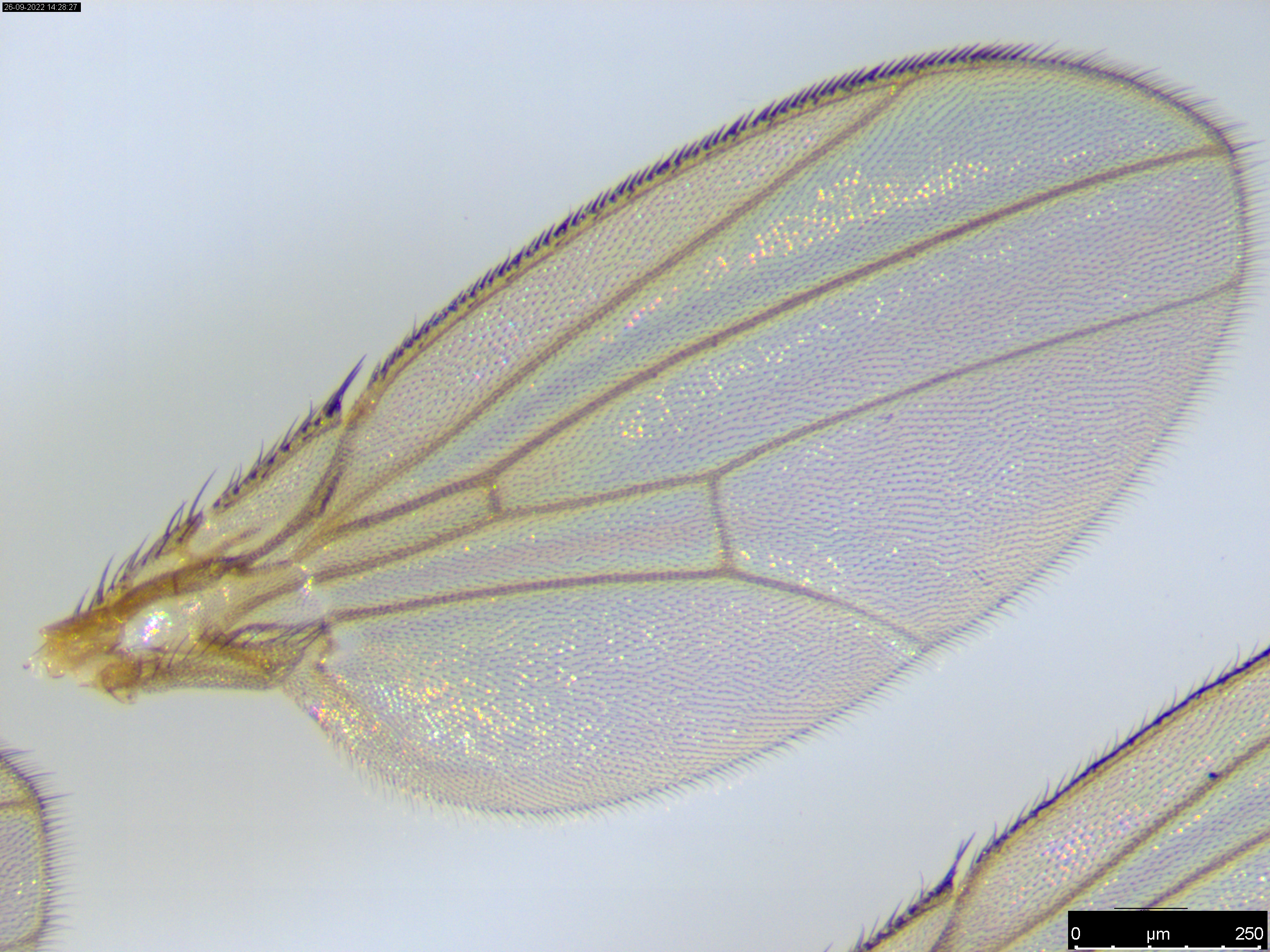

Supplement: Supplementary file 7 — Source data Fig. 5 [file 44319_2025_574_MOESM7_ESM.zip › Fig. 5/Fig. 5 k-o/Agpat3RNAi_wing_HSD_male.tif]

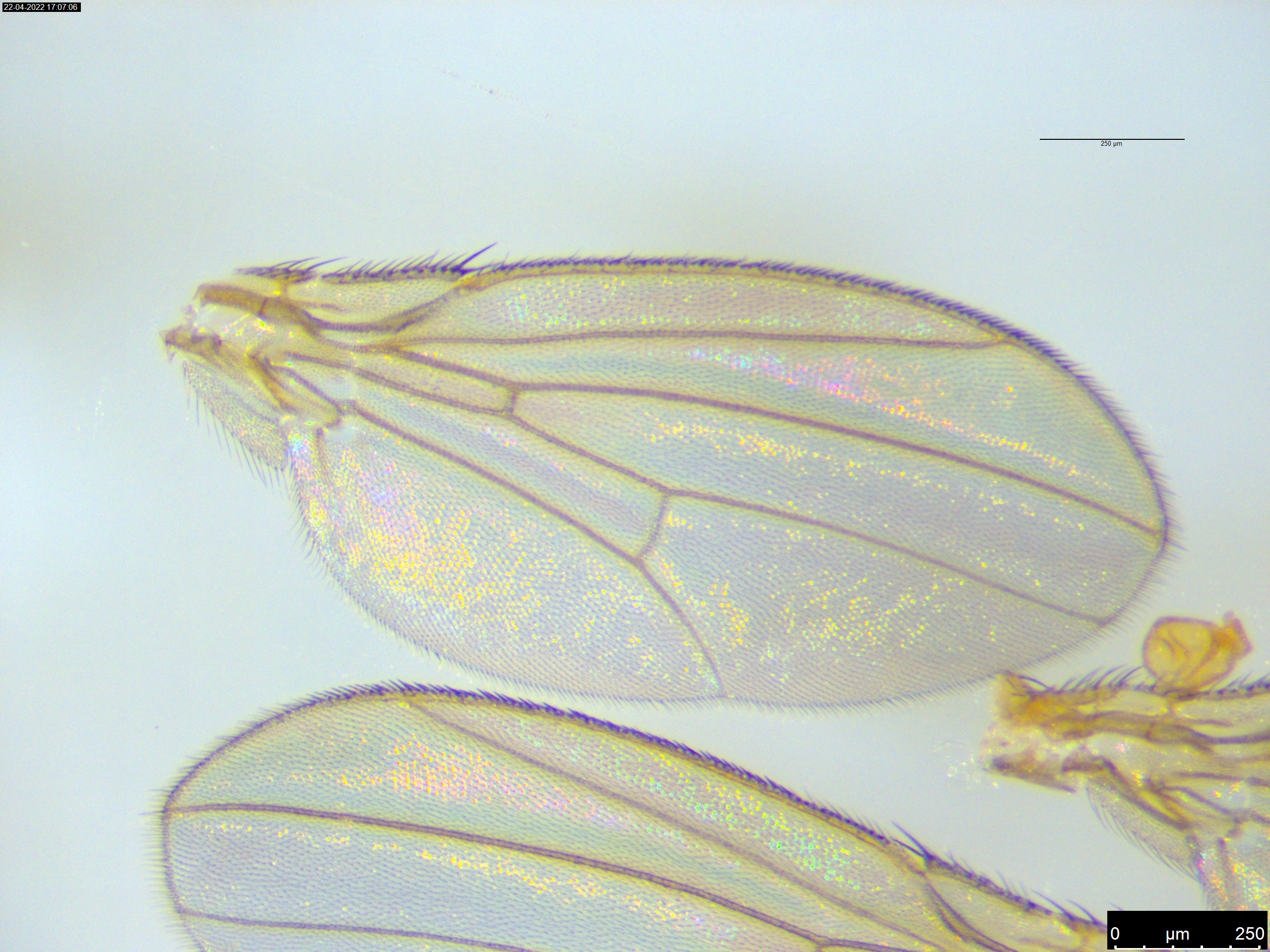

Supplement: Supplementary file 7 — Source data Fig. 5 [file 44319_2025_574_MOESM7_ESM.zip › Fig. 5/Fig. 5 k-o/Control_wing_HSD_male.tif]

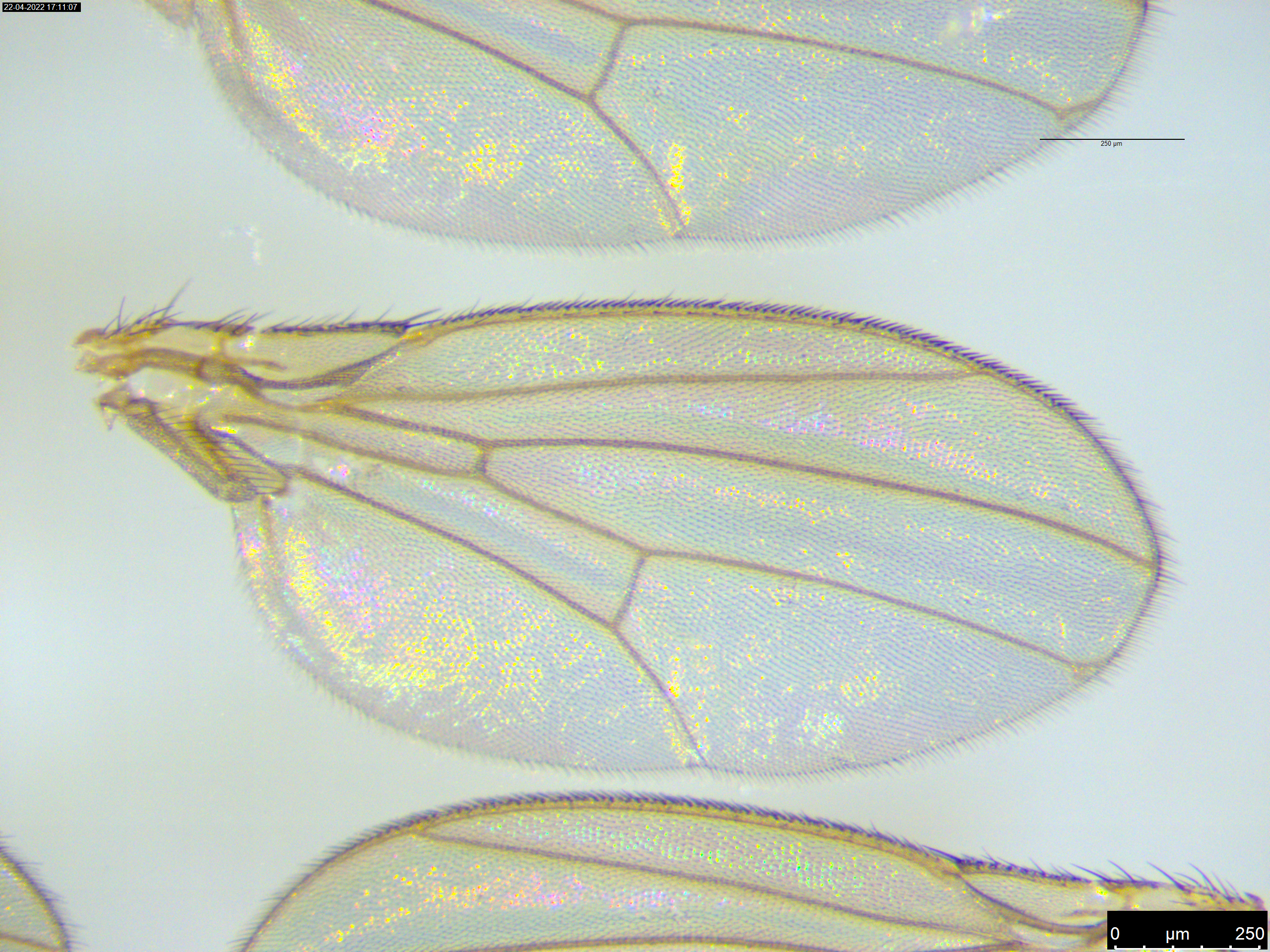

Supplement: Supplementary file 7 — Source data Fig. 5 [file 44319_2025_574_MOESM7_ESM.zip › Fig. 5/Fig. 5 k-o/UASACC_wing_HSD_male.tif]

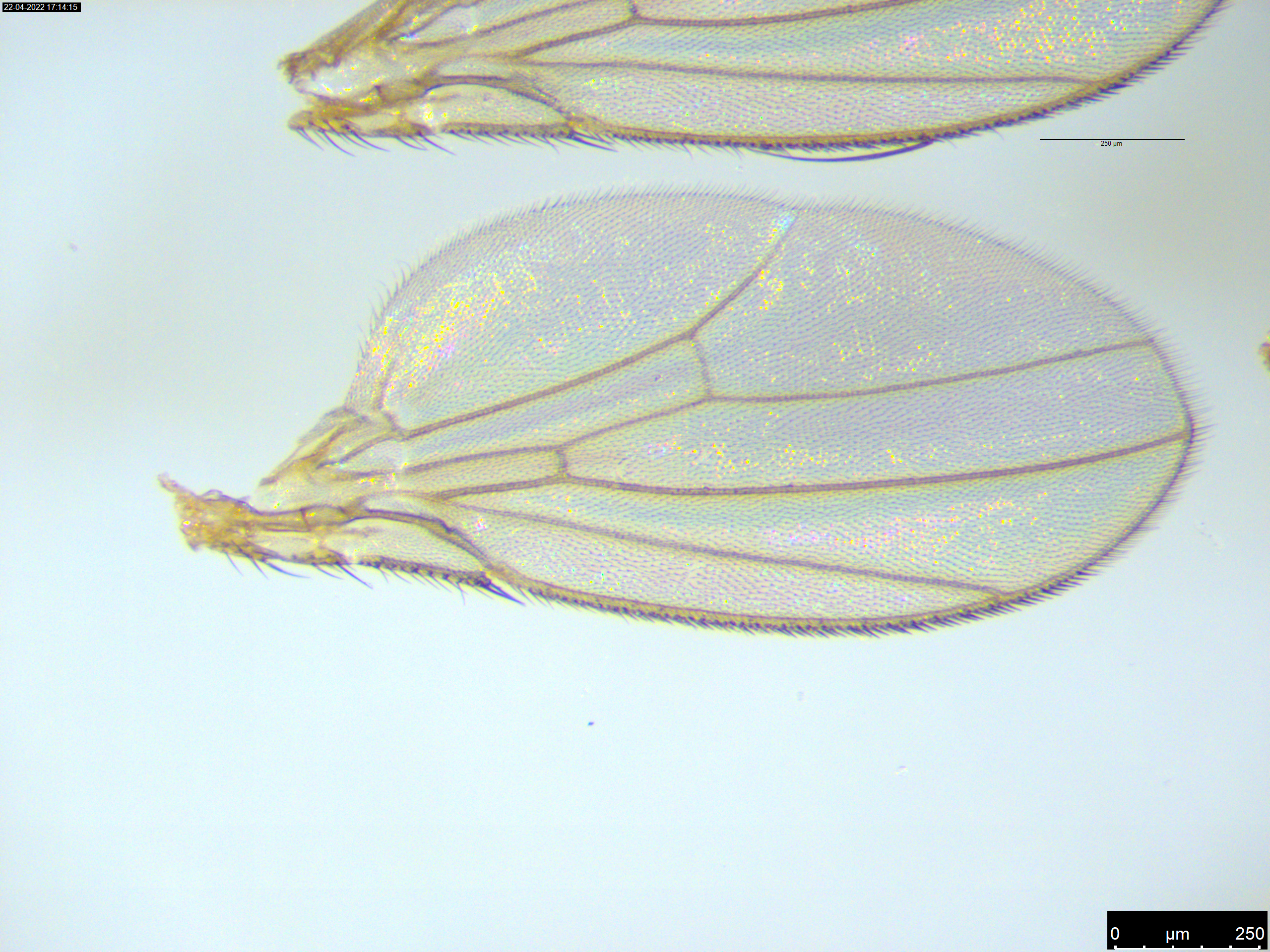

Supplement: Supplementary file 7 — Source data Fig. 5 [file 44319_2025_574_MOESM7_ESM.zip › Fig. 5/Fig. 5 k-o/ACCRNAi_wing_HSD_male.tif]

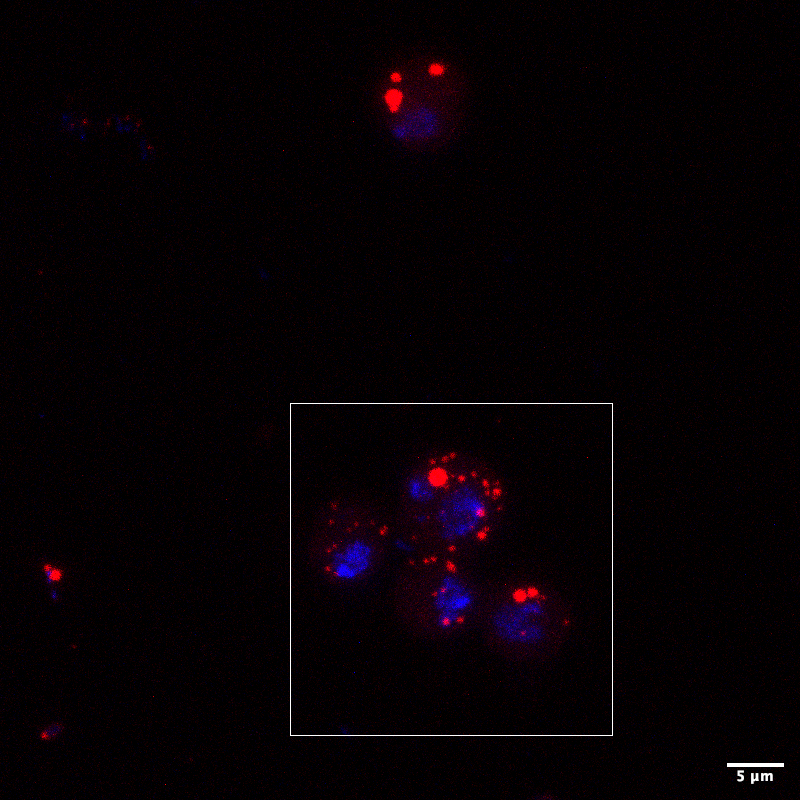

Supplement: Supplementary file 7 — Source data Fig. 5 [file 44319_2025_574_MOESM7_ESM.zip › Fig. 5/Fig. 5 f-j/Agpat3RNAi_nile red.tif]

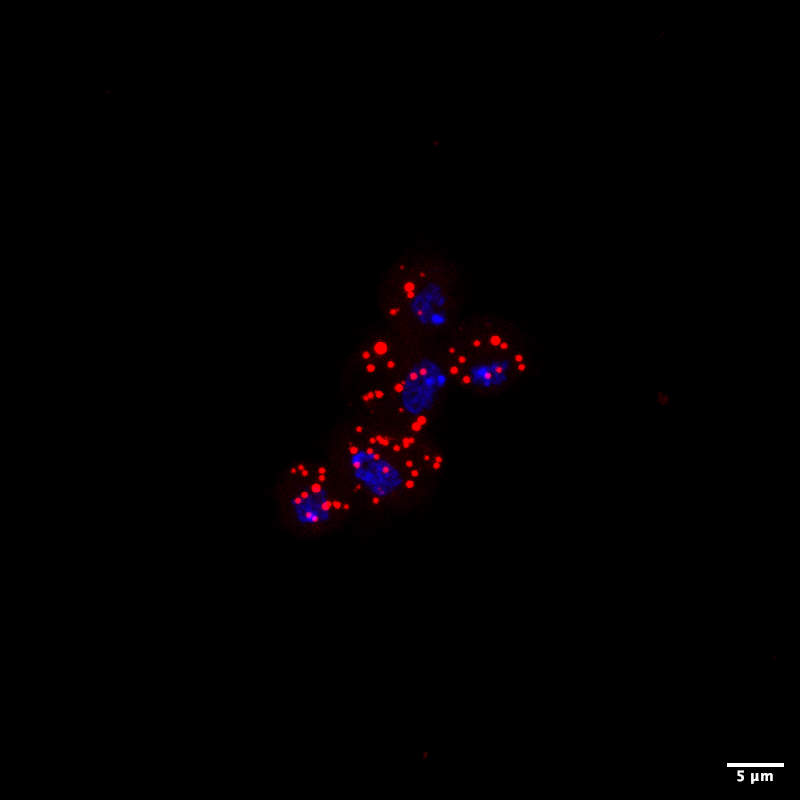

Supplement: Supplementary file 7 — Source data Fig. 5 [file 44319_2025_574_MOESM7_ESM.zip › Fig. 5/Fig. 5 f-j/Control_nile red.tif]

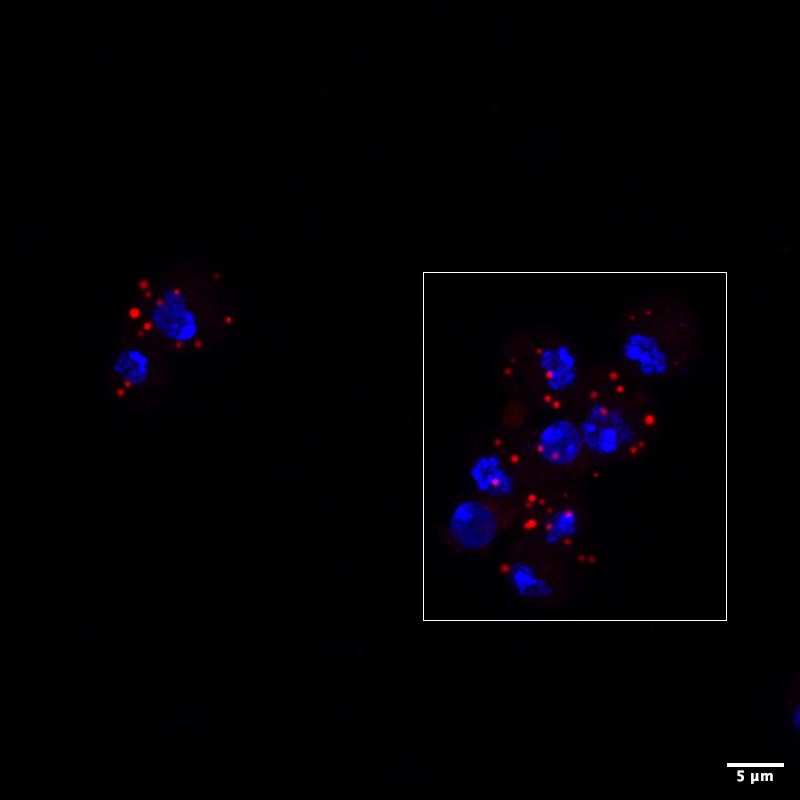

Supplement: Supplementary file 7 — Source data Fig. 5 [file 44319_2025_574_MOESM7_ESM.zip › Fig. 5/Fig. 5 f-j/Gpat4RNAi_nile red.tif]

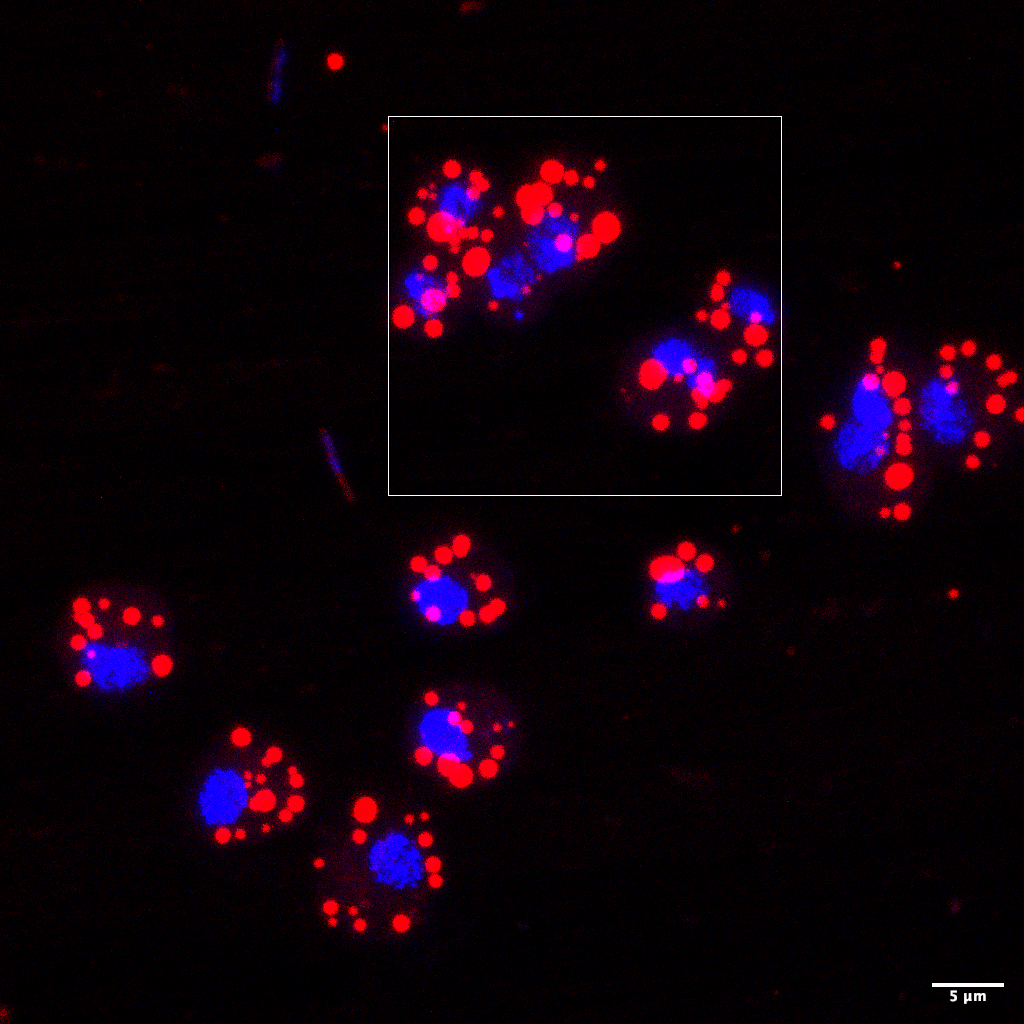

Supplement: Supplementary file 7 — Source data Fig. 5 [file 44319_2025_574_MOESM7_ESM.zip › Fig. 5/Fig. 5 f-j/UAS-ACC_nile red.tif]

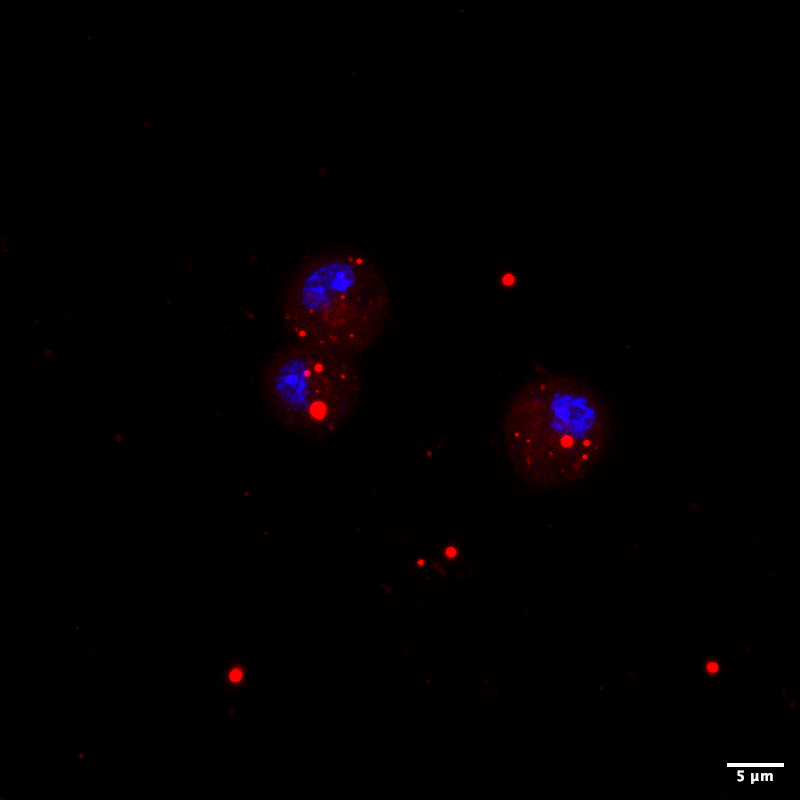

Supplement: Supplementary file 7 — Source data Fig. 5 [file 44319_2025_574_MOESM7_ESM.zip › Fig. 5/Fig. 5 f-j/ACCRNAi_nile red.tif]

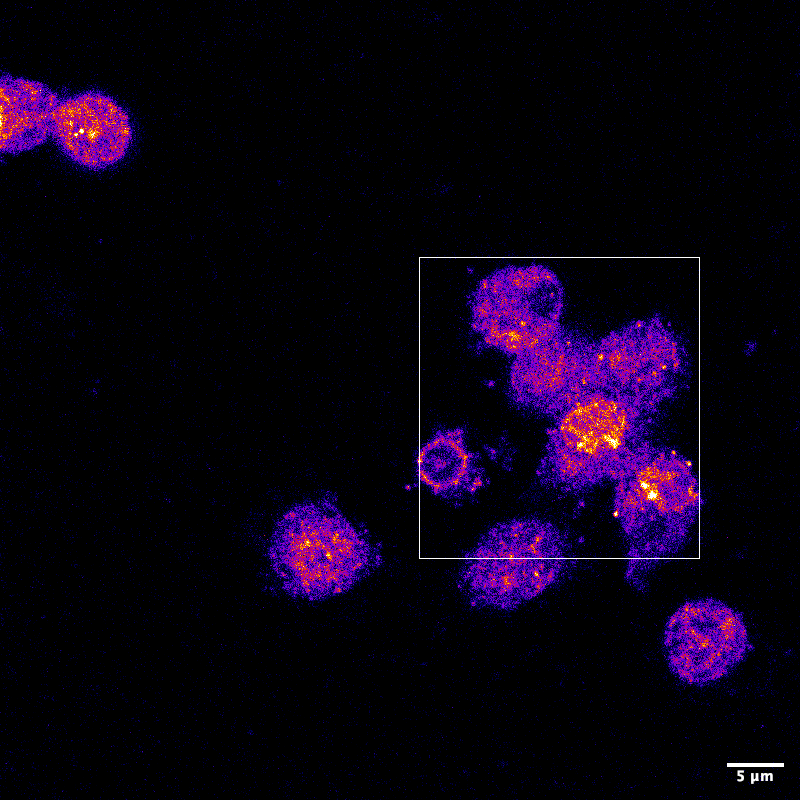

Supplement: Supplementary file 7 — Source data Fig. 5 [file 44319_2025_574_MOESM7_ESM.zip › Fig. 5/Fig. 5 b-d/CtHSD_ACC.tif]

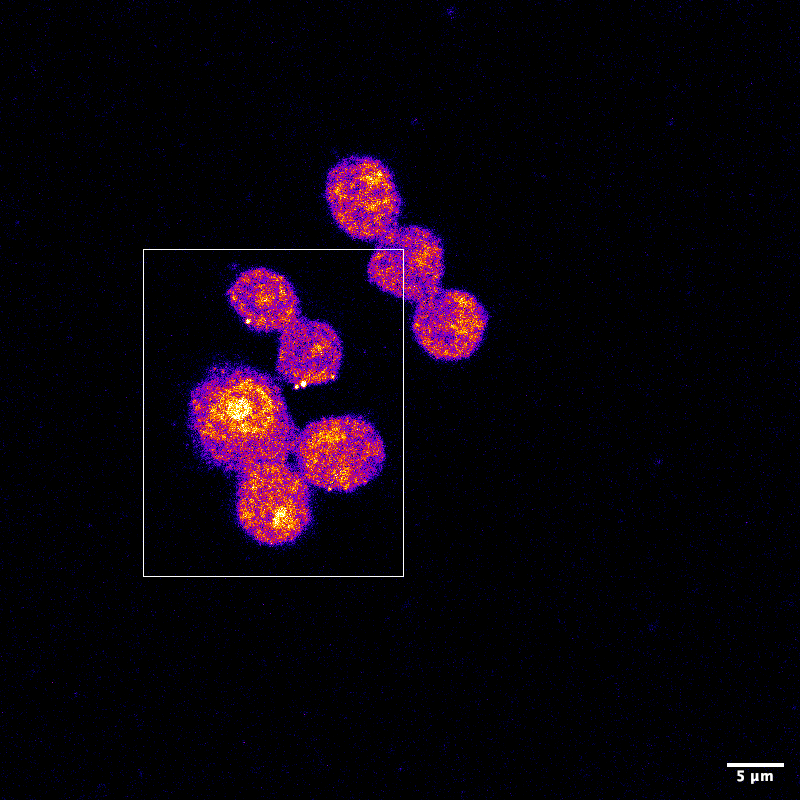

Supplement: Supplementary file 7 — Source data Fig. 5 [file 44319_2025_574_MOESM7_ESM.zip › Fig. 5/Fig. 5 b-d/RF_ACC.tif]

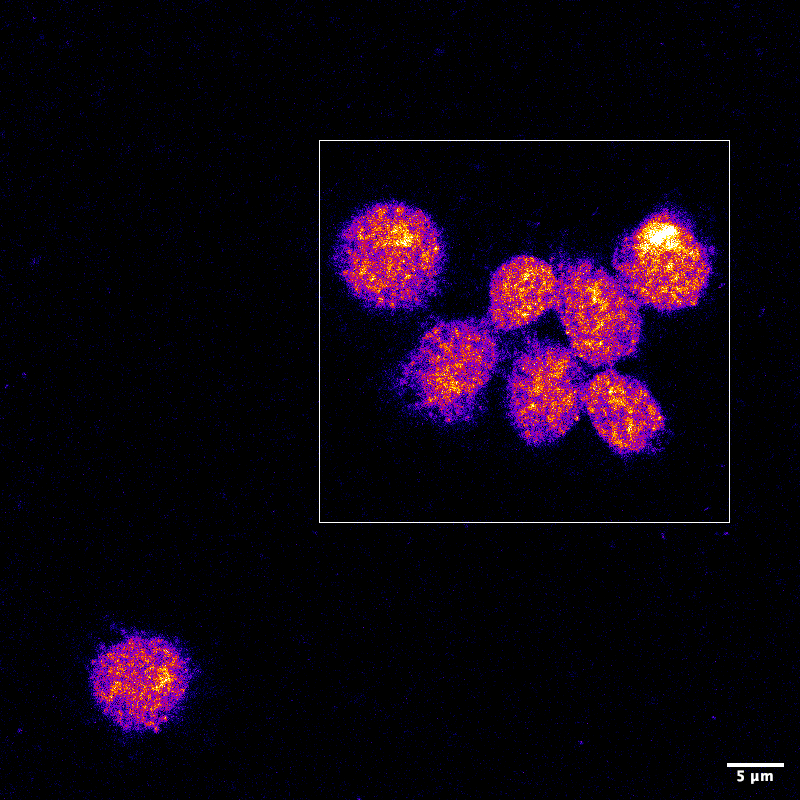

Supplement: Supplementary file 7 — Source data Fig. 5 [file 44319_2025_574_MOESM7_ESM.zip › Fig. 5/Fig. 5 b-d/4hr.HSD_ACC.tif]

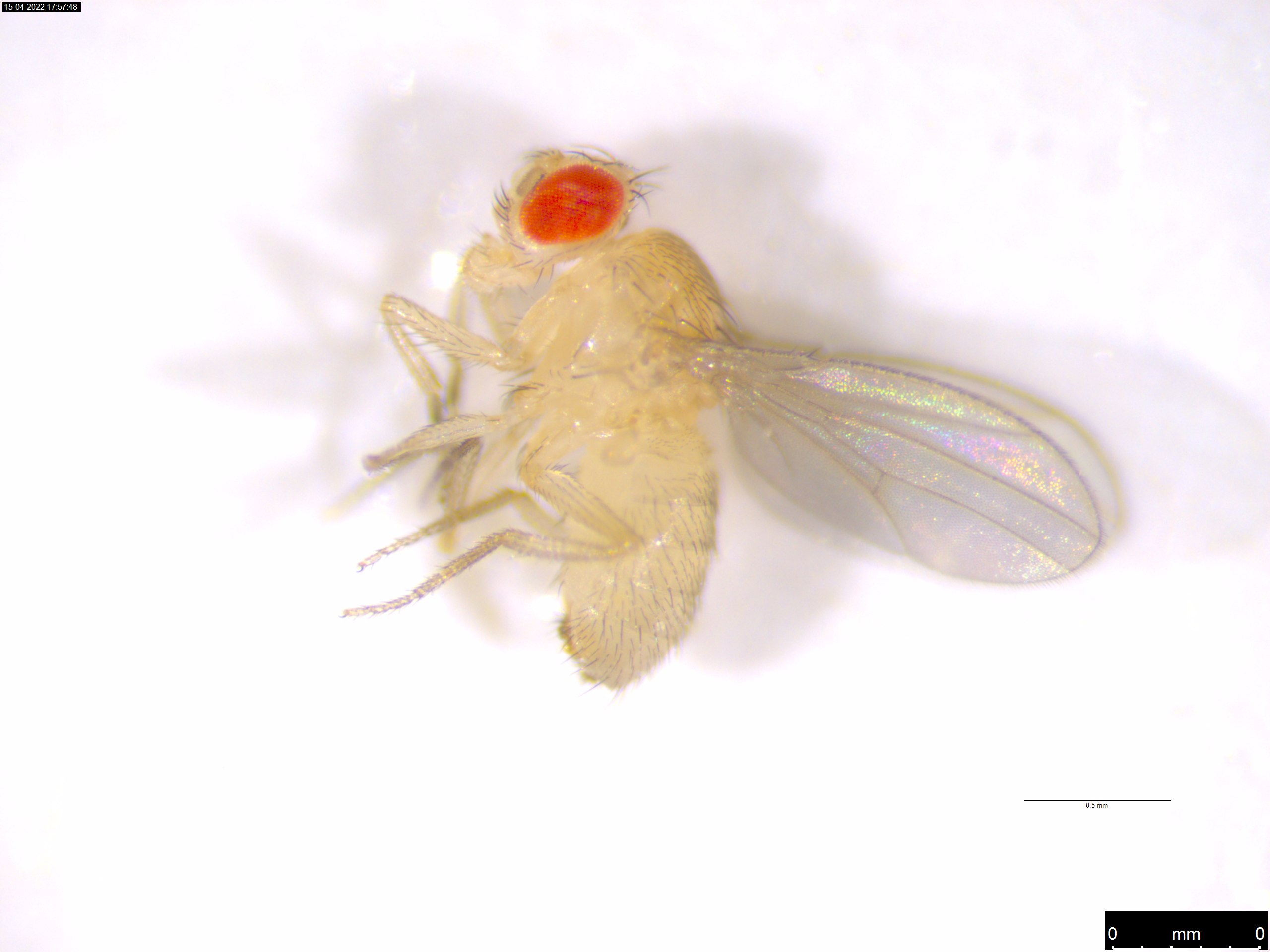

Supplement: Supplementary file 7 — Source data Fig. 5 [file 44319_2025_574_MOESM7_ESM.zip › Fig. 5/Fig. 5 k'-o'/ACCRNAi_fly.tif]

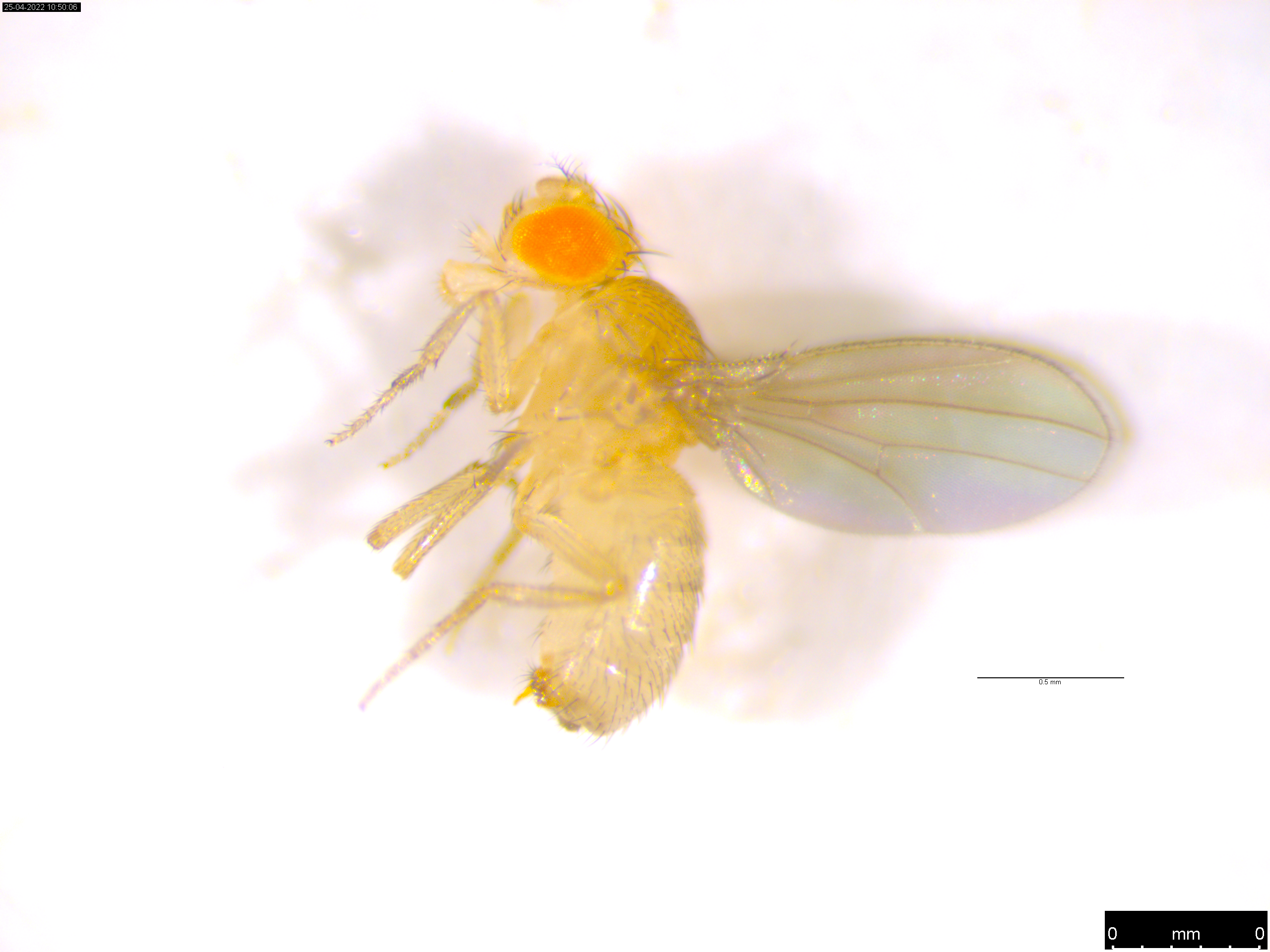

Supplement: Supplementary file 7 — Source data Fig. 5 [file 44319_2025_574_MOESM7_ESM.zip › Fig. 5/Fig. 5 k'-o'/Agpat3RNAi_fly.tif]

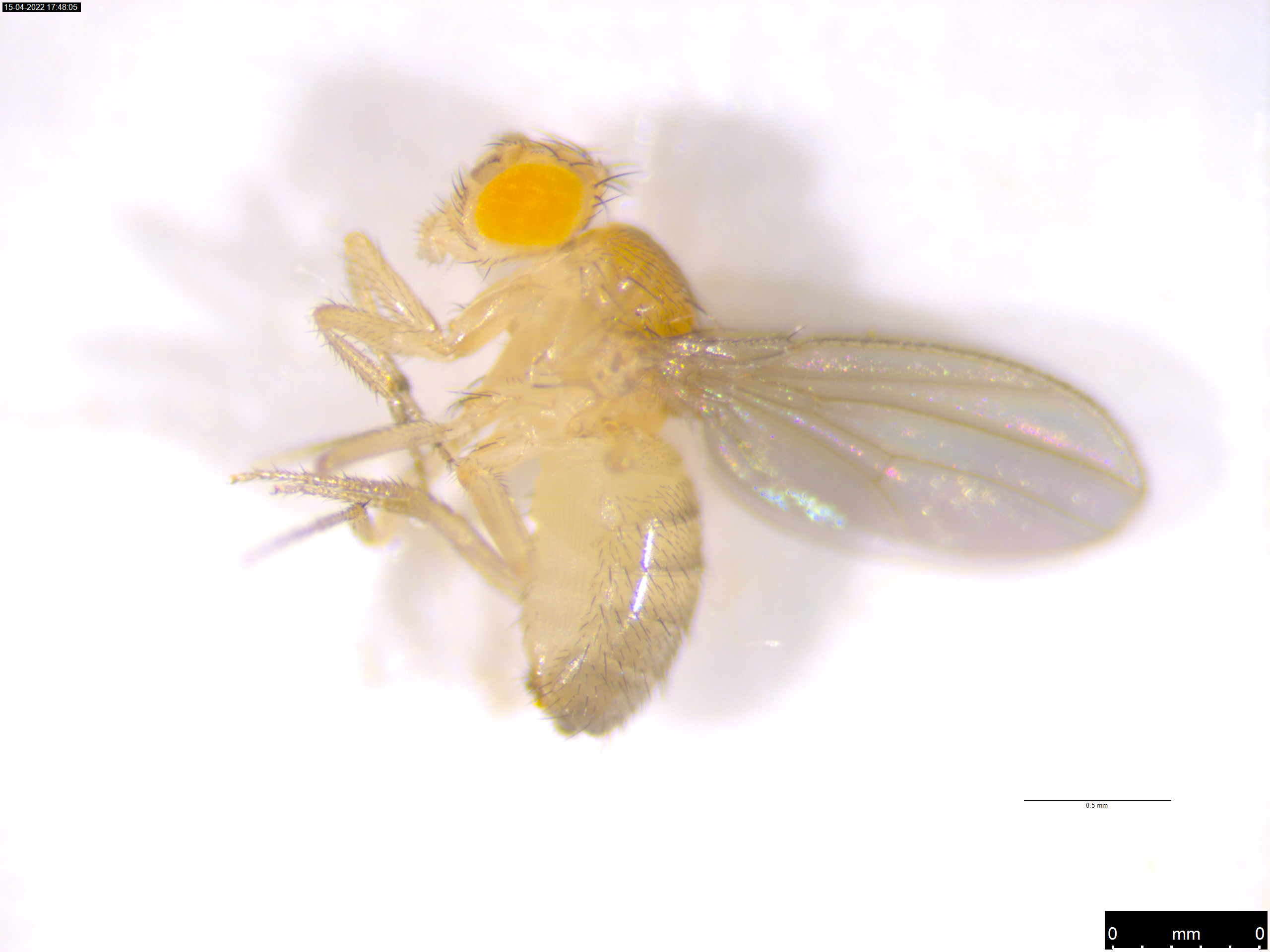

Supplement: Supplementary file 7 — Source data Fig. 5 [file 44319_2025_574_MOESM7_ESM.zip › Fig. 5/Fig. 5 k'-o'/Control_fly.tif]

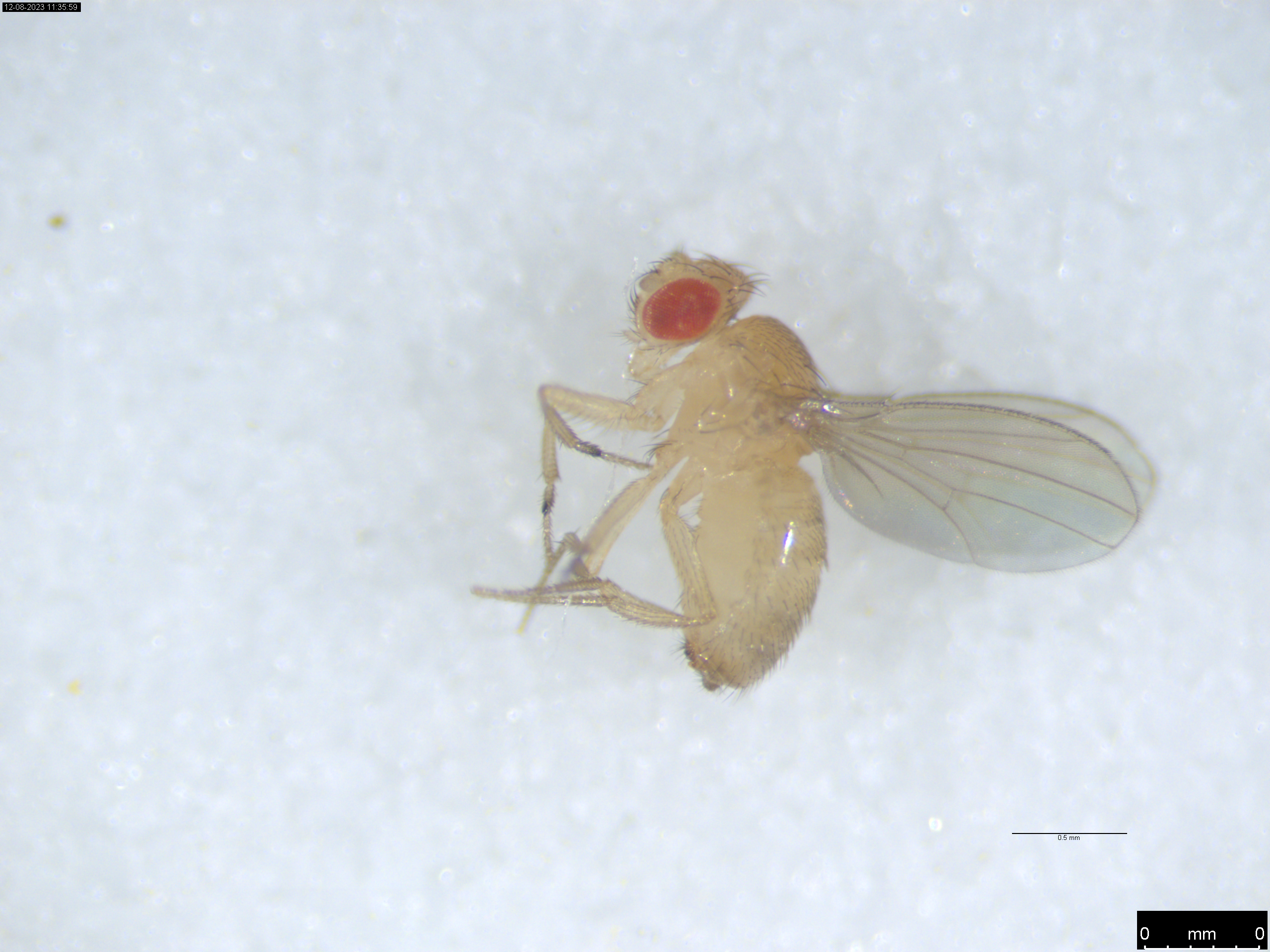

Supplement: Supplementary file 7 — Source data Fig. 5 [file 44319_2025_574_MOESM7_ESM.zip › Fig. 5/Fig. 5 k'-o'/Gpat4RNAi_fly.tif]

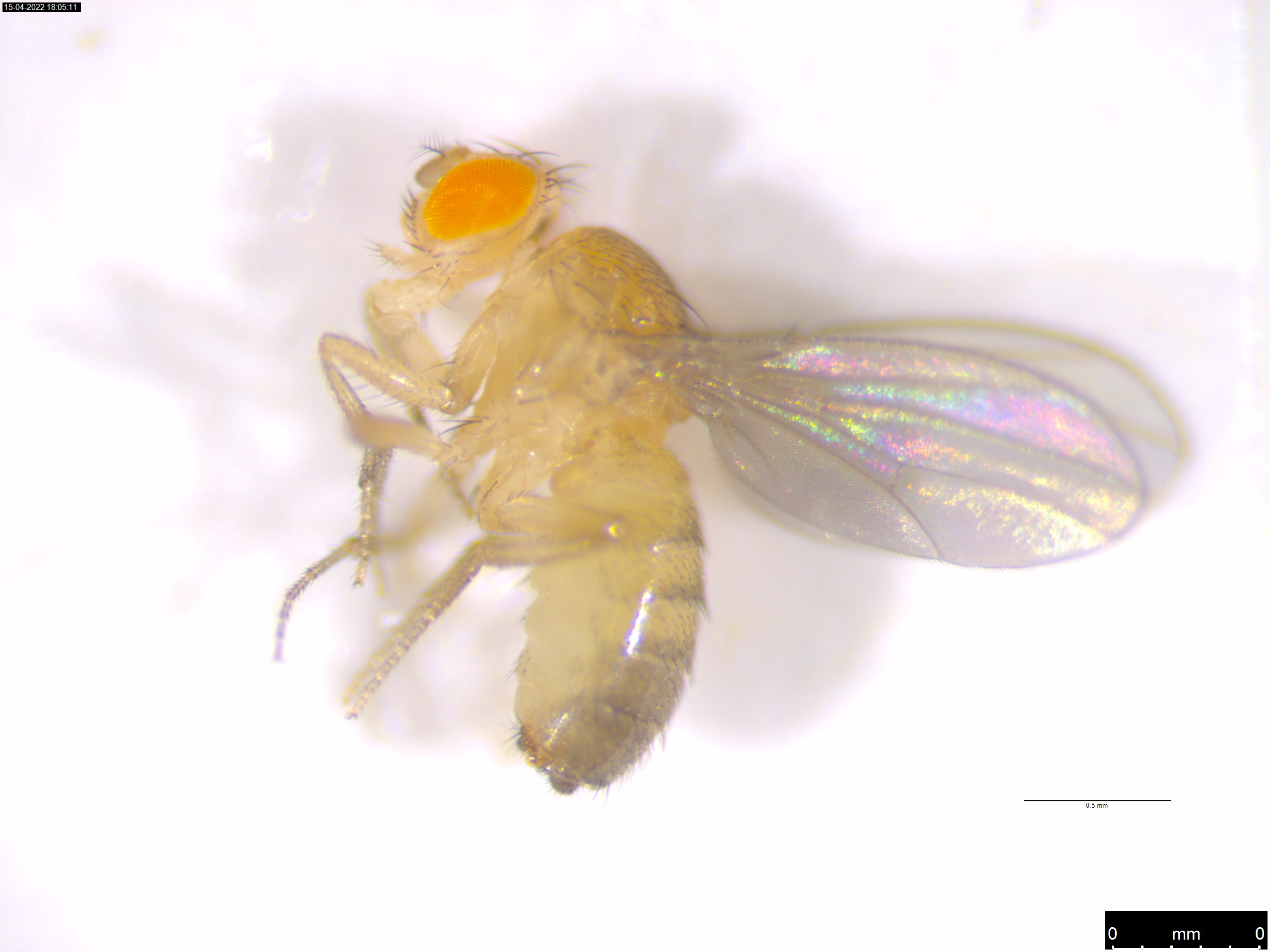

Supplement: Supplementary file 7 — Source data Fig. 5 [file 44319_2025_574_MOESM7_ESM.zip › Fig. 5/Fig. 5 k'-o'/UAS-ACC_fly.tif]

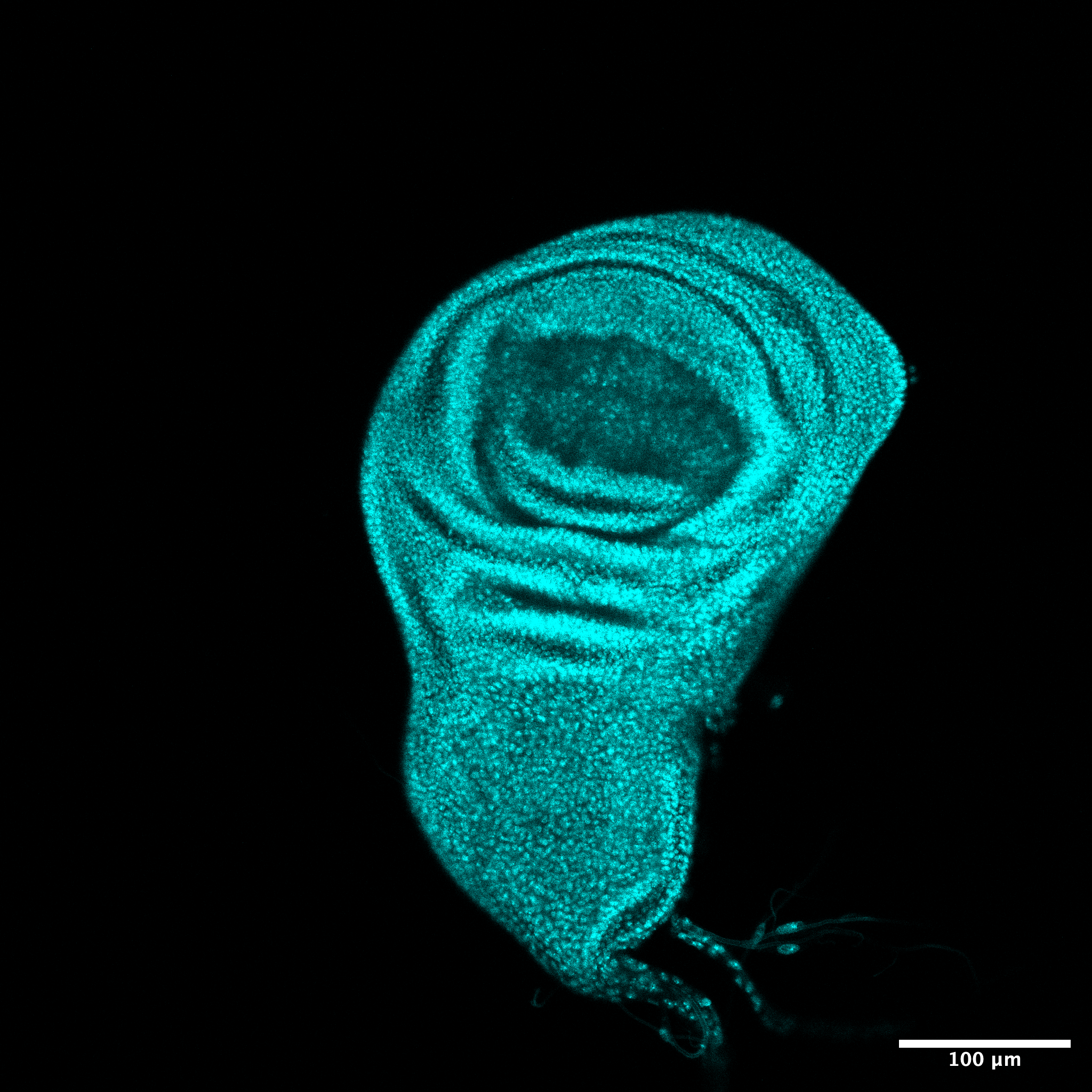

Supplement: Supplementary file 8 — Source data Fig. 6 [file 44319_2025_574_MOESM8_ESM.zip › Fig. 6/Fig. 6 h-n/Control_wingdisc.tif]

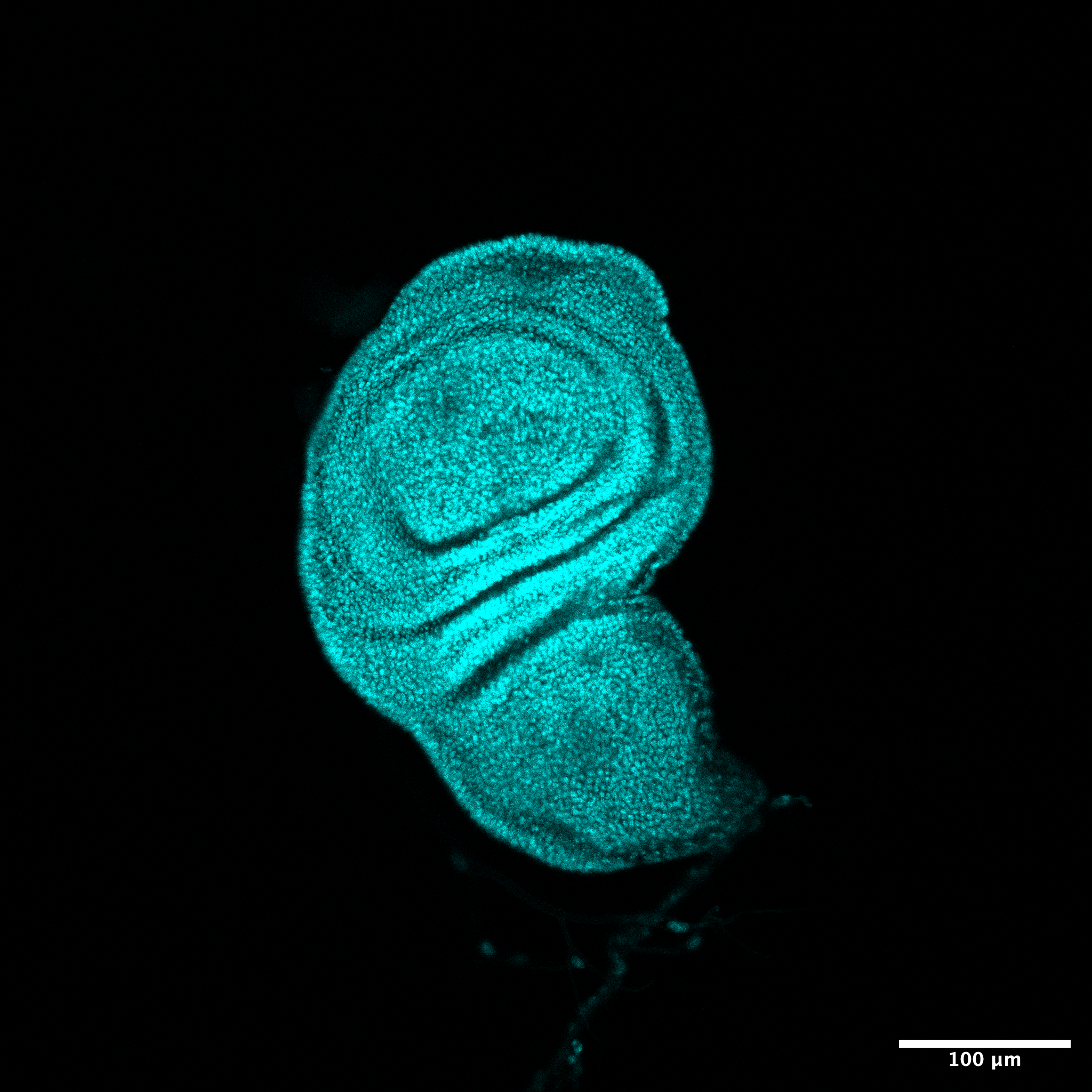

Supplement: Supplementary file 8 — Source data Fig. 6 [file 44319_2025_574_MOESM8_ESM.zip › Fig. 6/Fig. 6 h-n/Gpat4RNAi_wingdisc.tif]

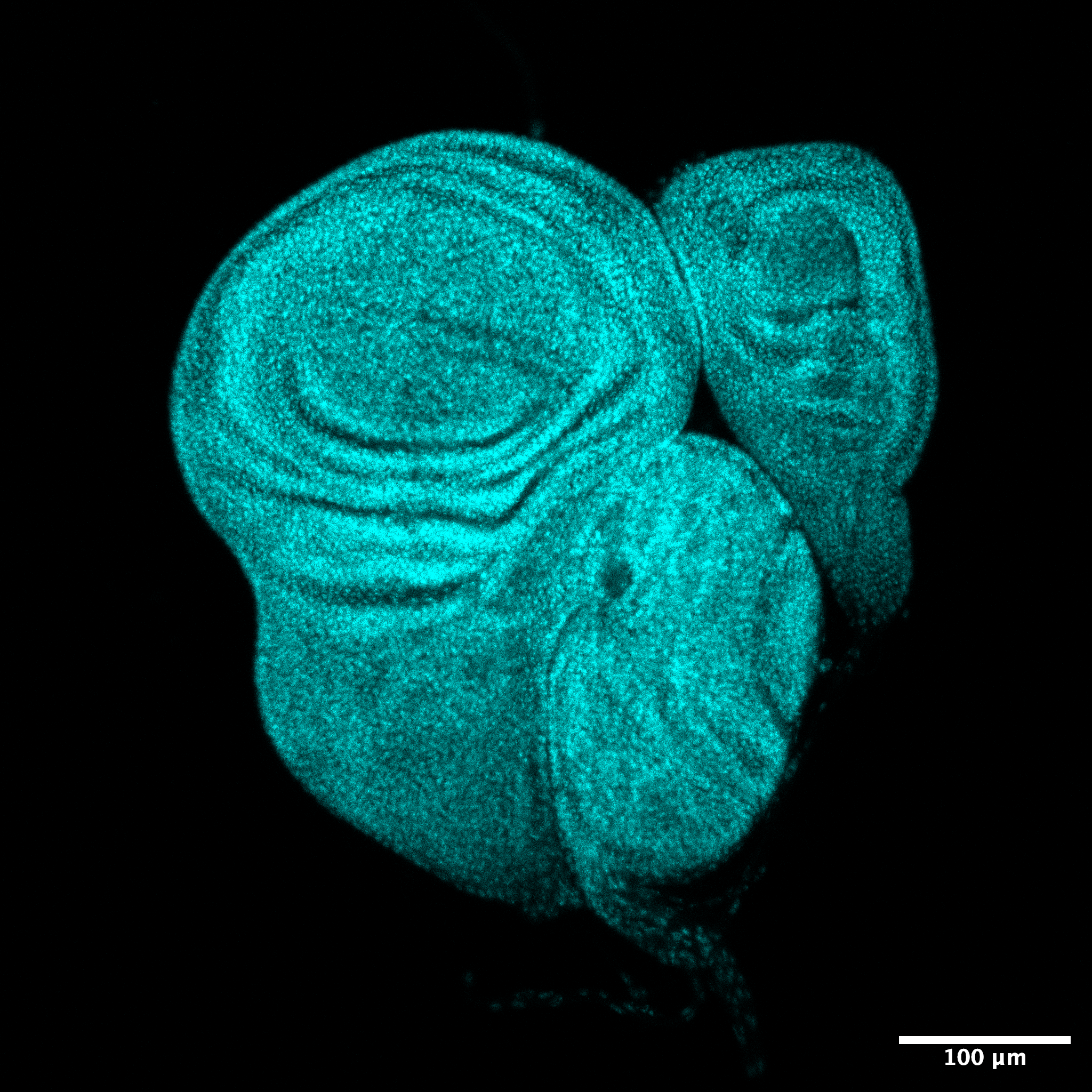

Supplement: Supplementary file 8 — Source data Fig. 6 [file 44319_2025_574_MOESM8_ESM.zip › Fig. 6/Fig. 6 h-n/BmmRNAi_wingdisc.tif]

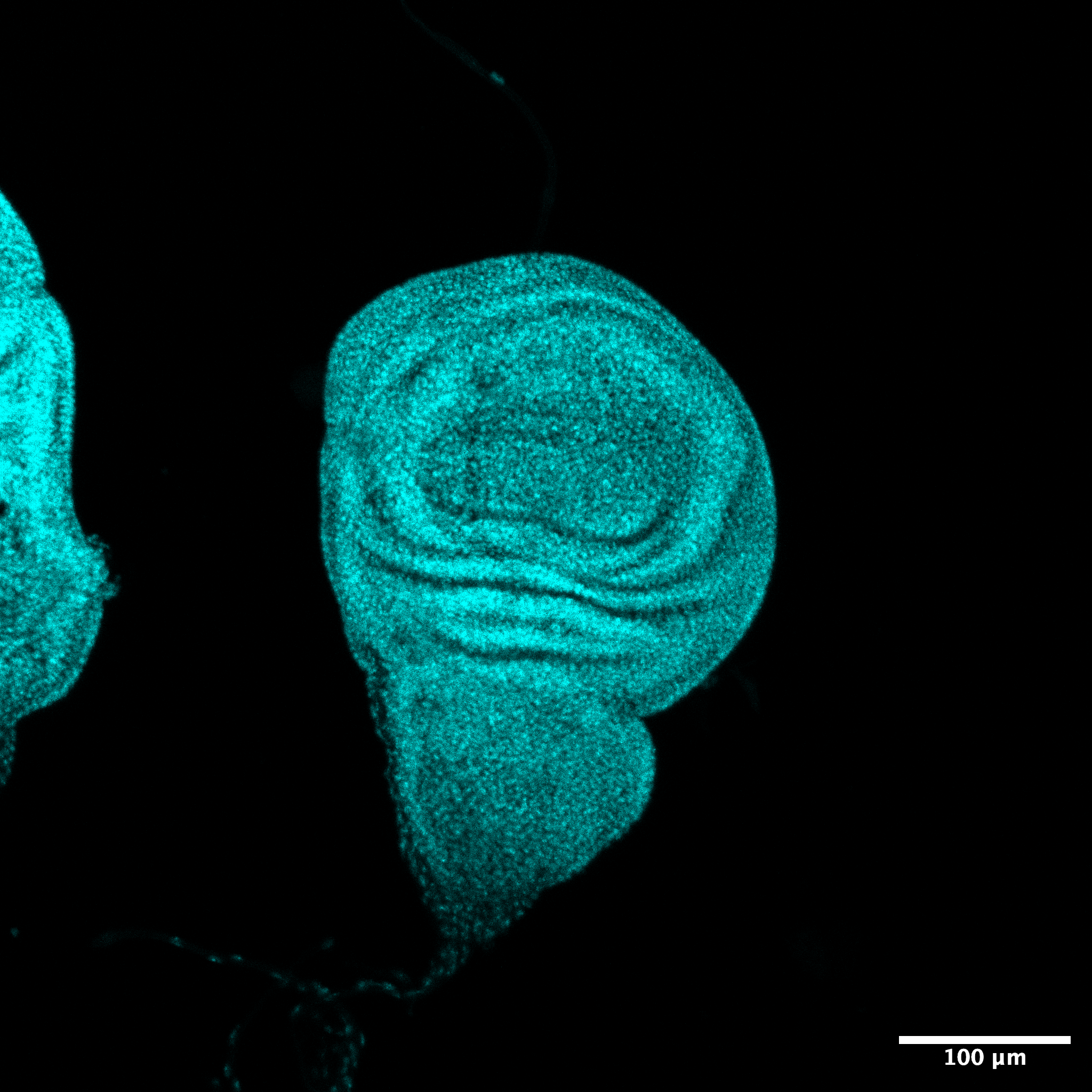

Supplement: Supplementary file 8 — Source data Fig. 6 [file 44319_2025_574_MOESM8_ESM.zip › Fig. 6/Fig. 6 h-n/crqRNAi_wingdisc.tif]

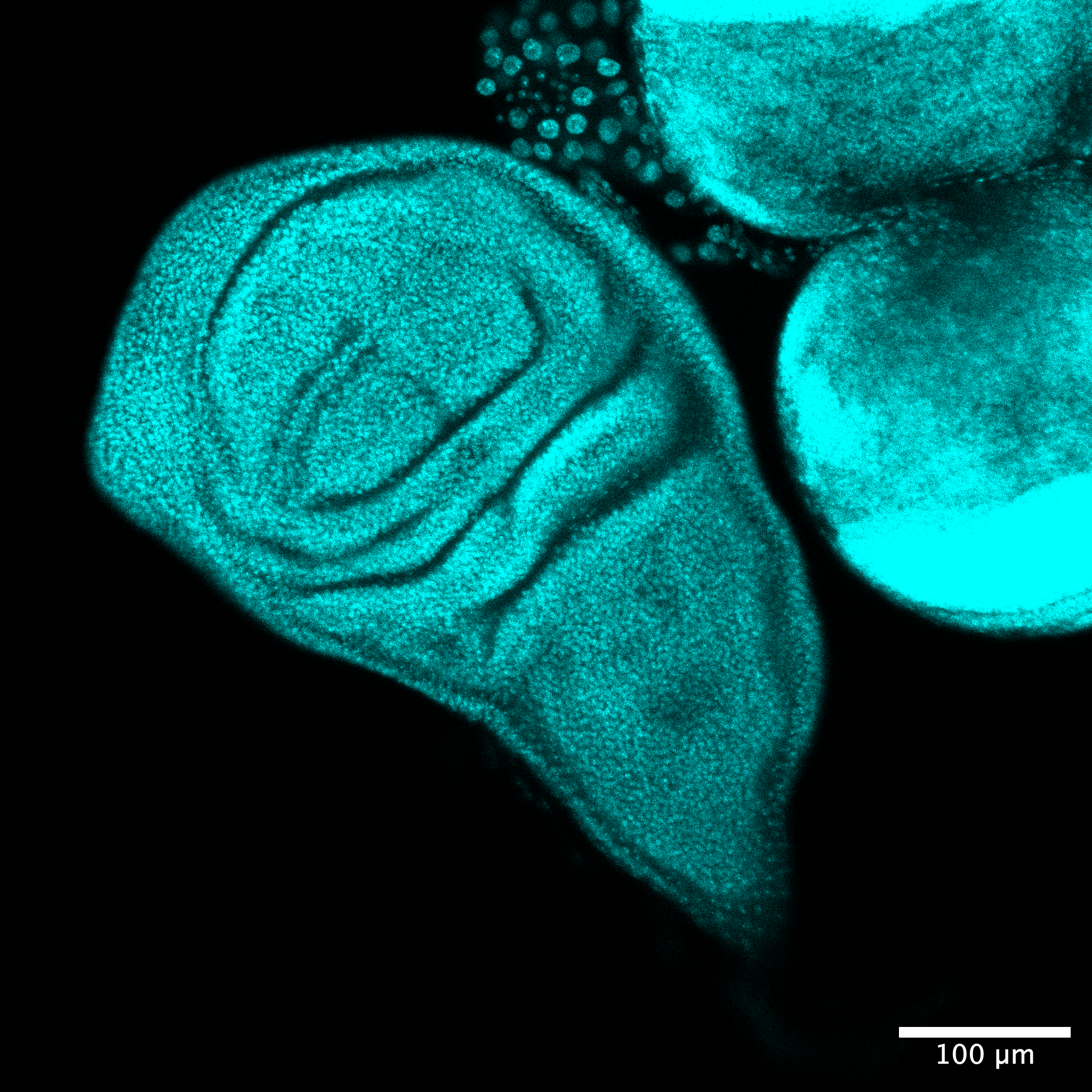

Supplement: Supplementary file 8 — Source data Fig. 6 [file 44319_2025_574_MOESM8_ESM.zip › Fig. 6/Fig. 6 h-n/UAS-ACC_wingdisc.tif]

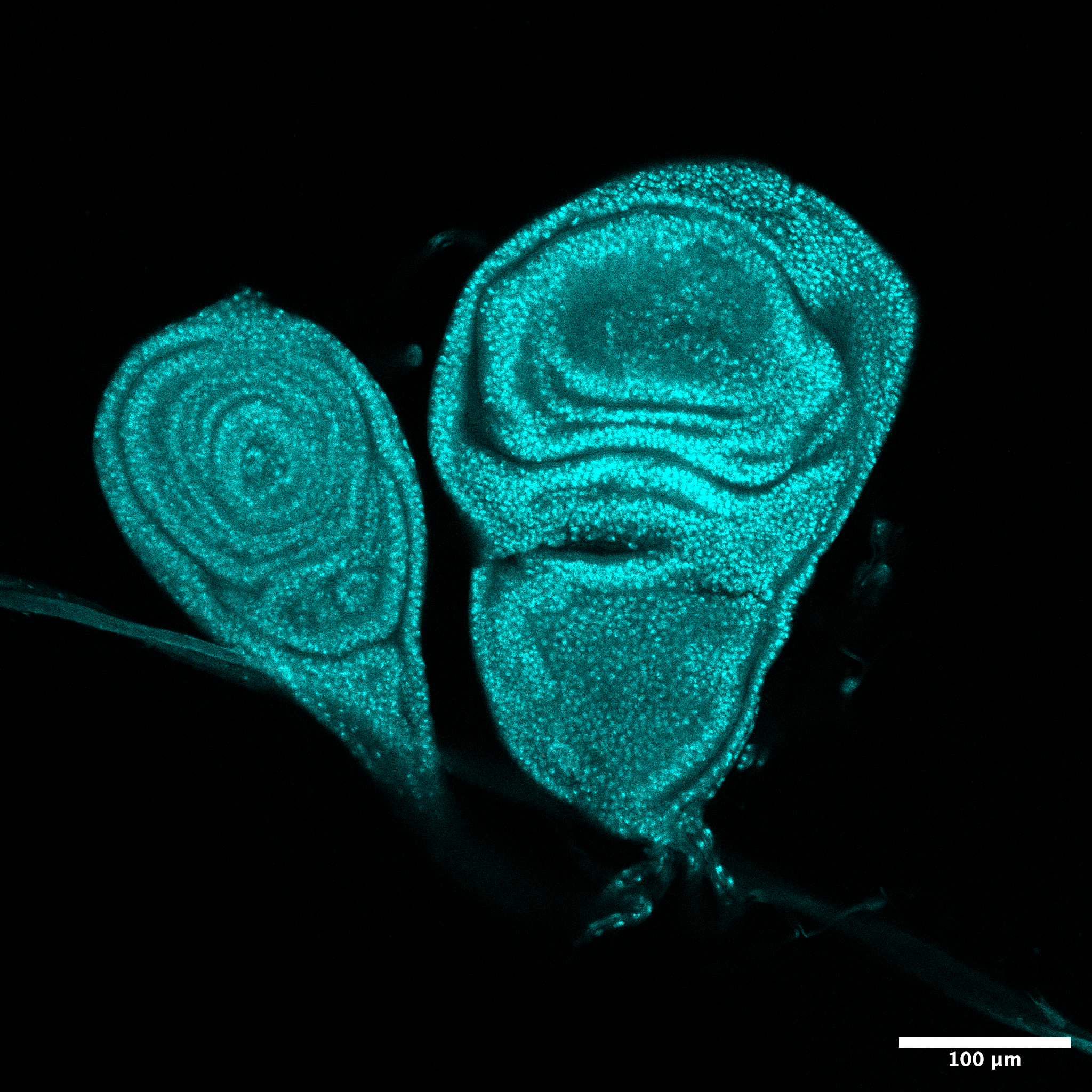

Supplement: Supplementary file 8 — Source data Fig. 6 [file 44319_2025_574_MOESM8_ESM.zip › Fig. 6/Fig. 6 h-n/ACCRNAi_wingdisc.tif]

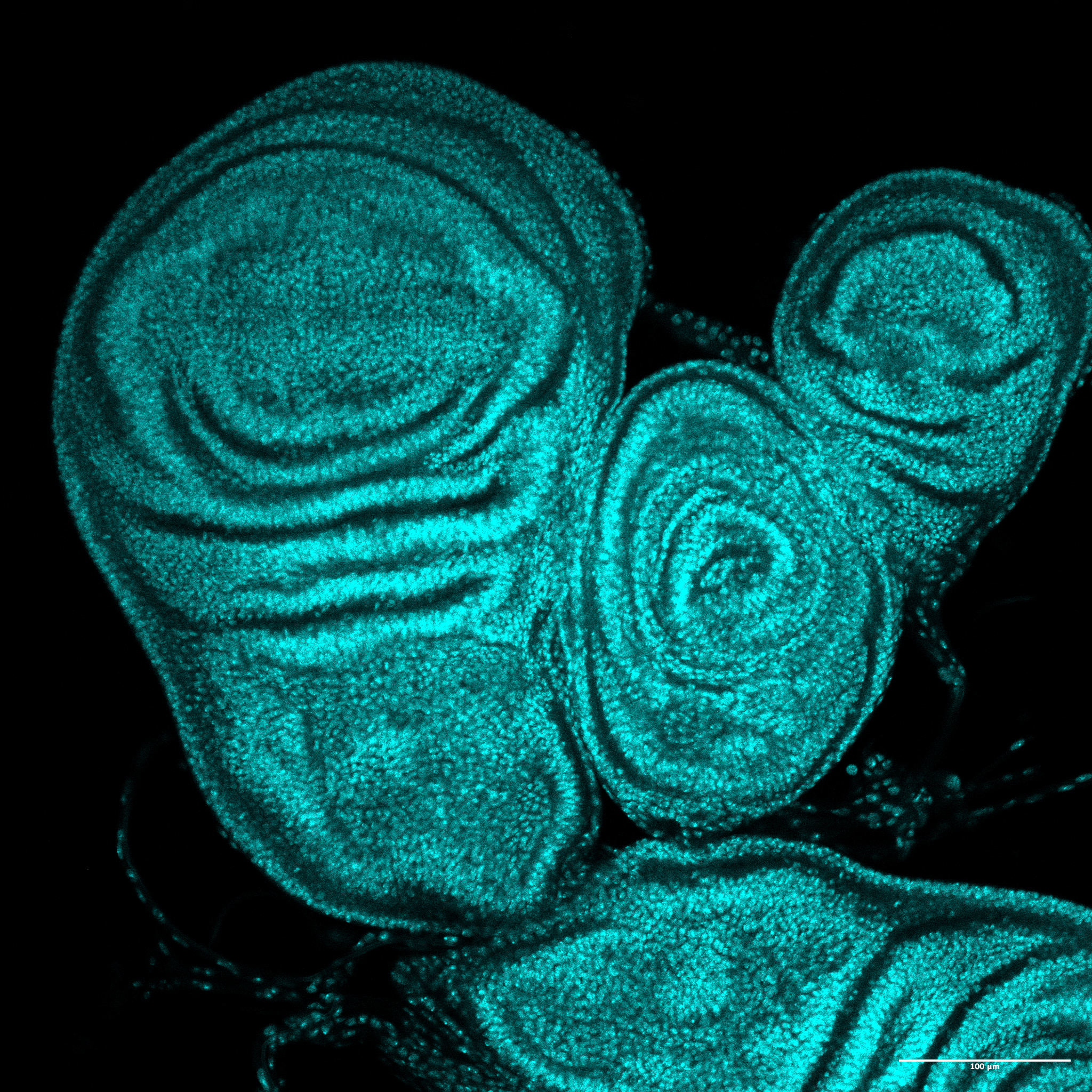

Supplement: Supplementary file 8 — Source data Fig. 6 [file 44319_2025_574_MOESM8_ESM.zip › Fig. 6/Fig. 6 h-n/LdhRNAi_wingdisc.tif]

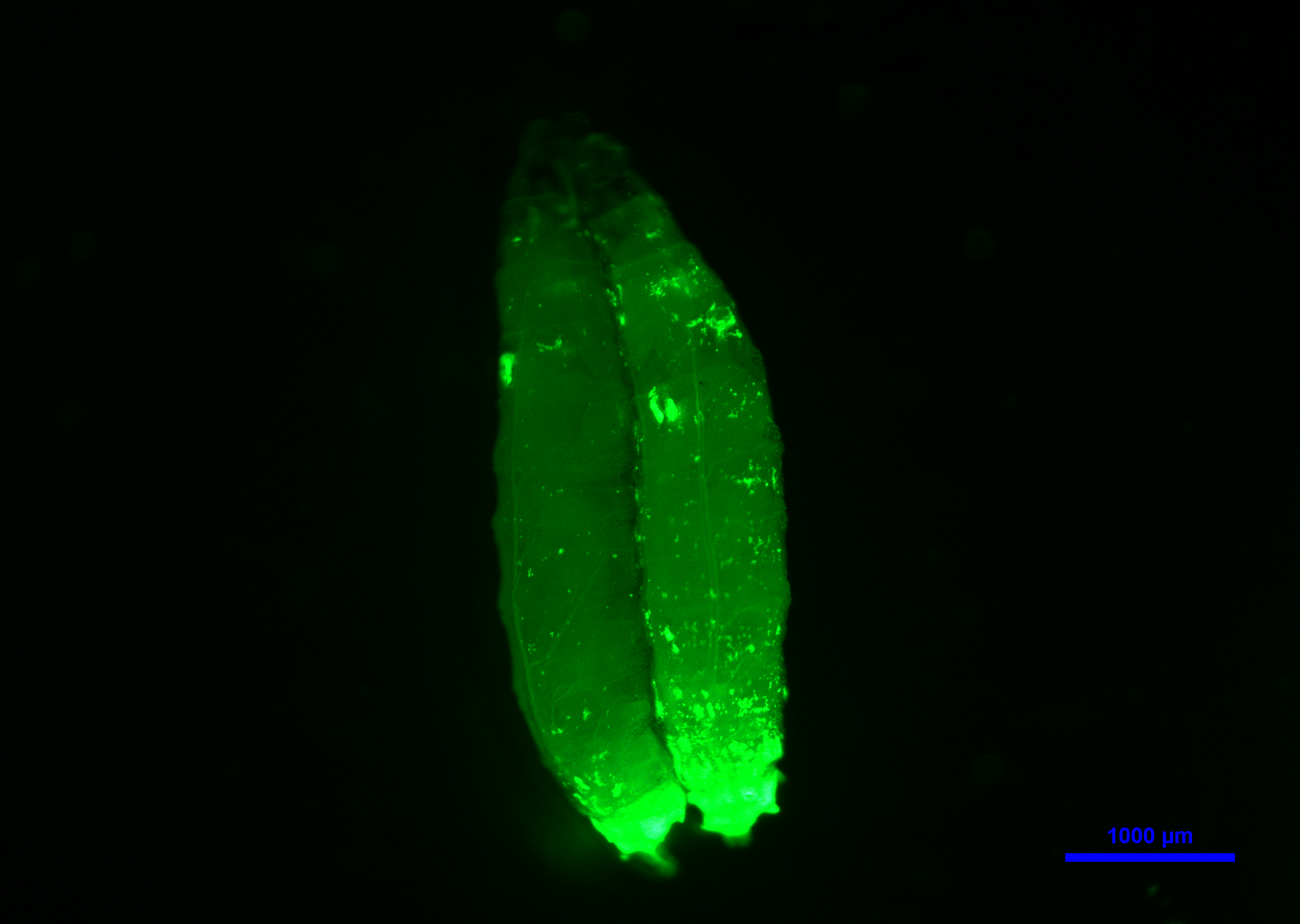

Supplement: Supplementary file 8 — Source data Fig. 6 [file 44319_2025_574_MOESM8_ESM.zip › Fig. 6/Fig. 6 a-g/crqRNAi_larvae_1.tif]

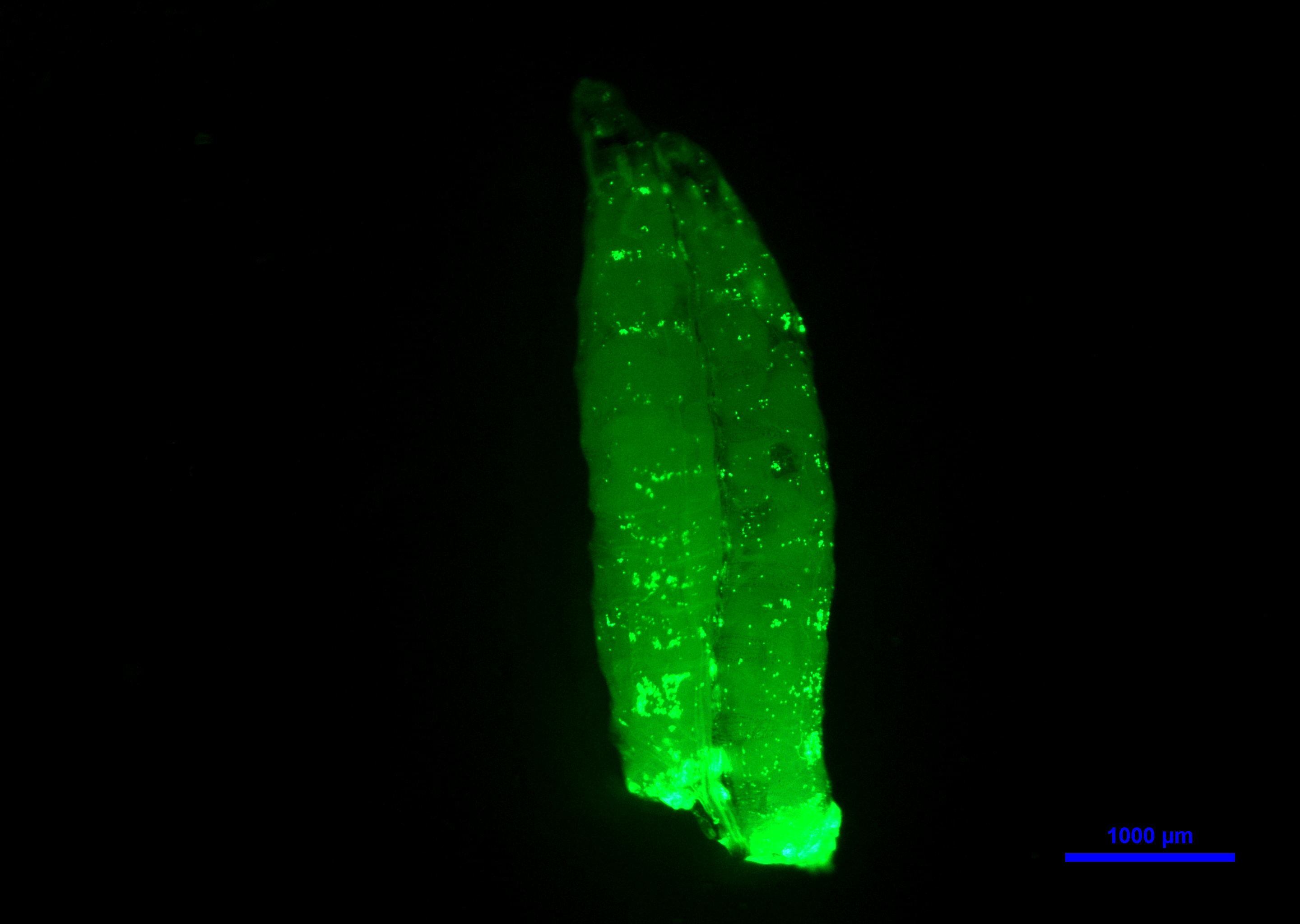

Supplement: Supplementary file 8 — Source data Fig. 6 [file 44319_2025_574_MOESM8_ESM.zip › Fig. 6/Fig. 6 a-g/BmmRNAi_larvae_1.tif]

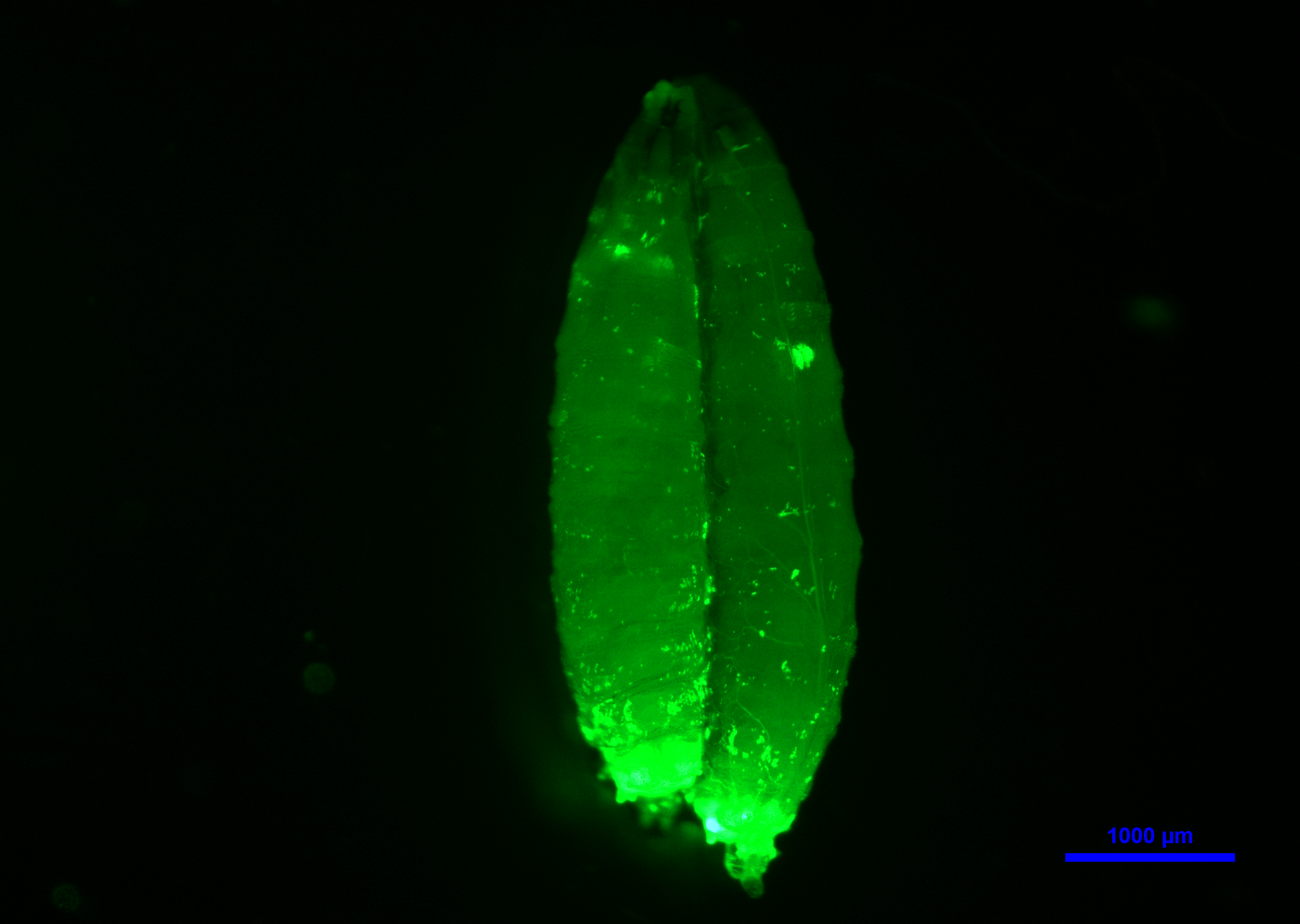

Supplement: Supplementary file 8 — Source data Fig. 6 [file 44319_2025_574_MOESM8_ESM.zip › Fig. 6/Fig. 6 a-g/crqRNAi_larvae_2.tif]

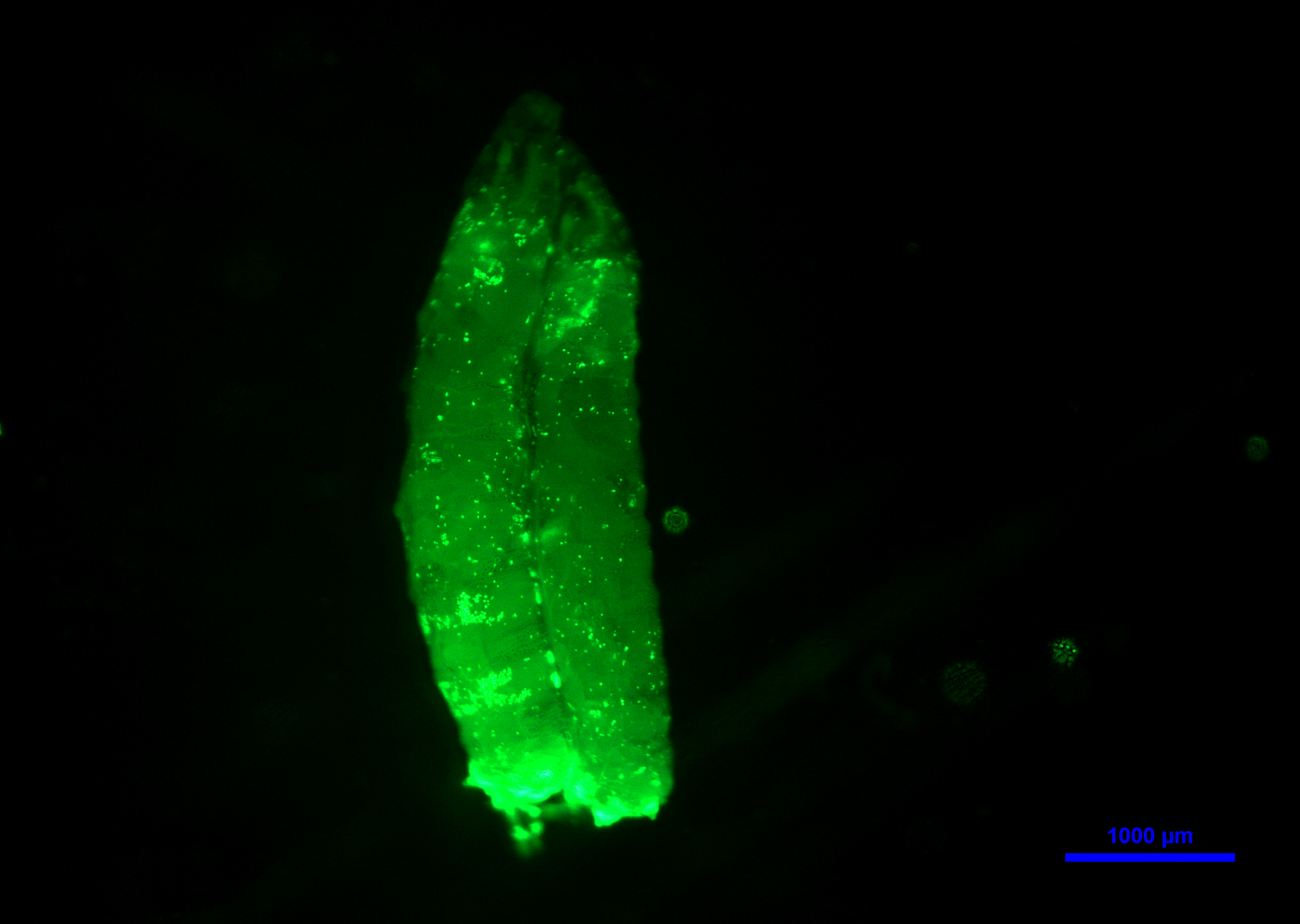

Supplement: Supplementary file 8 — Source data Fig. 6 [file 44319_2025_574_MOESM8_ESM.zip › Fig. 6/Fig. 6 a-g/BmmRNAi_larvae_2.tif]

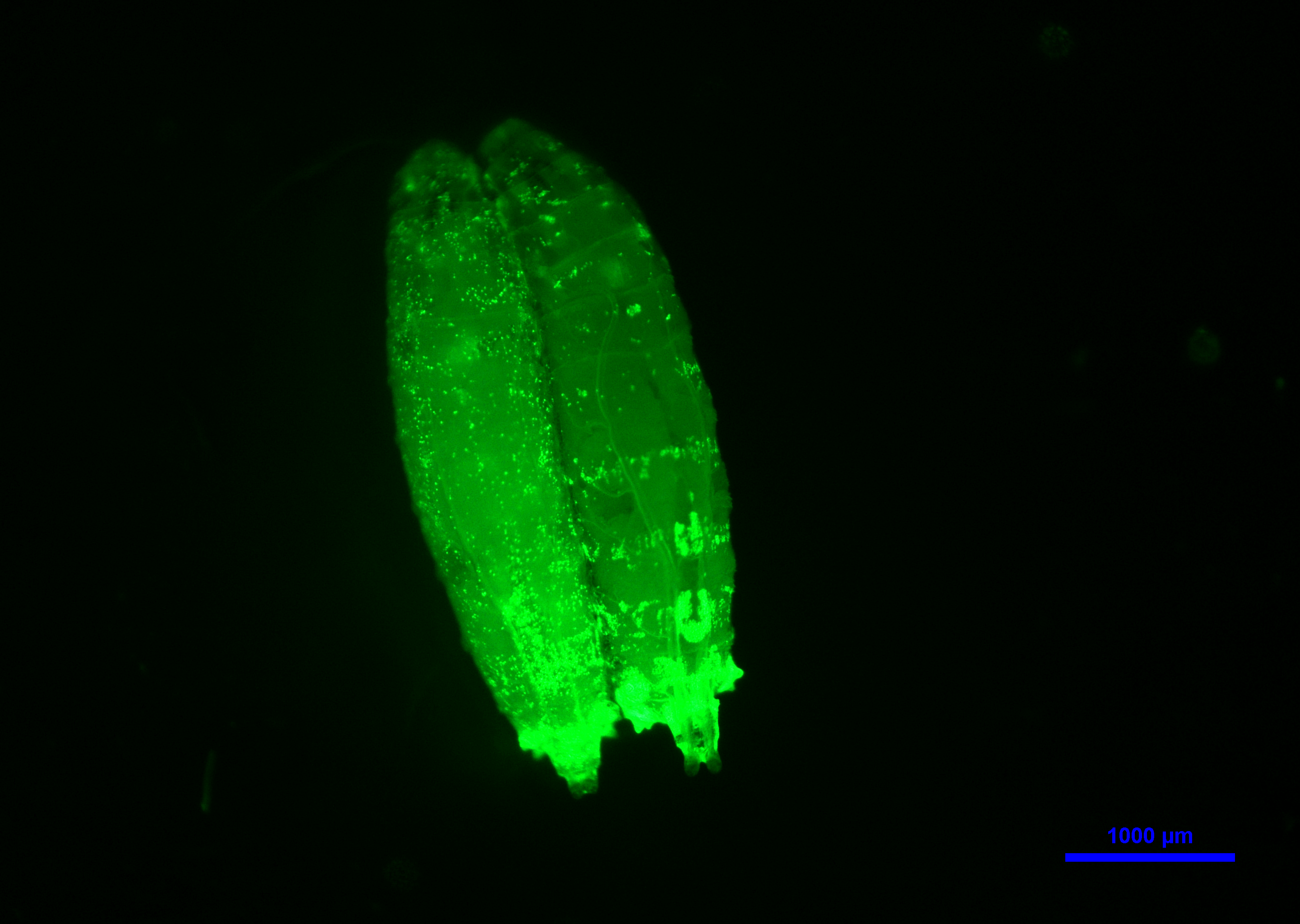

Supplement: Supplementary file 8 — Source data Fig. 6 [file 44319_2025_574_MOESM8_ESM.zip › Fig. 6/Fig. 6 a-g/Control_larvae_1.tif]

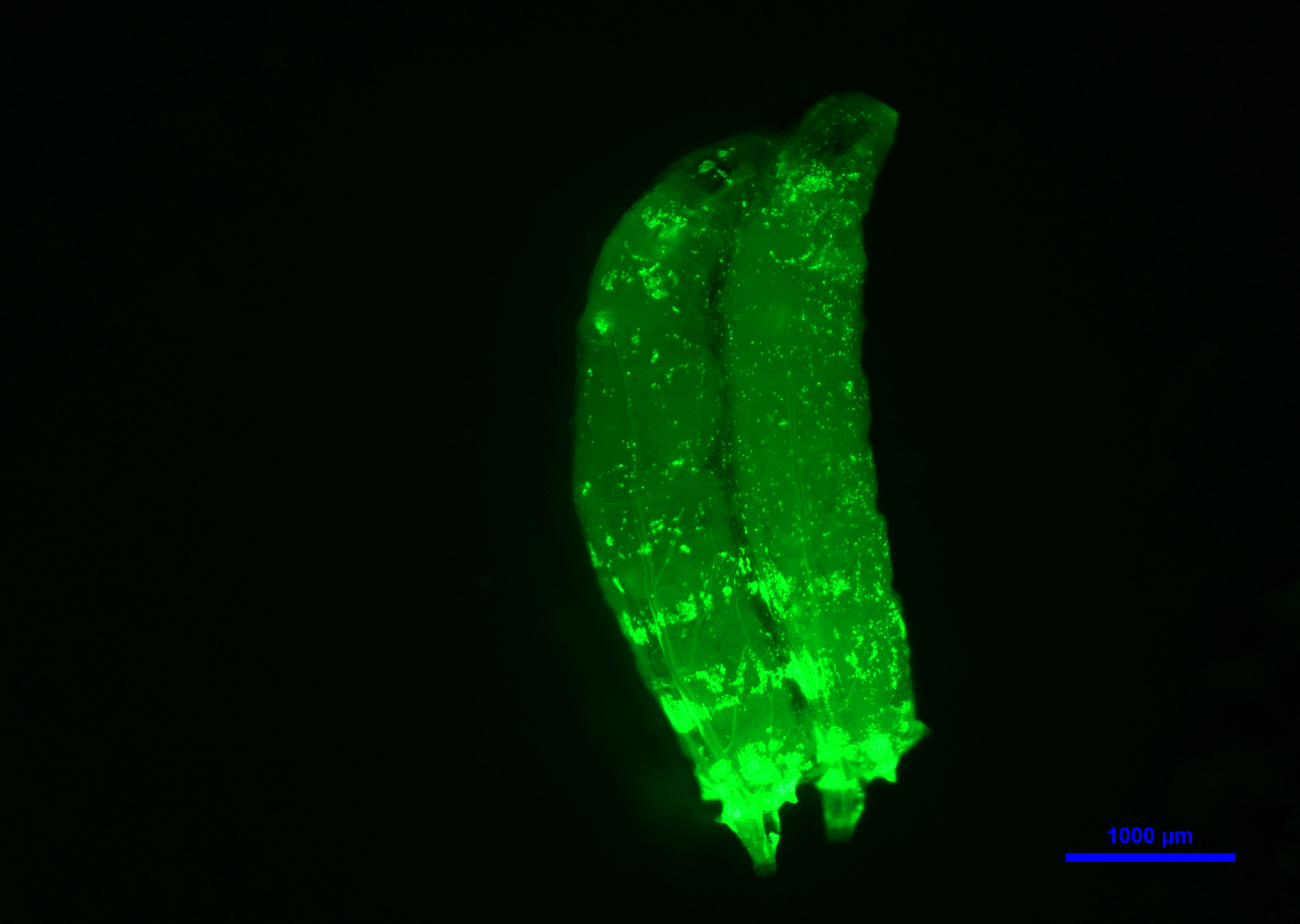

Supplement: Supplementary file 8 — Source data Fig. 6 [file 44319_2025_574_MOESM8_ESM.zip › Fig. 6/Fig. 6 a-g/Control_larvae_2.tif]

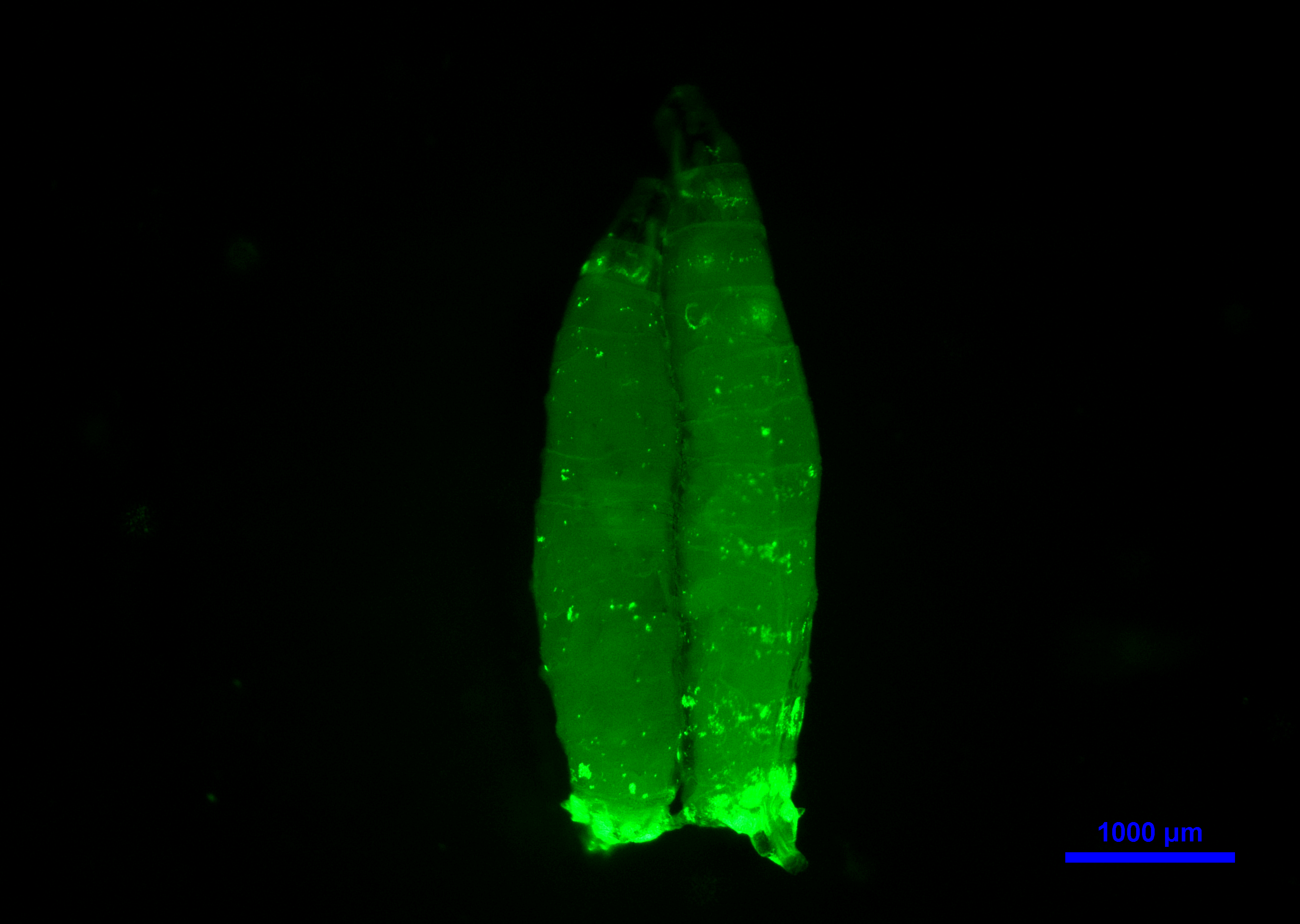

Supplement: Supplementary file 8 — Source data Fig. 6 [file 44319_2025_574_MOESM8_ESM.zip › Fig. 6/Fig. 6 a-g/Gpat4RNAi_larvae_2.tif]

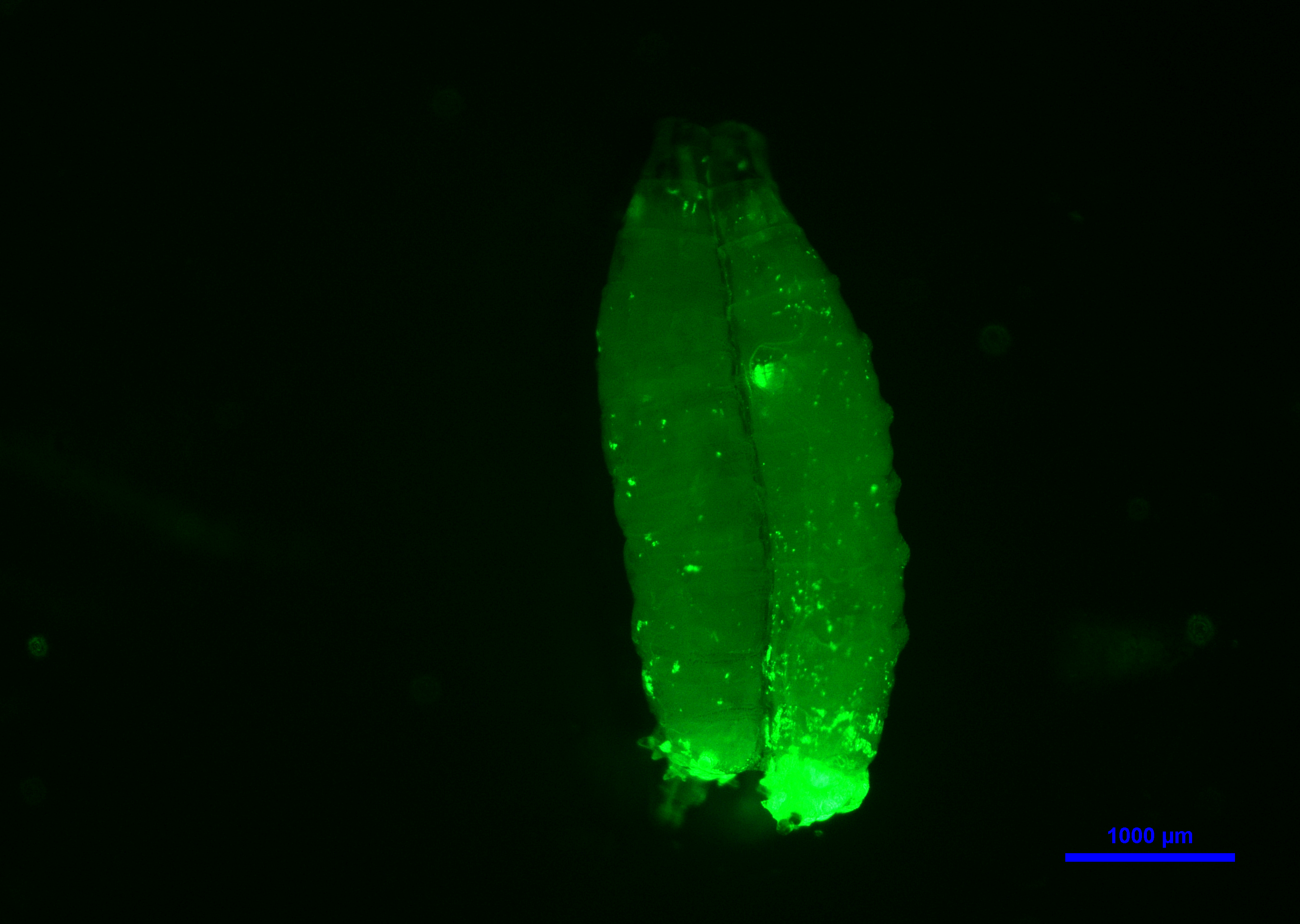

Supplement: Supplementary file 8 — Source data Fig. 6 [file 44319_2025_574_MOESM8_ESM.zip › Fig. 6/Fig. 6 a-g/Gpat4RNAi_larvae_1.tif]

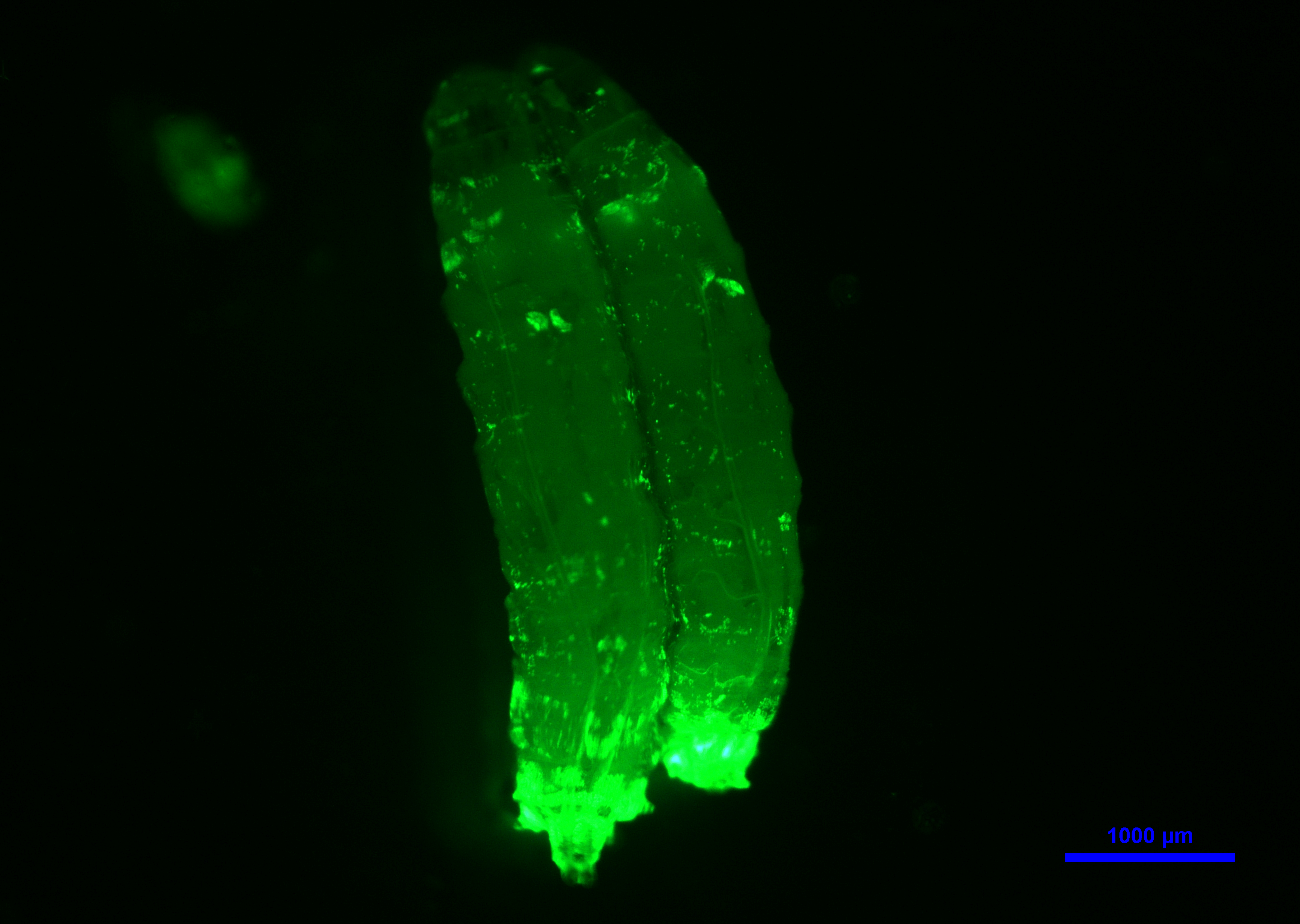

Supplement: Supplementary file 8 — Source data Fig. 6 [file 44319_2025_574_MOESM8_ESM.zip › Fig. 6/Fig. 6 a-g/LdhRNAi_larvae_2.tif]

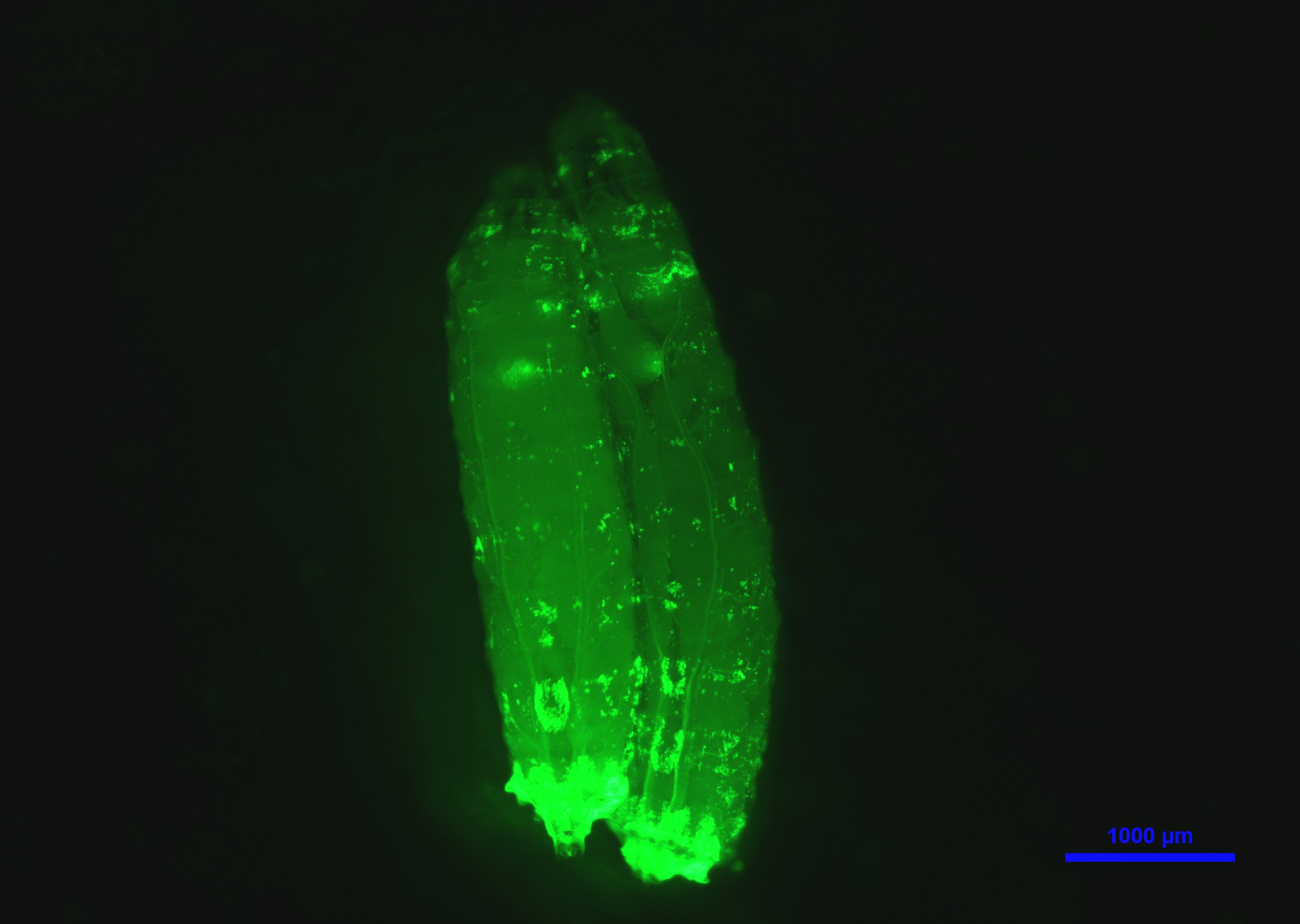

Supplement: Supplementary file 8 — Source data Fig. 6 [file 44319_2025_574_MOESM8_ESM.zip › Fig. 6/Fig. 6 a-g/LdhRNAi_larvae_1.tif]

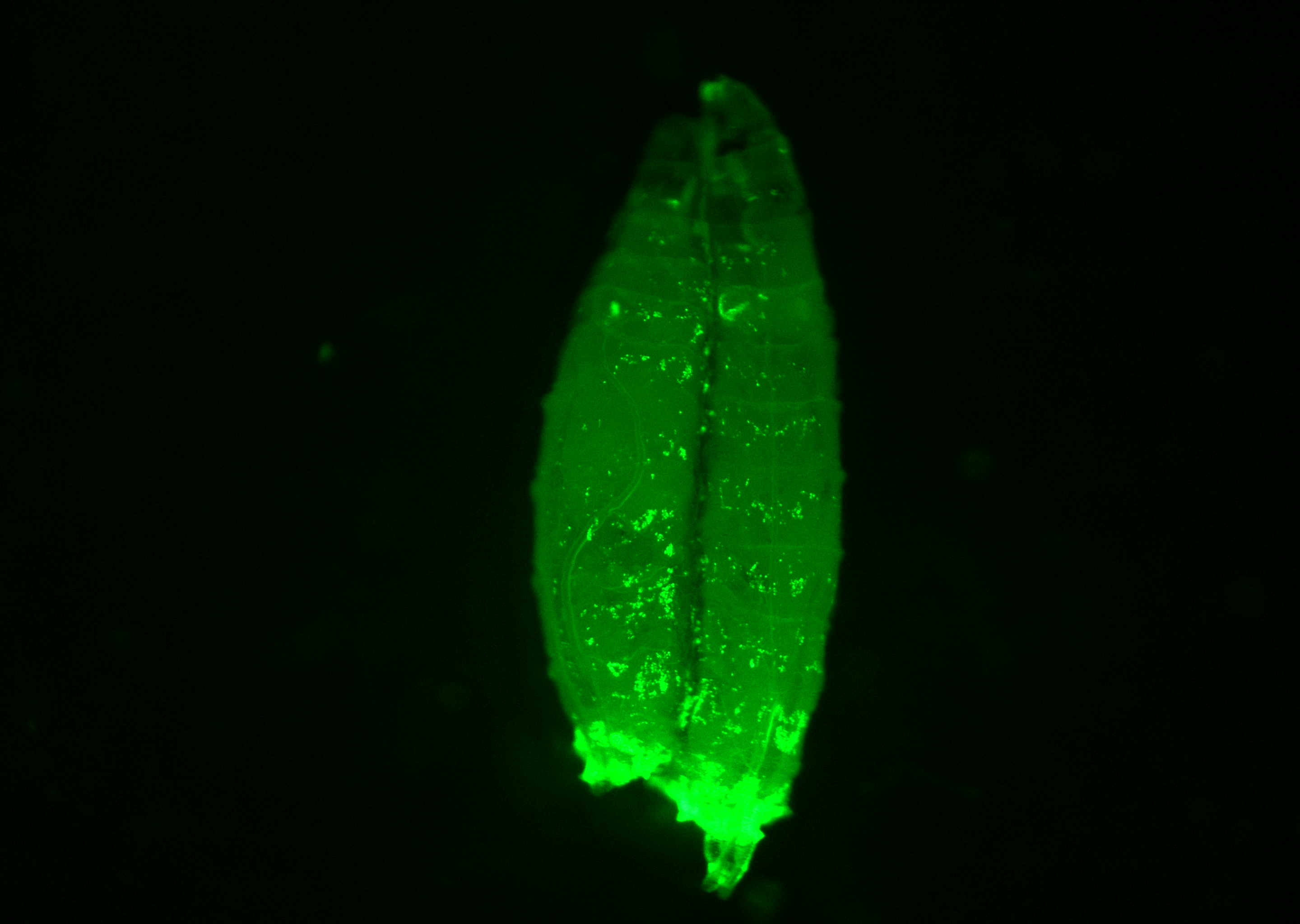

Supplement: Supplementary file 8 — Source data Fig. 6 [file 44319_2025_574_MOESM8_ESM.zip › Fig. 6/Fig. 6 a-g/UAS-ACC_larvae_2.tif]

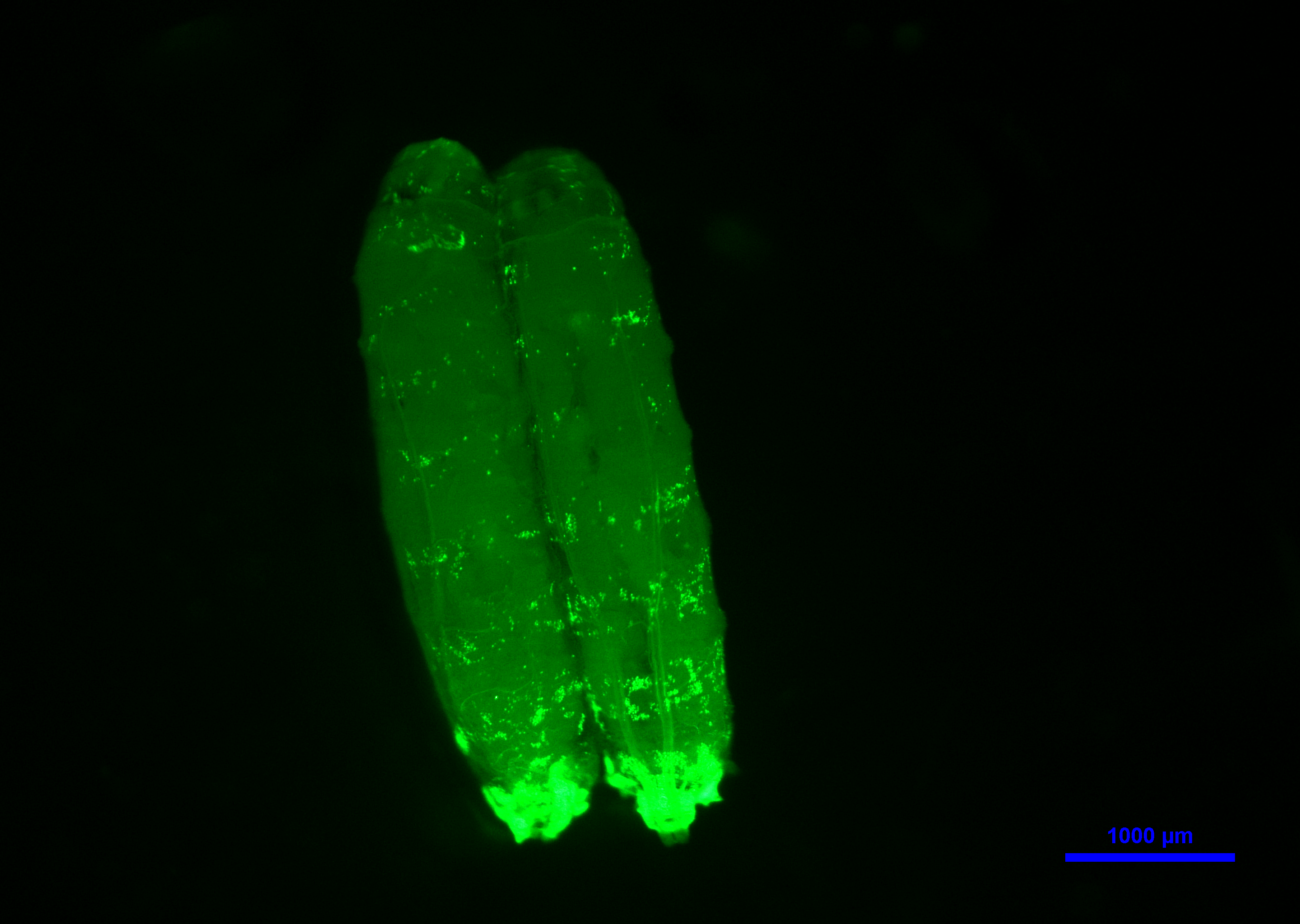

Supplement: Supplementary file 8 — Source data Fig. 6 [file 44319_2025_574_MOESM8_ESM.zip › Fig. 6/Fig. 6 a-g/UAS-ACC_larvae_1.tif]

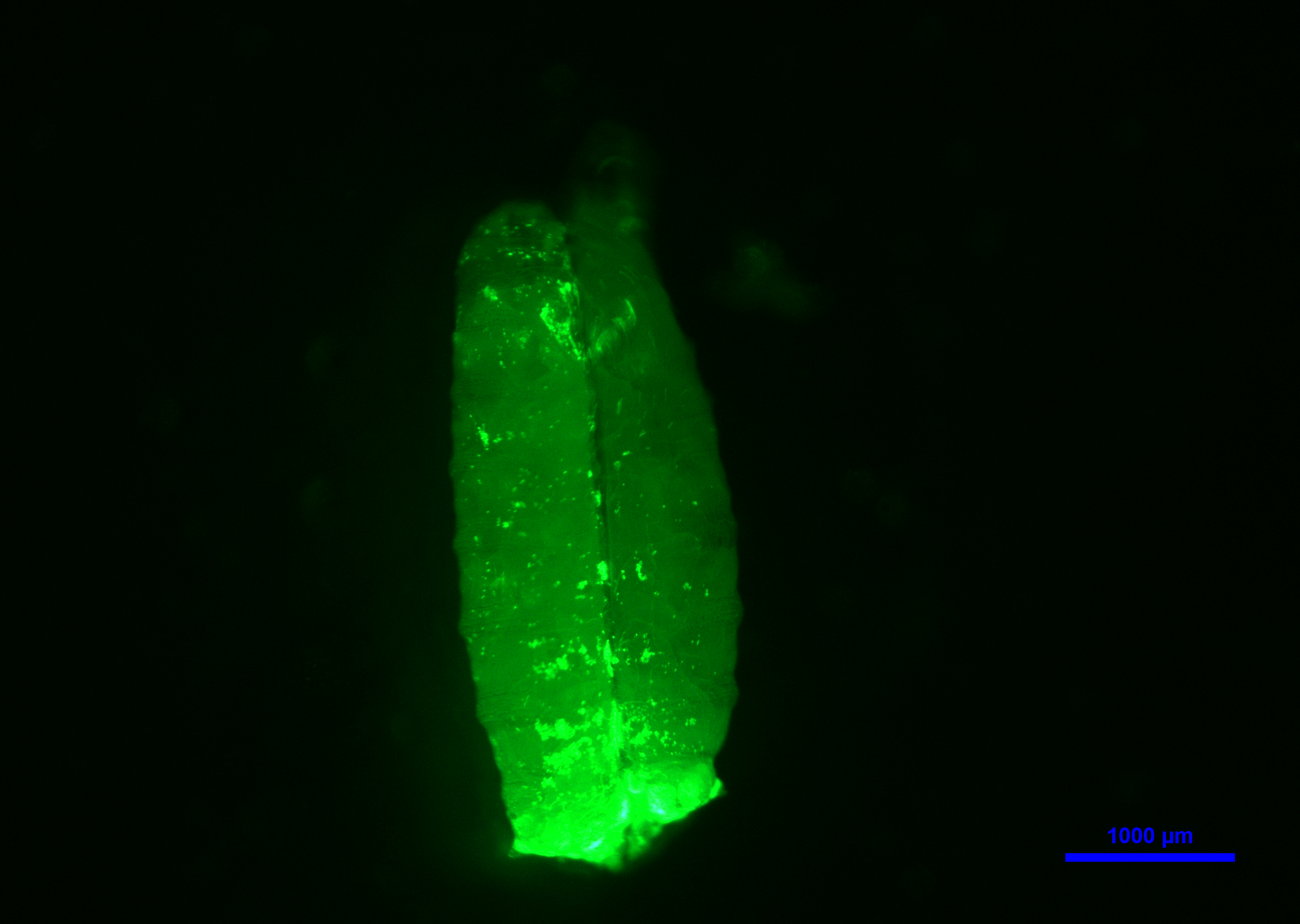

Supplement: Supplementary file 8 — Source data Fig. 6 [file 44319_2025_574_MOESM8_ESM.zip › Fig. 6/Fig. 6 a-g/ACCRNAi_larvae_2.tif]

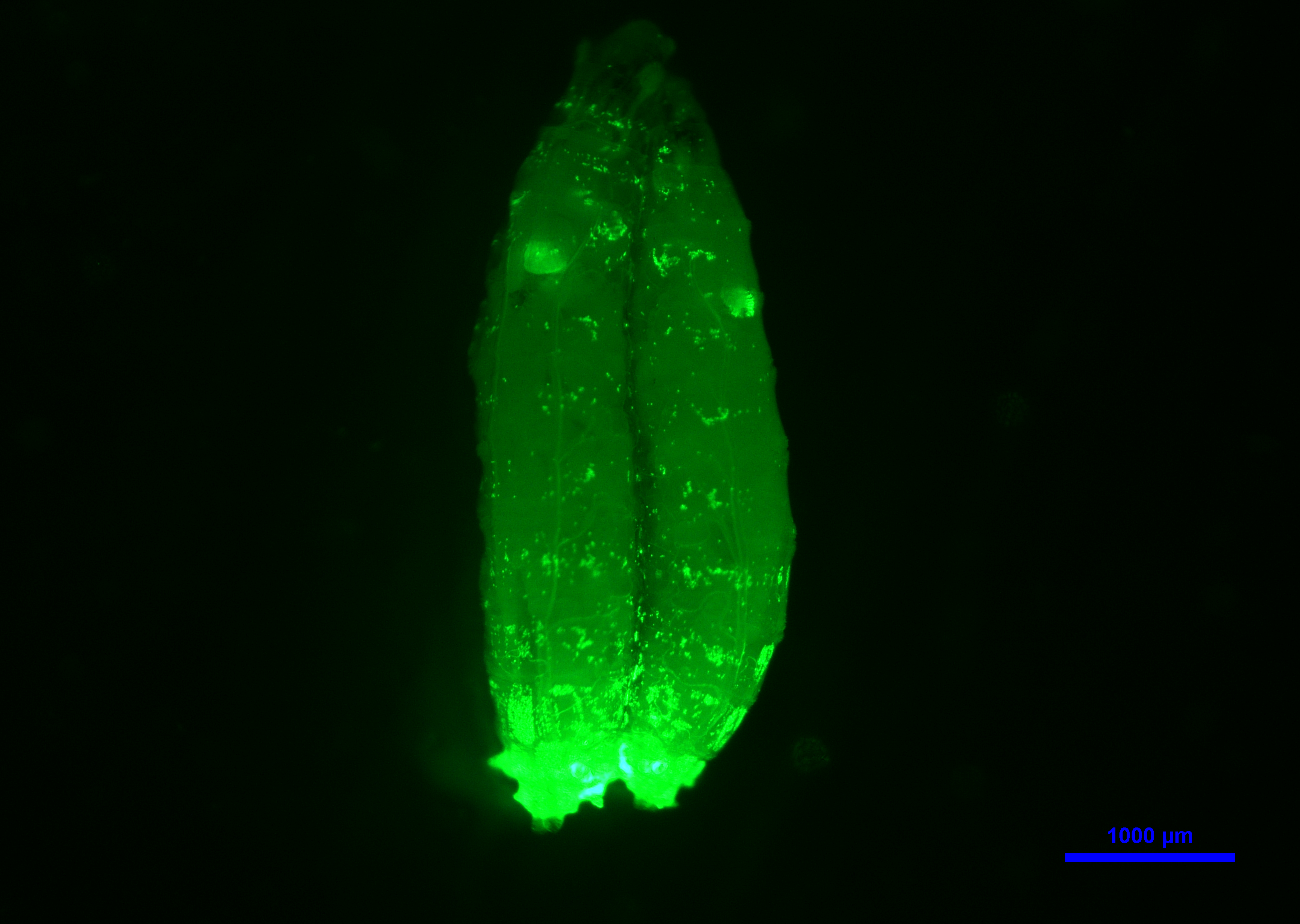

Supplement: Supplementary file 8 — Source data Fig. 6 [file 44319_2025_574_MOESM8_ESM.zip › Fig. 6/Fig. 6 a-g/ACCRNAi_larvae_1.tif]

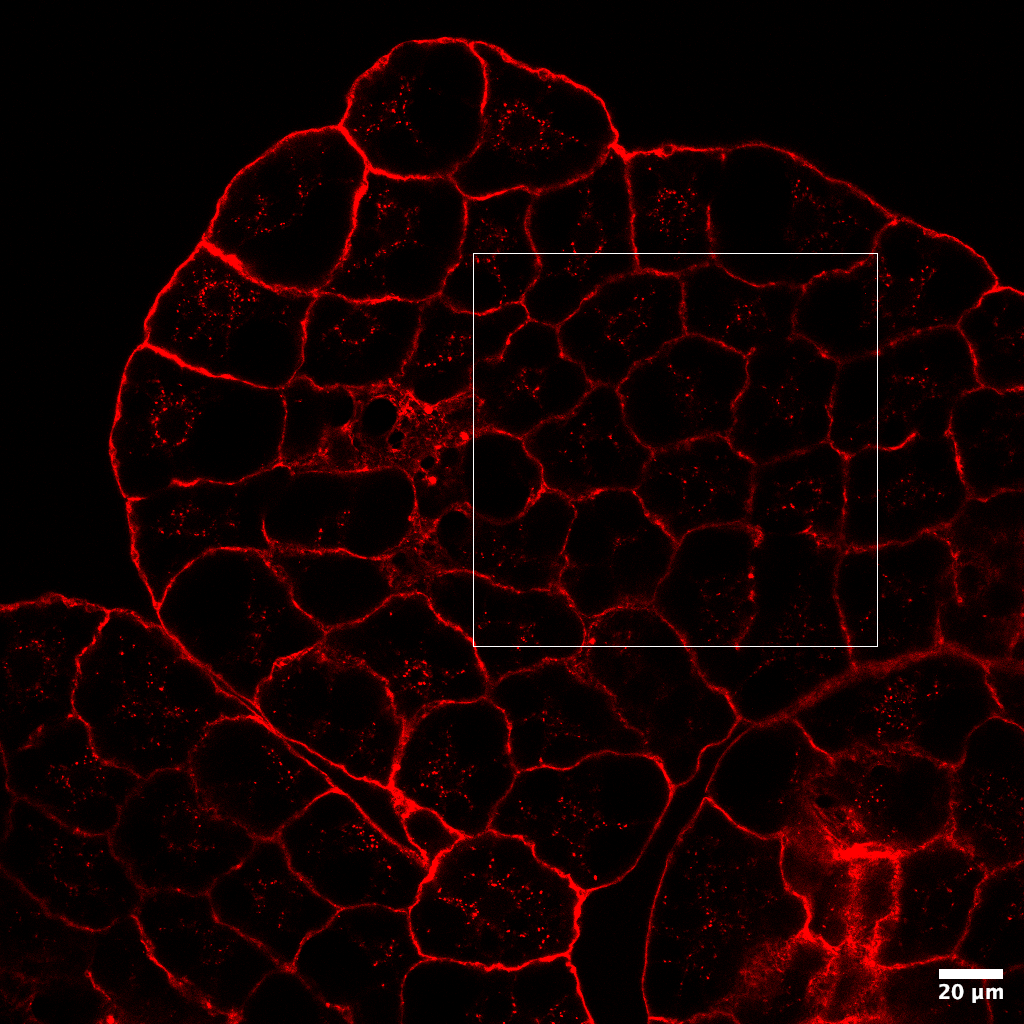

Supplement: Supplementary file 8 — Source data Fig. 6 [file 44319_2025_574_MOESM8_ESM.zip › Fig. 6/Fig. 6 o-u/crqRNAi_fat body.tif]

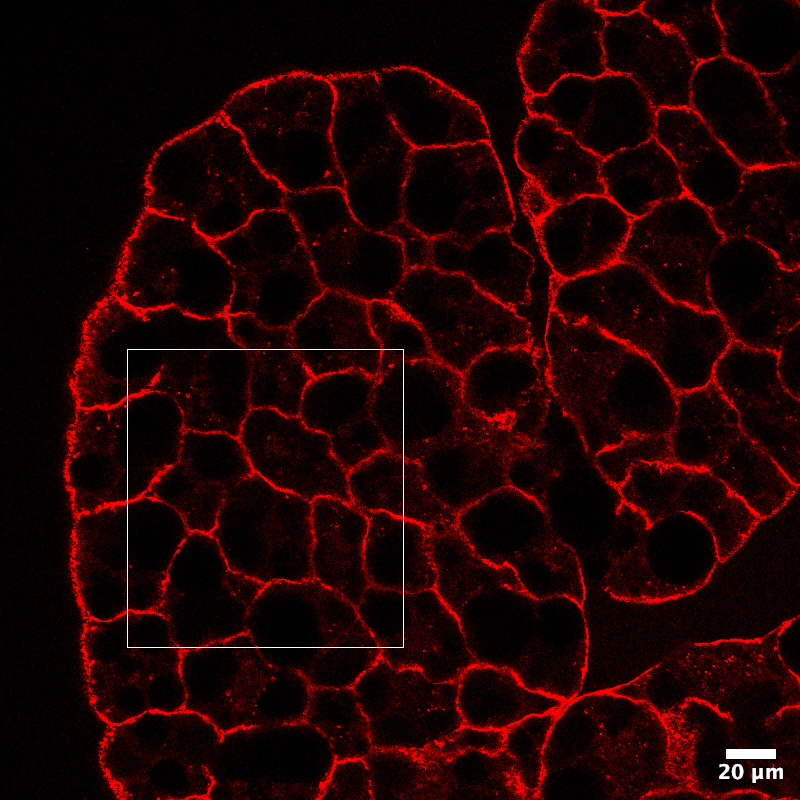

Supplement: Supplementary file 8 — Source data Fig. 6 [file 44319_2025_574_MOESM8_ESM.zip › Fig. 6/Fig. 6 o-u/Gpat4RNAi_fat body.tif]

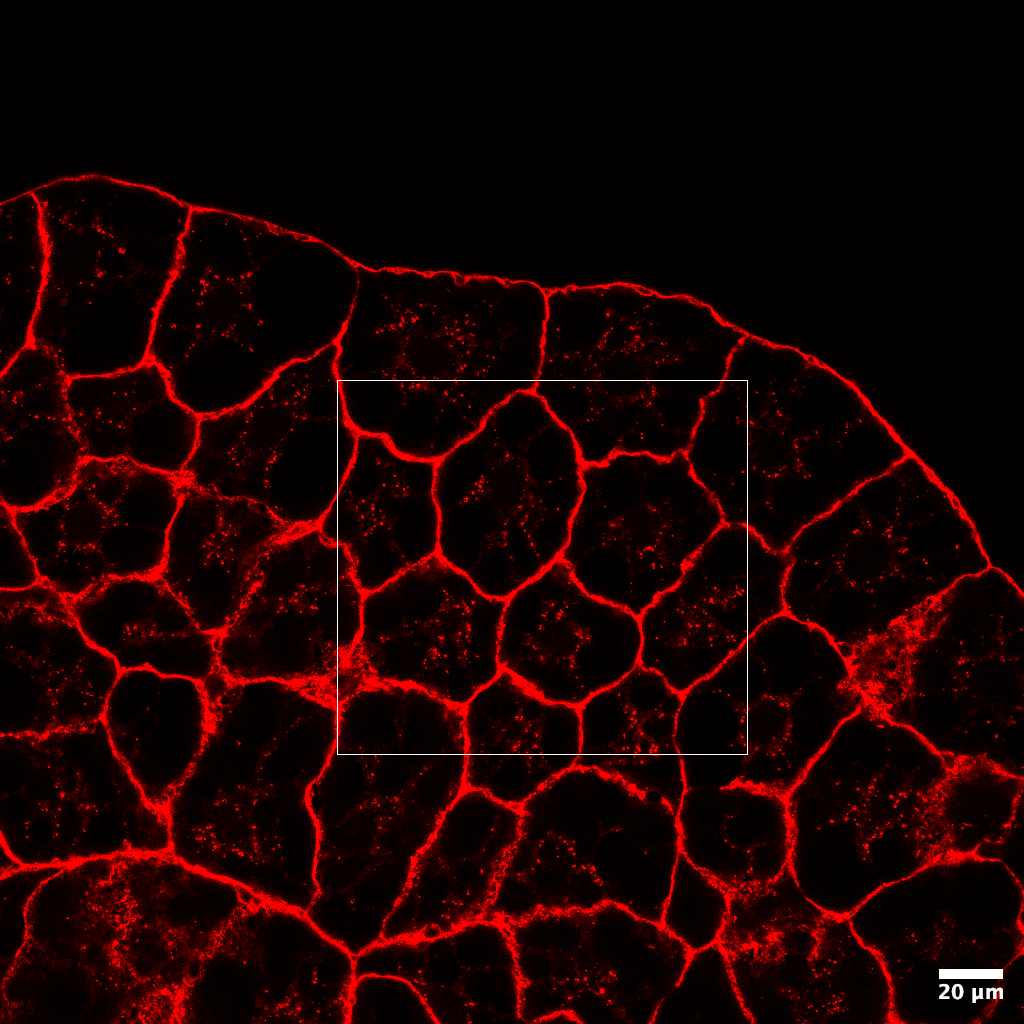

Supplement: Supplementary file 8 — Source data Fig. 6 [file 44319_2025_574_MOESM8_ESM.zip › Fig. 6/Fig. 6 o-u/Control_fat body.tif]

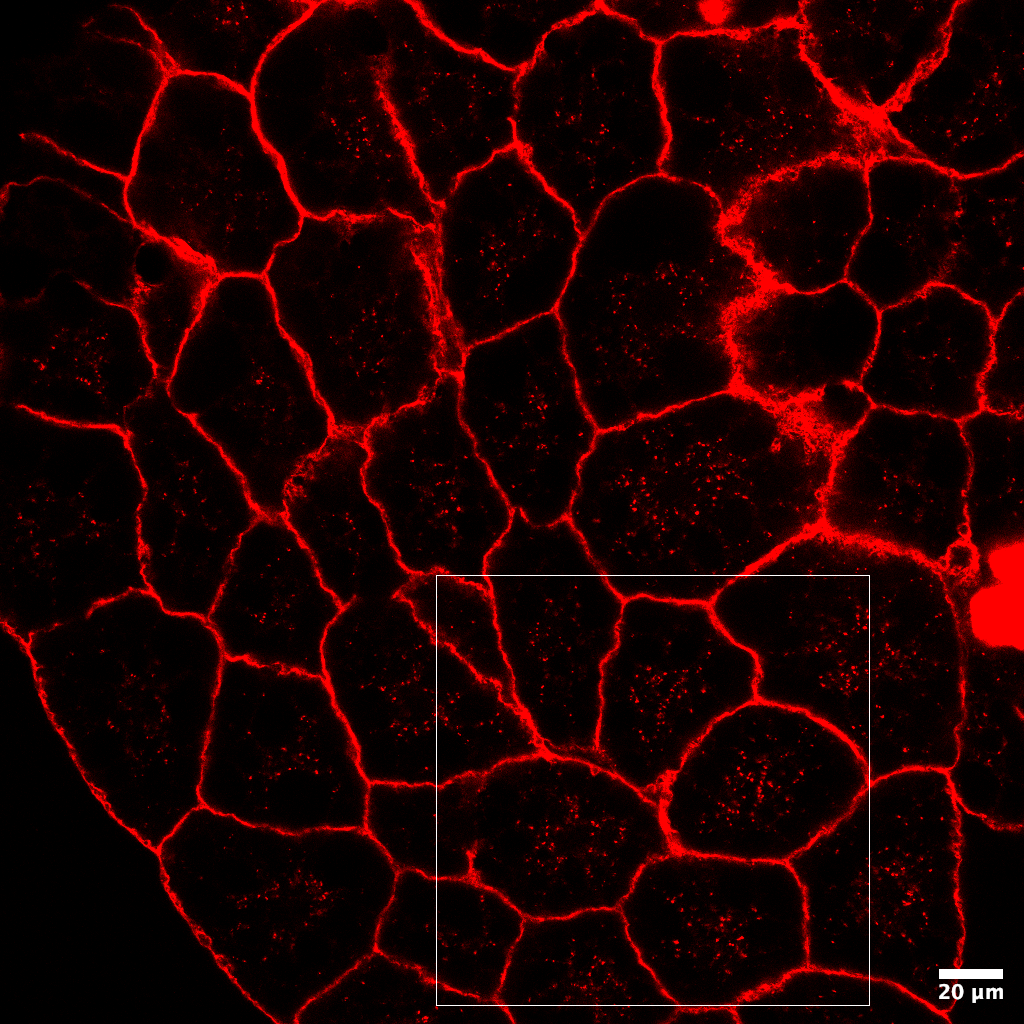

Supplement: Supplementary file 8 — Source data Fig. 6 [file 44319_2025_574_MOESM8_ESM.zip › Fig. 6/Fig. 6 o-u/LdhRNAi_fat body.tif]

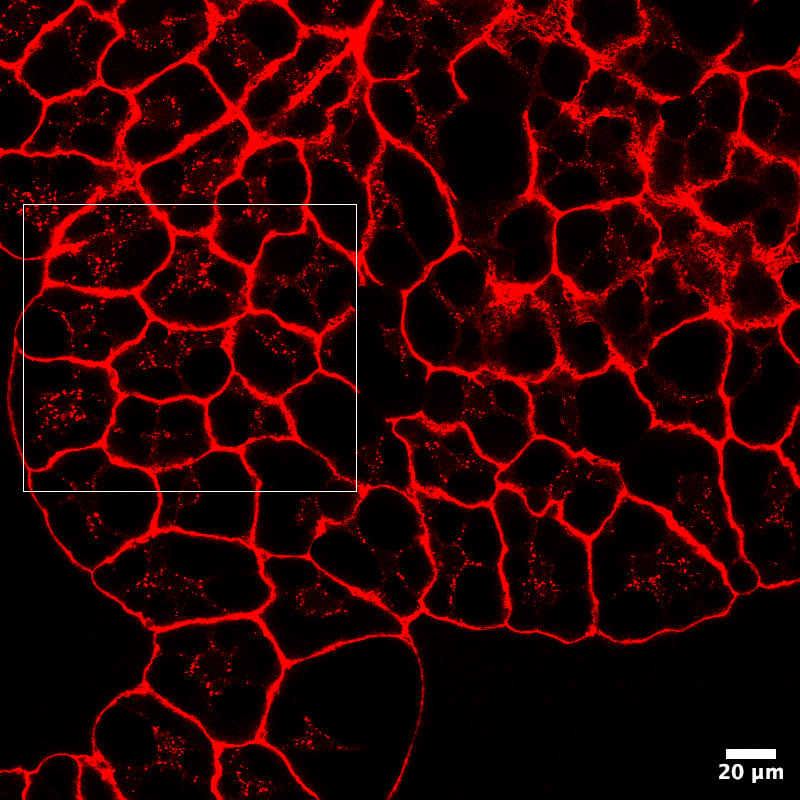

Supplement: Supplementary file 8 — Source data Fig. 6 [file 44319_2025_574_MOESM8_ESM.zip › Fig. 6/Fig. 6 o-u/ACCRNAi_fat body.tif]

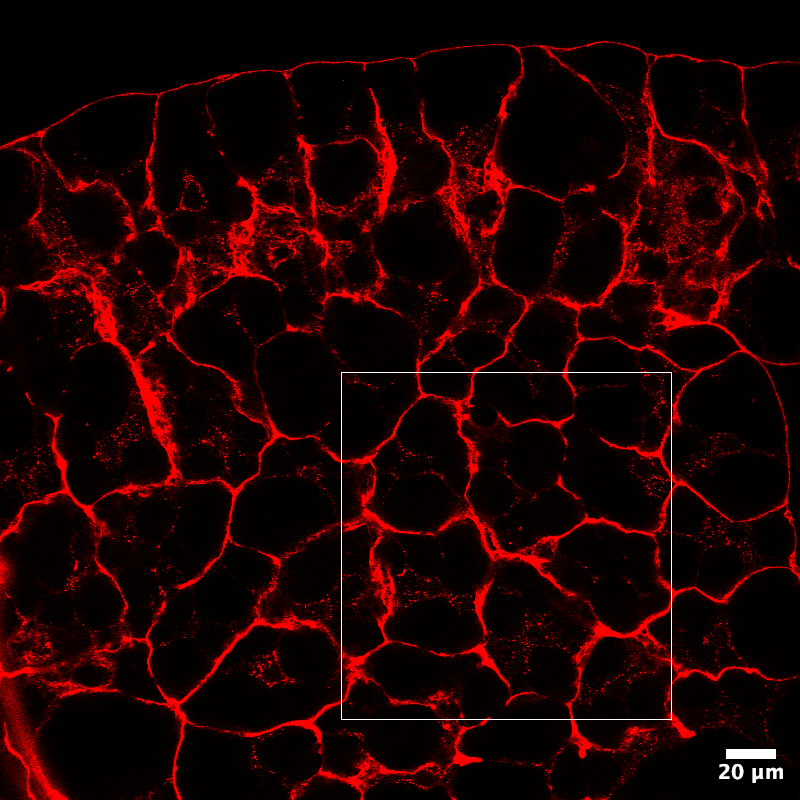

Supplement: Supplementary file 8 — Source data Fig. 6 [file 44319_2025_574_MOESM8_ESM.zip › Fig. 6/Fig. 6 o-u/bmmRNAi_fatbody.tif]

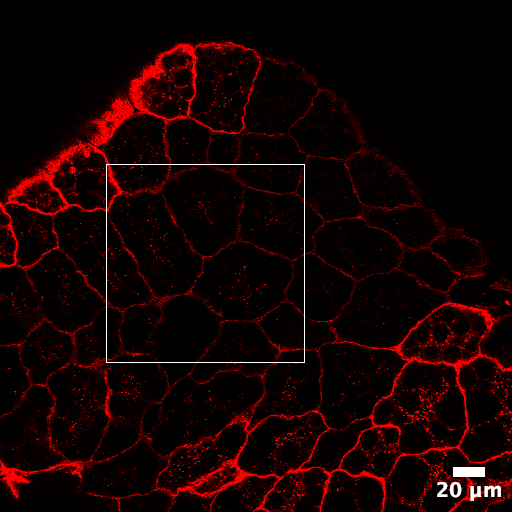

Supplement: Supplementary file 8 — Source data Fig. 6 [file 44319_2025_574_MOESM8_ESM.zip › Fig. 6/Fig. 6 o-u/UAS-ACC_fat body.tif]

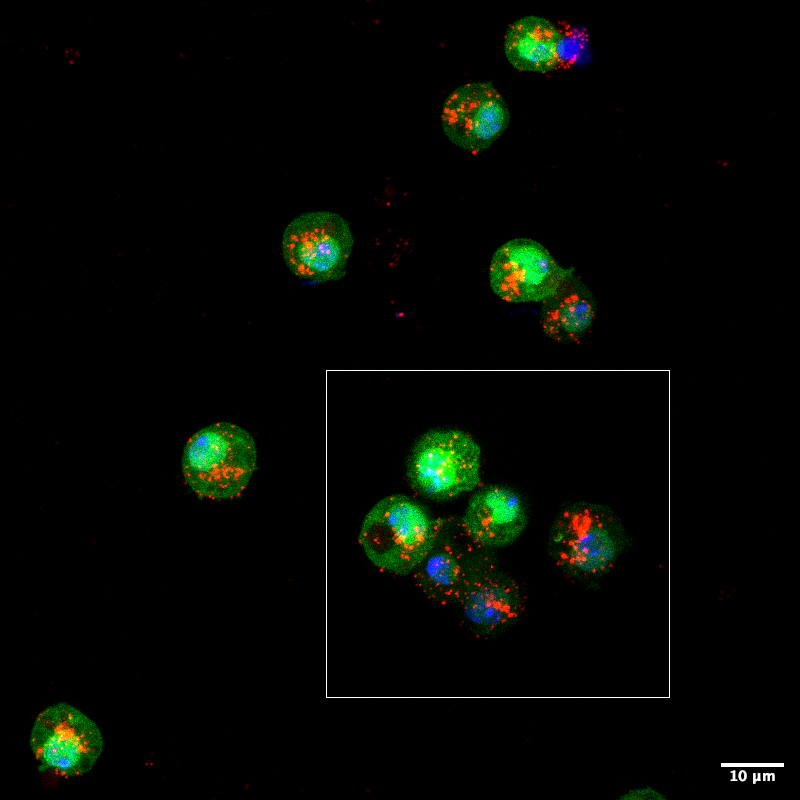

Supplement: Supplementary file 9 — Figure EV1 Source Data [file 44319_2025_574_MOESM9_ESM.zip › Fig. EV1/Fig. EV1_e-e''/4hr.HSD_phagocytosis.tif]

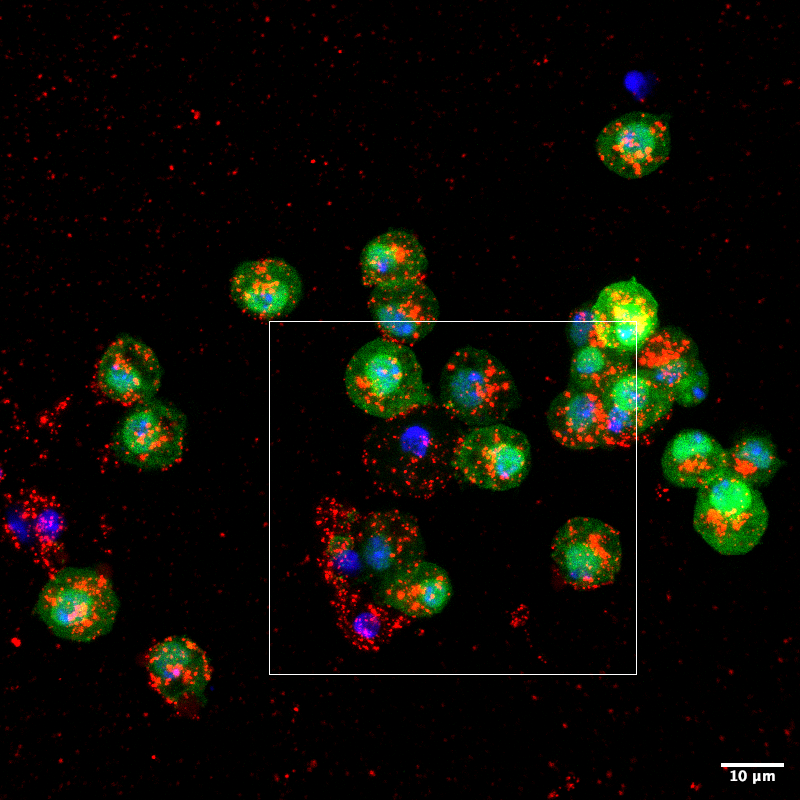

Supplement: Supplementary file 9 — Figure EV1 Source Data [file 44319_2025_574_MOESM9_ESM.zip › Fig. EV1/Fig. EV1_e-e''/RF_phagocytosis.tif]

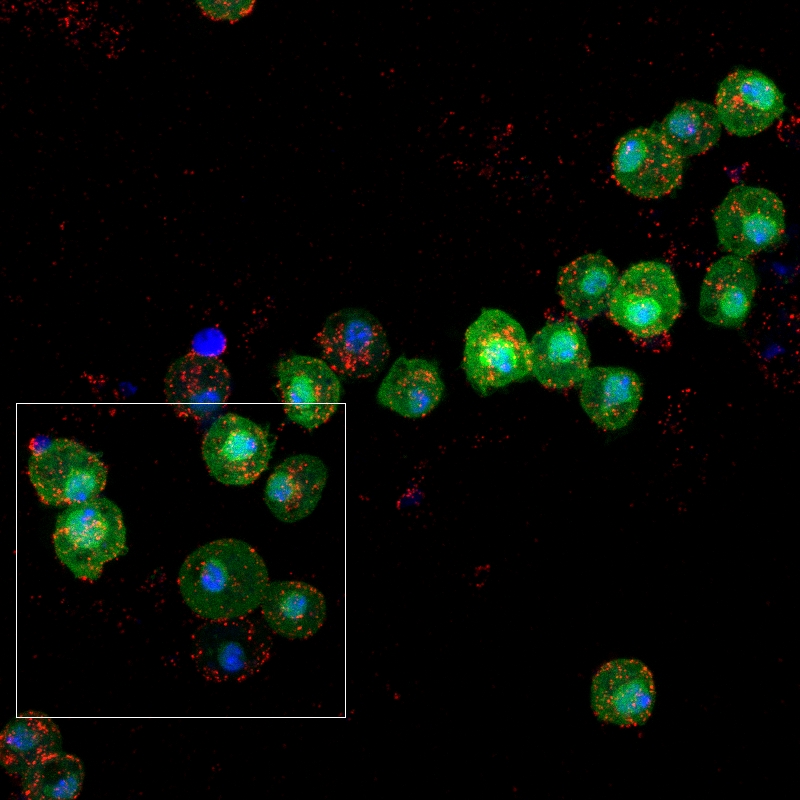

Supplement: Supplementary file 9 — Figure EV1 Source Data [file 44319_2025_574_MOESM9_ESM.zip › Fig. EV1/Fig. EV1_e-e''/Ct.HSD_phagocytosis.tif]

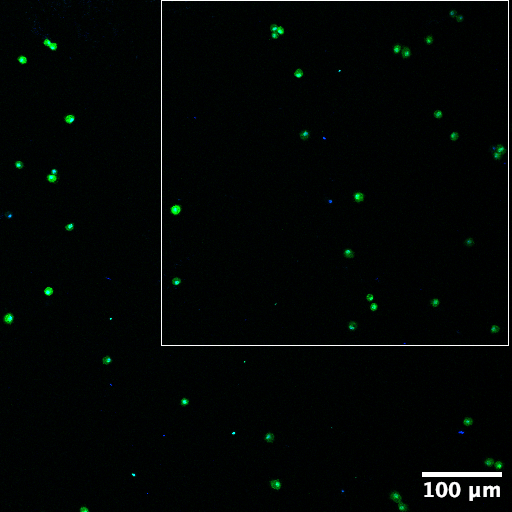

Supplement: Supplementary file 9 — Figure EV1 Source Data [file 44319_2025_574_MOESM9_ESM.zip › Fig. EV1/Fig. EV1_a-a''/Ct.HSD_sessile_bleeds.tif]

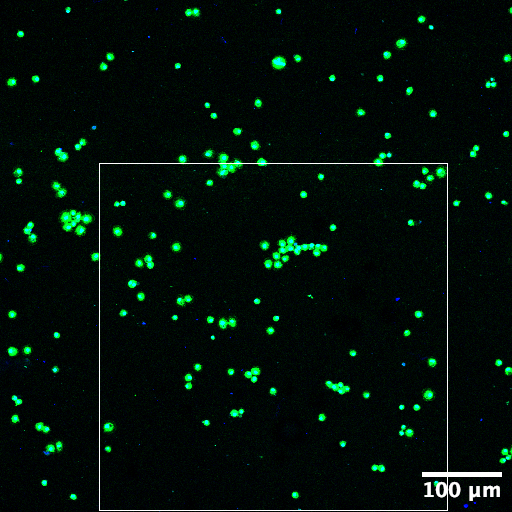

Supplement: Supplementary file 9 — Figure EV1 Source Data [file 44319_2025_574_MOESM9_ESM.zip › Fig. EV1/Fig. EV1_a-a''/4hr.HSD_sessile_bleeds.tif]

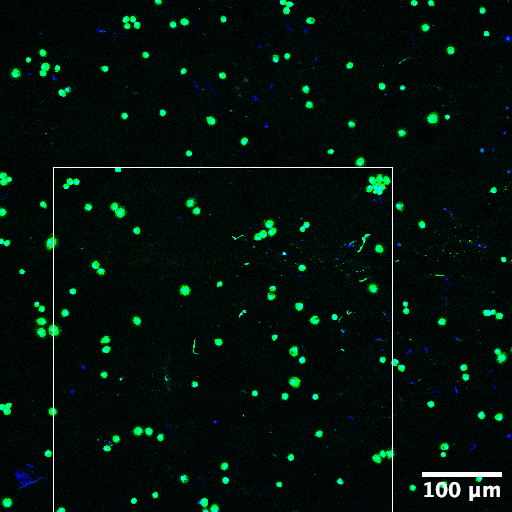

Supplement: Supplementary file 9 — Figure EV1 Source Data [file 44319_2025_574_MOESM9_ESM.zip › Fig. EV1/Fig. EV1_a-a''/RF_sessile_bleeds.tif]

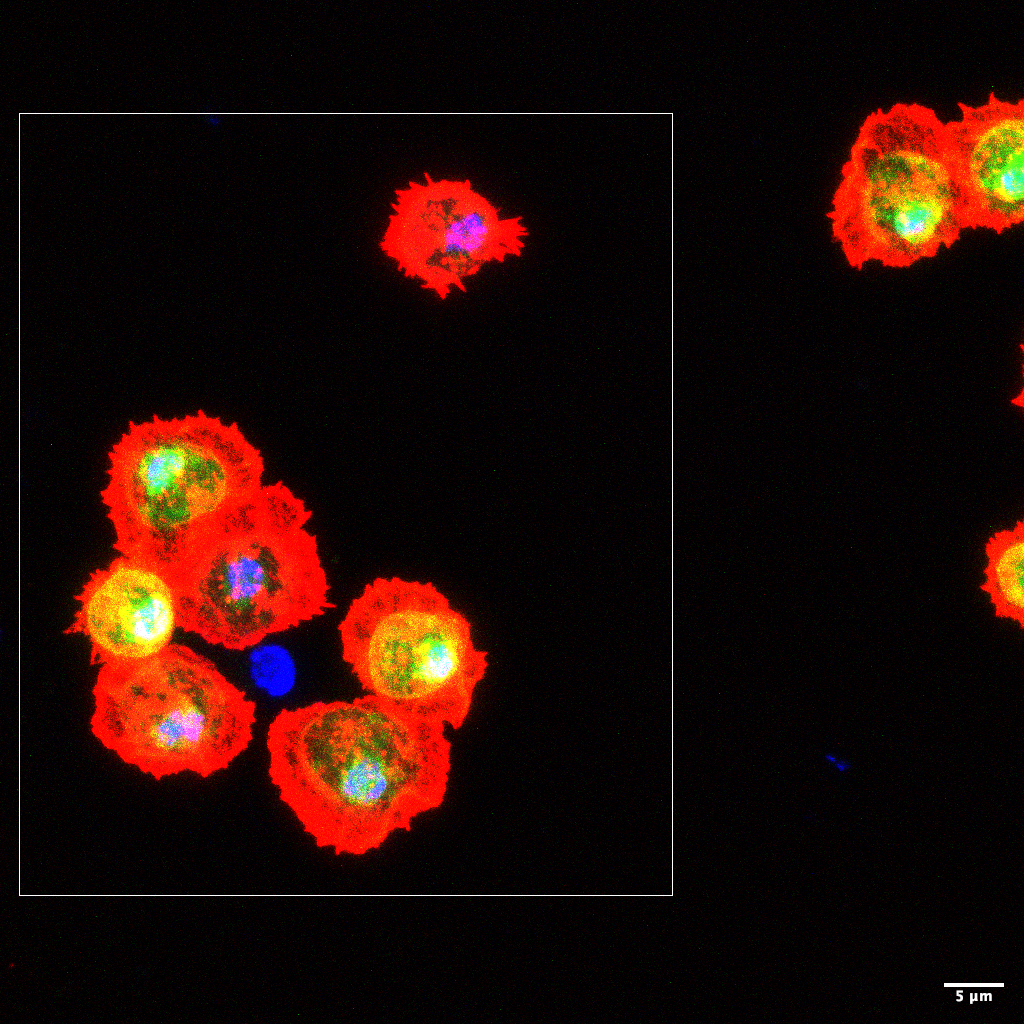

Supplement: Supplementary file 9 — Figure EV1 Source Data [file 44319_2025_574_MOESM9_ESM.zip › Fig. EV1/Fig. EV1_f-f''/Ct.HSD_phalloidin.tif]

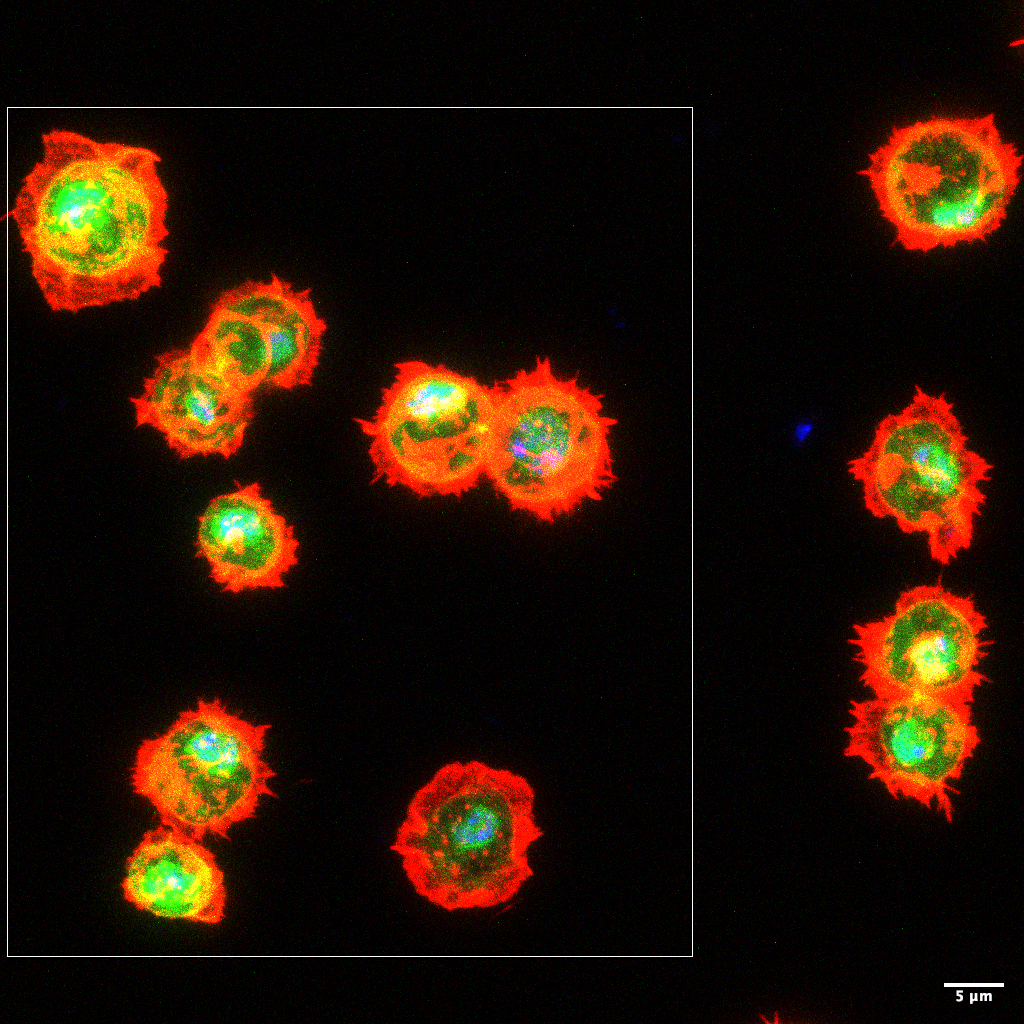

Supplement: Supplementary file 9 — Figure EV1 Source Data [file 44319_2025_574_MOESM9_ESM.zip › Fig. EV1/Fig. EV1_f-f''/4hr.HSD_phalloidin.tif]

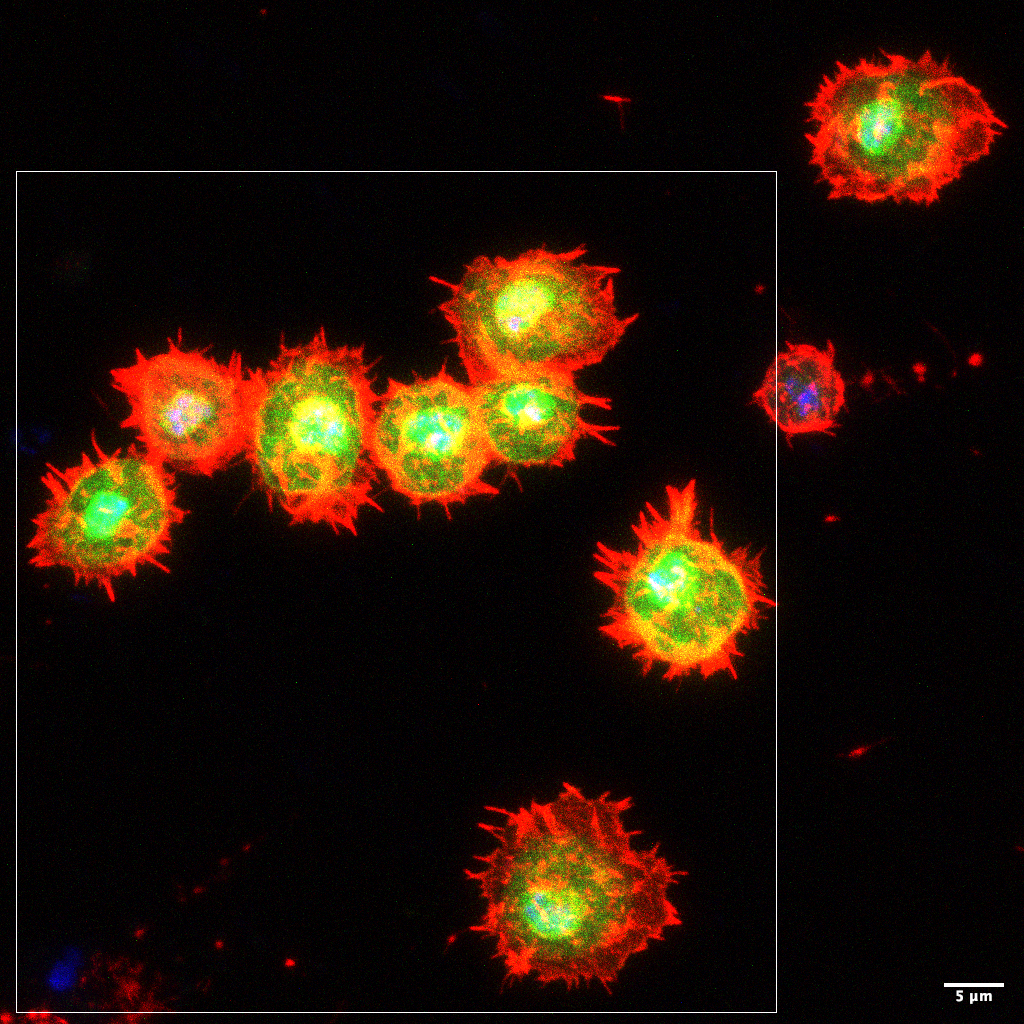

Supplement: Supplementary file 9 — Figure EV1 Source Data [file 44319_2025_574_MOESM9_ESM.zip › Fig. EV1/Fig. EV1_f-f''/RF_phalloidin.tif]

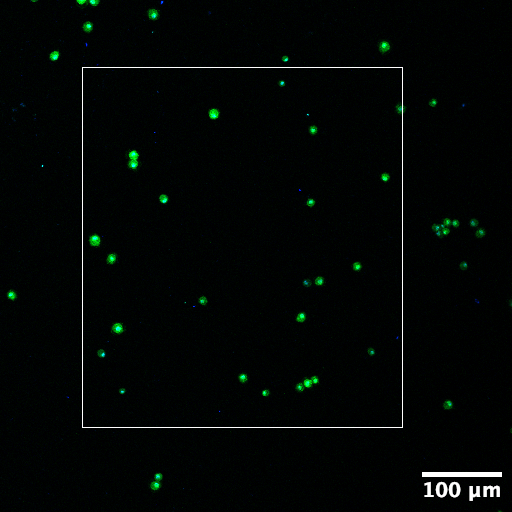

Supplement: Supplementary file 9 — Figure EV1 Source Data [file 44319_2025_574_MOESM9_ESM.zip › Fig. EV1/Fig. EV1_b-b''/RF_circulatory_bleeds.tif]

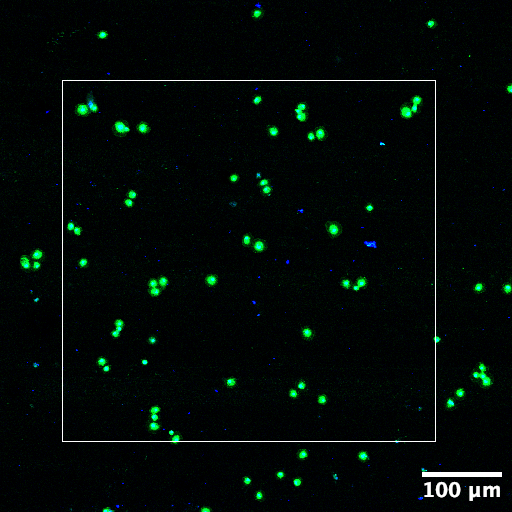

Supplement: Supplementary file 9 — Figure EV1 Source Data [file 44319_2025_574_MOESM9_ESM.zip › Fig. EV1/Fig. EV1_b-b''/4hr.HSD_circulatory_bleeds.tif]

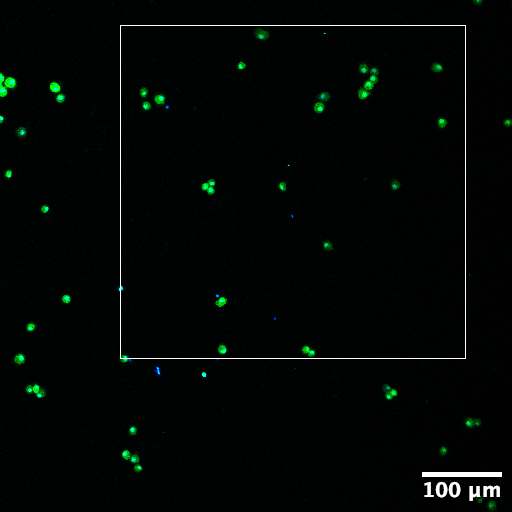

Supplement: Supplementary file 9 — Figure EV1 Source Data [file 44319_2025_574_MOESM9_ESM.zip › Fig. EV1/Fig. EV1_b-b''/Ct.HSD_circulatory_bleeds.tif]

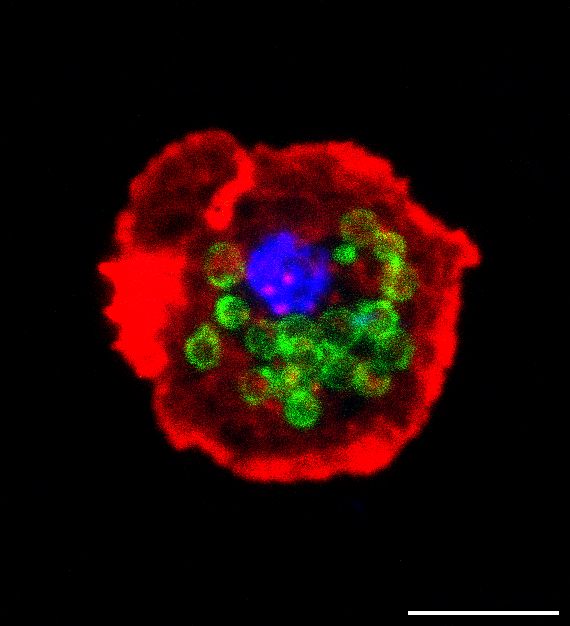

Supplement: Supplementary file 9 — Figure EV1 Source Data [file 44319_2025_574_MOESM9_ESM.zip › Fig. EV1/Fig. EV1_g-g''/Ct.HSD_Lsd-2.GFP.tif]

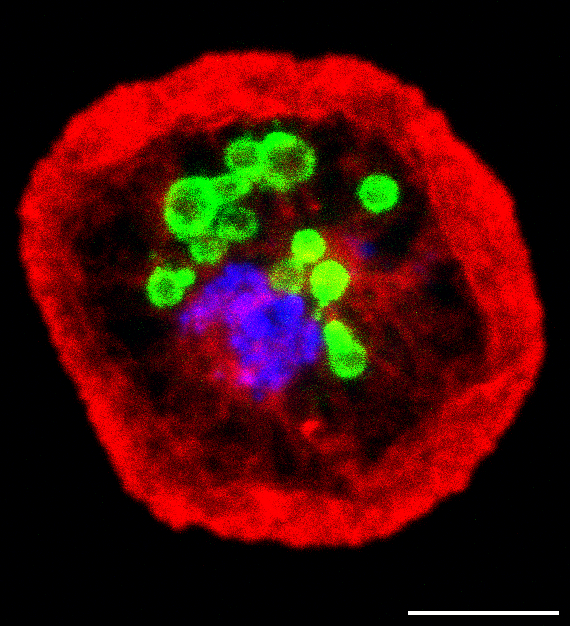

Supplement: Supplementary file 9 — Figure EV1 Source Data [file 44319_2025_574_MOESM9_ESM.zip › Fig. EV1/Fig. EV1_g-g''/4hr.HSD_Lsd-2.GFP.tif]

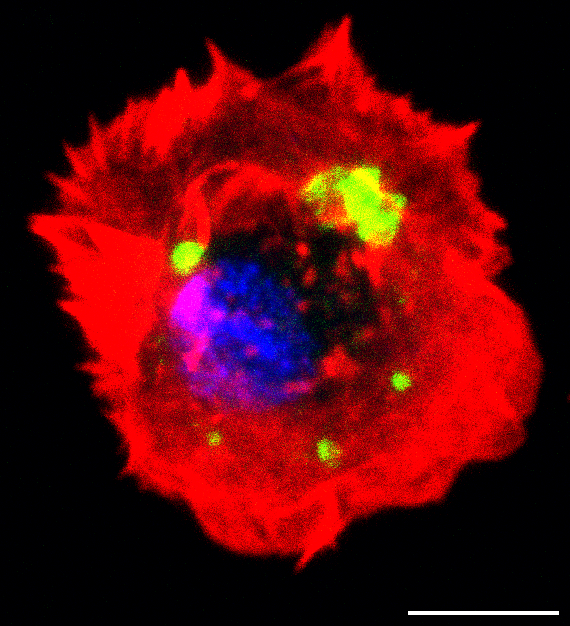

Supplement: Supplementary file 9 — Figure EV1 Source Data [file 44319_2025_574_MOESM9_ESM.zip › Fig. EV1/Fig. EV1_g-g''/RF_Lsd-2.GFP.tif]

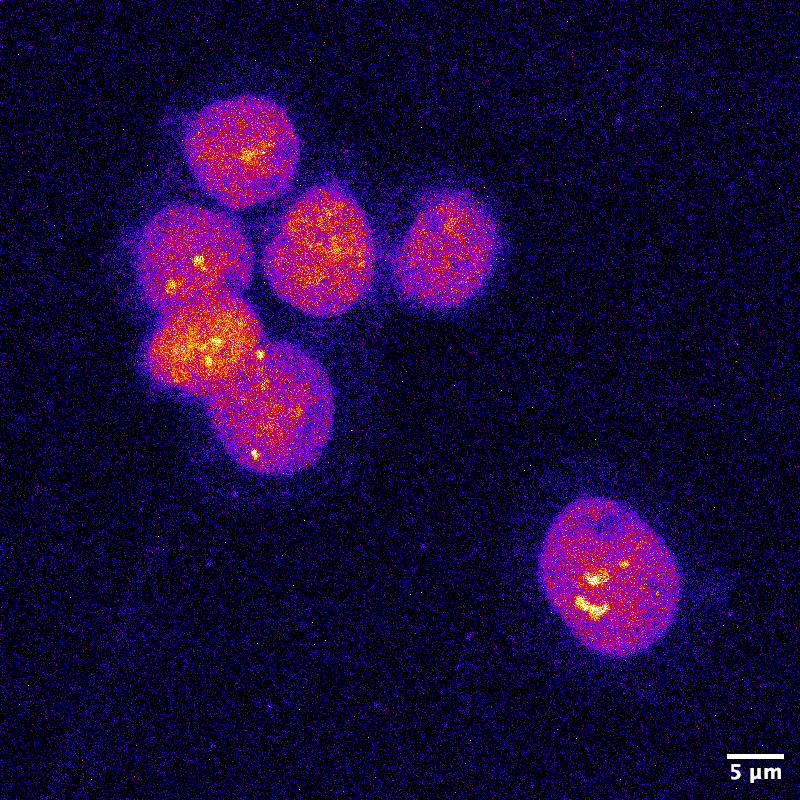

Supplement: Supplementary file 9 — Figure EV1 Source Data [file 44319_2025_574_MOESM9_ESM.zip › Fig. EV1/Fig. EV1_c-c''/4hr.HSD_ROS.tif]

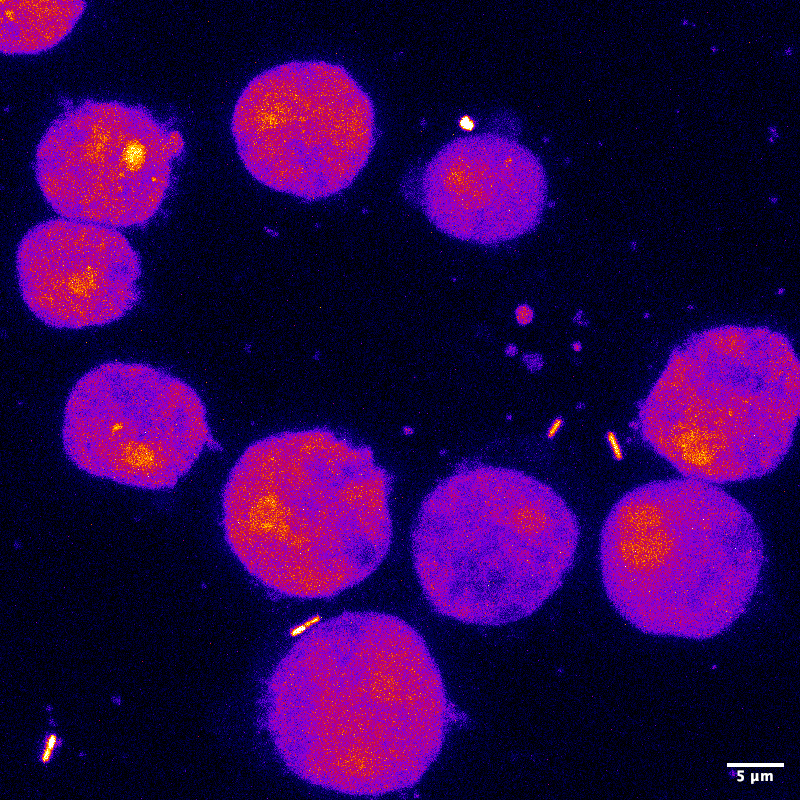

Supplement: Supplementary file 9 — Figure EV1 Source Data [file 44319_2025_574_MOESM9_ESM.zip › Fig. EV1/Fig. EV1_c-c''/RF_ROS.tif]

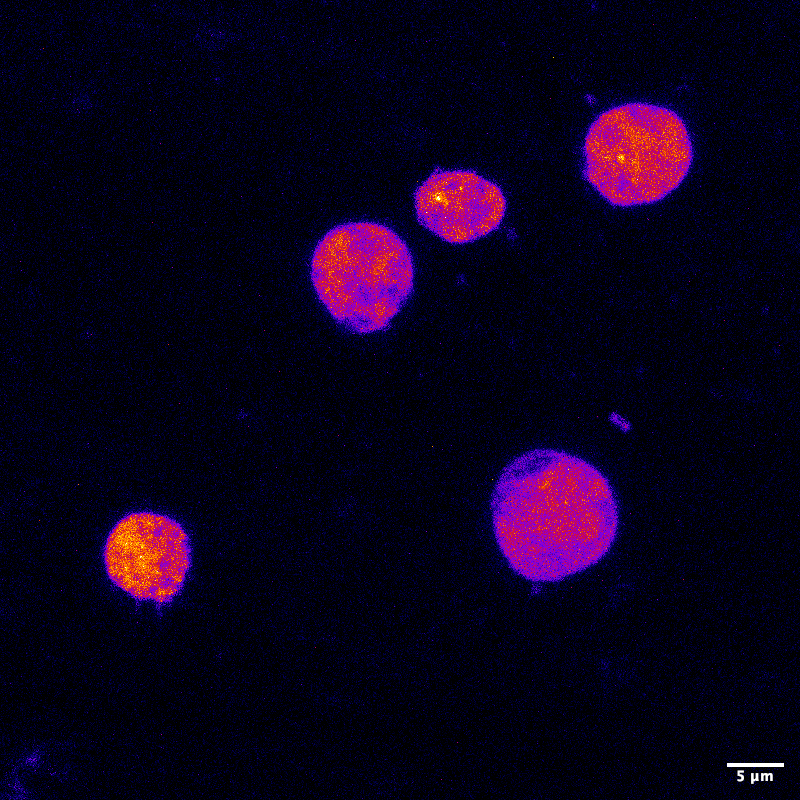

Supplement: Supplementary file 9 — Figure EV1 Source Data [file 44319_2025_574_MOESM9_ESM.zip › Fig. EV1/Fig. EV1_c-c''/Ct.HSD_ROS.tif]

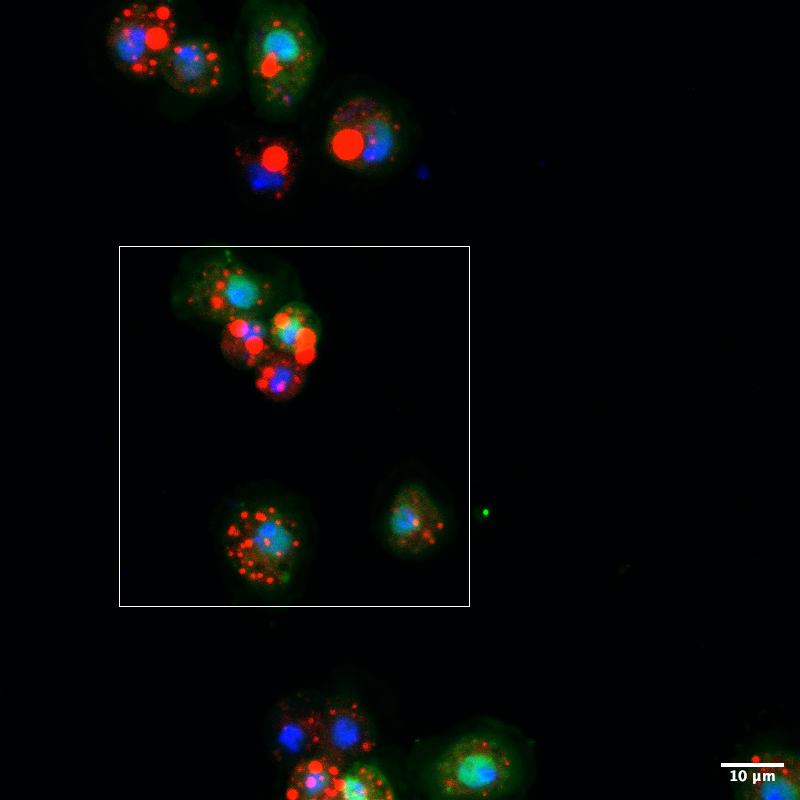

Supplement: Supplementary file 9 — Figure EV1 Source Data [file 44319_2025_574_MOESM9_ESM.zip › Fig. EV1/Fig. EV1_d-d''/4hr.HSD_lipids.tif]

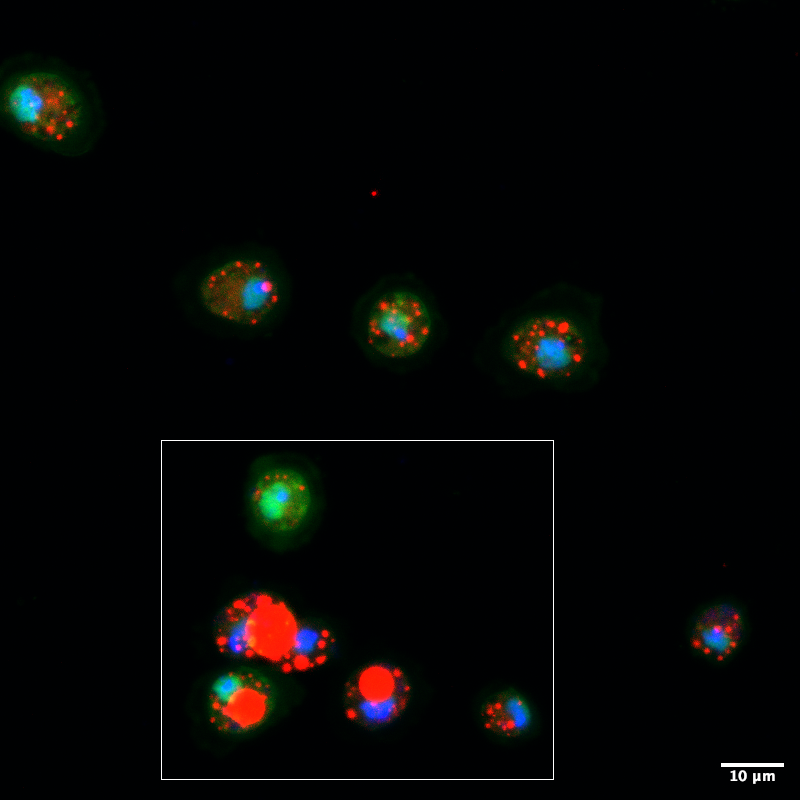

Supplement: Supplementary file 9 — Figure EV1 Source Data [file 44319_2025_574_MOESM9_ESM.zip › Fig. EV1/Fig. EV1_d-d''/Ct.HSD_lipids.tif]

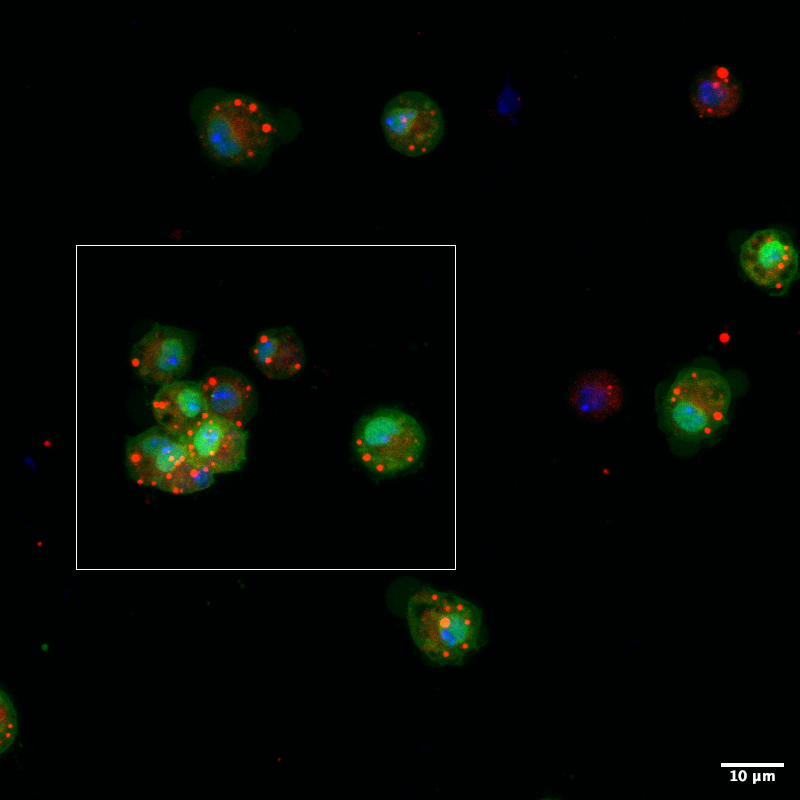

Supplement: Supplementary file 9 — Figure EV1 Source Data [file 44319_2025_574_MOESM9_ESM.zip › Fig. EV1/Fig. EV1_d-d''/RF_lipids.tif]

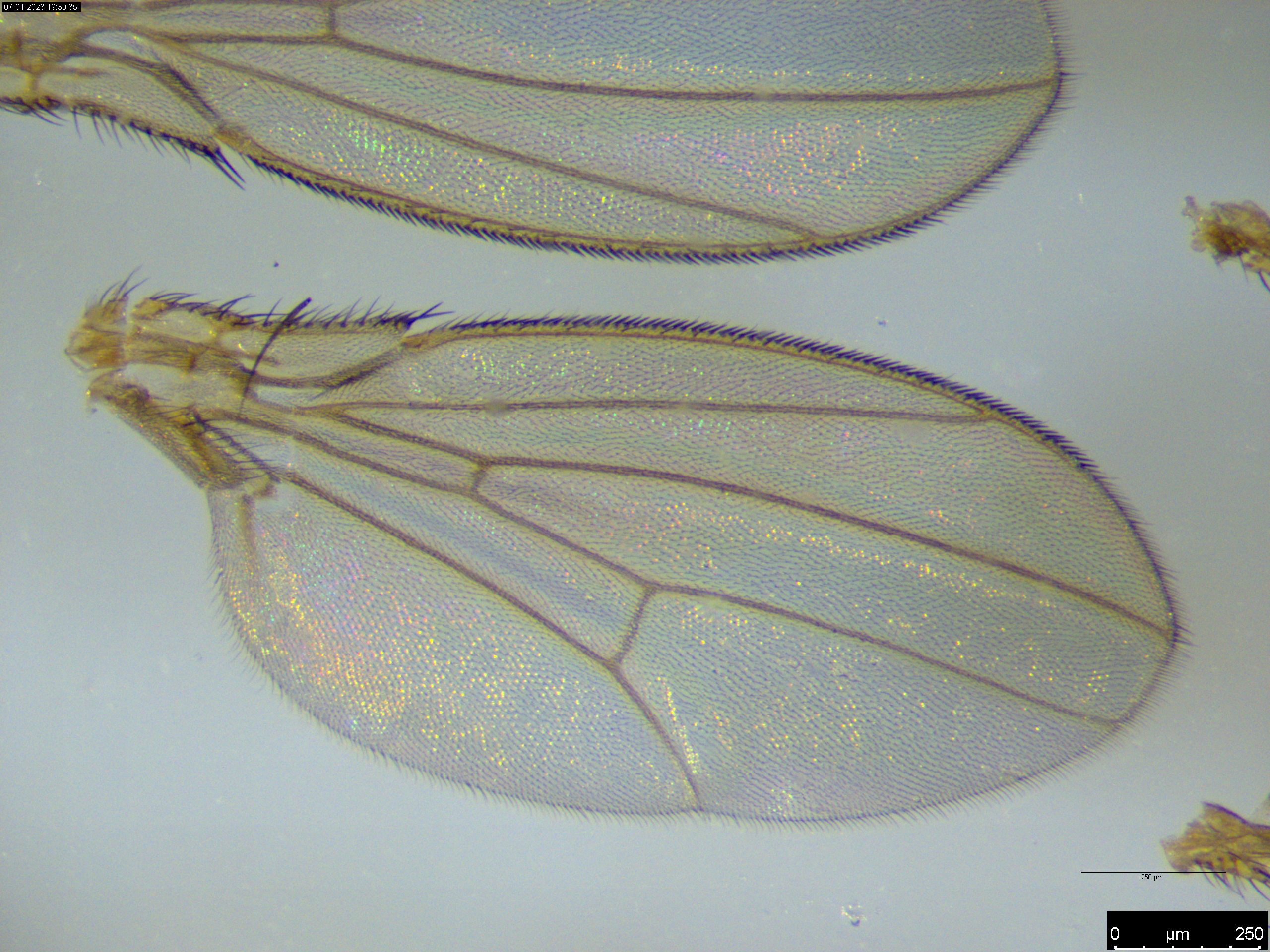

Supplement: Supplementary file 11 — Figure EV3 Source Data [file 44319_2025_574_MOESM11_ESM.zip › Fig. EV3/Fig. EV3_a-d/LdhRNAi_wing.tif]

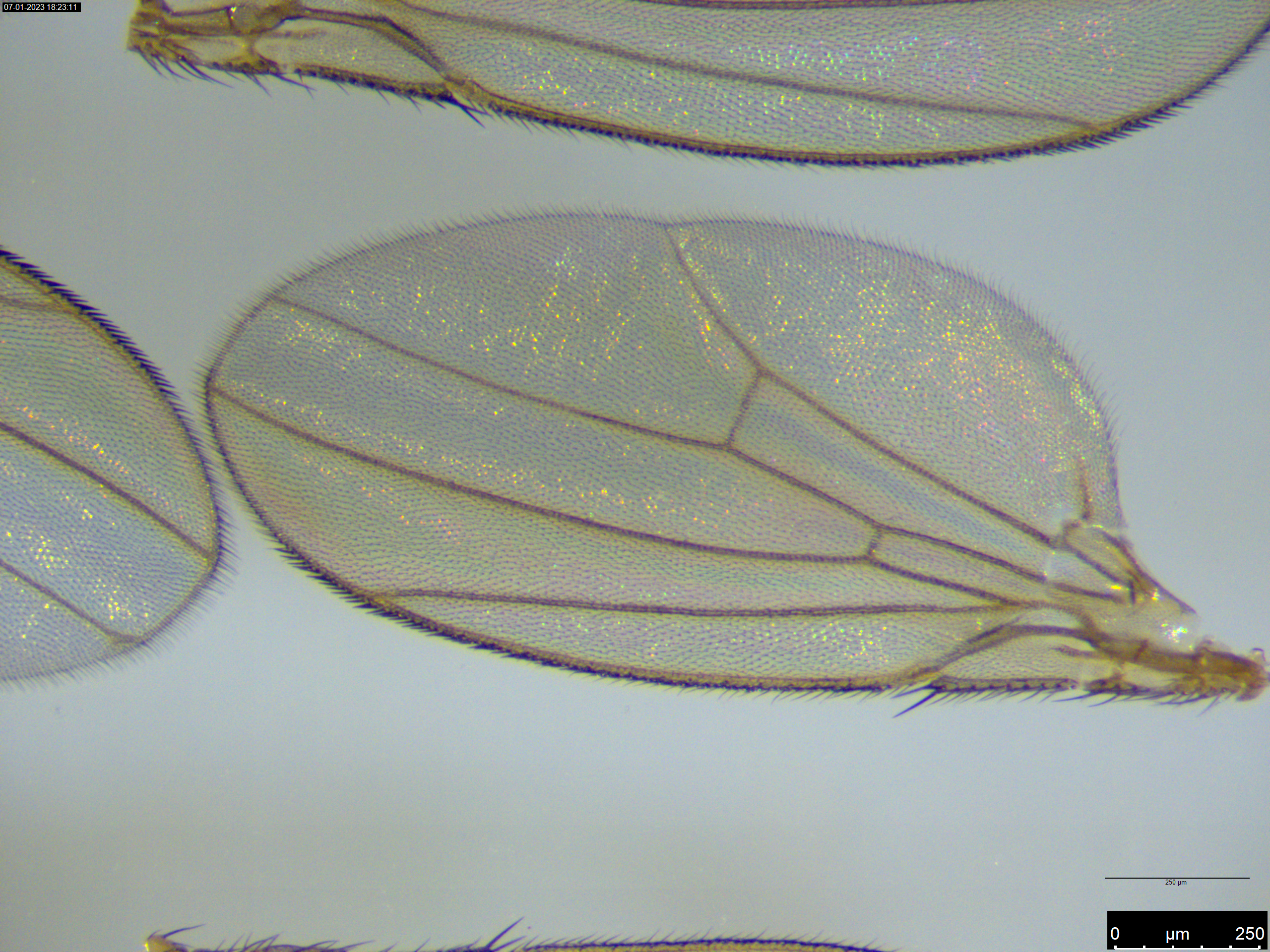

Supplement: Supplementary file 11 — Figure EV3 Source Data [file 44319_2025_574_MOESM11_ESM.zip › Fig. EV3/Fig. EV3_a-d/Control_wing.tif]

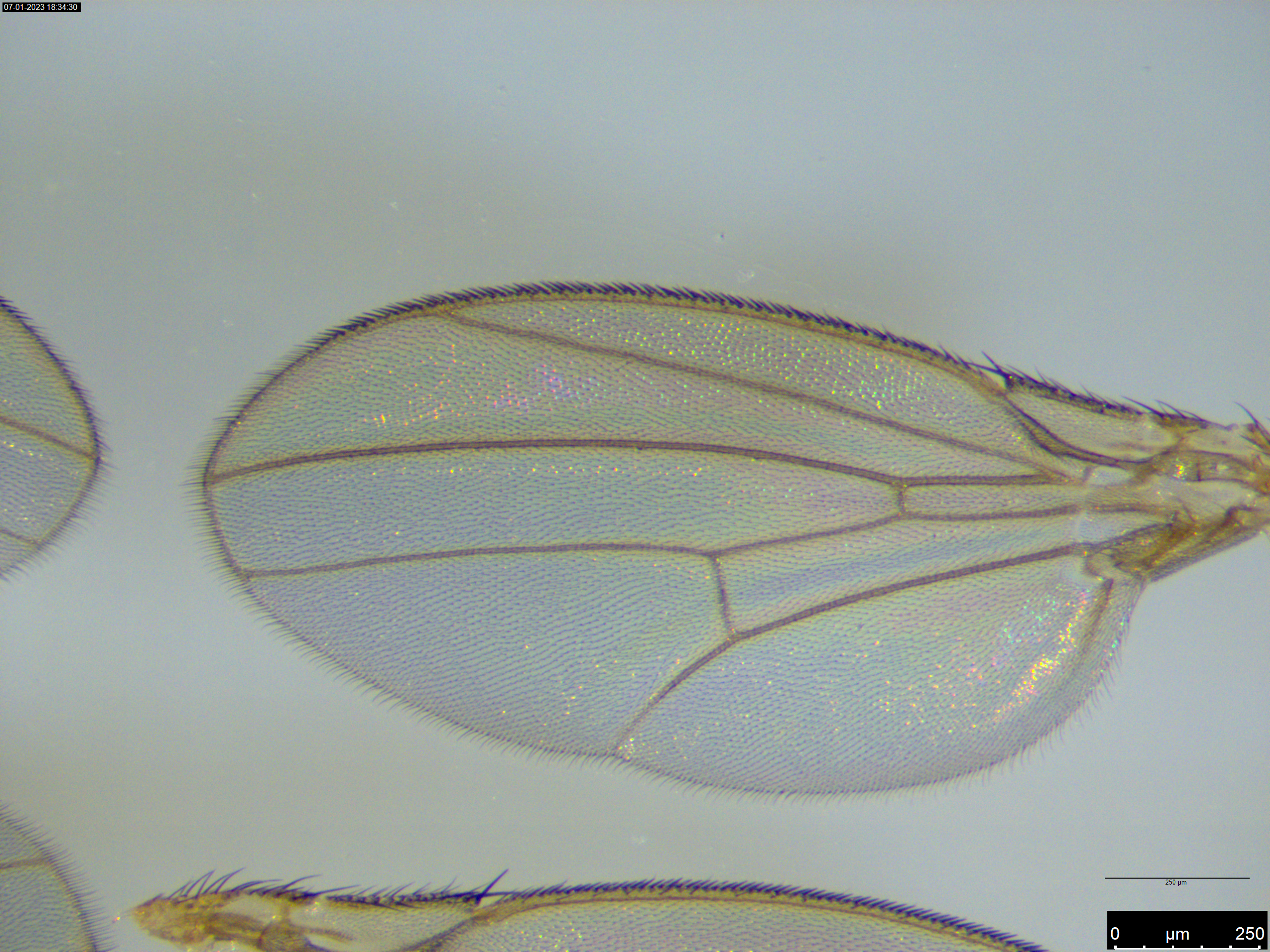

Supplement: Supplementary file 11 — Figure EV3 Source Data [file 44319_2025_574_MOESM11_ESM.zip › Fig. EV3/Fig. EV3_a-d/PdhaRNAi_wing.tif]

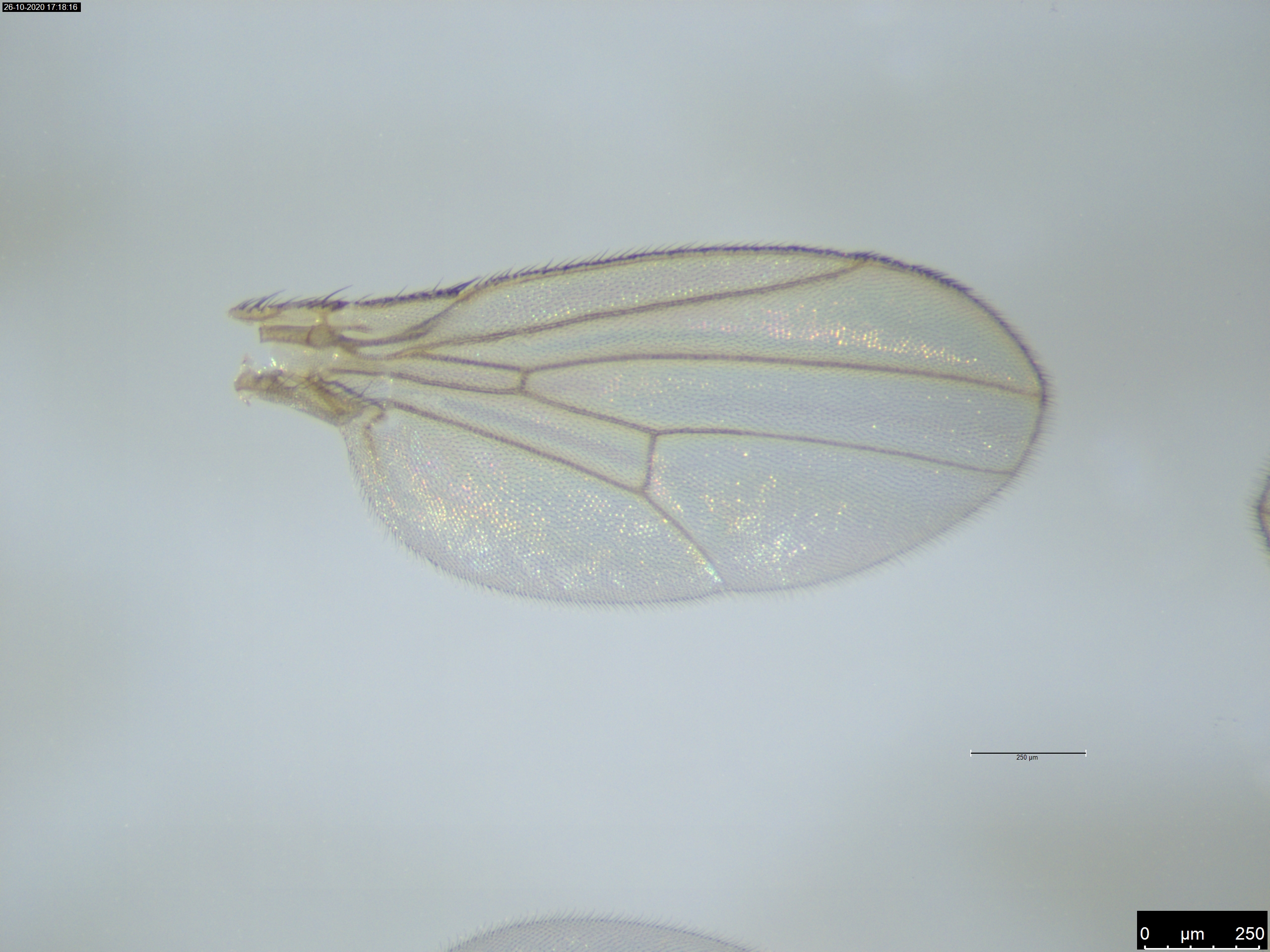

Supplement: Supplementary file 11 — Figure EV3 Source Data [file 44319_2025_574_MOESM11_ESM.zip › Fig. EV3/Fig. EV3_a-d/UAS-Ldh_wing.jpg]

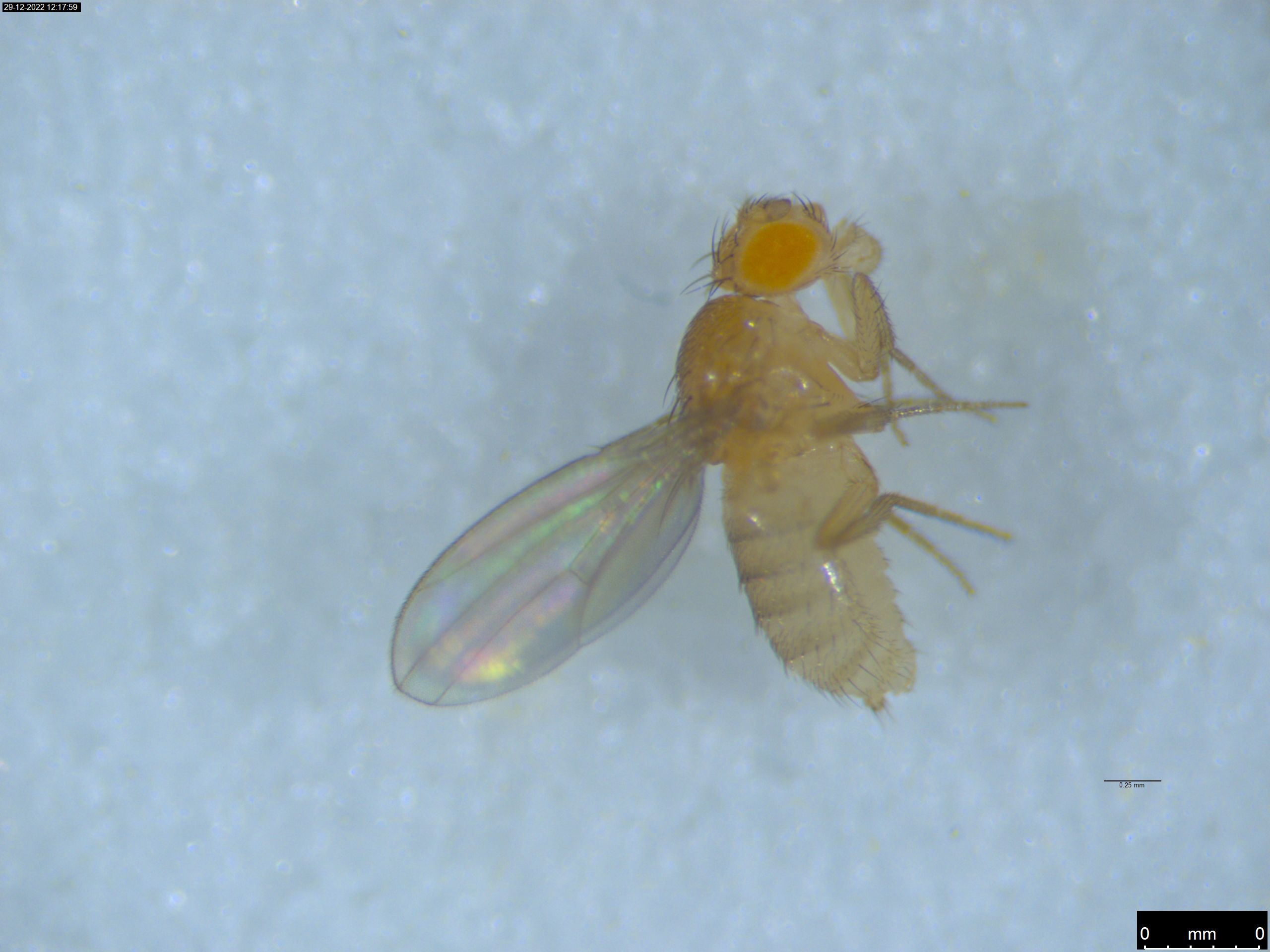

Supplement: Supplementary file 11 — Figure EV3 Source Data [file 44319_2025_574_MOESM11_ESM.zip › Fig. EV3/Fig. EV3_a'-d'/Control_fly.tif]

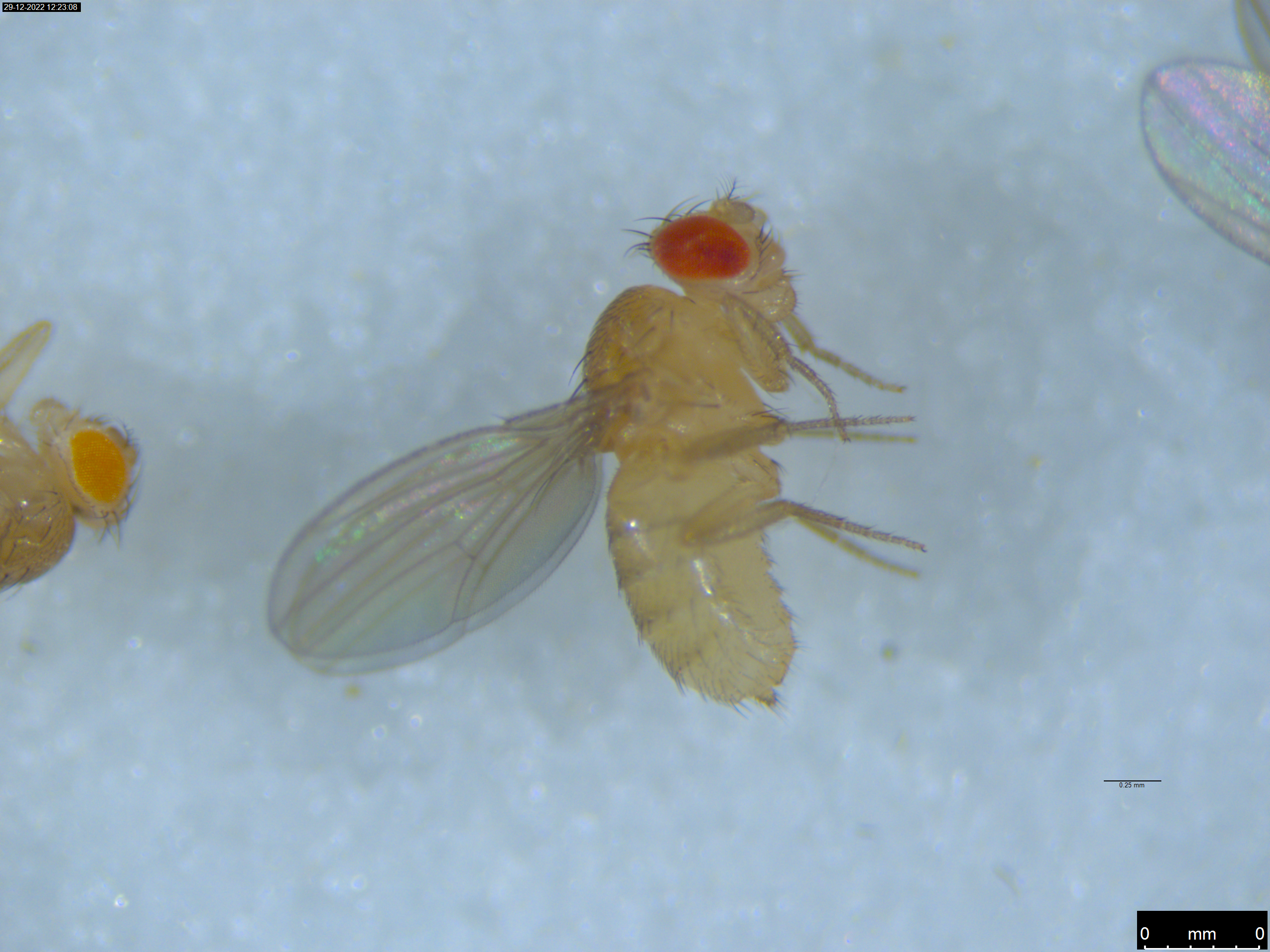

Supplement: Supplementary file 11 — Figure EV3 Source Data [file 44319_2025_574_MOESM11_ESM.zip › Fig. EV3/Fig. EV3_a'-d'/PdhaRNAi_fly.tif]

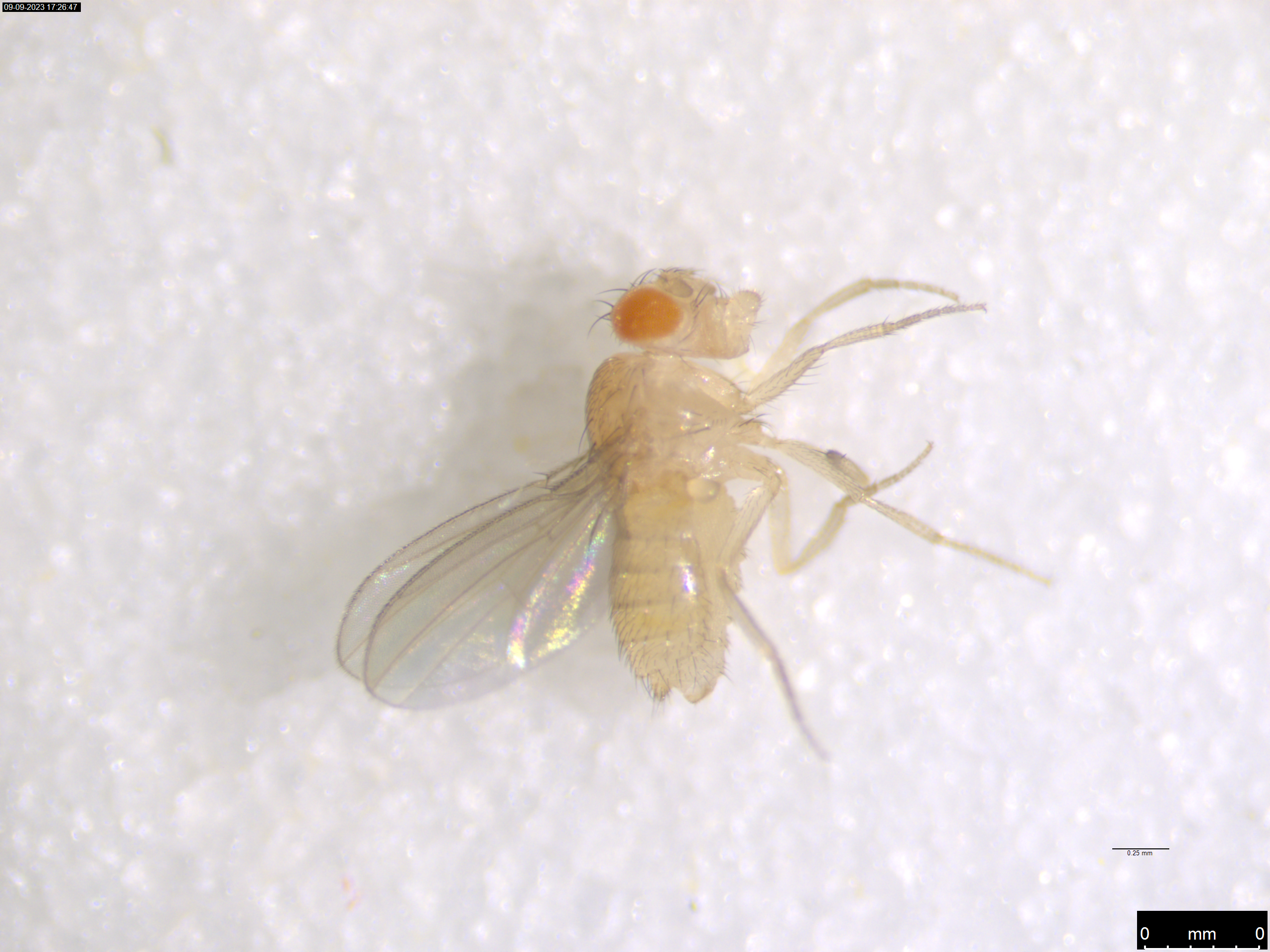

Supplement: Supplementary file 11 — Figure EV3 Source Data [file 44319_2025_574_MOESM11_ESM.zip › Fig. EV3/Fig. EV3_a'-d'/UAS-Ldh_fly.tif]
