# Supplementary figures and images for: Macrophage metabolic reprogramming during dietary stress influences adult body size in Drosophila (part 2 of 2)
Source: EMBO Rep. 2025 Sep 9;26(22):5397–430. doi: 10.1038/s44319-025-00574-7 (PMC12635341; doi:10.1038/s44319-025-00574-7)

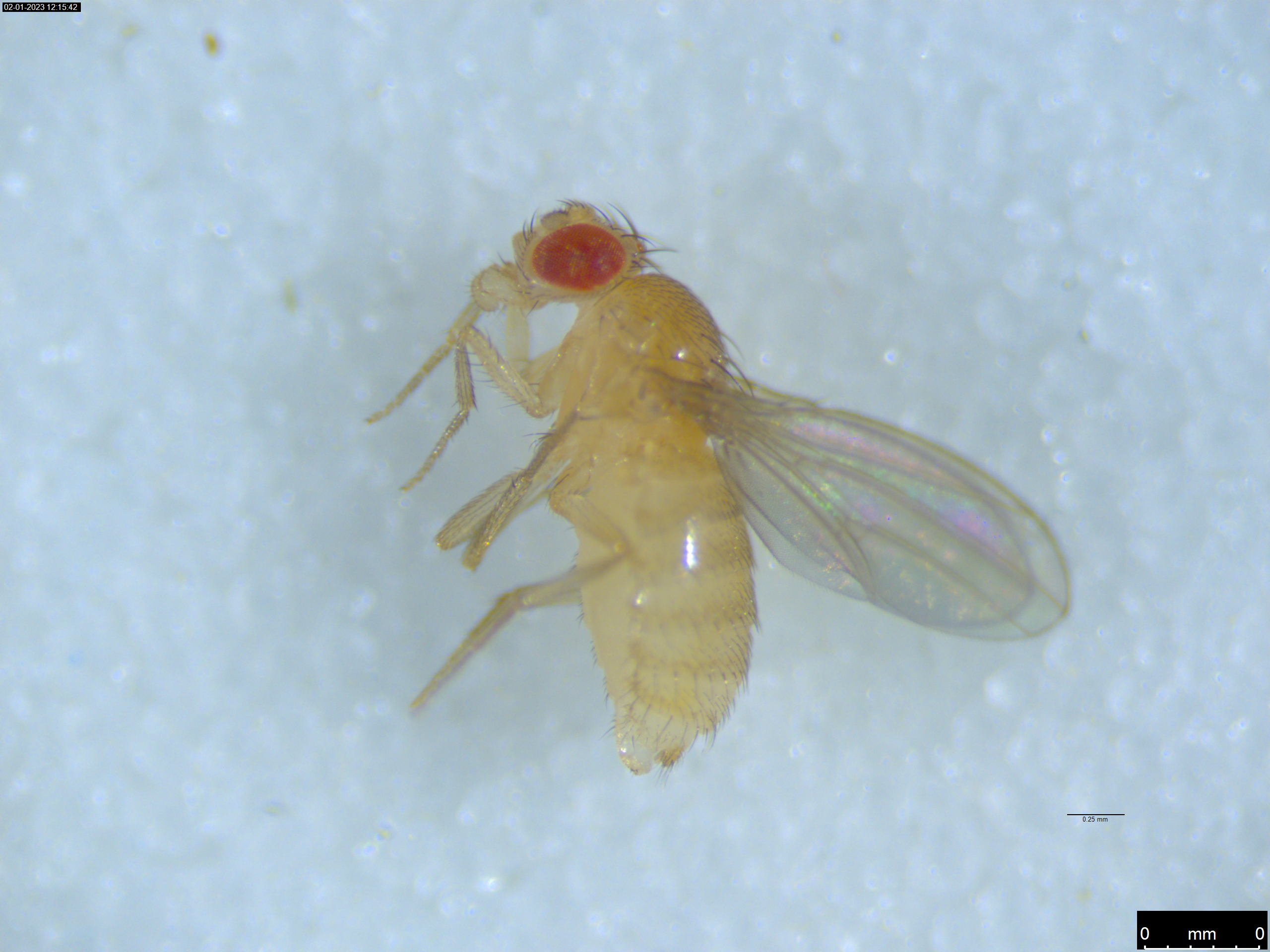

Supplement: Supplementary file 11 — Figure EV3 Source Data [file 44319_2025_574_MOESM11_ESM.zip › Fig. EV3/Fig. EV3_a'-d'/LdhRNAi_fly.tif]

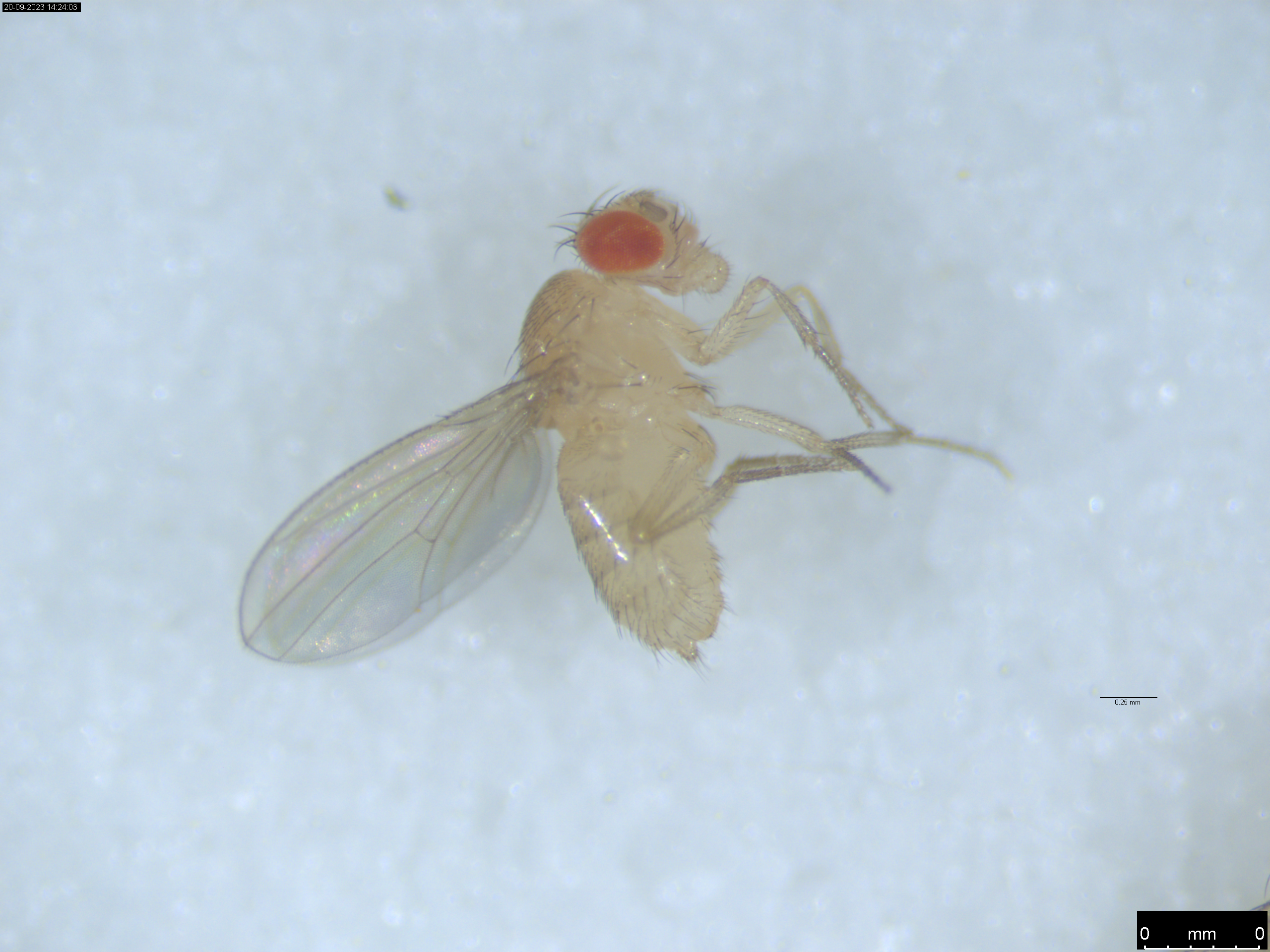

Supplement: Supplementary file 12 — Figure EV4 Source Data [file 44319_2025_574_MOESM12_ESM.zip › Fig. EV4/Fig. EV4_g'-k'/ACCRNAi_fly.tif]

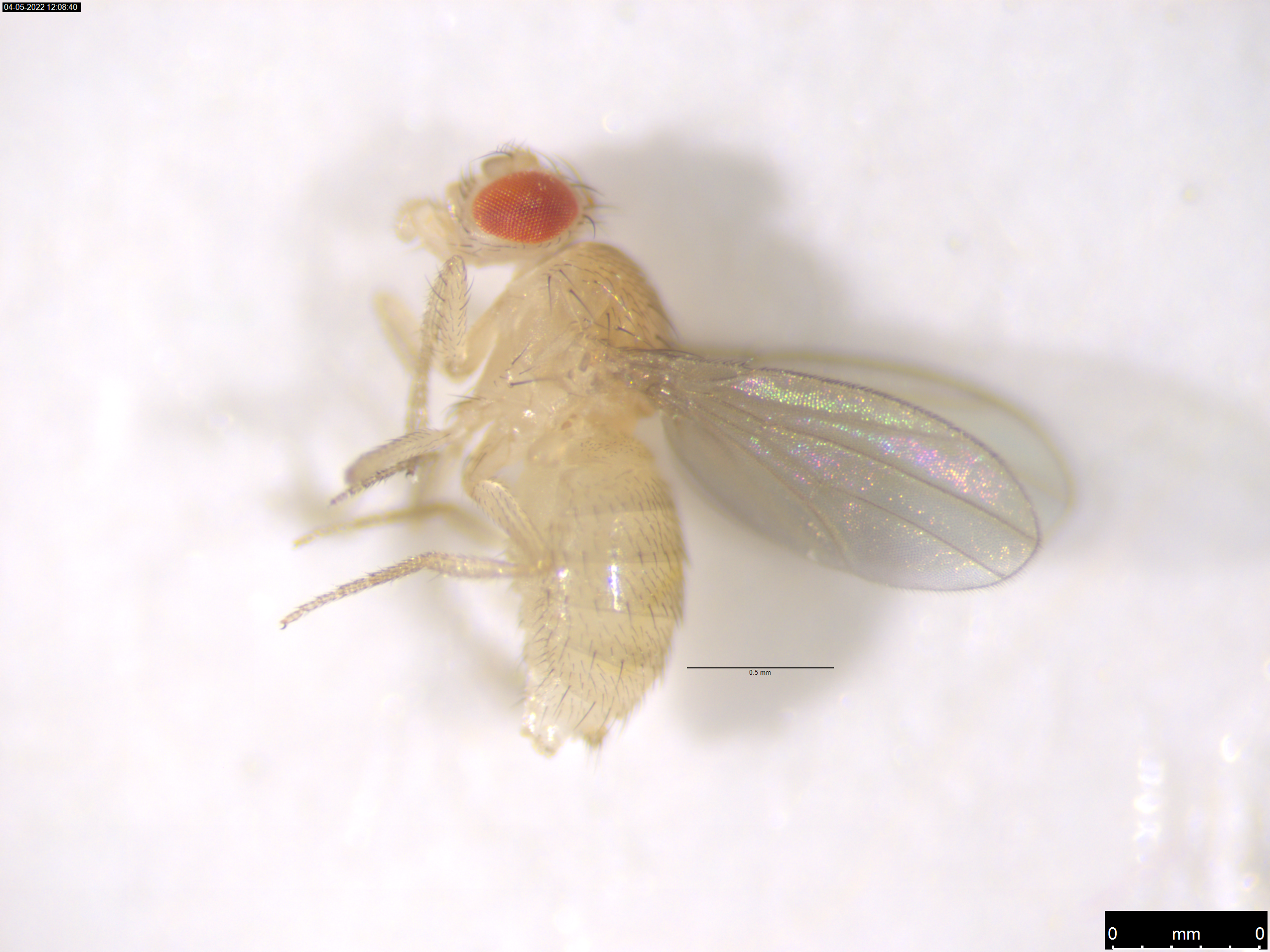

Supplement: Supplementary file 12 — Figure EV4 Source Data [file 44319_2025_574_MOESM12_ESM.zip › Fig. EV4/Fig. EV4_g'-k'/Agpat3RNAi_fly.tif]

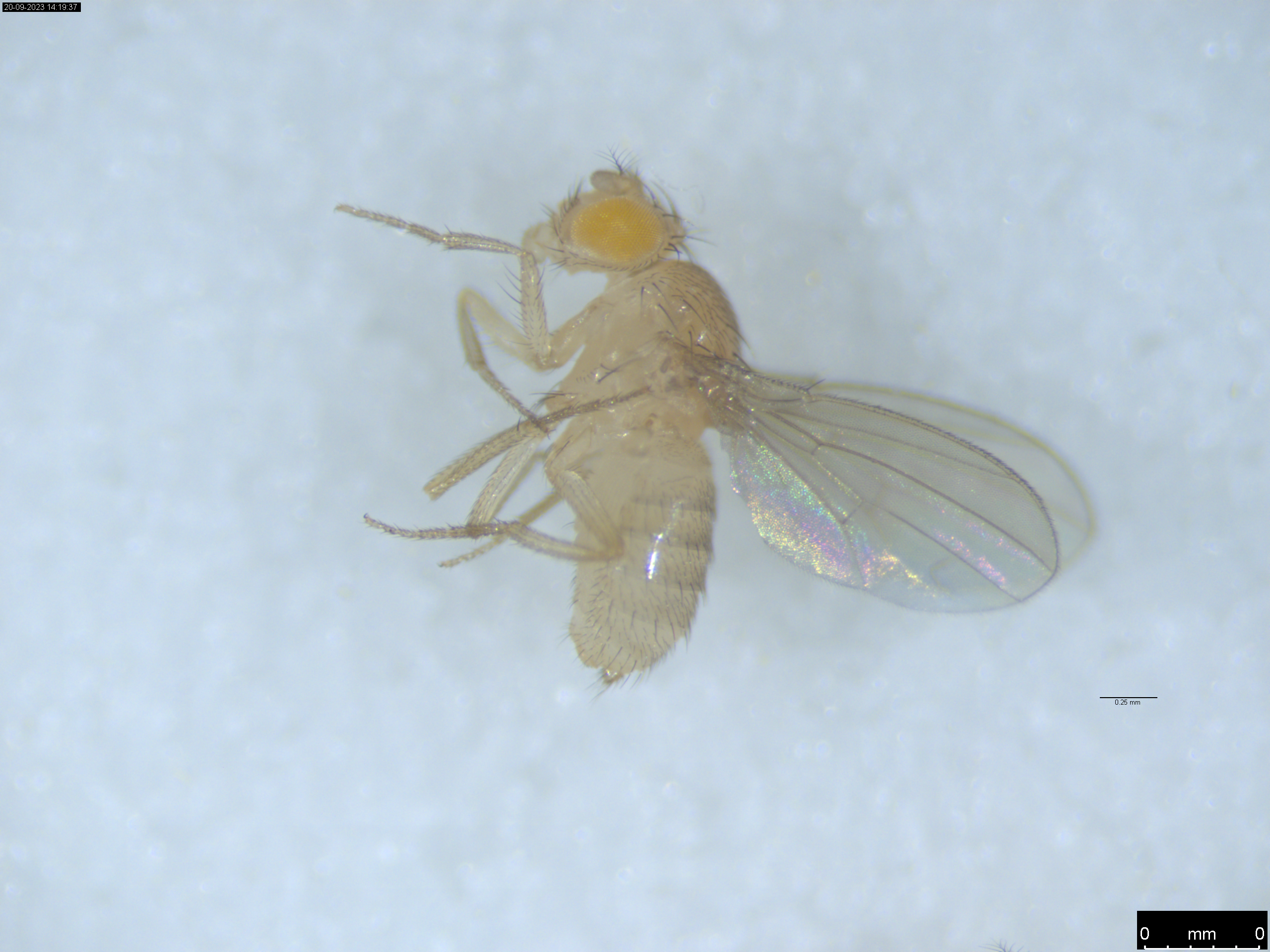

Supplement: Supplementary file 12 — Figure EV4 Source Data [file 44319_2025_574_MOESM12_ESM.zip › Fig. EV4/Fig. EV4_g'-k'/Control_fly.tif]

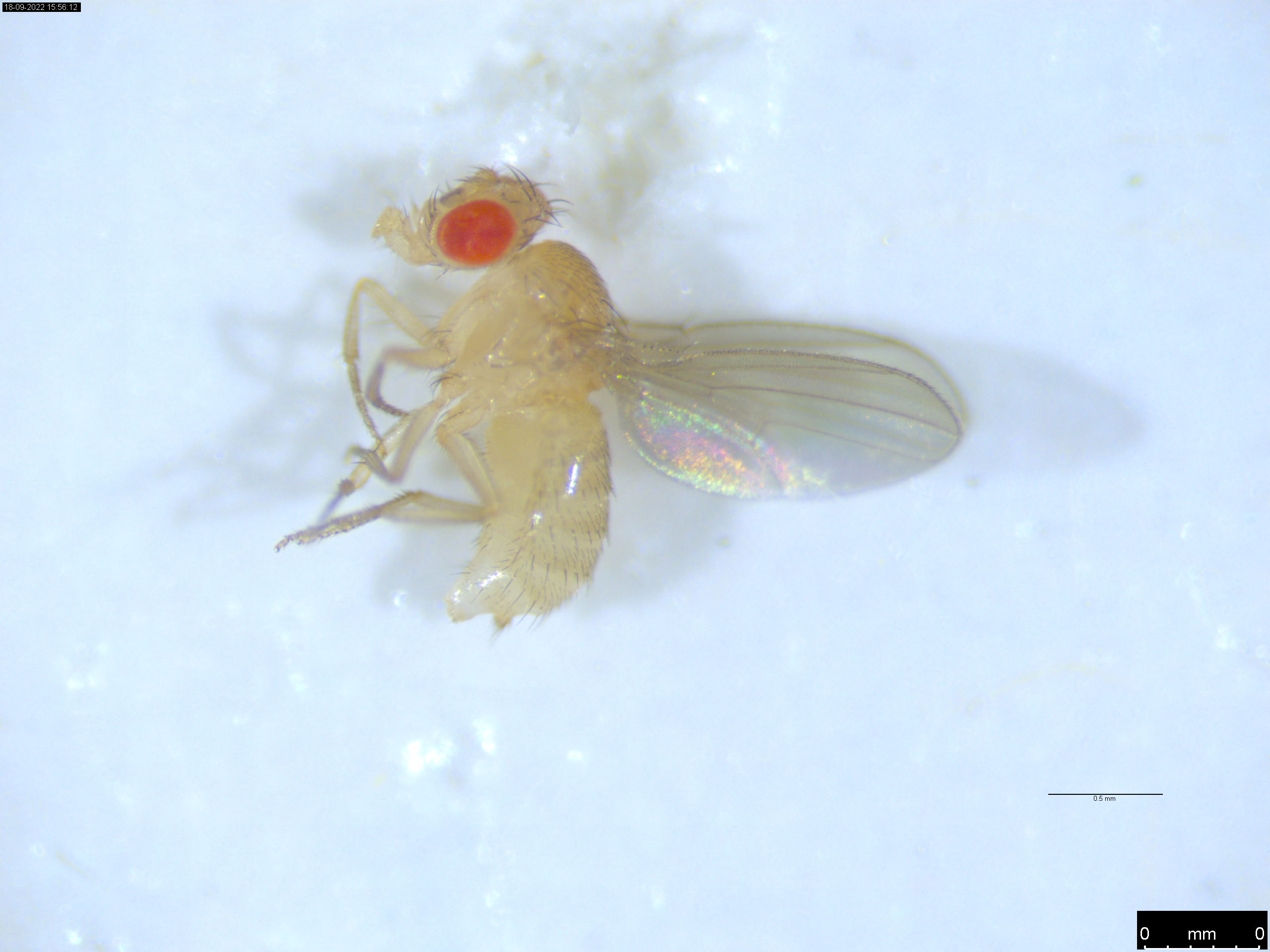

Supplement: Supplementary file 12 — Figure EV4 Source Data [file 44319_2025_574_MOESM12_ESM.zip › Fig. EV4/Fig. EV4_g'-k'/Gpat4RNAi_fly.tif]

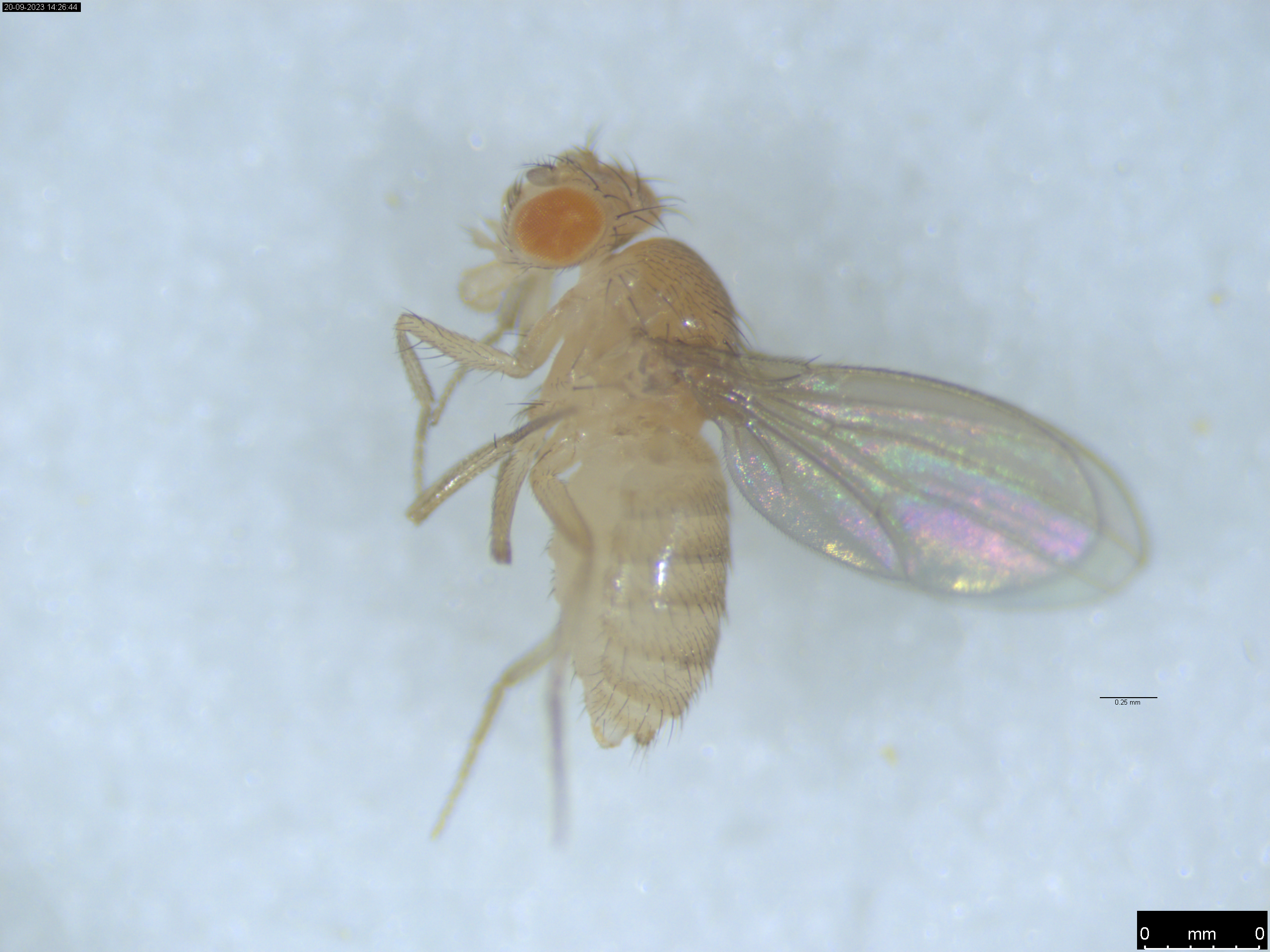

Supplement: Supplementary file 12 — Figure EV4 Source Data [file 44319_2025_574_MOESM12_ESM.zip › Fig. EV4/Fig. EV4_g'-k'/UAS-ACC_fly.tif]

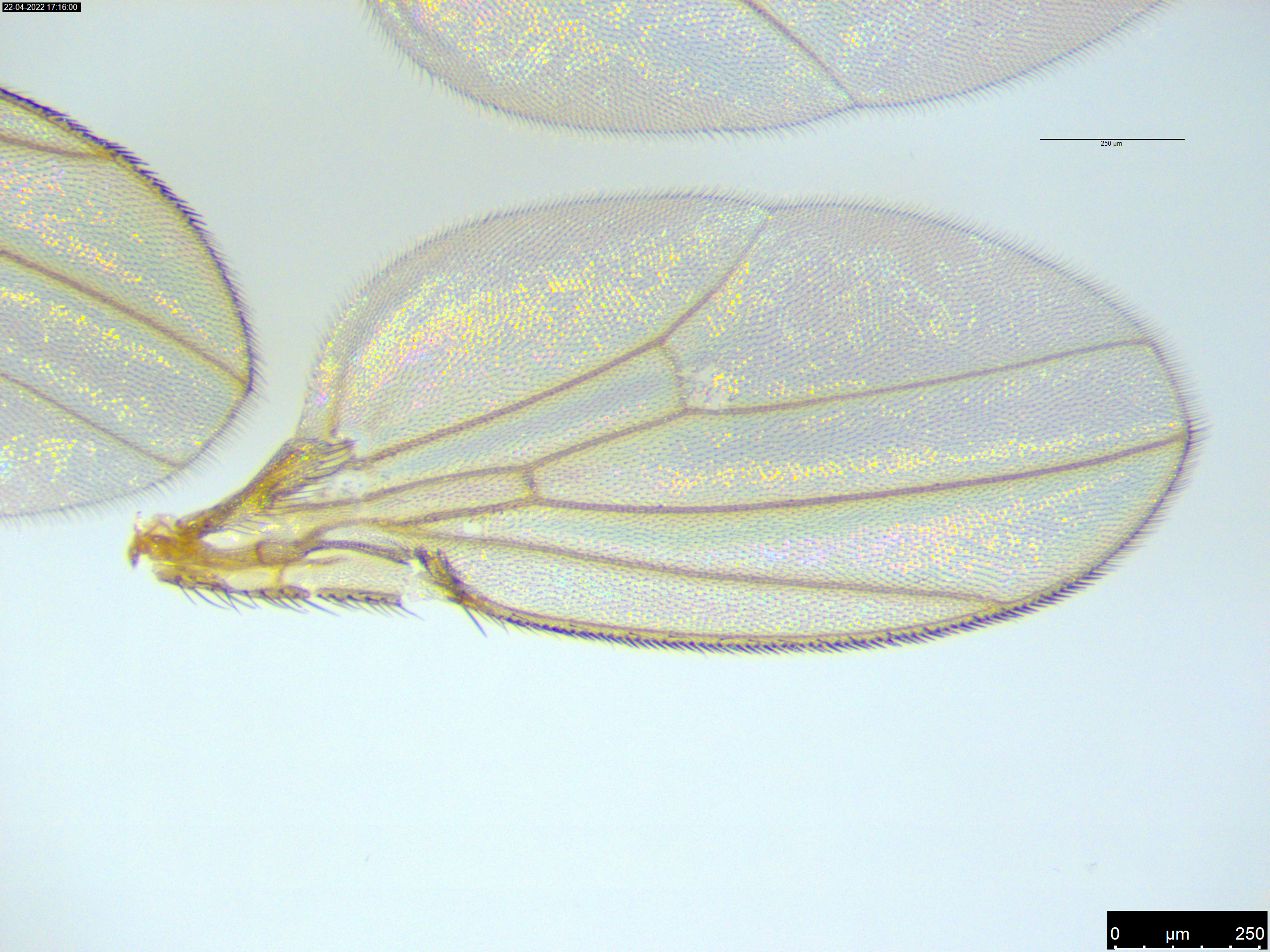

Supplement: Supplementary file 12 — Figure EV4 Source Data [file 44319_2025_574_MOESM12_ESM.zip › Fig. EV4/Fig. EV4_g-k/ACCRNAi_fly.tif]

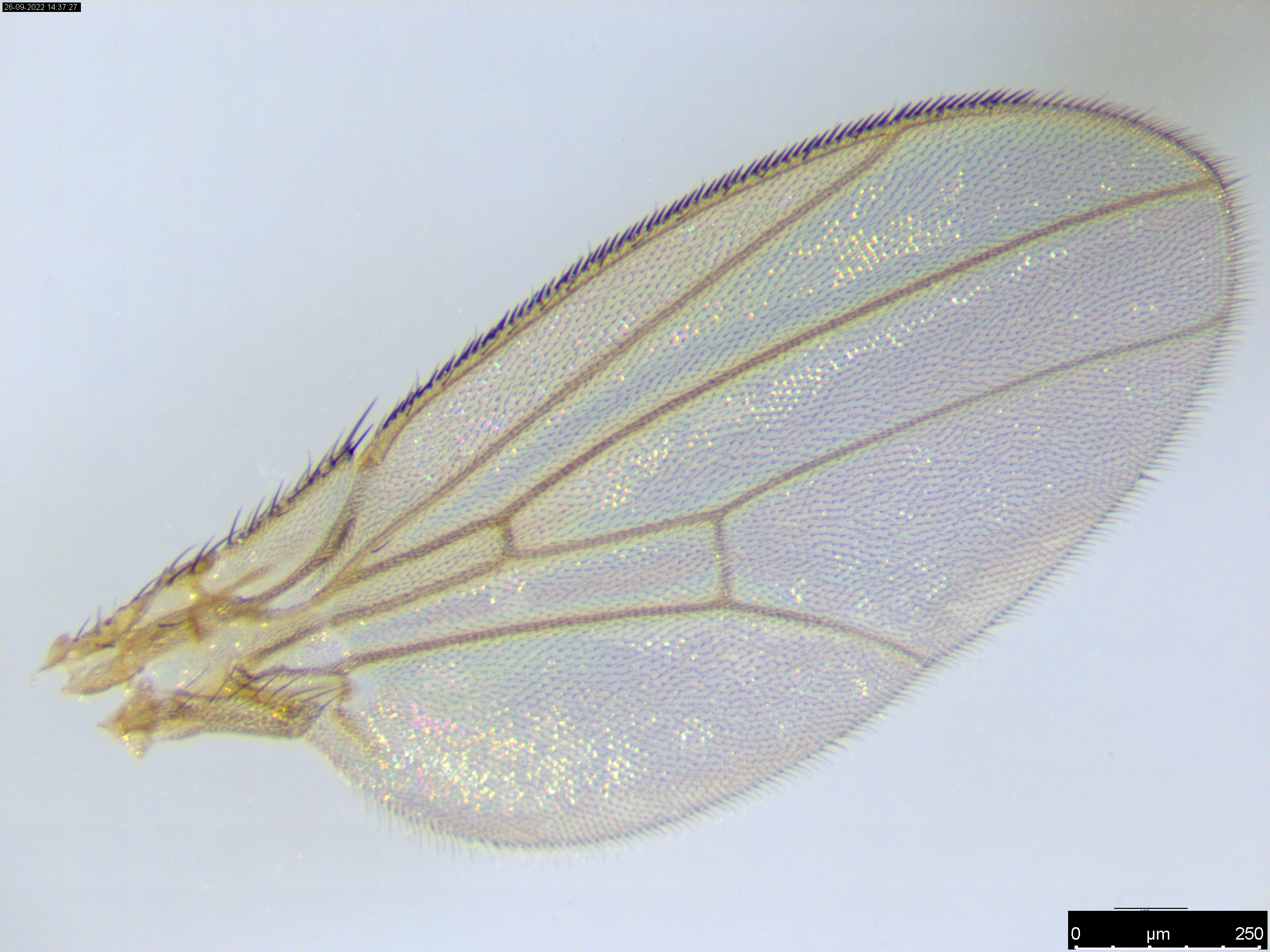

Supplement: Supplementary file 12 — Figure EV4 Source Data [file 44319_2025_574_MOESM12_ESM.zip › Fig. EV4/Fig. EV4_g-k/Agpat3RNAi_fly.tif]

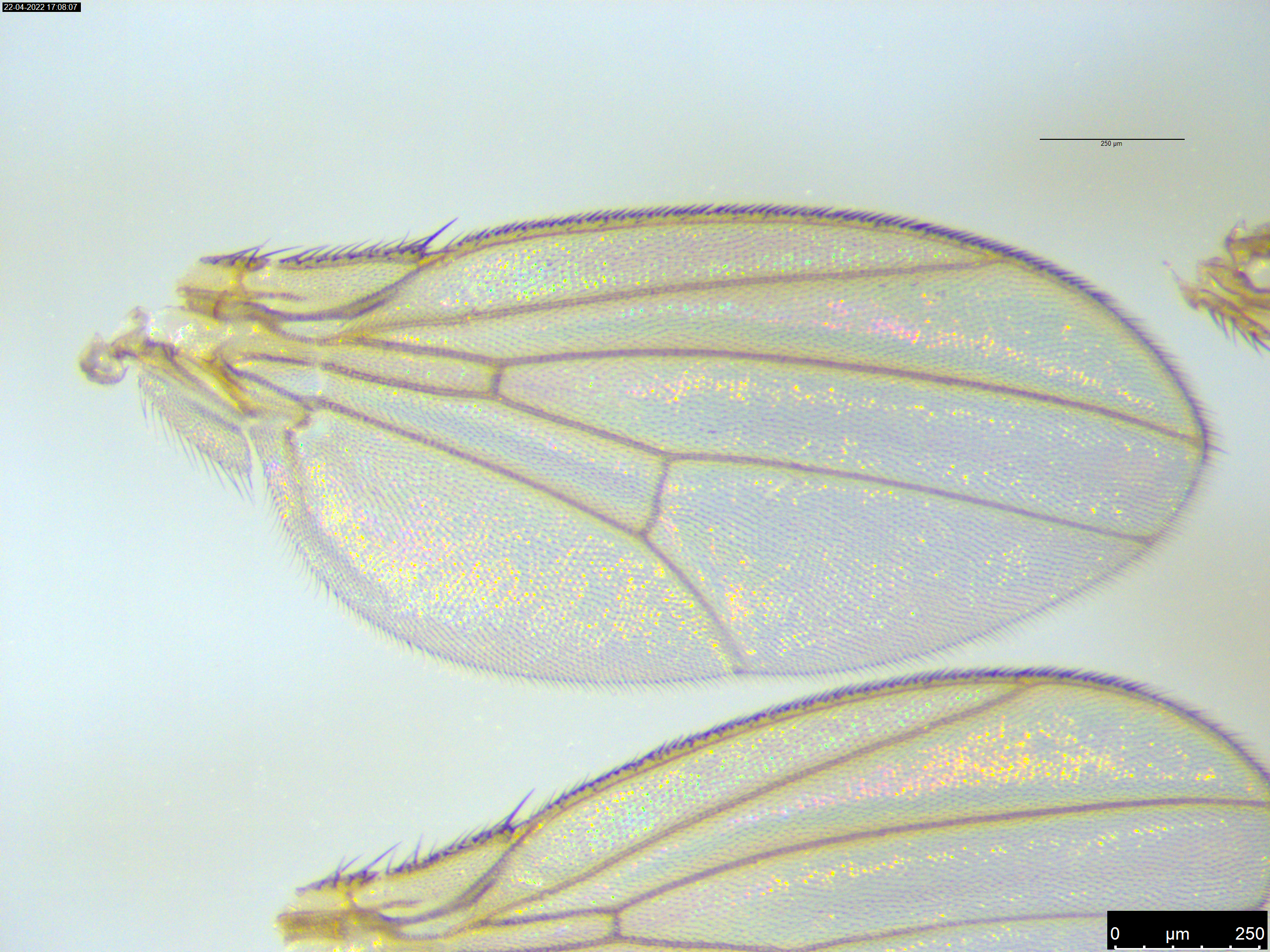

Supplement: Supplementary file 12 — Figure EV4 Source Data [file 44319_2025_574_MOESM12_ESM.zip › Fig. EV4/Fig. EV4_g-k/Control_fly.tif]

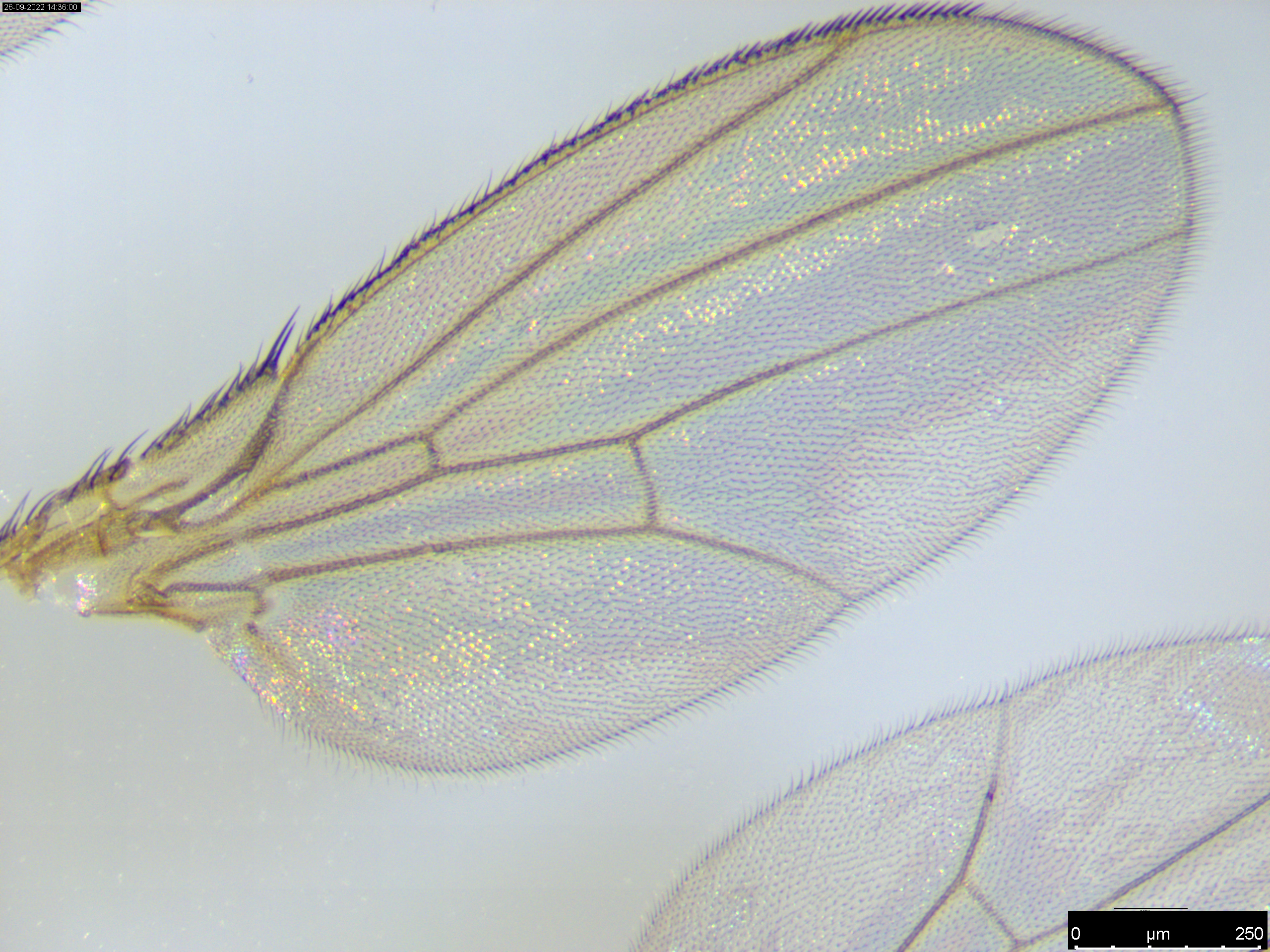

Supplement: Supplementary file 12 — Figure EV4 Source Data [file 44319_2025_574_MOESM12_ESM.zip › Fig. EV4/Fig. EV4_g-k/Gpat4RNAi_fly.tif]

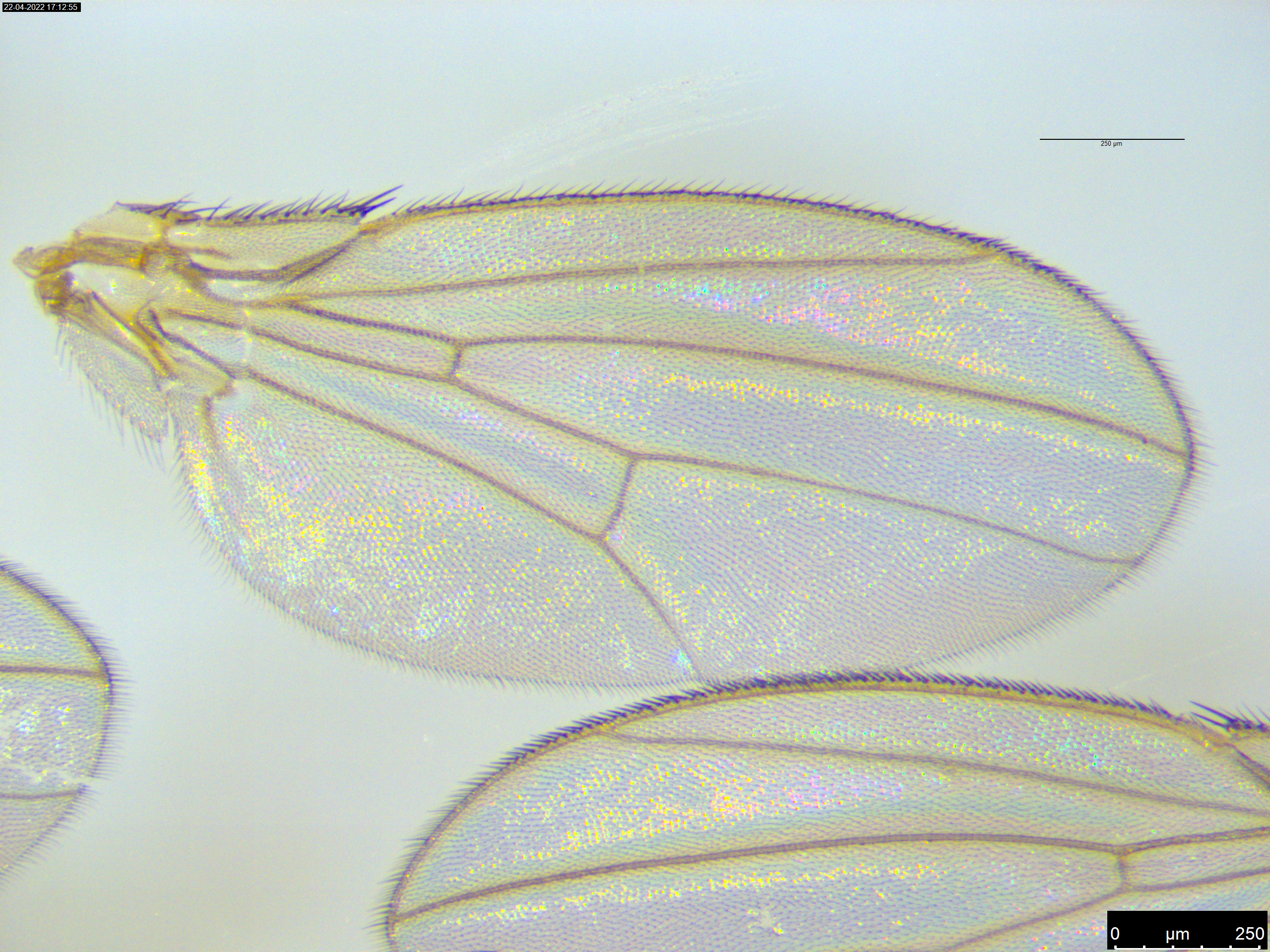

Supplement: Supplementary file 12 — Figure EV4 Source Data [file 44319_2025_574_MOESM12_ESM.zip › Fig. EV4/Fig. EV4_g-k/UAS-ACC_fly.tif]

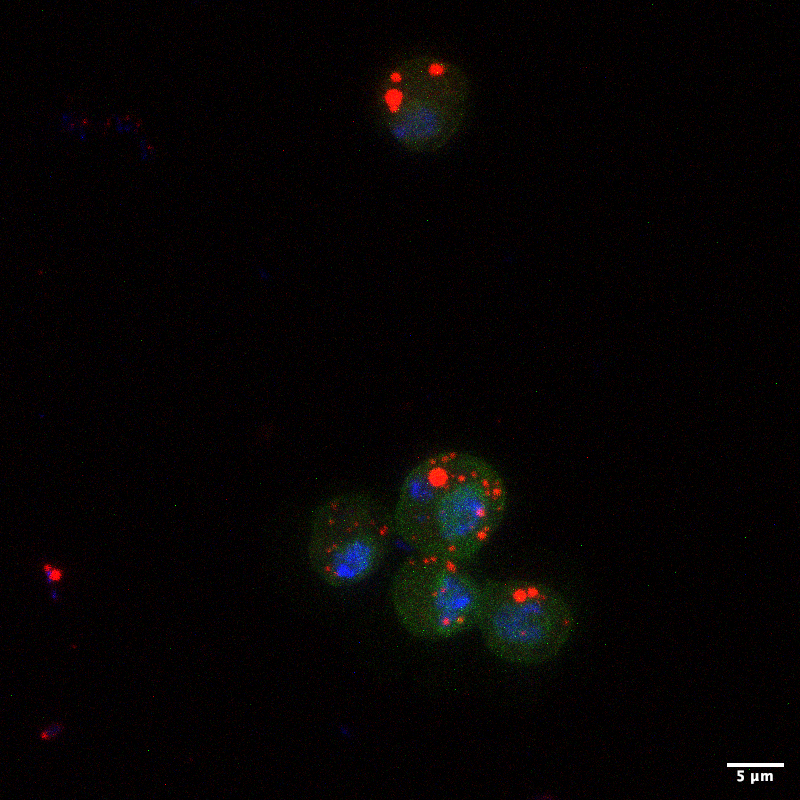

Supplement: Supplementary file 12 — Figure EV4 Source Data [file 44319_2025_574_MOESM12_ESM.zip › Fig. EV4/Fig. EV4_b-f/Agpat3RNAi_nile red.tif]

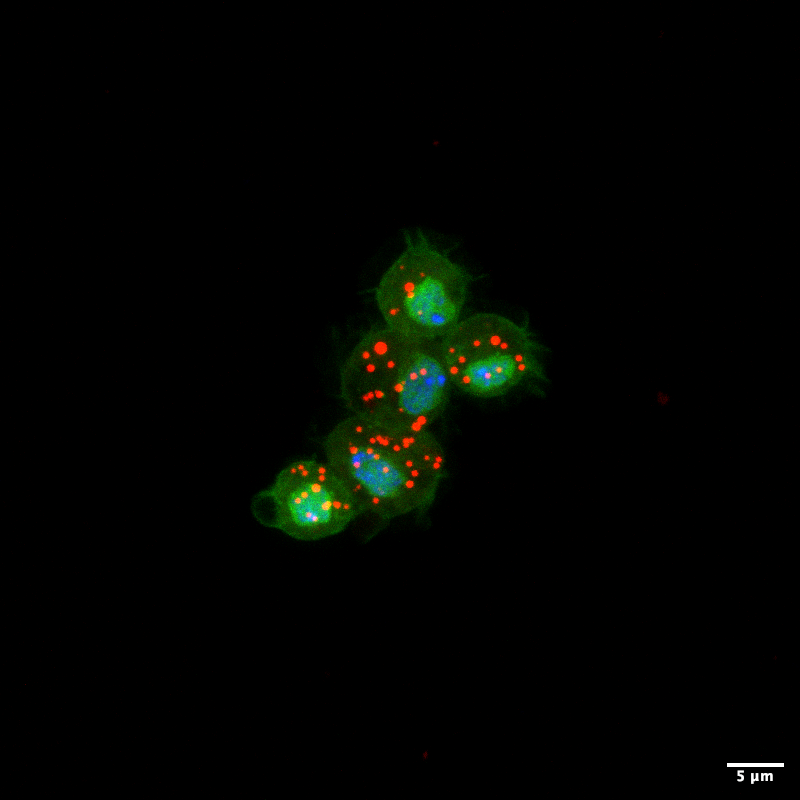

Supplement: Supplementary file 12 — Figure EV4 Source Data [file 44319_2025_574_MOESM12_ESM.zip › Fig. EV4/Fig. EV4_b-f/Control_nile red.tif]

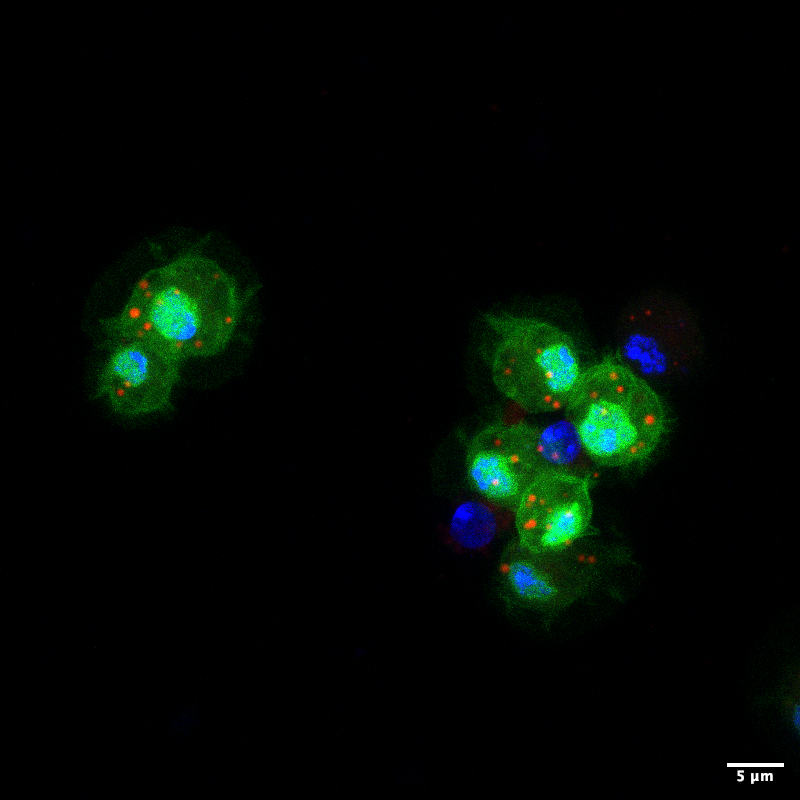

Supplement: Supplementary file 12 — Figure EV4 Source Data [file 44319_2025_574_MOESM12_ESM.zip › Fig. EV4/Fig. EV4_b-f/Gpat4RNAi_nile red.tif]

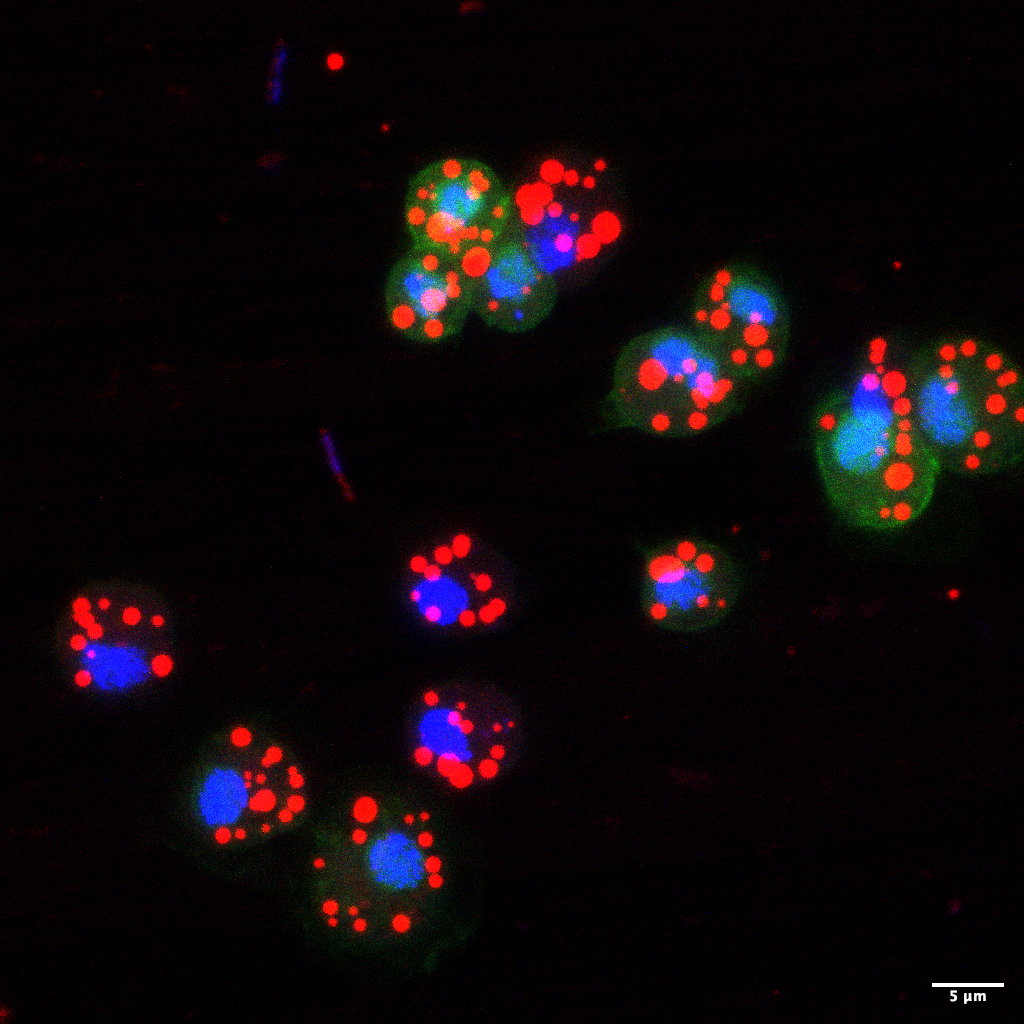

Supplement: Supplementary file 12 — Figure EV4 Source Data [file 44319_2025_574_MOESM12_ESM.zip › Fig. EV4/Fig. EV4_b-f/UAS-ACC_nile red.tif]

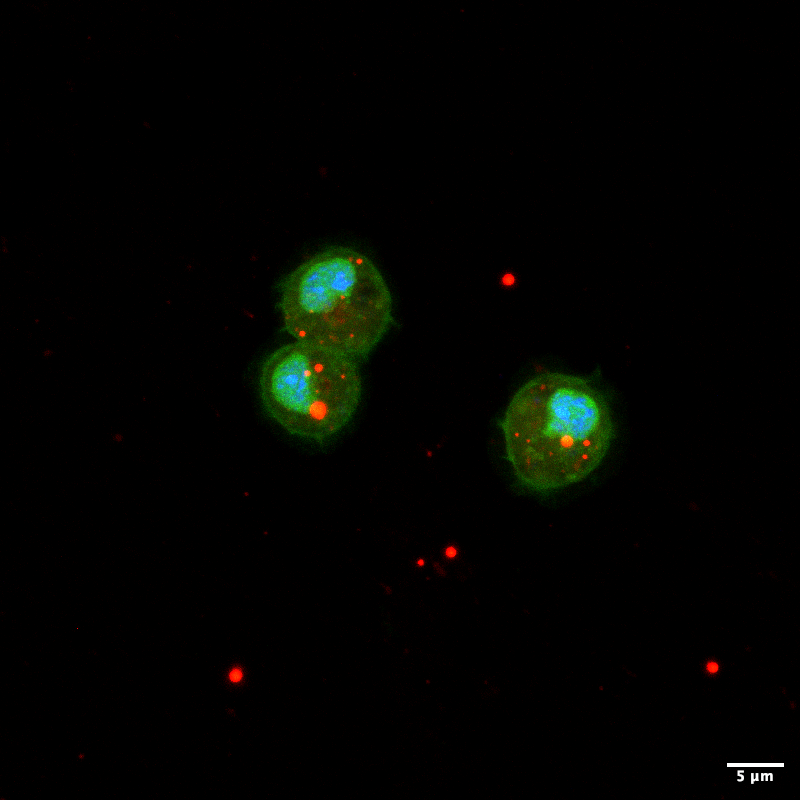

Supplement: Supplementary file 12 — Figure EV4 Source Data [file 44319_2025_574_MOESM12_ESM.zip › Fig. EV4/Fig. EV4_b-f/ACCRNAi_nile red.tif]

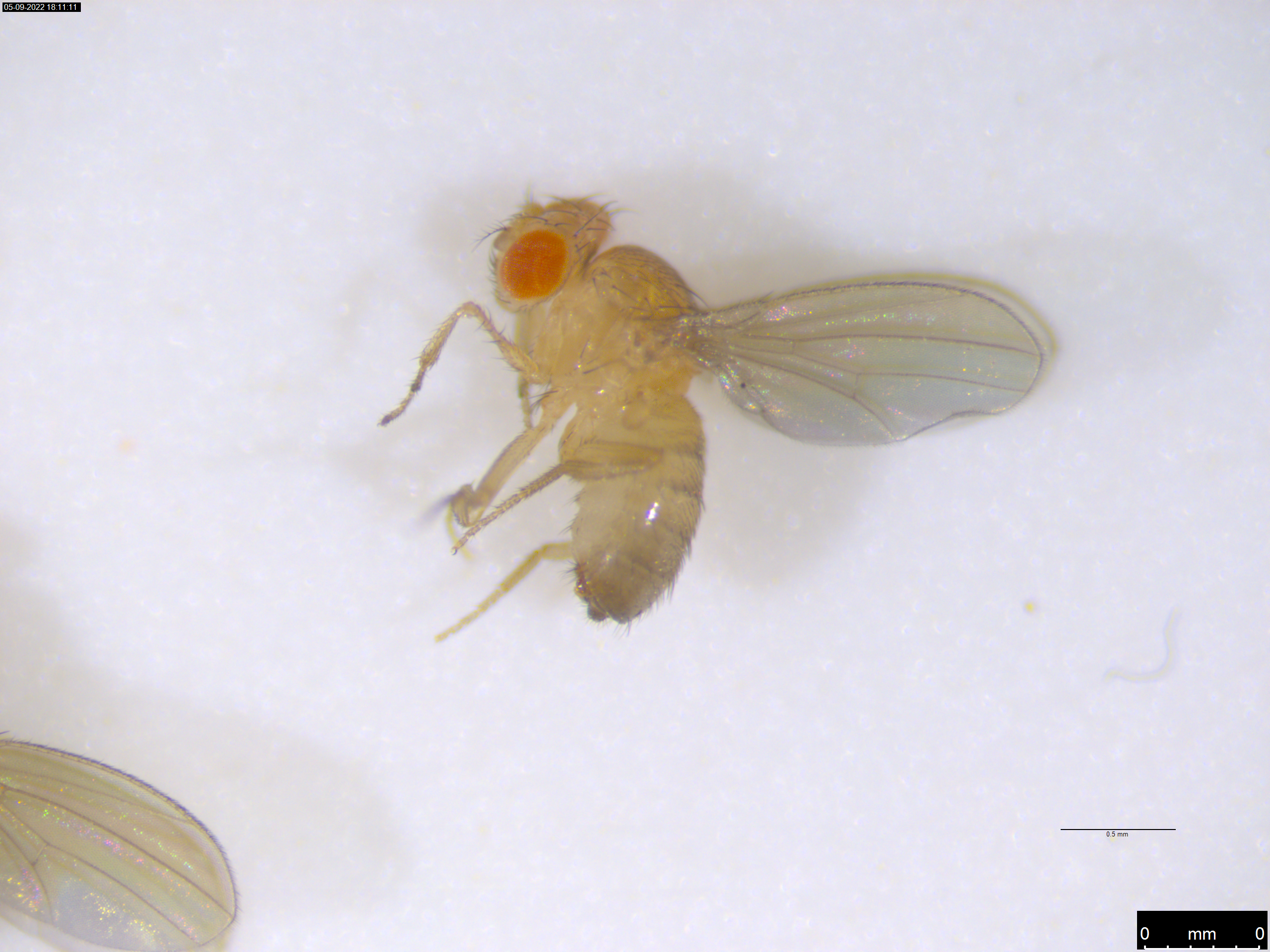

Supplement: Supplementary file 13 — Figure EV5 Source Data [file 44319_2025_574_MOESM13_ESM.zip › Fig. EV5/Fig. EV5_e'-g'/UAS-bmm_fly.tif]

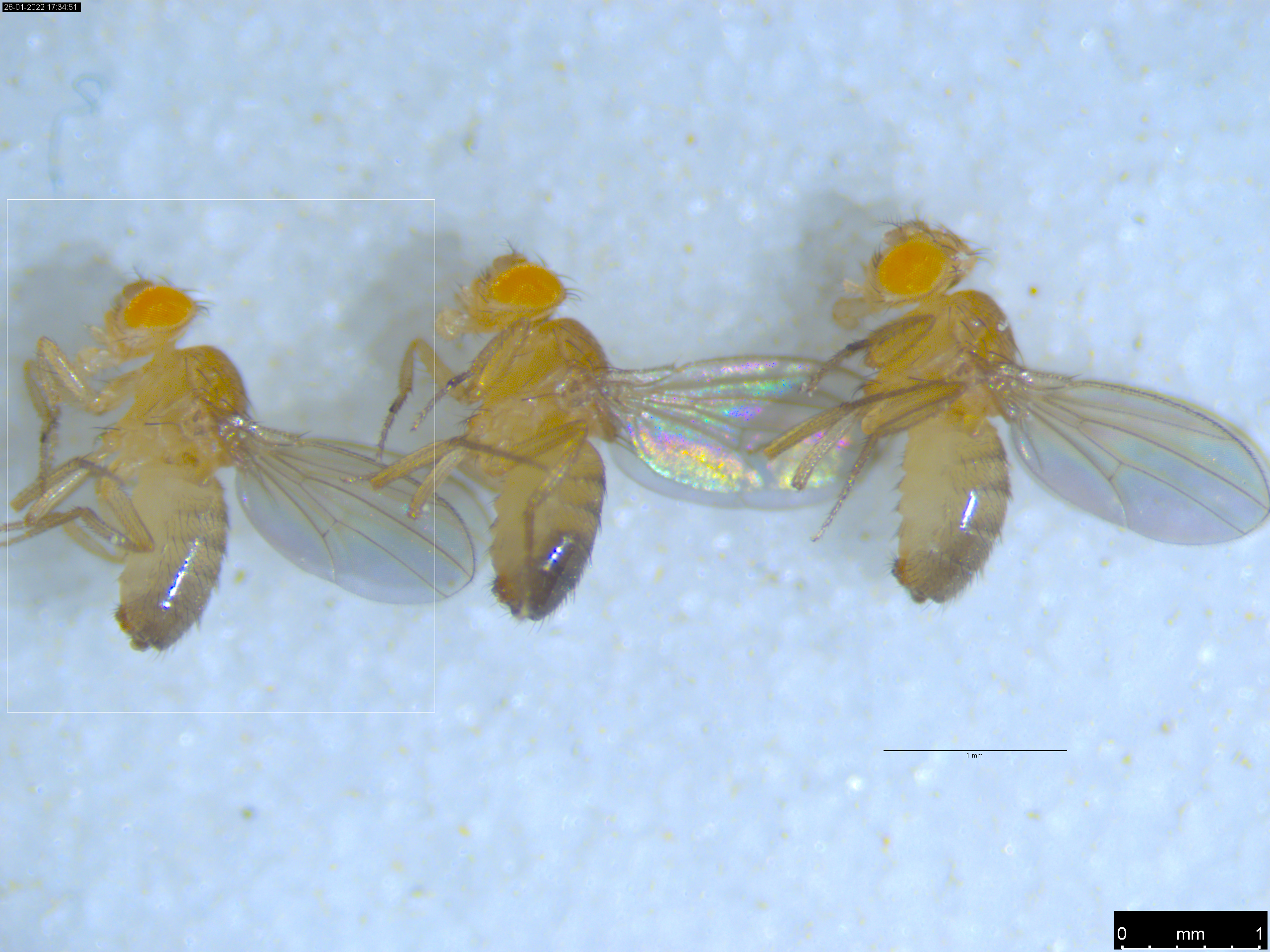

Supplement: Supplementary file 13 — Figure EV5 Source Data [file 44319_2025_574_MOESM13_ESM.zip › Fig. EV5/Fig. EV5_e'-g'/Control_fly.tif]

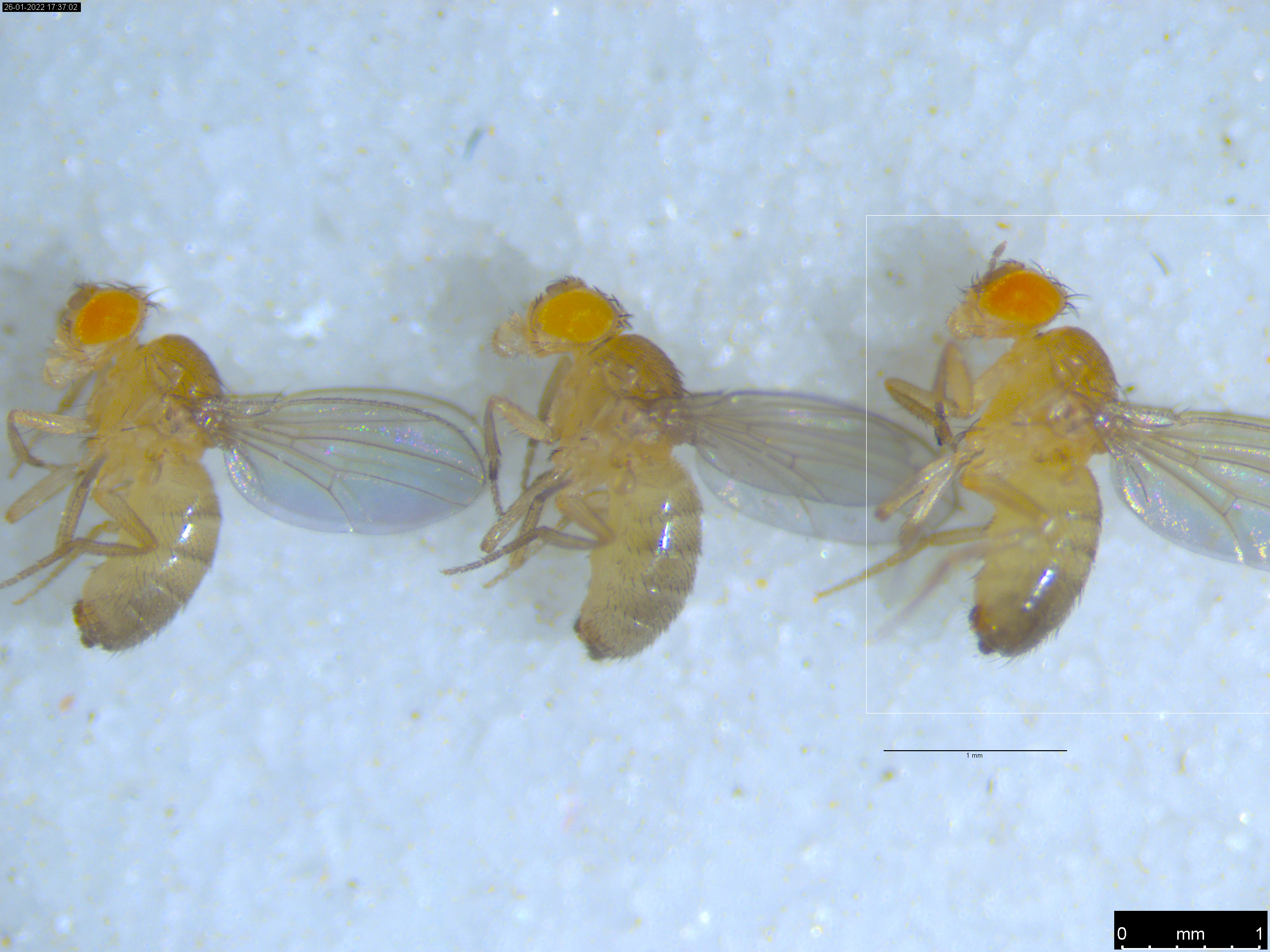

Supplement: Supplementary file 13 — Figure EV5 Source Data [file 44319_2025_574_MOESM13_ESM.zip › Fig. EV5/Fig. EV5_e'-g'/bmmRNAi_fly.tif]

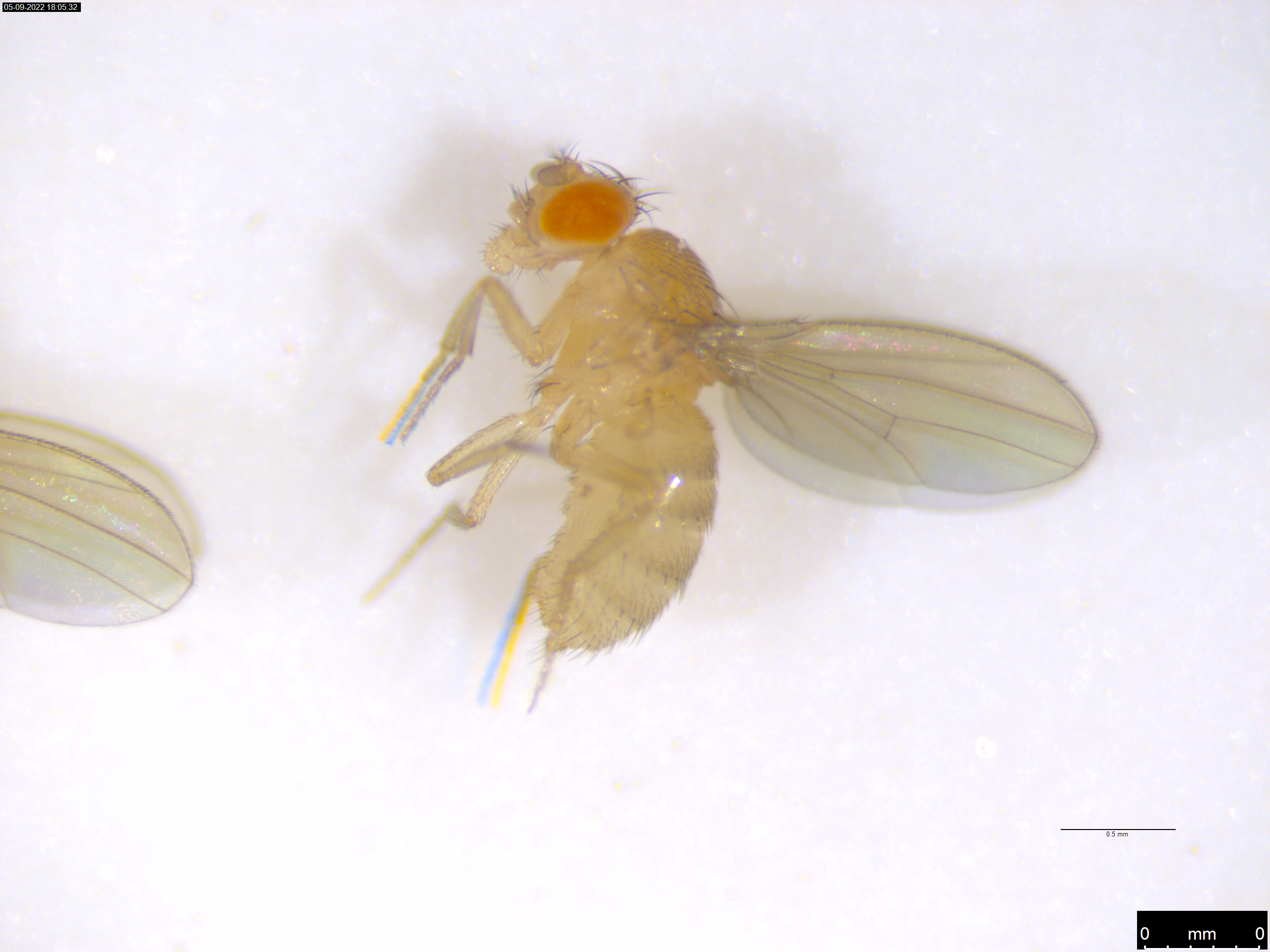

Supplement: Supplementary file 13 — Figure EV5 Source Data [file 44319_2025_574_MOESM13_ESM.zip › Fig. EV5/Fig. EV5_j'-l'/UAS-bmm_fly.tif]

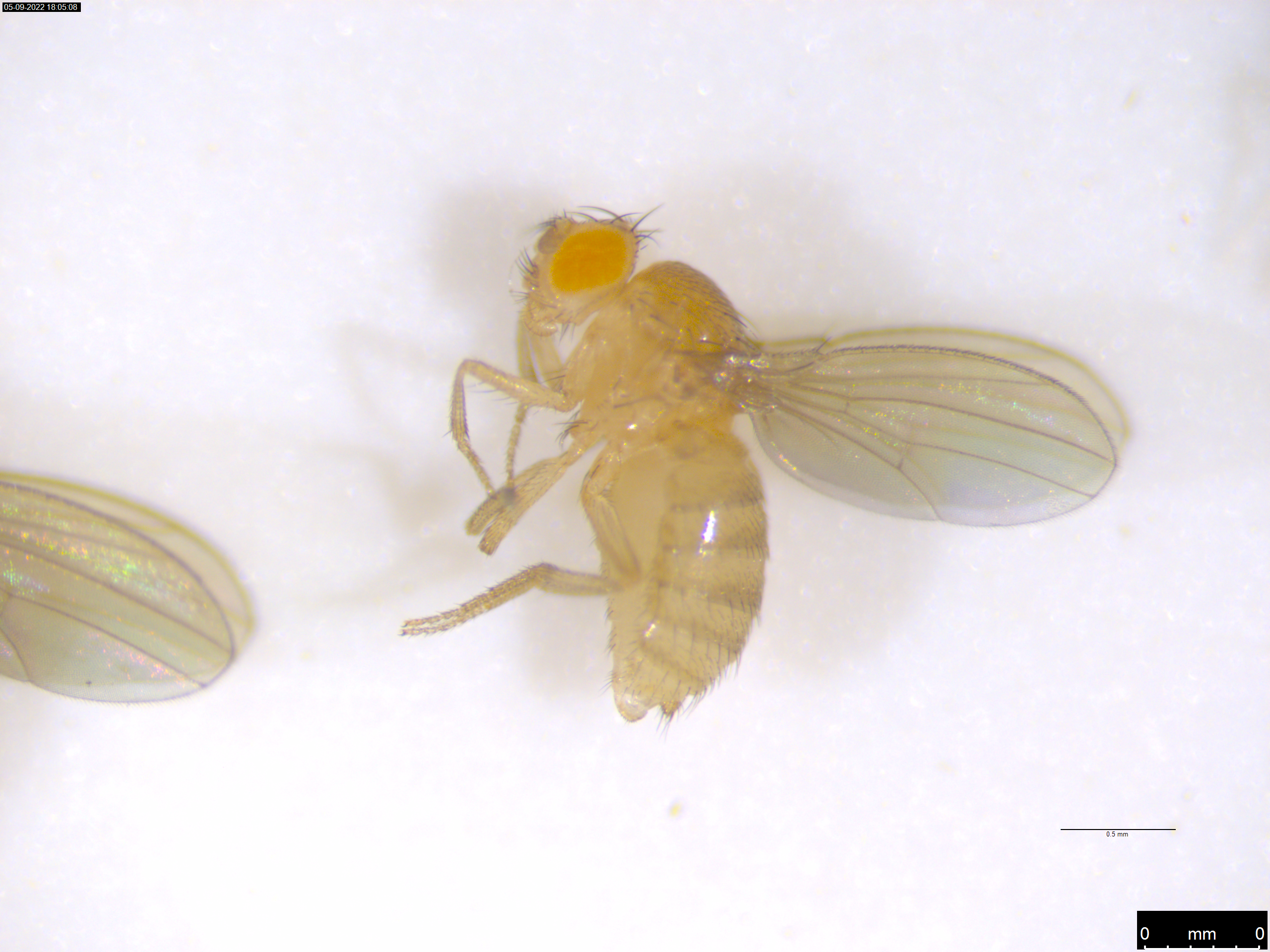

Supplement: Supplementary file 13 — Figure EV5 Source Data [file 44319_2025_574_MOESM13_ESM.zip › Fig. EV5/Fig. EV5_j'-l'/Control_fly.tif]

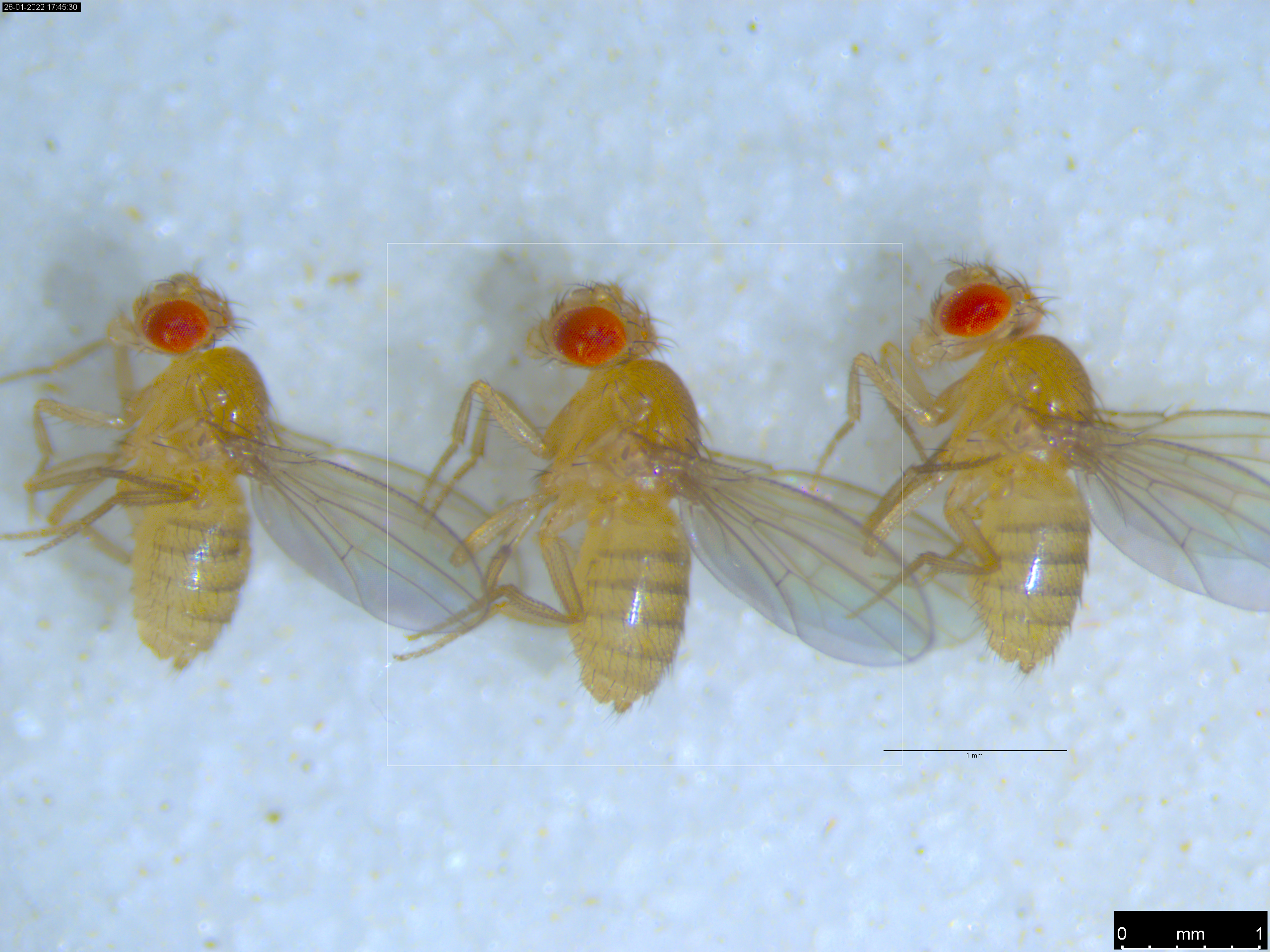

Supplement: Supplementary file 13 — Figure EV5 Source Data [file 44319_2025_574_MOESM13_ESM.zip › Fig. EV5/Fig. EV5_j'-l'/bmmRNAi_fly.tif]

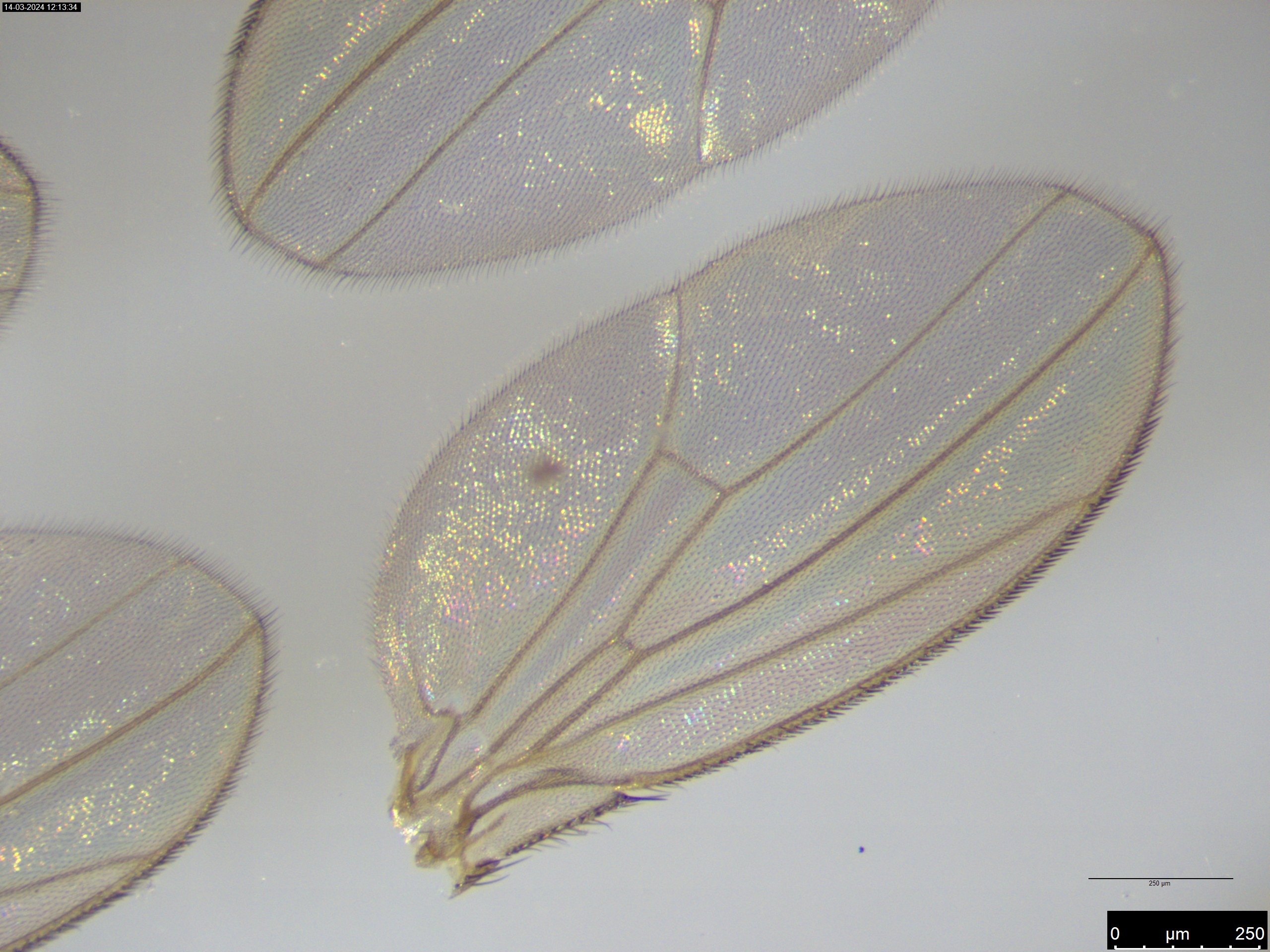

Supplement: Supplementary file 13 — Figure EV5 Source Data [file 44319_2025_574_MOESM13_ESM.zip › Fig. EV5/Fig. EV5_e-g/UAS-bmm_wing.tif]

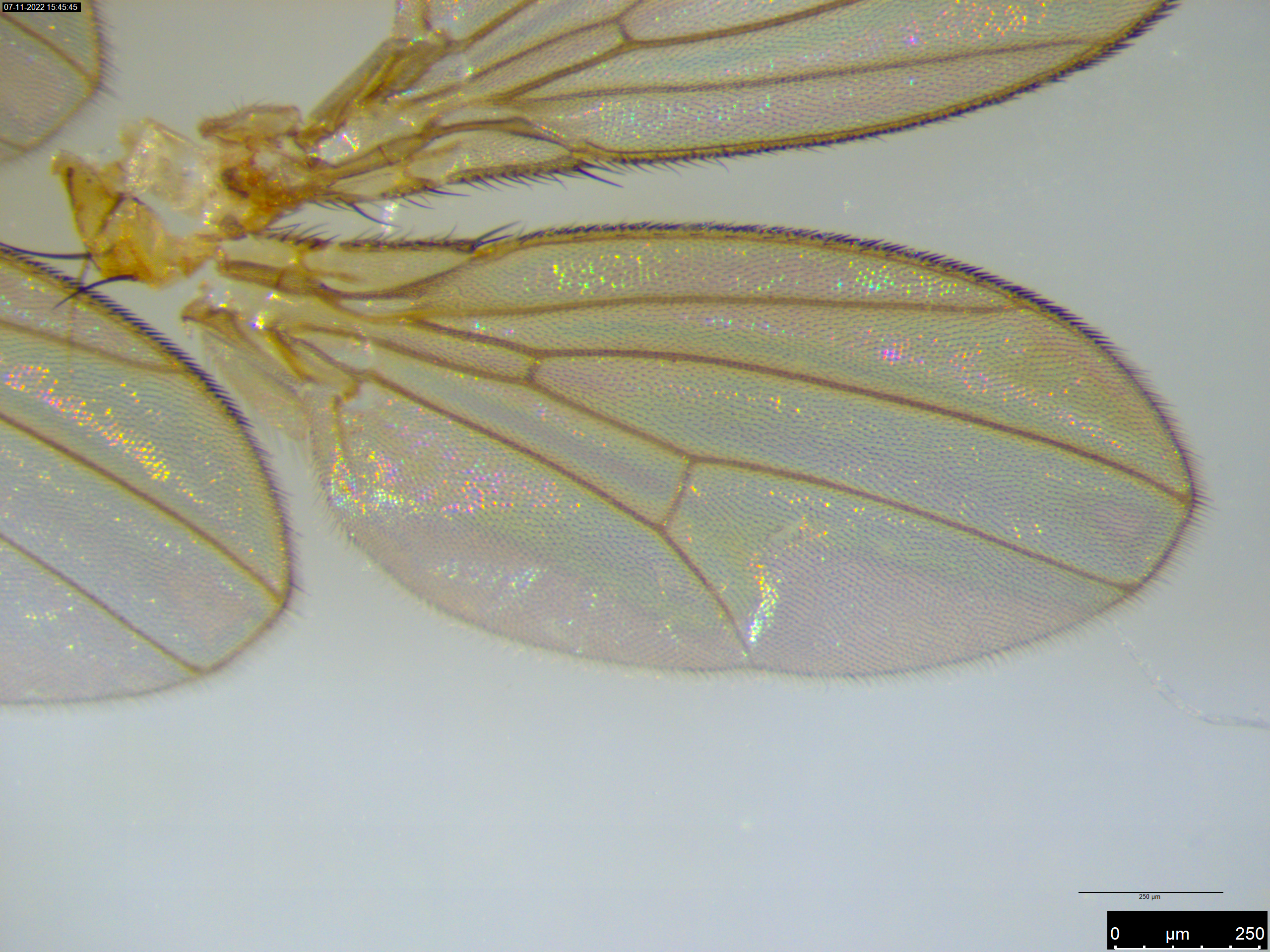

Supplement: Supplementary file 13 — Figure EV5 Source Data [file 44319_2025_574_MOESM13_ESM.zip › Fig. EV5/Fig. EV5_e-g/Control_wing.tif]

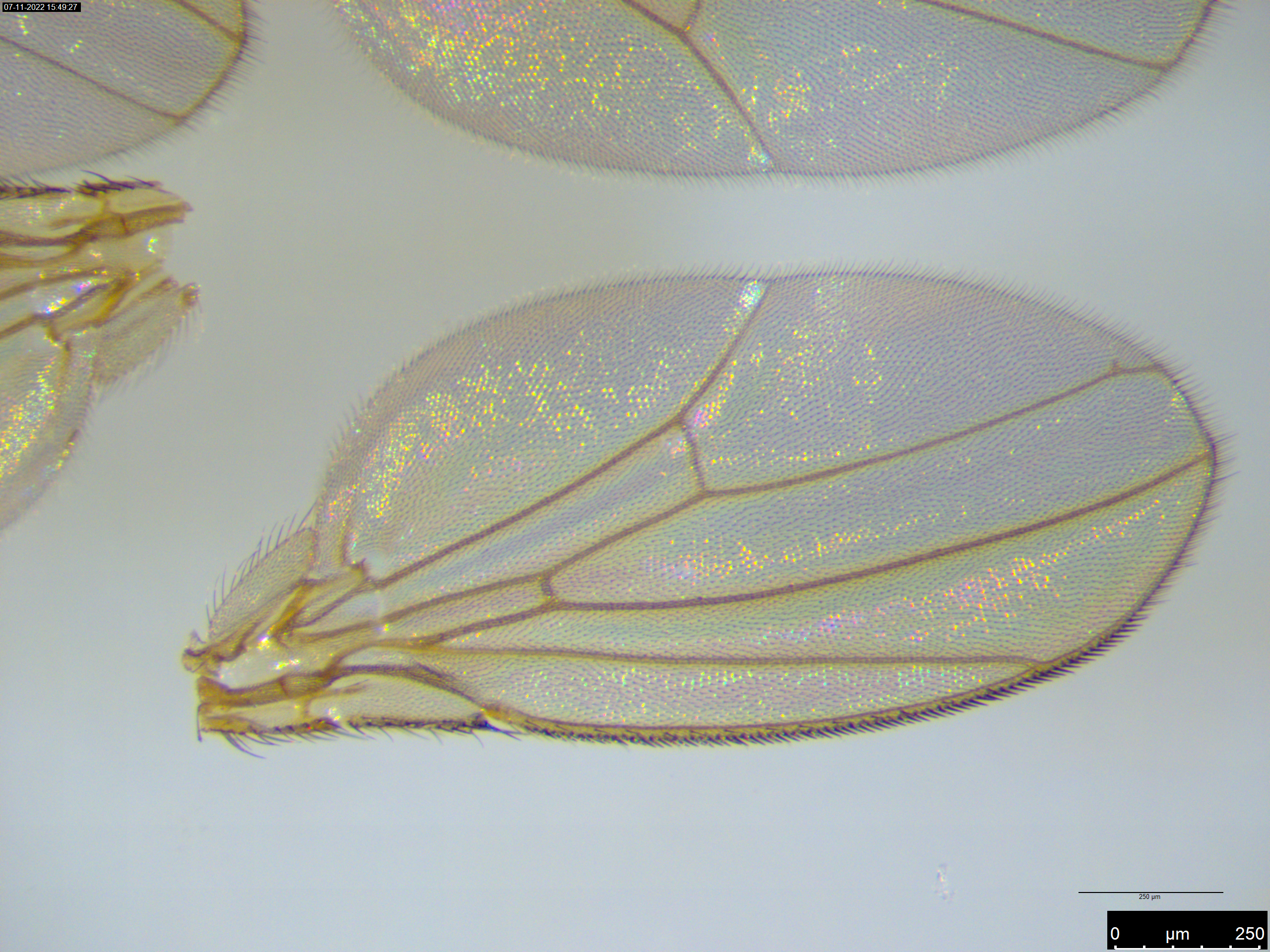

Supplement: Supplementary file 13 — Figure EV5 Source Data [file 44319_2025_574_MOESM13_ESM.zip › Fig. EV5/Fig. EV5_e-g/bmmRNAi_wing.tif]

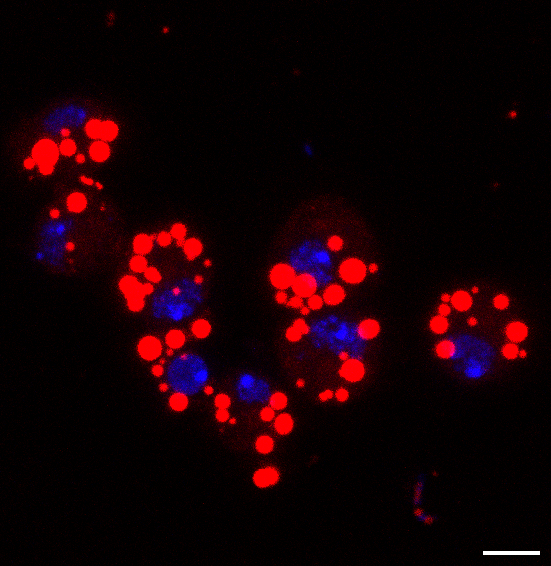

Supplement: Supplementary file 13 — Figure EV5 Source Data [file 44319_2025_574_MOESM13_ESM.zip › Fig. EV5/Fig. EV5_b-d'/bmmRNAi_nile red.tif]

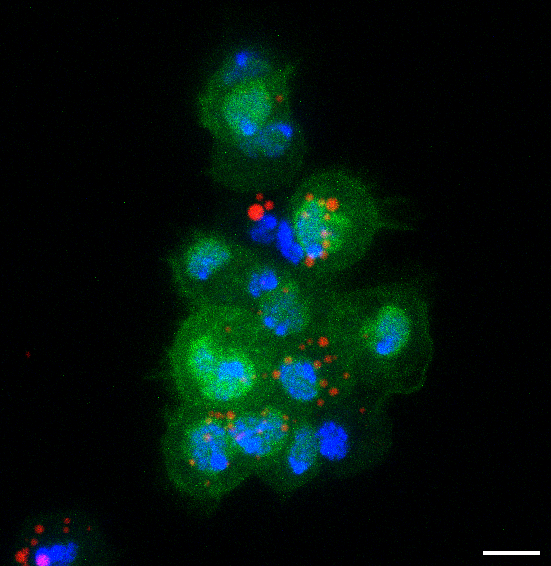

Supplement: Supplementary file 13 — Figure EV5 Source Data [file 44319_2025_574_MOESM13_ESM.zip › Fig. EV5/Fig. EV5_b-d'/UAS-bmm_nile red_GFP.tif]

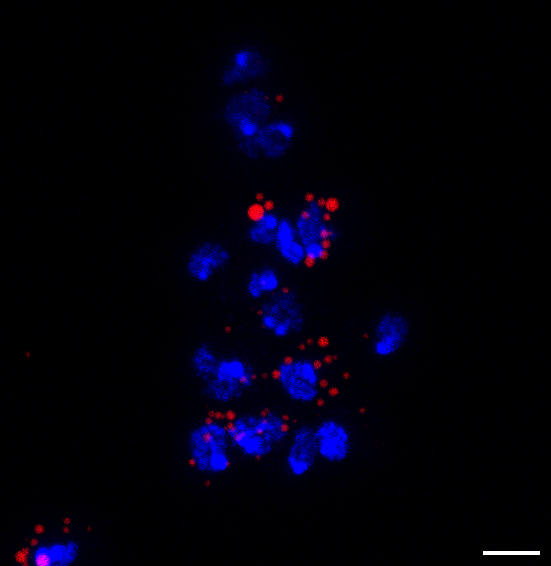

Supplement: Supplementary file 13 — Figure EV5 Source Data [file 44319_2025_574_MOESM13_ESM.zip › Fig. EV5/Fig. EV5_b-d'/UAS-bmm_nile red.tif]

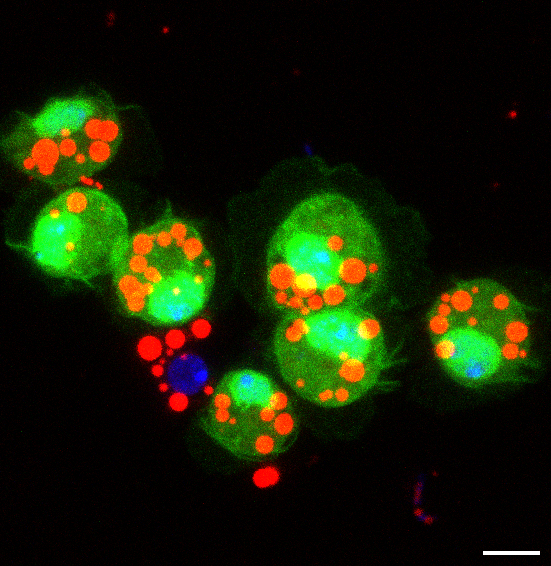

Supplement: Supplementary file 13 — Figure EV5 Source Data [file 44319_2025_574_MOESM13_ESM.zip › Fig. EV5/Fig. EV5_b-d'/bmmRNAi_nile red_GFP.tif]

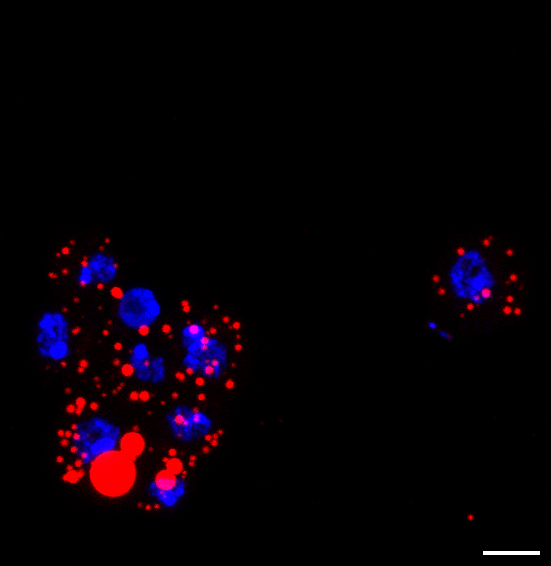

Supplement: Supplementary file 13 — Figure EV5 Source Data [file 44319_2025_574_MOESM13_ESM.zip › Fig. EV5/Fig. EV5_b-d'/Control_nile red.tif]

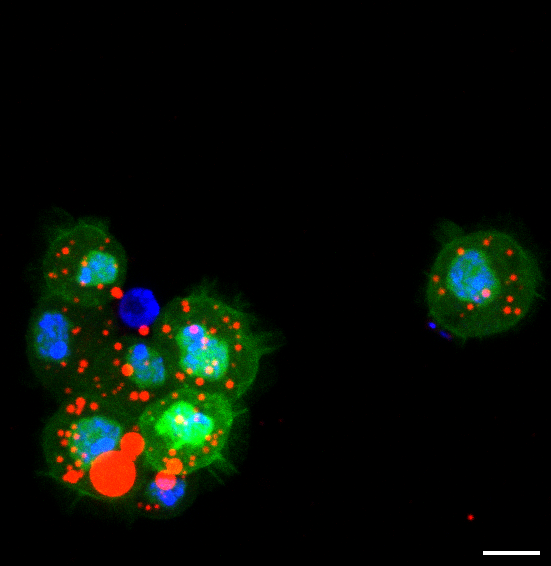

Supplement: Supplementary file 13 — Figure EV5 Source Data [file 44319_2025_574_MOESM13_ESM.zip › Fig. EV5/Fig. EV5_b-d'/Control_nile red_GFP.tif]

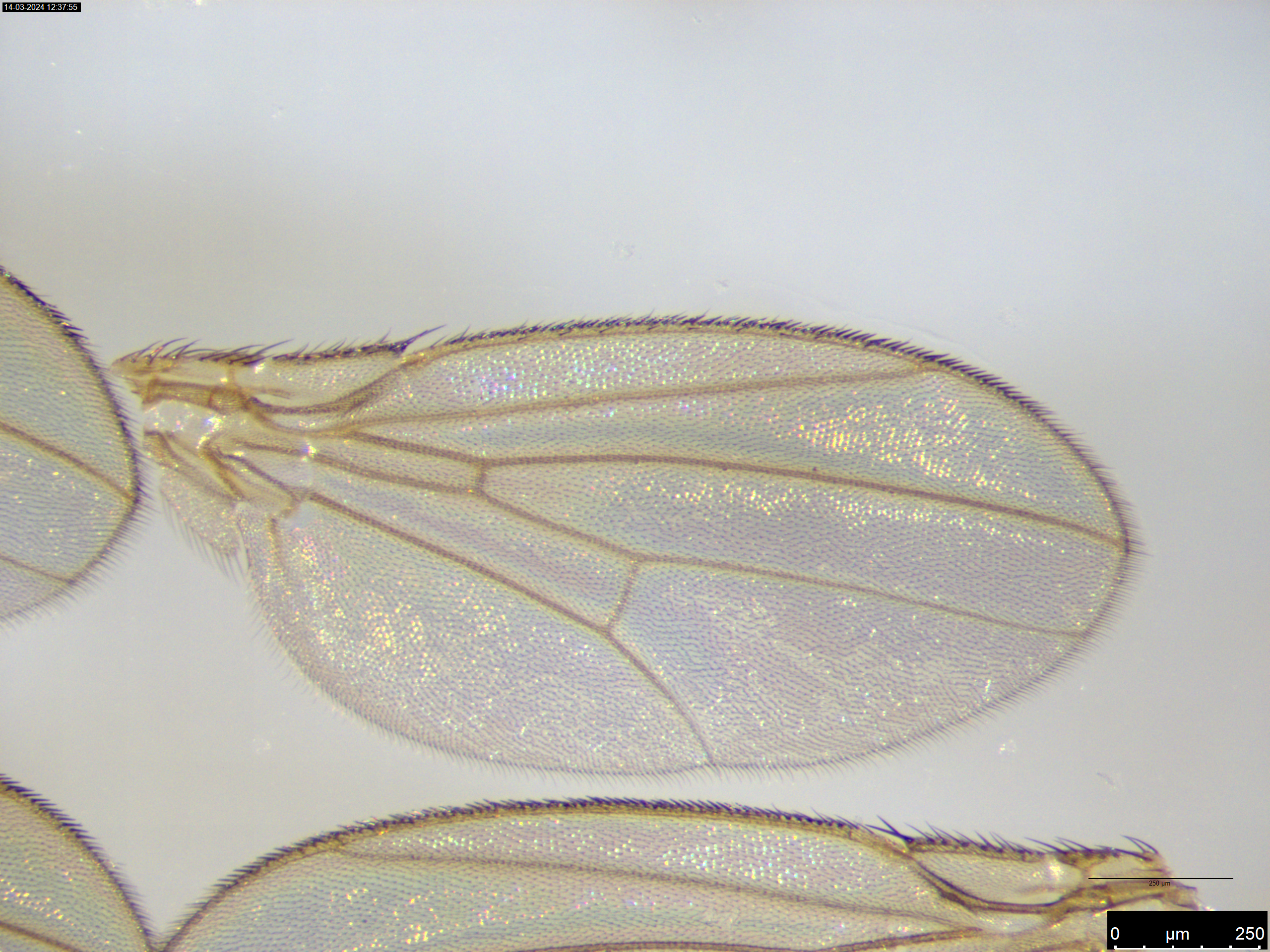

Supplement: Supplementary file 13 — Figure EV5 Source Data [file 44319_2025_574_MOESM13_ESM.zip › Fig. EV5/Fig. EV5_j-l/UAS-bmm_wing.tif]

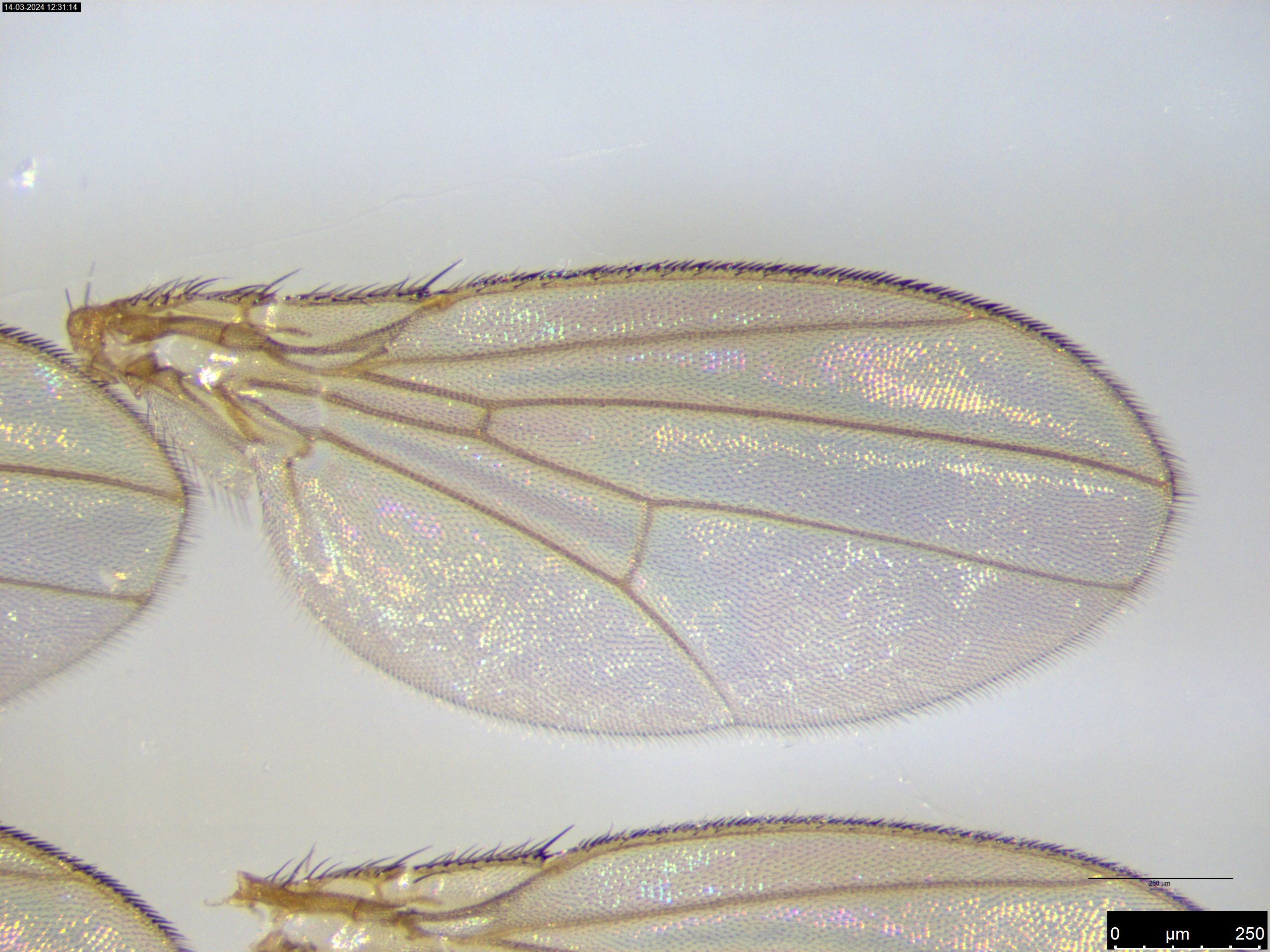

Supplement: Supplementary file 13 — Figure EV5 Source Data [file 44319_2025_574_MOESM13_ESM.zip › Fig. EV5/Fig. EV5_j-l/Control_wing.tif]

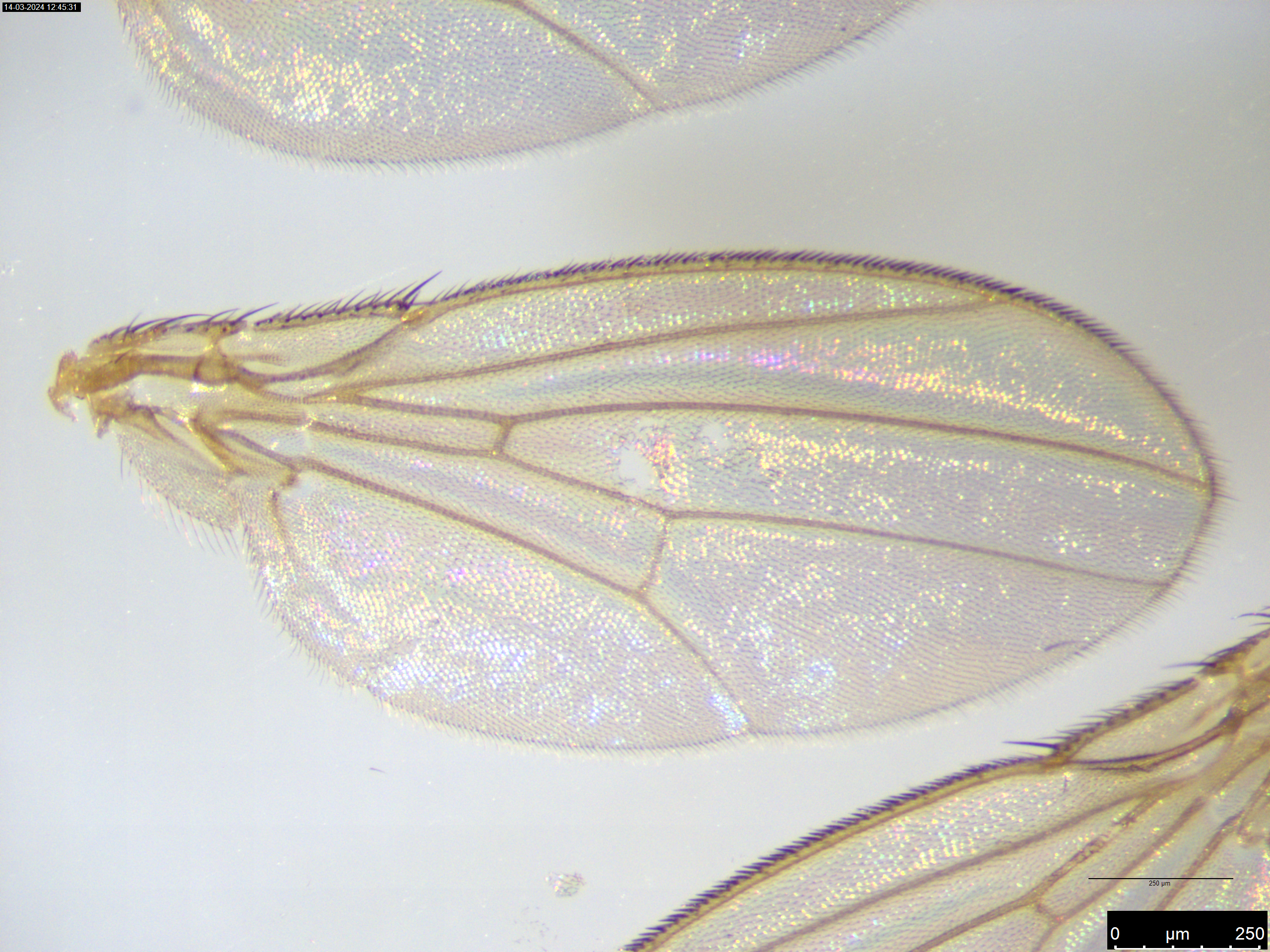

Supplement: Supplementary file 13 — Figure EV5 Source Data [file 44319_2025_574_MOESM13_ESM.zip › Fig. EV5/Fig. EV5_j-l/bmmRNAi_wing.tif]

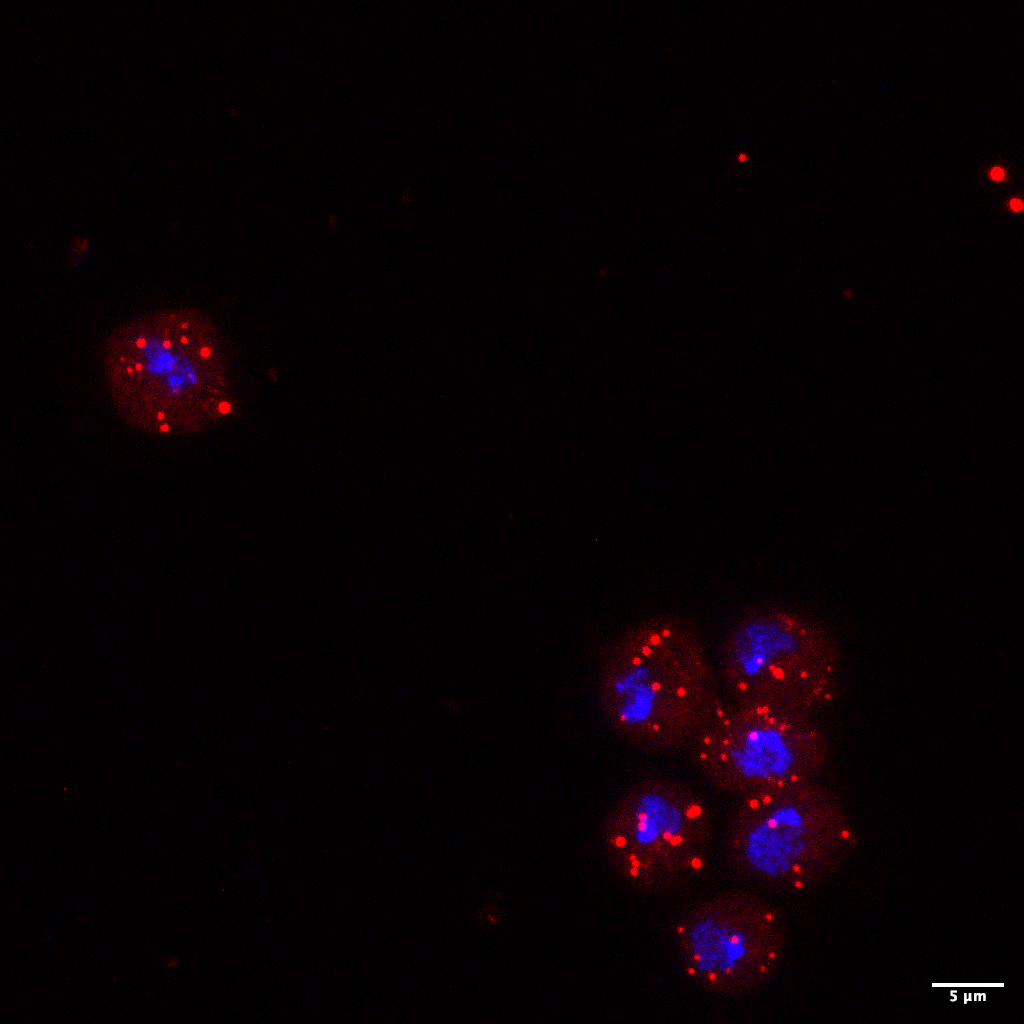

Supplement: Supplementary file 15 — Appendix Figures Source Data [file 44319_2025_574_MOESM15_ESM.zip › Appendix Figures/Appendix Fig. S3/Appendix Fig. S3_d-e'/crqRNAi_nile red.tif]

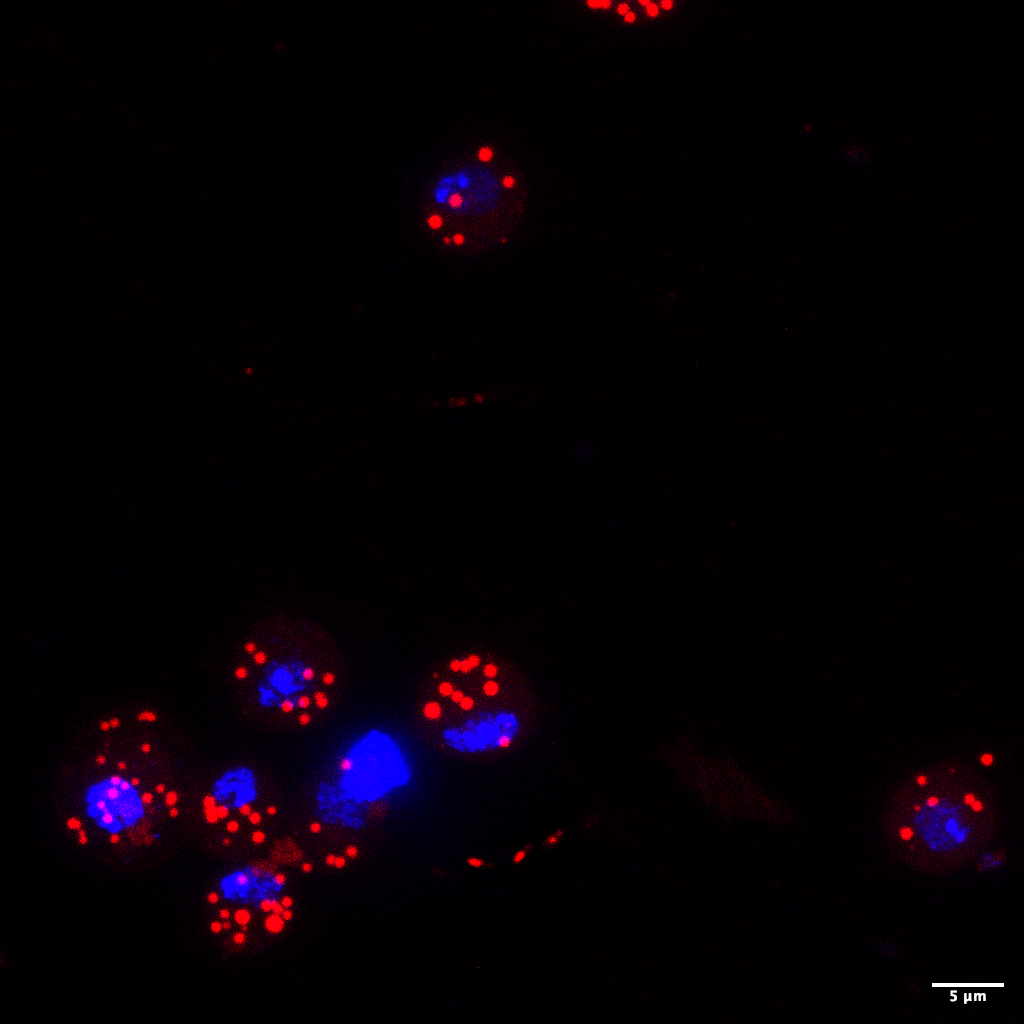

Supplement: Supplementary file 15 — Appendix Figures Source Data [file 44319_2025_574_MOESM15_ESM.zip › Appendix Figures/Appendix Fig. S3/Appendix Fig. S3_d-e'/Control_nile red.tif]

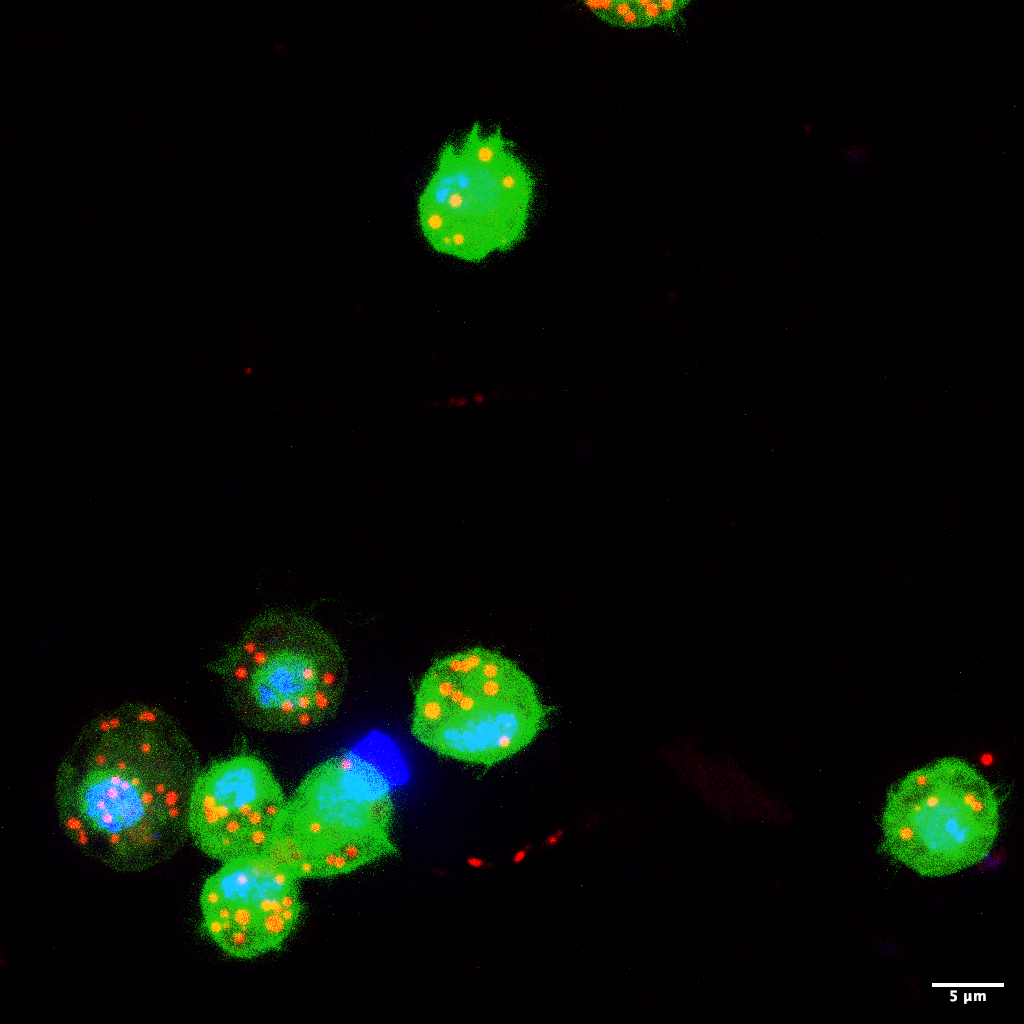

Supplement: Supplementary file 15 — Appendix Figures Source Data [file 44319_2025_574_MOESM15_ESM.zip › Appendix Figures/Appendix Fig. S3/Appendix Fig. S3_d-e'/Control_nile red_GFP.tif]

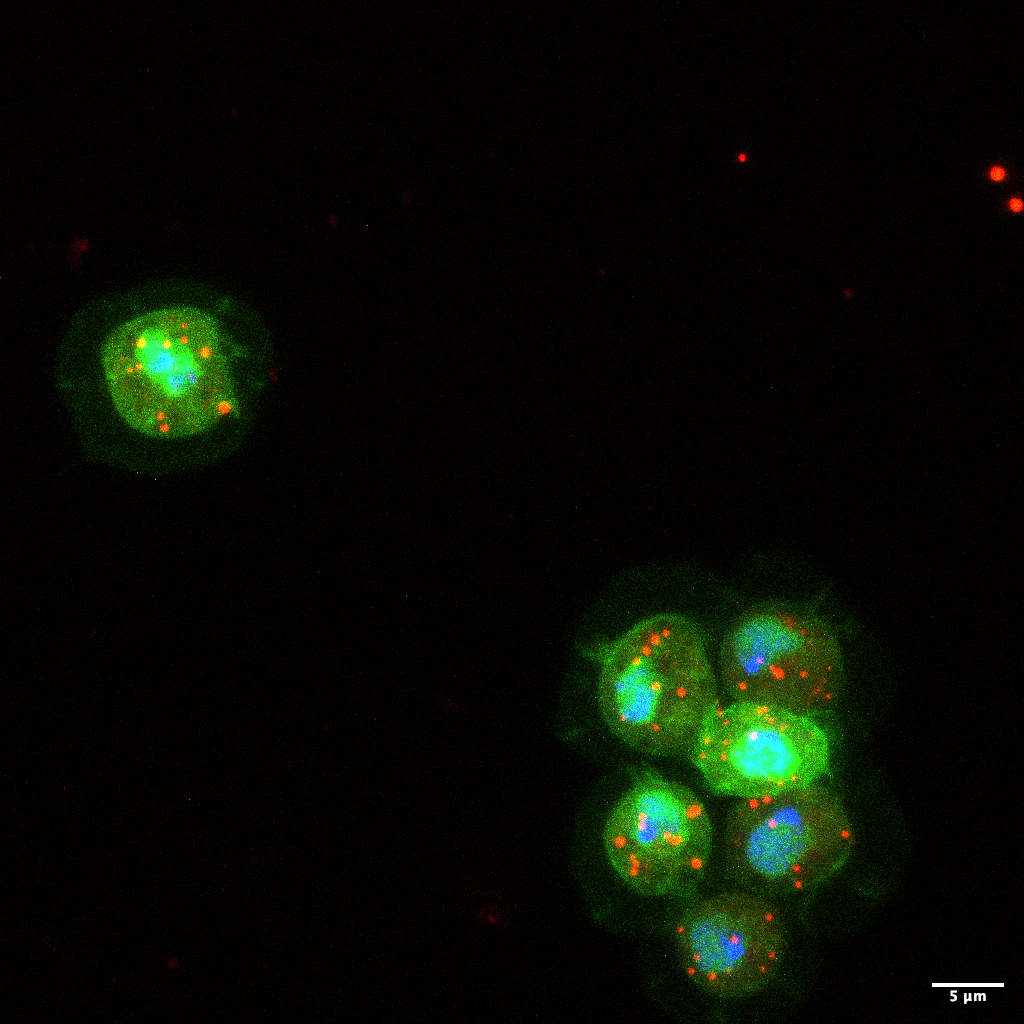

Supplement: Supplementary file 15 — Appendix Figures Source Data [file 44319_2025_574_MOESM15_ESM.zip › Appendix Figures/Appendix Fig. S3/Appendix Fig. S3_d-e'/crqRNAi_nile red_GFP.tif]

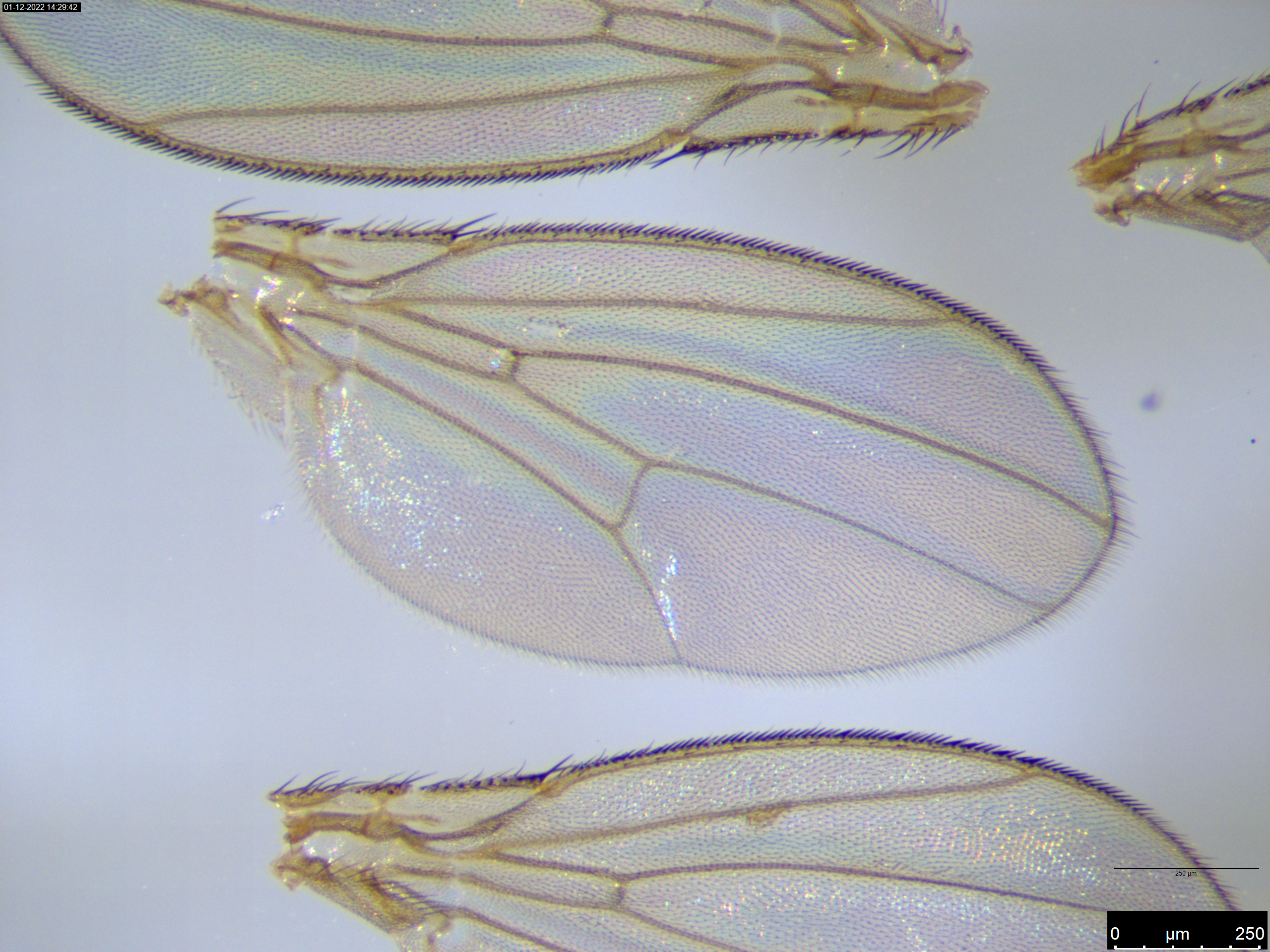

Supplement: Supplementary file 15 — Appendix Figures Source Data [file 44319_2025_574_MOESM15_ESM.zip › Appendix Figures/Appendix Fig. S3/Appendix Fig. S3_f-g'/crqRNAi_wing.tif]

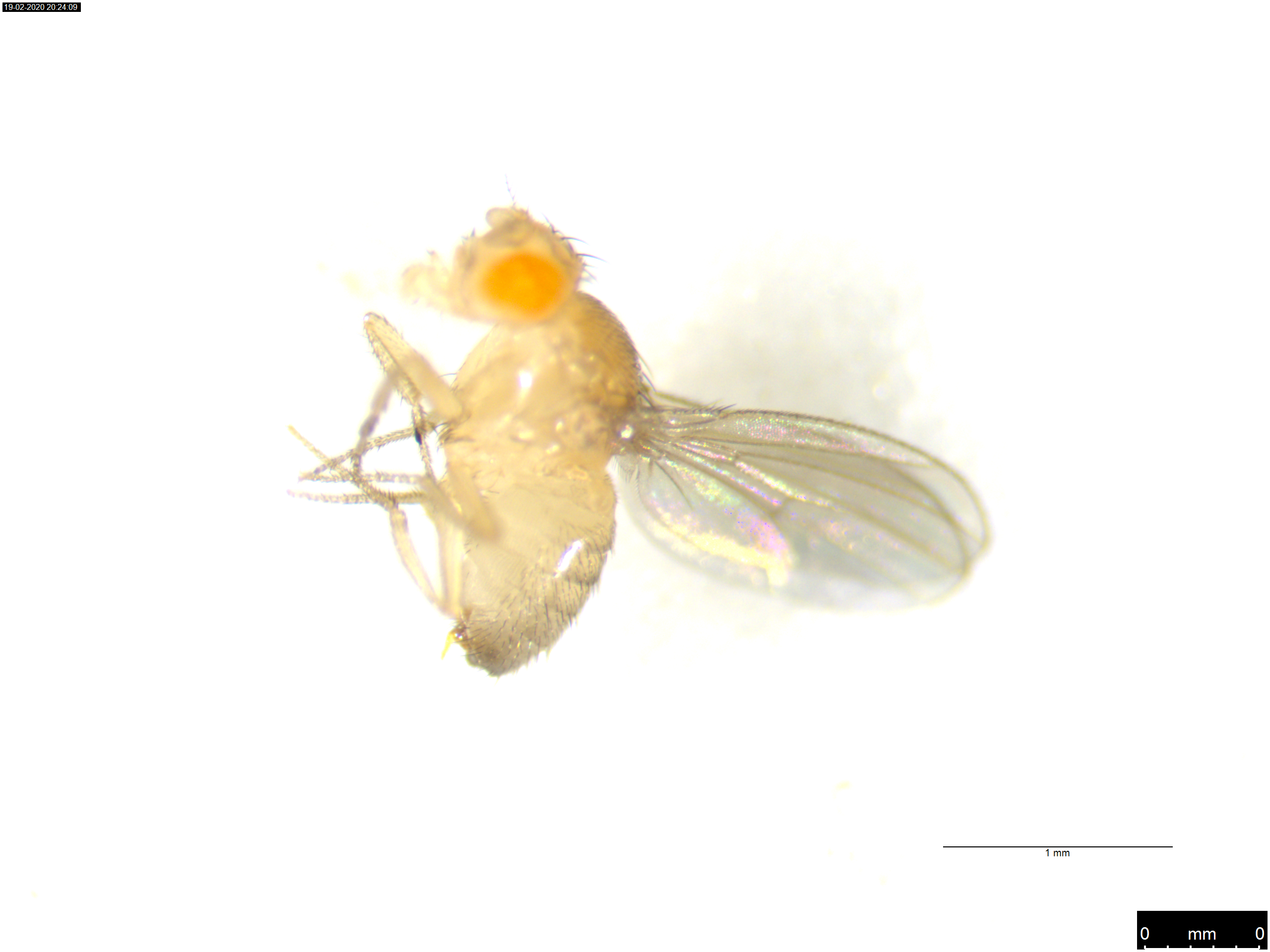

Supplement: Supplementary file 15 — Appendix Figures Source Data [file 44319_2025_574_MOESM15_ESM.zip › Appendix Figures/Appendix Fig. S3/Appendix Fig. S3_f-g'/Control_fly.tif]

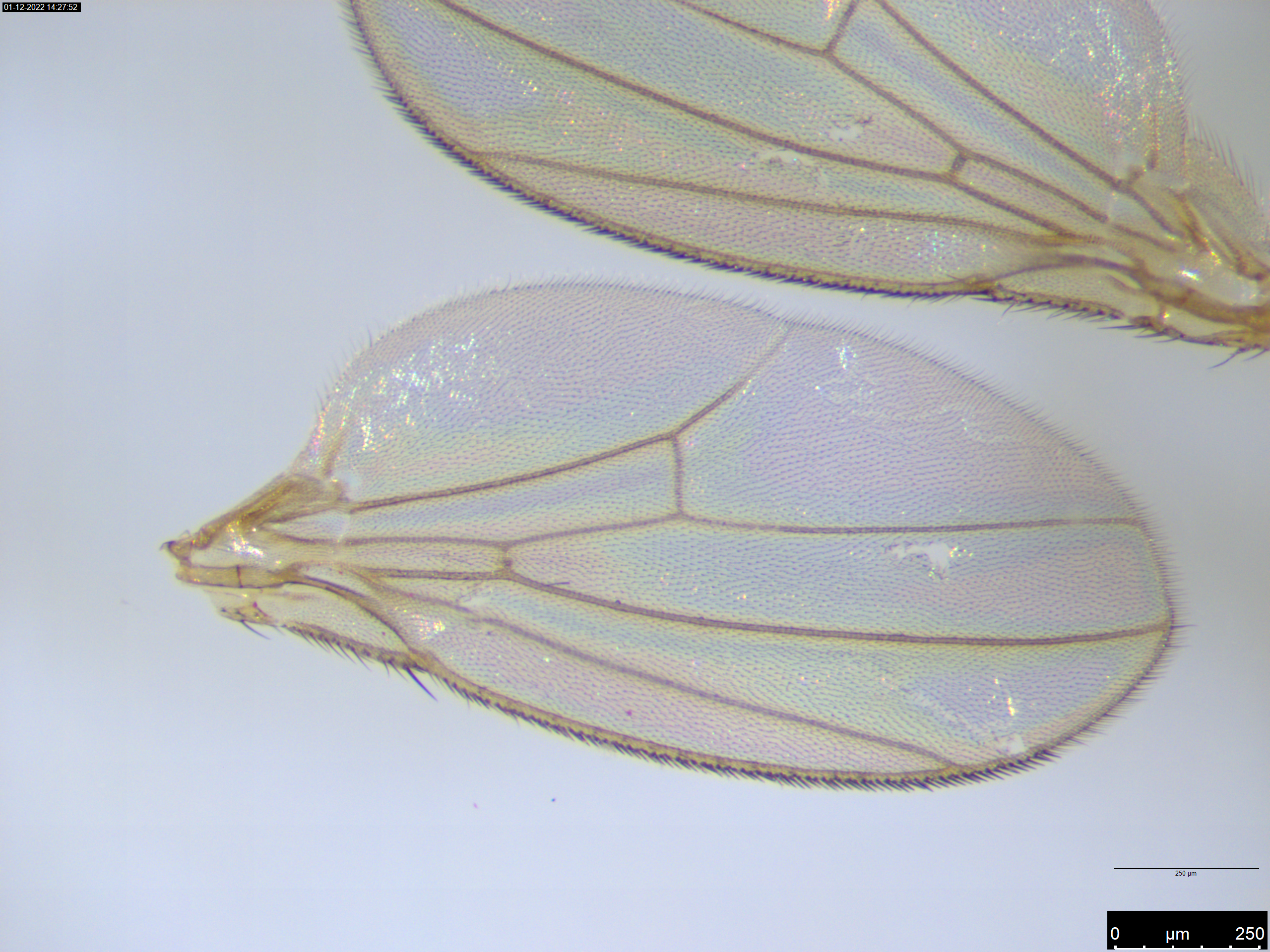

Supplement: Supplementary file 15 — Appendix Figures Source Data [file 44319_2025_574_MOESM15_ESM.zip › Appendix Figures/Appendix Fig. S3/Appendix Fig. S3_f-g'/Control_wing.tif]

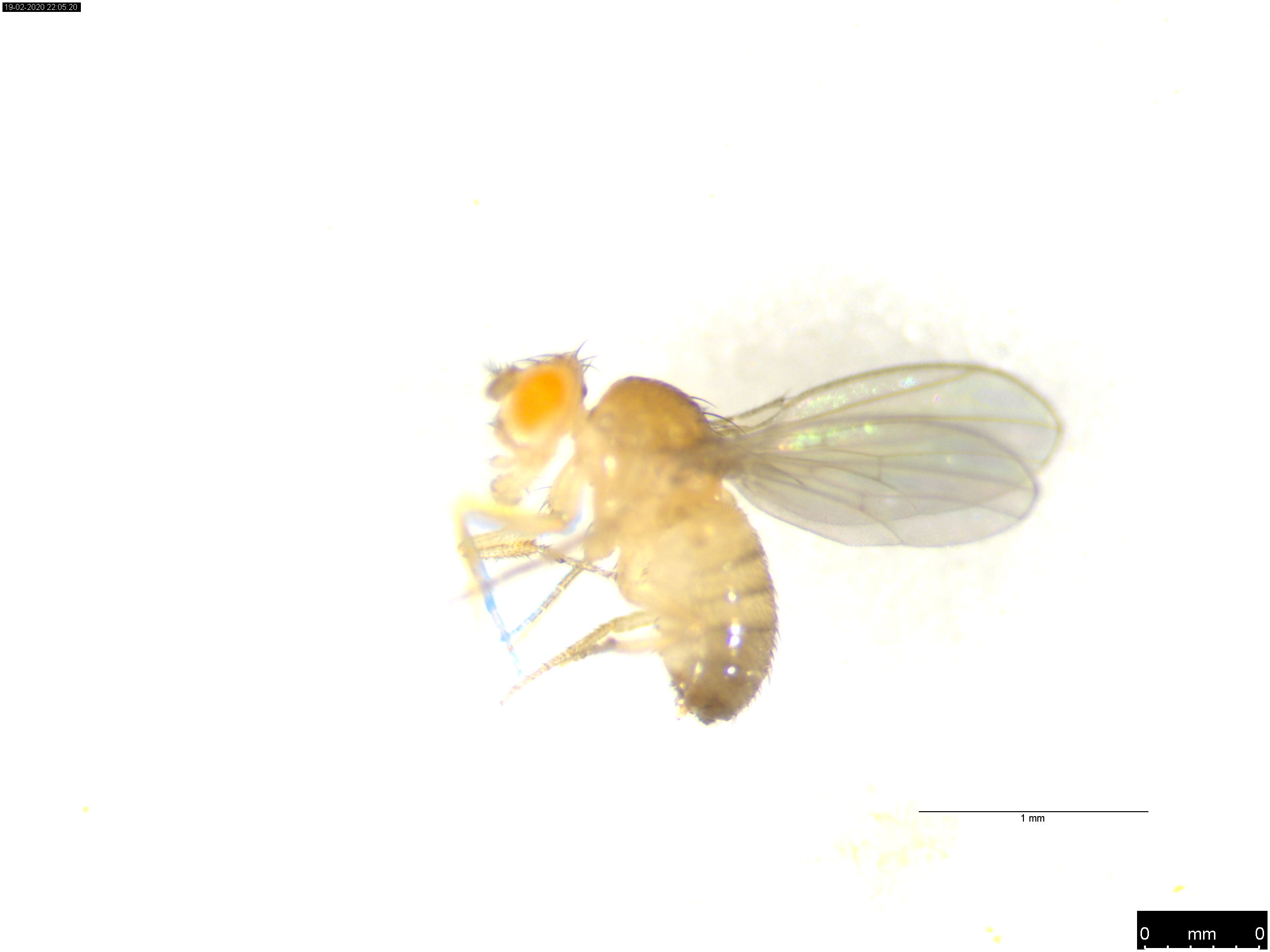

Supplement: Supplementary file 15 — Appendix Figures Source Data [file 44319_2025_574_MOESM15_ESM.zip › Appendix Figures/Appendix Fig. S3/Appendix Fig. S3_f-g'/crqRNAi_fly.tif]

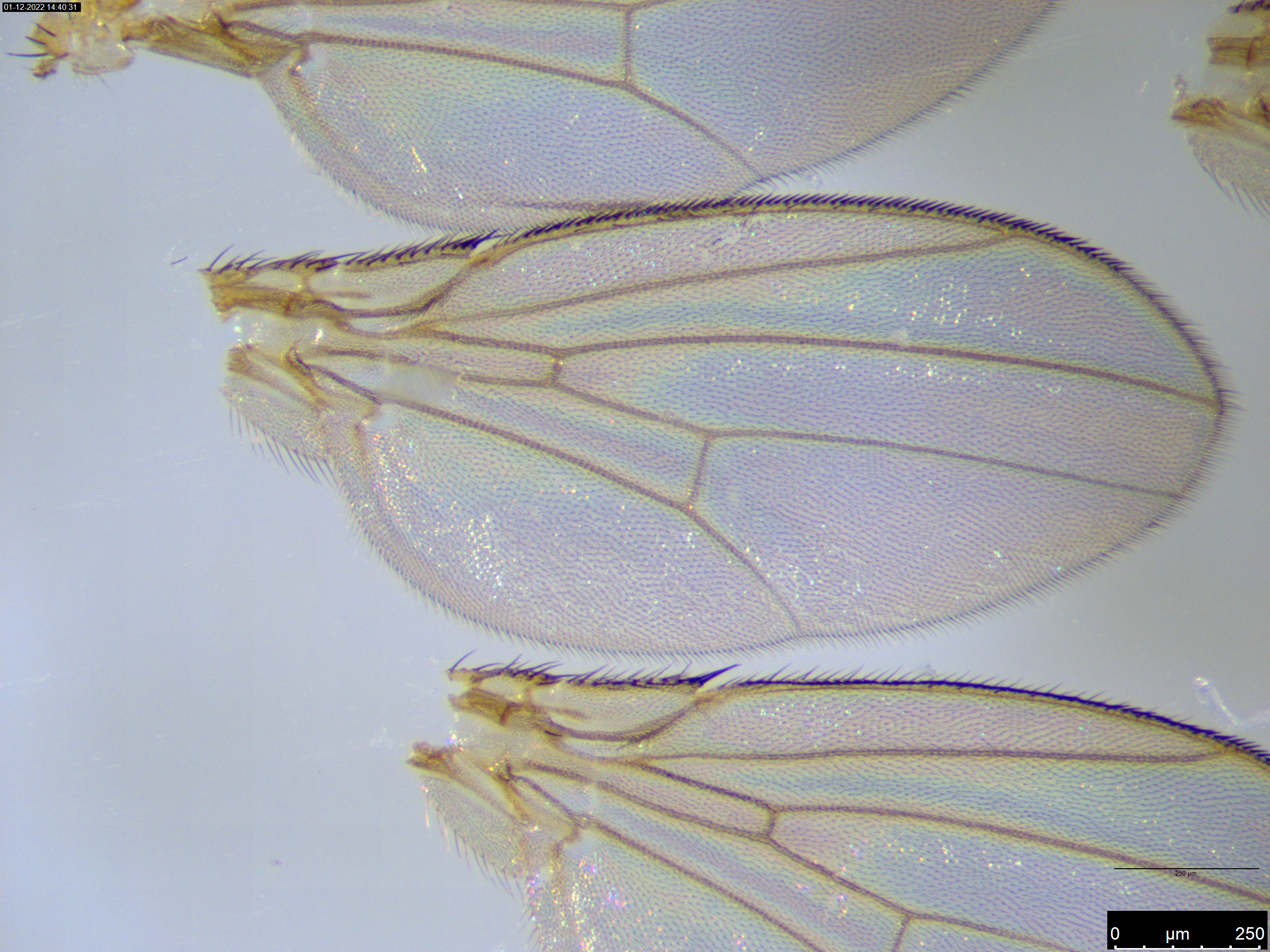

Supplement: Supplementary file 15 — Appendix Figures Source Data [file 44319_2025_574_MOESM15_ESM.zip › Appendix Figures/Appendix Fig. S3/Appendix Fig. S3_i-j'/crqRNAi_wing.tif]

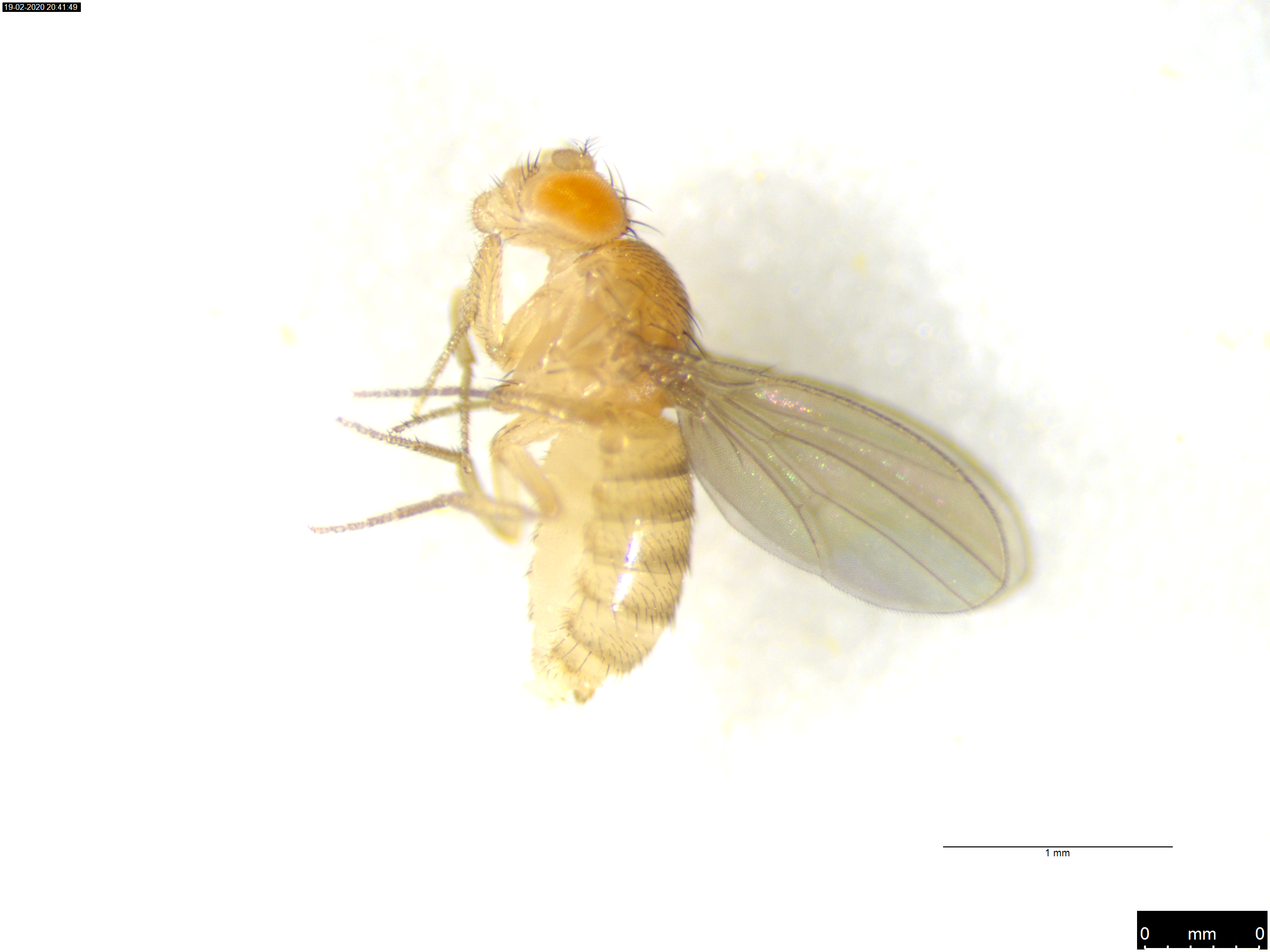

Supplement: Supplementary file 15 — Appendix Figures Source Data [file 44319_2025_574_MOESM15_ESM.zip › Appendix Figures/Appendix Fig. S3/Appendix Fig. S3_i-j'/Control_fly.tif]

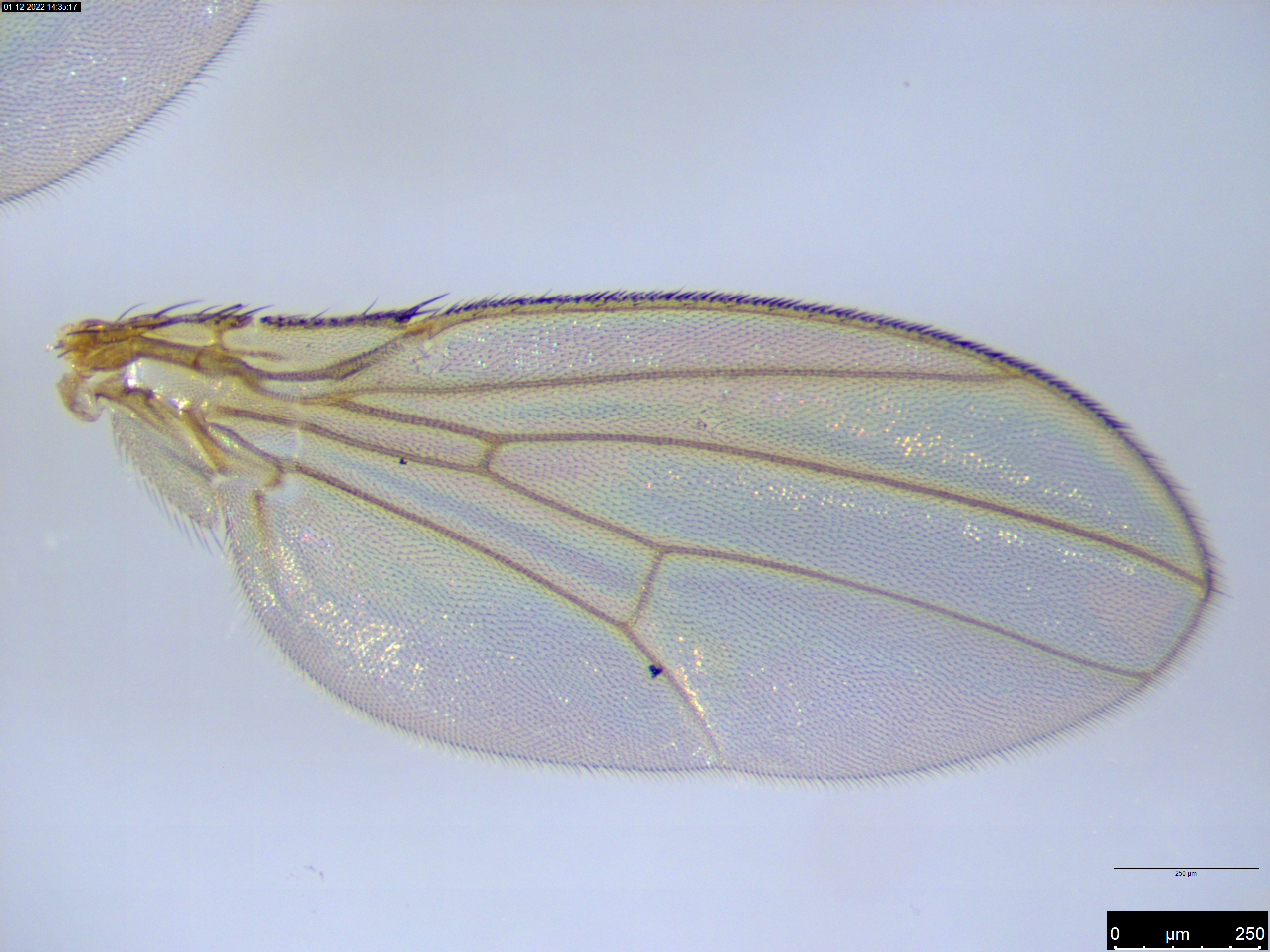

Supplement: Supplementary file 15 — Appendix Figures Source Data [file 44319_2025_574_MOESM15_ESM.zip › Appendix Figures/Appendix Fig. S3/Appendix Fig. S3_i-j'/Control_wing.tif]

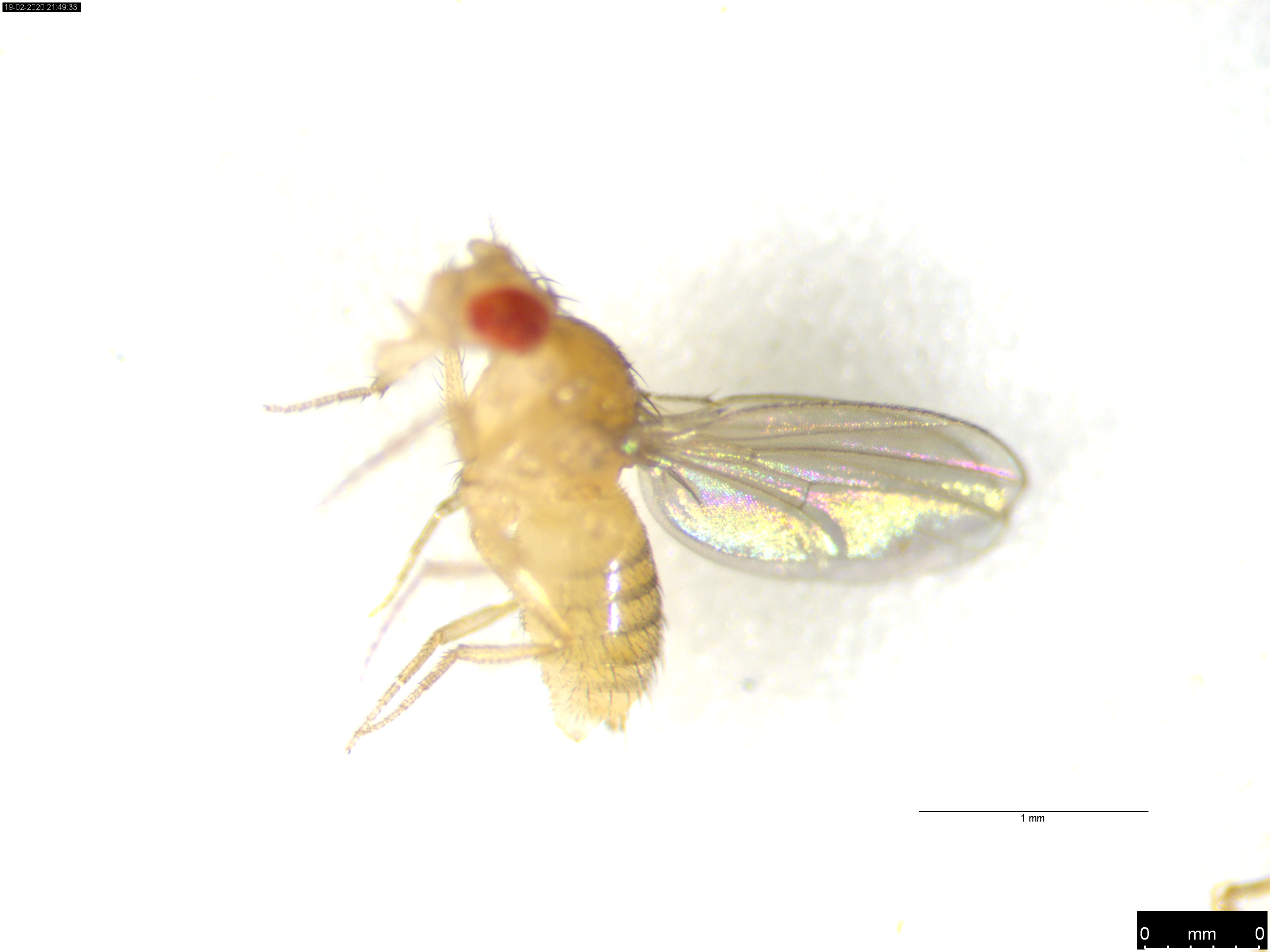

Supplement: Supplementary file 15 — Appendix Figures Source Data [file 44319_2025_574_MOESM15_ESM.zip › Appendix Figures/Appendix Fig. S3/Appendix Fig. S3_i-j'/crqRNAi_fly.tif]

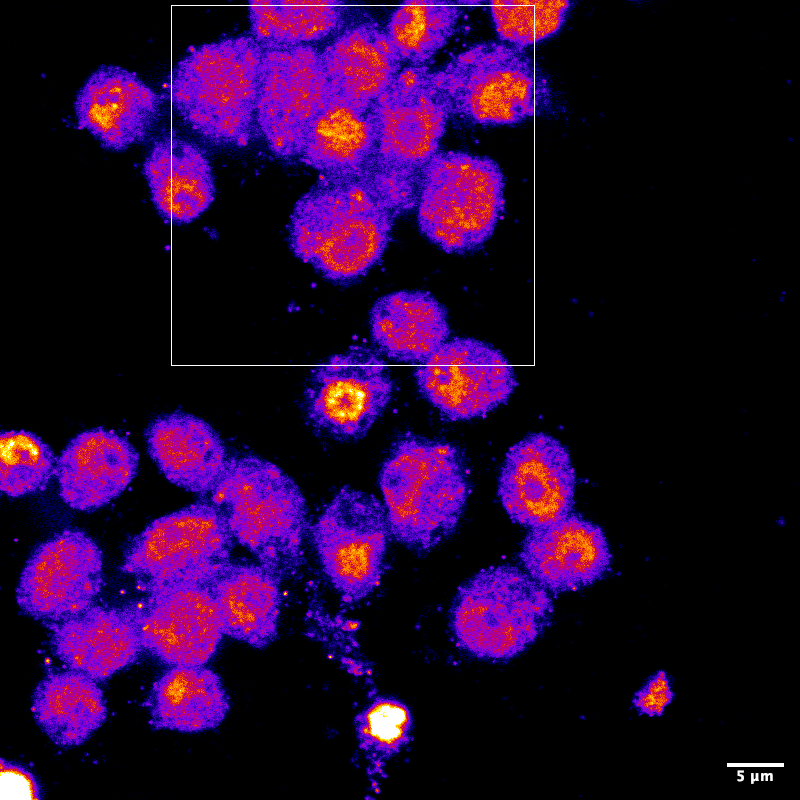

Supplement: Supplementary file 15 — Appendix Figures Source Data [file 44319_2025_574_MOESM15_ESM.zip › Appendix Figures/Appendix Fig. S3/Appendix Fig. S3_a,b/RF_crq.tif]

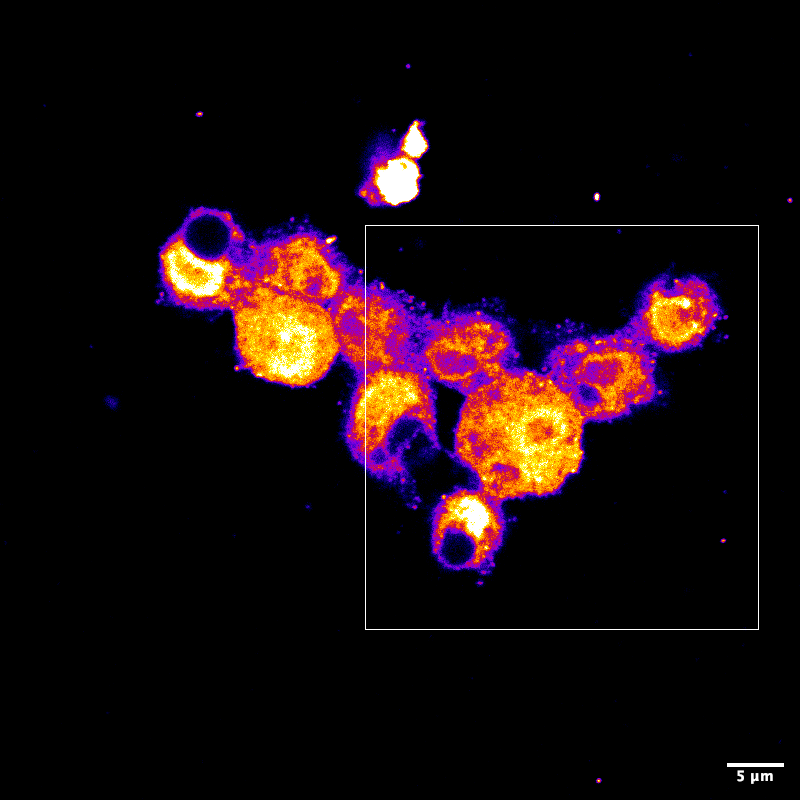

Supplement: Supplementary file 15 — Appendix Figures Source Data [file 44319_2025_574_MOESM15_ESM.zip › Appendix Figures/Appendix Fig. S3/Appendix Fig. S3_a,b/Ct.HSD_crq.tif]
