# Supplementary material for: Macrophage metabolic reprogramming during dietary stress influences adult body size in Drosophila
Source: EMBO Rep. 2025 Sep 9;26(22):5397–430. doi: 10.1038/s44319-025-00574-7 (PMC12635341; doi:10.1038/s44319-025-00574-7)
Supplement: Supplementary file 16 — Expanded View Figures [file 44319_2025_574_MOESM16_ESM.pdf]

Expanded View Figures

Figure EV1. Dietary sugar stress affects larval macrophage physiology.

(**A–B‴**) HSD alters Hml+ immune cell numbers. Representative images of sessile (**A–A‴**) and circulatory immune cells (**B–B‴**), on RF (**A**, **B**), 4 hr.HSD (**A′**, **B′**) and Ct.HSD (**A″**, **B″**). Compared to sessile immune cells in (**A**) *Hml*$^{\Delta}$*>GFP/w*^{1118} (Control (RF), (**A′**) *Hml*$^{\Delta}$*>GFP/w*^{1118} (4 hr.HSD) did not show any dramatic change in their numbers, but (**A″**) *Hml*$^{\Delta}$*>GFP/w*^{1118} (Ct.HSD) larvae show significant reduction in Hml+ sessile population. See quantifications in (**A‴**). Circulating cell numbers in (**B′**) *Hml*$^{\Delta}$*>GFP/w*^{1118} (4 hr.HSD) and (**B″**) *Hml*$^{\Delta}$*>GFP/w*^{1118} (Ct.HSD) showed no striking difference compared to the Control (**B**). See quantifications in (**B‴**). (**A‴**) Quantification of sessile Hml+ immune cell numbers in *Hml*$^{\Delta}$*>GFP/w*^{1118} (Control, RF, *N* = 3, *n* = 18), *Hml*$^{\Delta}$*>GFP/w*^{1118} (4 hr.HSD, *N* = 3, *n* = 18, *P* = 0.5504) and HmlΔ>GFP/w1118 (Ct.HSD, *N* = 3, *n* = 18, *P* < 0.0001). (**B‴**) Quantification of circulatory Hml+ immune cell numbers in *Hml*$^{\Delta}$*>GFP/w*^{1118} (Control, RF, *N* = 3, *n* = 18), *Hml*$^{\Delta}$*>GFP/w*^{1118} (4 hr.HSD, *N* = 3, *n* = 18, *P* = 0.1771) and *Hml*$^{\Delta}$*>GFP/w*^{1118} (Ct.HSD, *N* = 3, *n* = 18, *P* = 0.9668). (**C–C″**) Representative images of immune cells to assess ROS levels. (**C**) ROS level in immune cell of *Hml*$^{\Delta}$*>GFP/w*^{1118} (Control, RF). (**C′**) *Hml*$^{\Delta}$*>GFP/w*^{1118} (4 hr.HSD) and (**C″**) *Hml*$^{\Delta}$*>GFP/w*^{1118} (Ct.HSD) show no change in ROS levels as compared to Control (**C**). (**D–D″**) Representative images of immune cells with Nile red staining to assess for lipid droplet accumulation. (**D**) *Hml*$^{\Delta}$*>GFP/w*^{1118} (Control, RF). (**D′**) *Hml*$^{\Delta}$*>GFP/w*^{1118} (4 hr.HSD) and (**D″**) *Hml*$^{\Delta}$*>GFP/w*^{1118} (Ct.HSD) show gradual increase in immune cell lipid content compared to Control (**D**). (**E–E″**) Representative images of immune cells to assess phagocytosis through bead uptake assay 15 min post incubation. (**E**) *Hml*$^{\Delta}$*>GFP/w*^{1118} (Control, RF). (**E′**) *Hml*$^{\Delta}$*>GFP/w*^{1118} (4 hr.HSD) and (**E″**) *Hml*$^{\Delta}$*>GFP/w*^{1118} (Ct.HSD) show reduction in number of internalized beads when compared to Control (**E**). (**F–F″**) Representative confocal images of immune cells assessed for cellular morphology. (**F**) *Hml*$^{\Delta}$*>GFP/w*^{1118} (Control, RF). (**F′**) *Hml*$^{\Delta}$*>GFP/w*^{1118} (4 hr.HSD) and (**F″**) *Hml*$^{\Delta}$*>GFP/w*^{1118} (Ct.HSD) show reduction both in number as well as in length of filopodia compared to Control (**F**). (**G–G″**) Representative images of immune cells assessed for lipid droplets with *UAS-LSD2-GFP* reporter line. (**G**) *actin-GAL4/UAS-LSD2-GFP* (Control, RF). (**G′**) *actin GAL4/UAS-LSD2-GFP* (4 hr.HSD) and (**G″**) *actin-GAL4/UAS-LSD2-GFP* (Ct.HSD) show gradual increase in immune cell lipid droplets (green) compared to Control (**G**). Data information: DNA is stained with DAPI (blue), immune cells are marked in green (*Hml*$^{\Delta}$*>UAS-GFP*). (**A–B″**) scale bar is 100 µm and (**C–G″**) scale bar is 5 µm. Comparisons for significance are with regular food (RF) conditions and asterisks mark statistically significant differences (**P* < 0.05; ***P* < 0.01; ****P* < 0.001; *****P* < 0.0001). The statistical analysis applied for (**A‴**, **B‴**) is two-way ANOVA with Dunnett's multiple comparison test. RF, 4 hr.HSD and Ct.HSD indicate conditions of larvae fed on regular food (RF), 4 hour high sugar diet (4 hr.HSD) and constitutive high sugar diet (Ct.HSD), respectively. *N* indicates the number of independent biological replicates, and n refers to the total number of animals analyzed. See methods for further details on larval numbers and sample analysis for each of the experiments. In bar graphs, data are presented as mean ± SD. Source data are available online for this figure.

 © The Author(s)

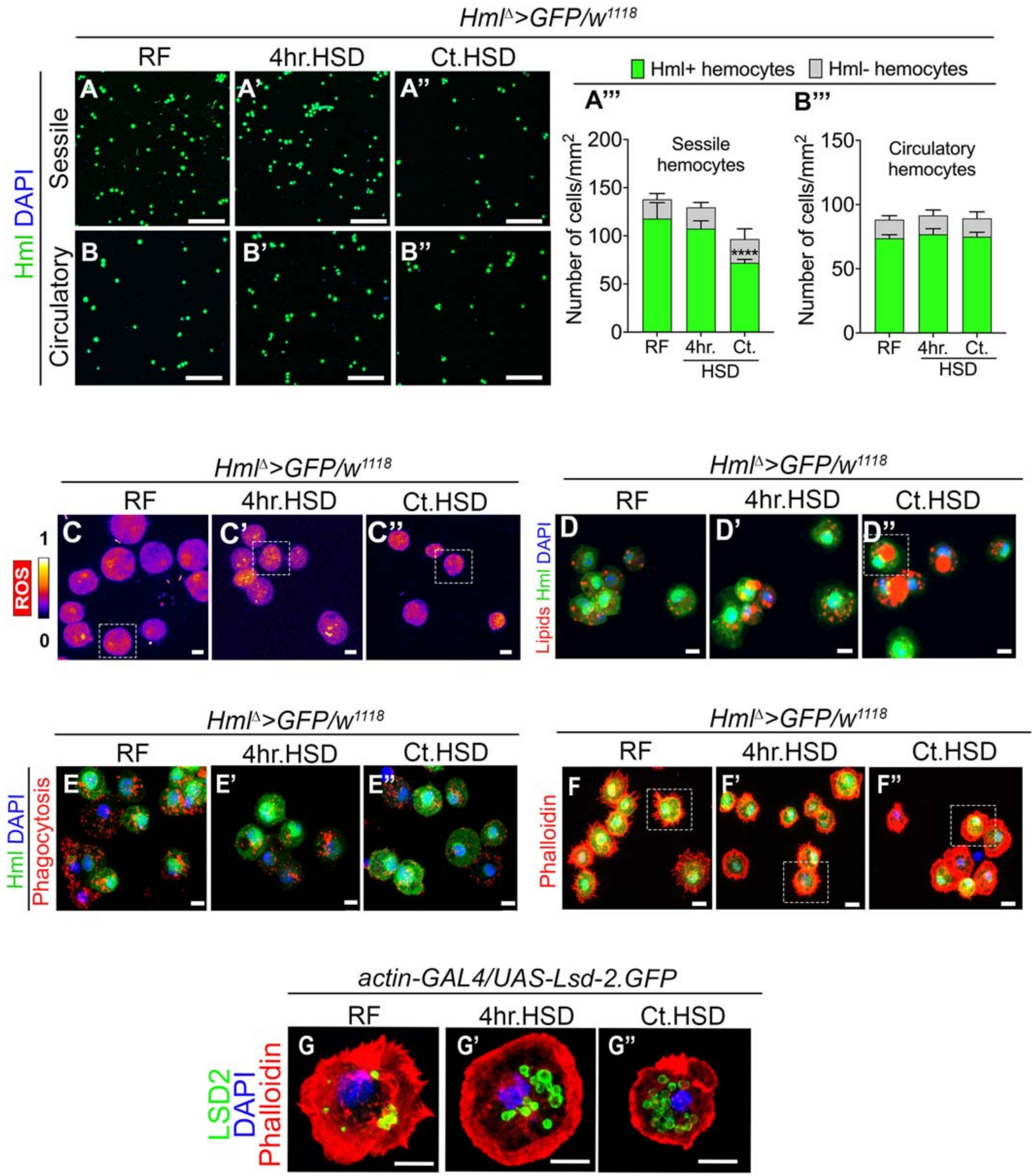
HmlΔ>GFP/w1118
RF
4hr.HSD
Ct.HSD
Hml DAPI
Sessile
Circulatory
A
A'
A''
B
B'
B''
Hml+ hemocytes
Hml- hemocytes
A'''
B'''
Sessile hemocytes
Circulatory hemocytes
Number of cells/mm2
****
4hr.
Ct.
HSD
ROS
C
C'
C''
Lipids Hml DAPI
D
D'
D''
Hml DAPI Phagocytosis
E
E'
E''
Phalloidin
F
F'
F''
actin-GAL4/UAS-Lsd-2.GFP
LSD2 DAPI Phalloidin
G
G'
G''

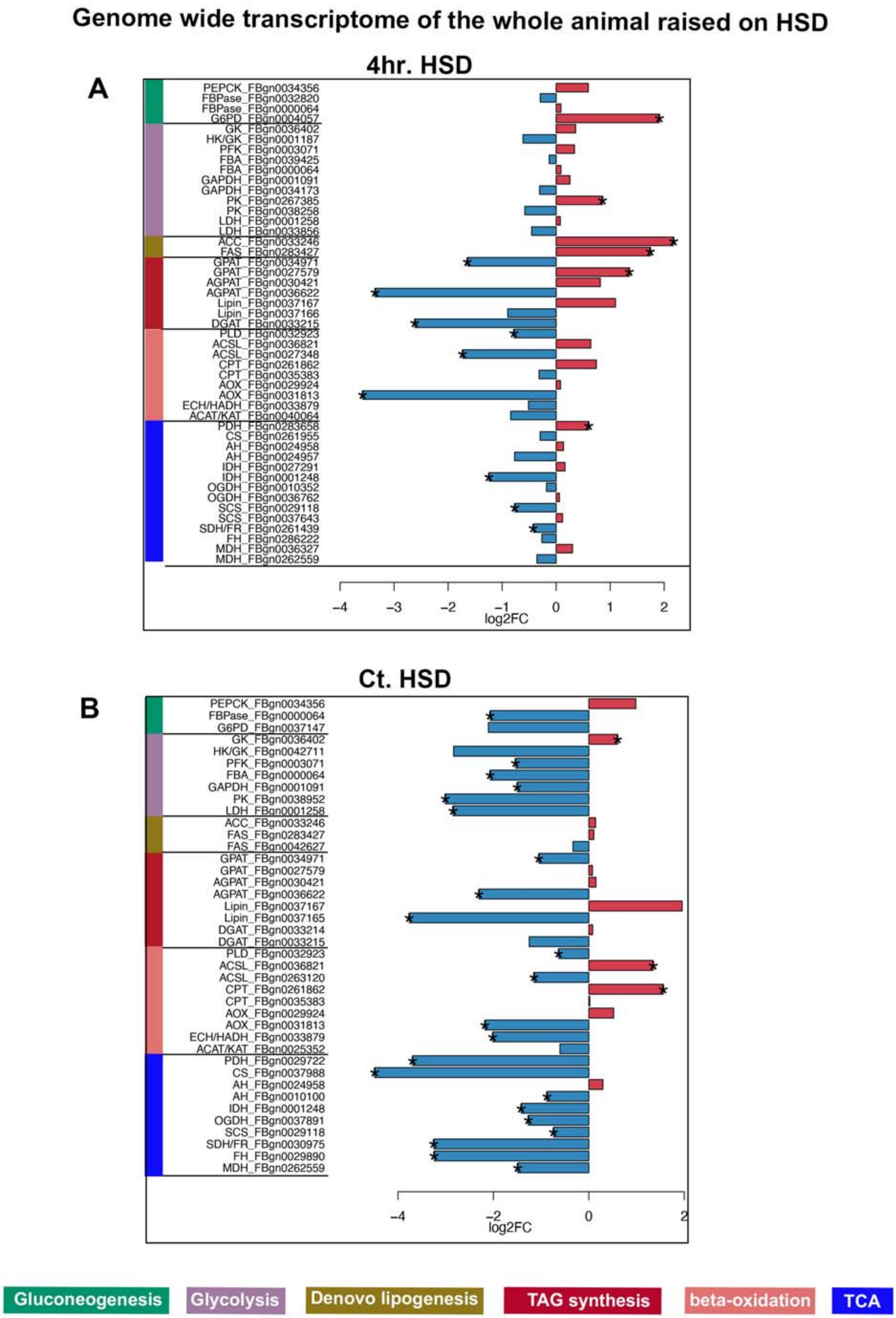
Genome wide transcriptome of the whole animal raised on HSD
A
4hr. HSD
PEPCK_FBgn0034356
FBPase_FBgn0032820
FBPase_FBgn0000064
G6PD_FBgn0004057
GK_FBgn0036402
HK/GK_FBgn0001187
PFK_FBgn0003071
FBA_FBgn0039425
FBA_FBgn0000064
GAPDH_FBgn0001091
GAPDH_FBgn0034173
PK_FBgn0267385
PK_FBgn0038258
LDH_FBgn0001258
LDH_FBgn0033856
ACC_FBgn0033246
FAS_FBgn0283427
GPAT_FBgn0034971
GPAT_FBgn0027579
AGPAT_FBgn0030421
AGPAT_FBgn0036622
Lipin_FBgn0037167
Lipin_FBgn0037166
DGAT_FBgn0033215
PLD_FBgn0032923
ACSL_FBgn0036821
ACSL_FBgn0027348
CPT_FBgn0261862
CPT_FBgn0035383
AOX_FBgn0029924
AOX_FBgn0031813
ECH/HADH_FBgn0033879
ACAT/KAT_FBgn0040064
PDH_FBgn0283658
CS_FBgn0261955
AH_FBgn0024958
AH_FBgn0024957
IDH_FBgn0027291
IDH_FBgn0001248
OGDH_FBgn0010352
OGDH_FBgn0036762
SCS_FBgn0029118
SCS_FBgn0037643
SDH/FR_FBgn0261439
FH_FBgn0286222
MDH_FBgn0036327
MDH_FBgn0262559
−4 −3 −2 −1 0 1 2
log2FC
B
Ct. HSD
PEPCK_FBgn0034356
FBPase_FBgn0000064
G6PD_FBgn0037147
GK_FBgn0036402
HK/GK_FBgn0042711
PFK_FBgn0003071
FBA_FBgn0000064
GAPDH_FBgn0001091
PK_FBgn0038952
LDH_FBgn0001258
ACC_FBgn0033246
FAS_FBgn0283427
FAS_FBgn0042627
GPAT_FBgn0034971
GPAT_FBgn0027579
AGPAT_FBgn0030421
AGPAT_FBgn0036622
Lipin_FBgn0037167
Lipin_FBgn0037165
DGAT_FBgn0033214
DGAT_FBgn0033215
PLD_FBgn0032923
ACSL_FBgn0036821
ACSL_FBgn0263120
CPT_FBgn0261862
CPT_FBgn0035383
AOX_FBgn0029924
AOX_FBgn0031813
ECH/HADH_FBgn0033879
ACAT/KAT_FBgn0025352
PDH_FBgn0029722
CS_FBgn0037988
AH_FBgn0024958
AH_FBgn0010100
IDH_FBgn0001248
OGDH_FBgn0037891
SCS_FBgn0029118
SDH/FR_FBgn0030975
FH_FBgn0029890
MDH_FBgn0262559
−4 −2 0 2
log2FC
Gluconeogenesis
Glycolysis
Denovo lipogenesis
TAG synthesis
beta-oxidation
TCA

 © The Author(s)

Figure EV2. High sugar diet dampens metabolic events in whole larvae.

(**A**, **B**) Bar plots of upregulated (in red) and downregulated genes (in blue) of different metabolic pathways in whole animal (larvae) raised on 4 hr.HSD and Ct.HSD, respectively. Metabolic genes are downregulated in 4 hr.HSD whole larvae and this is sustained in long-term Ct.HSD animals. Source data are available online for this figure.

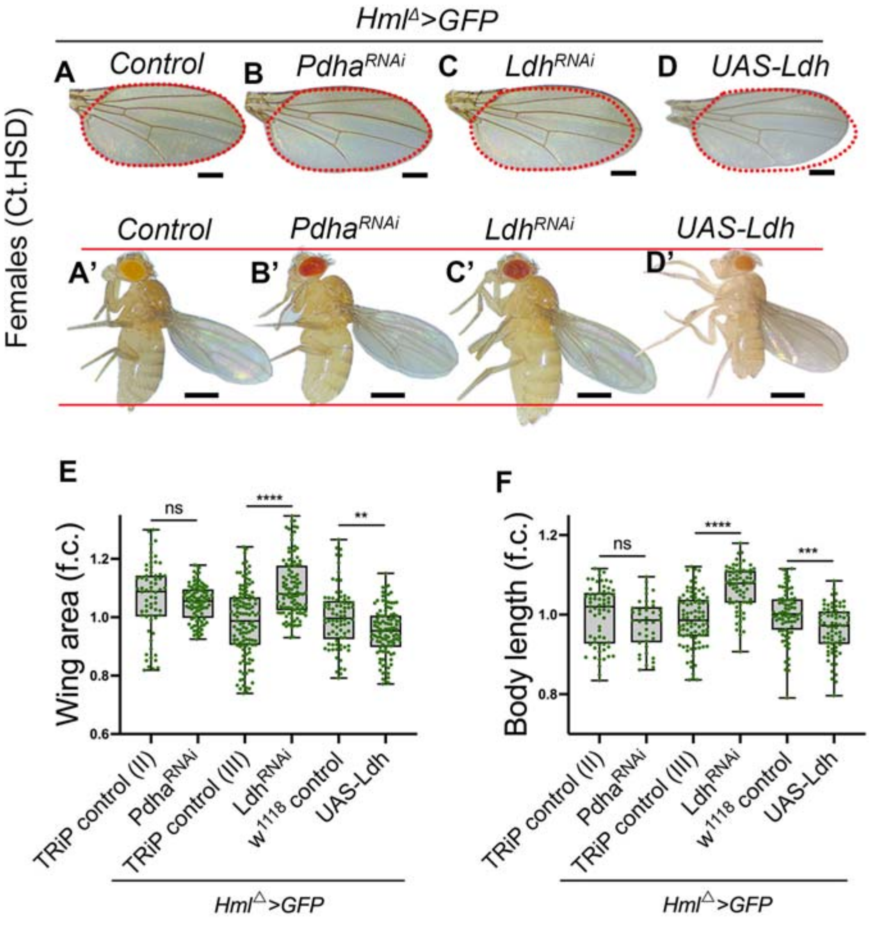

Figure EV3. Glycolytic state in immune cells represses growth on HSD.

(**A–D′**) Modulating larval immune cell TCA and glycolytic activity to show adult size change. Representative images of wings (**A–D**) and adult females (**A′–D′**) showing size phenotype on Ct.HSD from respective genetic backgrounds. Compared to (**A**, **A′**) Ct.HSD Control (*Hml^{Δ}>GFP/w^{1118}*), (**B**, **B′**) expressing *Pdha^{RNAi}* (*Hml^{Δ}>GFP/Pdha^{RNAi}*) in immune cells to reduce TCA activity did not show any size change. (**C**, **C′**) Downregulating immune cell glycolytic activity by expressing *Ldh^{RNAi}* (*Hml^{Δ}>GFP/Ldh^{RNAi}*) causes increase in size. (**D**, **D′**) Overexpression of Ldh (*Hml^{Δ}>GFP/UAS-Ldh*) showed size reduction. (**E**) Quantification of wing area in *Hml^{Δ}>GFP/Pdha^{RNAi}* (Ct.HSD, $N = 3$, $n = 91$, $P = 0.1102$) in comparison to *Hml^{Δ}>GFP/TRiP (II) control* (Ct.HSD, $N = 3$, $n = 64$), *Hml^{Δ}>GFP/Ldh^{RNAi}* (Ct.HSD, $N = 3$, $n = 106$, $P < 0.0001$) in comparison to *Hml^{Δ}>GFP/TRiP (III) control* (Ct.HSD, $N = 3$, $n = 121$) and *Hml^{Δ}>GFP/UAS-Ldh* (Ct.HSD, $N = 3$, $n = 104$, $P = 0.0015$) in comparison to *Hml^{Δ}>GFP/w^{1118}* control (Ct.HSD, $N = 3$, $n = 81$). (**F**) Quantification of body length in *Hml^{Δ}>GFP/Pdha^{RNAi}* (Ct.HSD, $N = 3$, $n = 40$, $P = 0.0565$) in comparison to *Hml^{Δ}>GFP/TRiP (II) control* (Ct.HSD, $N = 3$, $n = 65$) and *Hml^{Δ}>GFP/Ldh^{RNAi}* (Ct.HSD, $N = 3$, $n = 70$, $P < 0.0001$) in comparison to *Hml^{Δ}>GFP/TRiP (III) control* (Ct.HSD, $N = 3$, $n = 89$) and *Hml^{Δ}>GFP/UAS-Ldh* (Ct.HSD, $N = 3$, $n = 73$, $P = 0.0003$) in comparison to *Hml^{Δ}>GFP/w^{1118} control* (Ct.HSD, $N = 3$, $n = 75$). Data information: Scale bar: 0.5 mm for flies and 0.25 mm for wings. In quantification graphs, shown in panel (**E**, **F**) each dot represents an animal. Comparison for significance is with respect to respective background control on Ct.HSD. Asterisks mark statistically significant differences (*$P < 0.05$; **$P < 0.01$; ***$P < 0.001$; ****$P < 0.0001$). The statistical analysis applied for (**E**, **F**) is Mann–Whitney test. *N* indicates the number of independent biological replicates, and *n* refers to the total number of animals analyzed. Only right wing from each adult fly was selected for quantification. The differences in wing areas or fly body lengths in panels is indicated with a red dotted line or two horizontal red lines that highlight changes across genotypes. RF and Ct.HSD correspond to regular food and constitutive high sugar diet respectively. Box plots show the median (center line), 25th–75th percentiles (bounds of box), and whiskers extending to the minimum and maximum values; all individual data points are shown. Source data are available online for this figure.

© The Author(s)

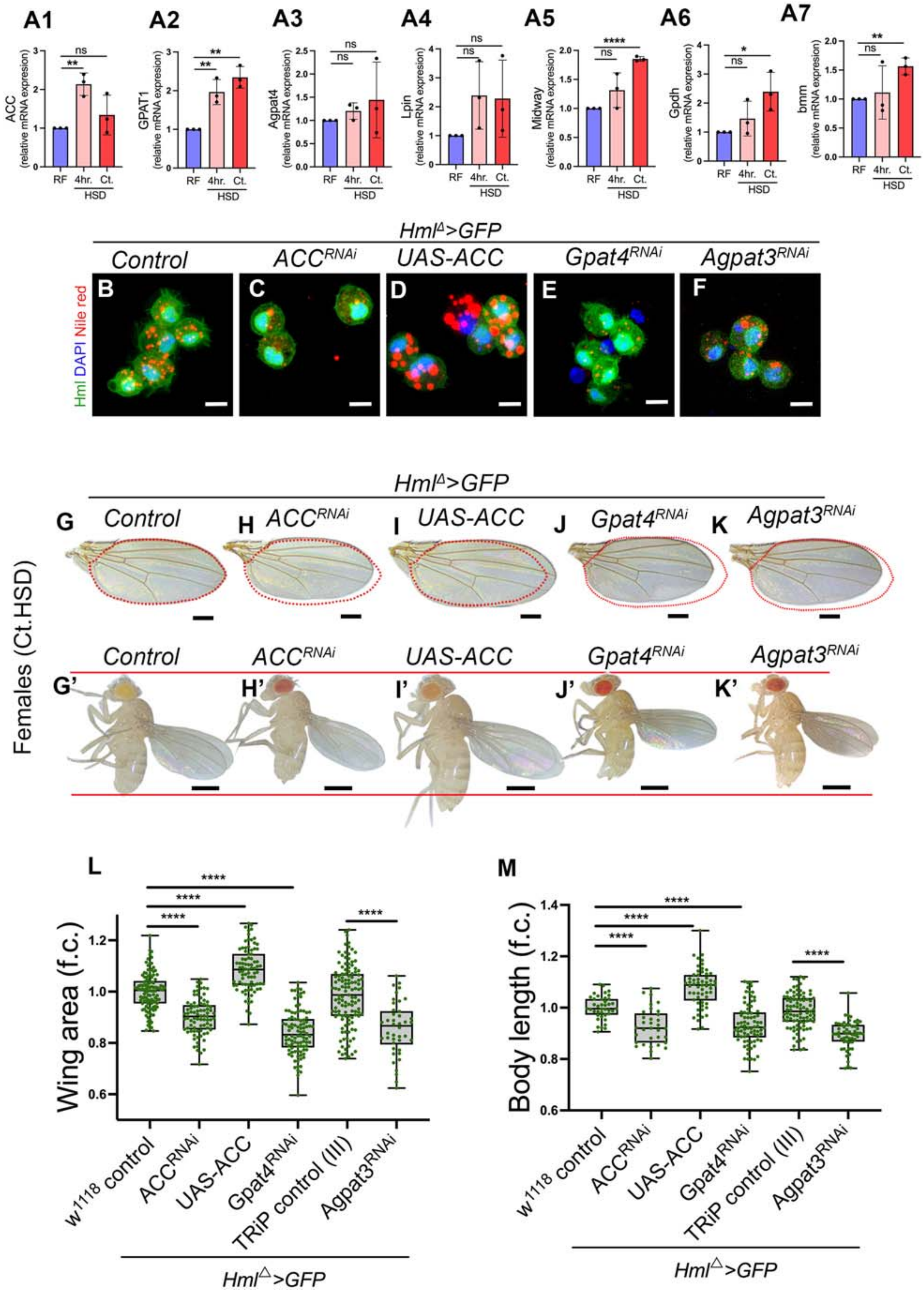
A1
A2
A3
A4
A5
A6
A7
ACC (relative mRNA expresion)
GPAT1 (relative mRNA expresion)
Agpat4 (relative mRNA expresion)
Lpin (relative mRNA expresion)
Midway (relative mRNA expresion)
Gpdh (relative mRNA expresion)
bmm (relative mRNA expresion)
RF 4hr. Ct.
HSD
ns
**
****
*
Hml$^{\Delta}$>GFP
Control
ACC^{RNAi}
UAS-ACC
Gpat4^{RNAi}
Agpat3^{RNAi}
B
C
D
E
F
Hml DAPI Nile red
G
H
I
J
K
G'
H'
I'
J'
K'
Females (Ct.HSD)
L
M
Wing area (f.c.)
Body length (f.c.)
w^{1118} control
TRiP control (III)

Figure EV4. Immune cell lipid homeostasis and systemic growth regulation on HSD.

(**A1–A7**) Relative expression of immune-specific lipid metabolism genes in *Hml*$^{\Delta}$*>GFP/w*^{1118} larvae exposed to 4 hr.HSD and Ct.HSD, measured by RT-PCR. (**A1**) ACC show a significant upregulation at 4 hr.HSD ($N = 3$, $n = 105$, $P = 0.0025$) but not at Ct.HSD ($N = 3$, $n = 120$, $P = 0.3125$). (**A2**) GPAT1 was upregulated at 4 hr.HSD ($N = 3$, $n = 120$, $P = 0.0066$) and Ct.HSD ($N = 3$, $n = 120$, $P = 0.0011$) as well. (**A3**) Agpat4 and (**A4**) Lpin did not show any change at 4 hr.HSD and Ct.HSD. (**A5**) Midway showed no change at 4 hr.HSD ($N = 3$, $n = 120$, $P = 0.1424$) however showed upregulation at Ct.HSD ($N = 3$, $n = 120$, $P < 0.0001$). (**A6**) Gpdh1 showed no change at 4 hr.HSD ($N = 3$, $n = 120$, $P = 0.2473$) however it was upregulated in Ct.HSD ($N = 3$, $n = 120$, $P = 0.0223$). (**A7**) bmm show upregulation at Ct.HSD ($N = 3$, $n = 120$, $P = 0.0025$) and no change at 4 hr.HSD ($N = 3$, $n = 120$, $P = 0.6920$). All comparisons are with control *Hml*$^{\Delta}$*GFP > /w*^{1118} on RF ($N = 3$, $n = 105$). (**B–F**) Representative images of immune cells stained with Nile Red (red) to visualize lipid droplets in respective genotypes under Ct.HSD. (**B**) Ct.HSD Control. (**C**) Immune-specific knockdown of ACC (*Hml*$^{\Delta}$*>GFP/ACC*^{RNAi}) reduces de novo lipid synthesis and results in fewer lipid droplets compared to control. (**D**) Immune-specific overexpression of ACC (*Hml*$^{\Delta}$*>GFP/UAS-ACC*) increases de novo lipid synthesis and shows more lipid droplets. (**E**, **F**) Knockdown of Gpat4 (*Hml*$^{\Delta}$*>GFP/Gpat4*^{RNAi}) or Agpat3 (*Hml*$^{\Delta}$*>GFP/Agpat3*^{RNAi}) reduces TAG synthesis and leads to fewer lipid droplets compared to control. (**G–K′**) Modulating larval immune cell lipid homeostasis affects adult growth. Representative images of wings (**G–K**) and adult females (**G′–K′**) showing size phenotype on Ct.HSD from respective genetic backgrounds. Compared to (**G**, **G′**) Ct.HSD Control (*Hml*$^{\Delta}$*>GFP/w*^{1118}), (**H**, **H′**) loss of ACC function (*Hml*$^{\Delta}$*>GFP/ACC*^{RNAi}) leads to growth retardation while (**I**, **I′**) ACC gain of function (*Hml*$^{\Delta}$*>GFP/UAS-ACC*) shows growth recovery and the flies are much larger than Ct.HSD Control adults (**G**, **G′**). Similarly, loss of TAG synthesis, by blocking (**I**, **I′**) Gpat4 (*Hml*$^{\Delta}$*>GFP/Gpat4*^{RNAi}) or (**K**, **K′**) Agpat3 (*Hml*$^{\Delta}$*>GFP/Agpat3*^{RNAi}) shows reduction in animal size. (**L**) Quantification of wing area in *Hml*$^{\Delta}$*>GFP/ACC*^{RNAi} ($N = 3$, $n = 81$, $P < 0.0001$), *Hml*$^{\Delta}$*>GFP/UAS-ACC* ($N = 3$, $n = 83$, $P < 0.0001$), *Hml*$^{\Delta}$*>GFP/Gpat4*^{RNAi} ($N = 3$, $n = 96$, $P < 0.0001$) in comparison to Ct.HSD *control*, *Hml*$^{\Delta}$*>GFP/w*^{1118}, $N = 3$, $n = 115$). *Hml*$^{\Delta}$*>GFP/Agpat3*^{RNAi} ($N = 3$, $n = 42$, $P < 0.0001$ in comparison to *Hml*$^{\Delta}$*>GFP/TRiP (III) control* (Ct.HSD, $N = 3$, $n = 121$). (**M**) Quantification of body length in *Hml*$^{\Delta}$*>GFP/ACC*^{RNAi} ($N = 3$, $n = 33$, $P < 0.0001$), *Hml*$^{\Delta}$*>GFP/UAS-ACC* ($N = 3$, $n = 58$, $P < 0.0001$), *Hml*$^{\Delta}$*>GFP/Gpat4*^{RNAi} ($N = 3$, $n = 78$, $P < 0.0001$) in comparison to Ct.HSD Control, *Hml*$^{\Delta}$*>GFP/w*^{1118}, $N = 3$, $n = 40$). *Hml*$^{\Delta}$*>GFP/Agpat3*^{RNAi} ($N = 3$, $n = 56$, $P < 0.0001$ in comparison to *Hml*$^{\Delta}$*>GFP/TRiP (III) control* (Ct.HSD, $N = 3$, $n = 89$). Data information: DNA is stained with DAPI (blue), immune cells are shown in green (*Hml*$^{\Delta}$*>UAS-GFP*). Nile red (red) staining to mark lipids in (**B–F**). Scale bar: 5 µm for immune cells, 0.5 mm for flies and 0.25 mm for wings. In quantification graphs (**A1–A7**) each dot represents an experimental repeat and in graphs (**L**, **M**) each dot represents an animal. Except for panel (**A1–A7**) where comparisons are with respect to Control on RF, in all other panels comparison for significance is with respect to respective background control on Ct.HSD. Asterisks mark statistically significant differences (*$P < 0.05$; **$P < 0.01$; ***$P < 0.001$; ****$P < 0.0001$). The statistical analysis applied for (**A1–A7**) is unpaired *t* test, for other panels (**L**, **M**) Mann– Whitney test. *N* indicates the number of independent biological replicates, and n refers to the total number of animals analyzed. Only right wing from each adult fly was selected for quantification. The differences in wing areas or fly body lengths in panels is indicated with a red dotted line or two horizontal red lines that highlight changes across genotypes. RF, 4 hr.HSD and Ct.HSD correspond to regular food, 4 h high sugar diet and constitutive high sugar diet respectively. See methods for further details on larval numbers and sample analysis for each of the experiments. In bar graphs data are presented as mean ± SD. Box plots show the median (center line), 25th–75th percentiles (bounds of box), and whiskers extending to the minimum and maximum values; all individual data points are shown. Source data are available online for this figure.

 © The Author(s)

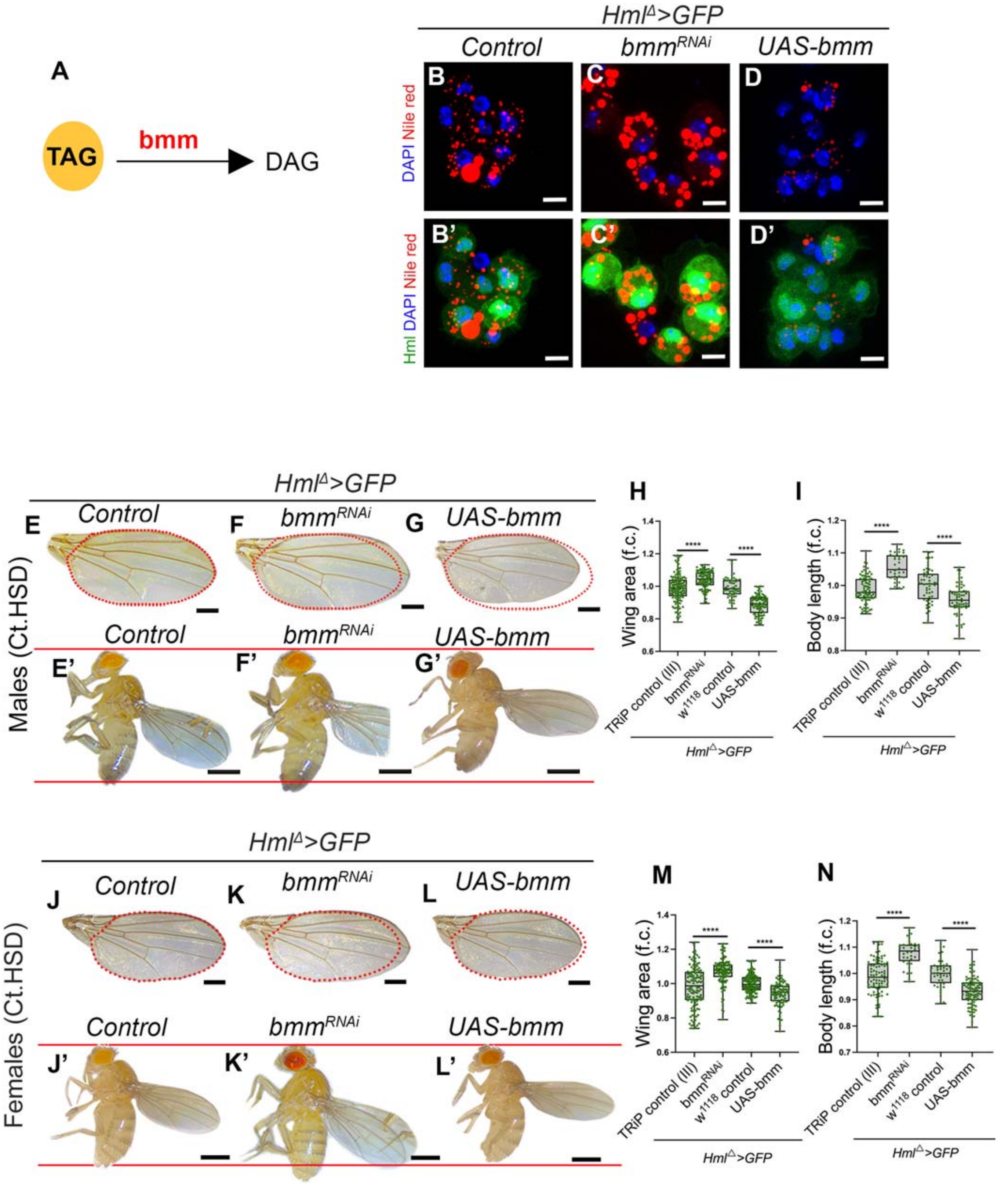
A
TAG
bmm
DAG
Hml$^{\Delta}$>GFP
Control
bmm^{RNAi}
UAS-bmm
B
C
D
DAPI Nile red
B'
C'
D'
Hml DAPI Nile red
Hml$^{\Delta}$>GFP
Control
bmm^{RNAi}
UAS-bmm
E
F
G
Males (Ct.HSD)
E'
F'
G'
H
I
Wing area (f.c.)
Body length (f.c.)
TRiP control (III)
bmm^{RNAi}
w^{1118} control
UAS-bmm
Hml$^{\Delta}$>GFP
J
K
L
Females (Ct.HSD)
J'
K'
L'
M
N

Figure EV5. Immune cell lipolytic state as inhibitor of systemic growth on HSD.

(**A**) Schematic shows bmm is a lipase enzyme which breaks down triacylglycerol (TAG) into diacylglycerol (DAG). (**B–D′**) Representative images of immune cells from Ct.HSD larvae stained with Nile Red (red) to visualize lipid droplets. (**B**, **B′**) Ct.HSD Control (*Hml*$^{\Delta}$*>GFP/w*^{1118}). (**C**, **C′**) Immune-specific knockdown of bmm (*Hml*$^{\Delta}$*>GFP/bmm*^{RNAi}) increases lipid droplet accumulation compared to control. (**D**, **D′**) Immune-specific overexpression of bmm (*Hml*$^{\Delta}$*>GFP/UAS-bmm*) reduces lipid droplet levels compared to control. (**E–G′**) Modulating larval immune cell lipolysis affects adult growth. Representative images of wings and flies of adult males (**E–G′**) showing size phenotype on Ct.HSD from respective genetic perturbations. Compared to (**E**, **E′**) Ct.HSD Control (*Hml*$^{\Delta}$*>GFP/w*^{1118}), (**F**, **F′**) loss of bmm (*Hml*$^{\Delta}$*>GFP/bmm*^{RNAi}) resulted in recovery in adult fly size while (**G**, **G′**) increase in bmm expression (*Hml*$^{\Delta}$*>GFP/UAS-bmm*) in immune cells caused a further reduction in size. (**H**) Quantification of wing area in (**H**) *Hml*$^{\Delta}$*>GFP/bmm*^{RNAi} ($N = 3$, $n = 95$, $P < 0.0001$) in comparison to *Hml*$^{\Delta}$*>GFP/TRiP (III) control* (Ct.HSD, $N = 3$, $n = 131$). *Hml*$^{\Delta}$*>GFP/UAS- bmm* ($N = 3$, $n = 89$, $P < 0.0001$ in comparison to Ct.HSD Control, *Hml*$^{\Delta}$*>GFP/w*^{1118}, $N = 3$, $n = 54$). (**I**) Quantification of body length in (**H**) *Hml*$^{\Delta}$*>GFP/bmm*^{RNAi} ($N = 3$, $n = 32$, $P < 0.0001$) in comparison to *Hml*$^{\Delta}$*>GFP/TRiP (III) control* (Ct.HSD, $N = 3$, $n = 82$). *Hml*$^{\Delta}$*>GFP/UAS-bmm* ($N = 3$, $n = 67$, $P < 0.0001$ in comparison to Ct.HSD Control, *Hml*$^{\Delta}$*>GFP/w*^{1118}, $N = 3$, $n = 52$). (**J–L**) Representative images of fly wings and (**J′–L′**) adult females showing size phenotype on Ct.HSD from respective genetic backgrounds. Compared to (**J**, **J′**) Ct.HSD Control (*Hml*$^{\Delta}$*>GFP/w*^{1118}), (**K**, **K′**) knockdown of bmm (*Hml*$^{\Delta}$*>GFP/bmm*^{RNAi}) or (**L**, **L′**) increase in its expression (*Hml*$^{\Delta}$*>GFP/UAS-bmm*) in immune cells causes either a recovery in adult fly size or a further reduction in size, respectively. (**M**) Quantification of female wing area in *Hml*$^{\Delta}$*>GFP/bmm*^{RNAi} ($N = 3$, $n = 108$, $P < 0.0001$) in comparison to *Hml*$^{\Delta}$*>GFP/TRiP (II) control* (Ct.HSD, $N = 3$, $n = 121$), and *Hml*$^{\Delta}$*>GFP/UAS-bmm* ($N = 3$, $n = 85$, $P < 0.0001$) in comparison to HSD *Control*, (*Hml*$^{\Delta}$*>GFP/w*^{1118}, $N = 3$, $n = 132$). (**N**) Quantification of female body length in *Hml*$^{\Delta}$*>GFP/bmm*^{RNAi} ($N = 3$, $n = 37$, $P < 0.0001$) in comparison to *Hml*$^{\Delta}$*>GFP/TRiP (III) control* (Ct.HSD, $N = 3$, $n = 89$), and *Hml*$^{\Delta}$*>GFP/UAS-bmm* ($N = 3$, $n = 93$, $P < 0.0001$) in comparison to HSD *Control*, (*Hml*$^{\Delta}$*>GFP/w*^{1118}, $N = 3$, $n = 47$). Data information: DNA is stained with DAPI (blue), immune cells are shown in green (*Hml*$^{\Delta}$*>UAS-GFP*). Nile red (red) staining to mark lipids in (**B–D′**). Scale bar: 5 µm for immune cells, 0.5 mm for flies and 0.25 mm for wings. In quantification (**H**, **I**, **M**, **N**), each dot represents an animal. Comparison for significance is with respective background control on HSD. Asterisks mark statistically significant differences (**P* < 0.05; ***P* < 0.01; ****P* < 0.001; *****P* < 0.0001). The statistical analysis applied for (**H**, **I**, **M**, **N**) is Mann–Whitney test. *N* indicates the number of independent biological replicates, and n refers to the total number of animals analyzed. Only right wing from each adult fly was selected for quantification. The differences in wing areas or fly body lengths in panels is indicated with a red dotted line or two horizontal red lines that highlight changes across genotypes. RF and Ct.HSD correspond to regular food and constitutive high sugar diet, respectively. Box plots show the median (center line), 25th–75th percentiles (bounds of box), and whiskers extending to the minimum and maximum values; all individual data points are shown. Source data are available online for this figure.

 © The Author(s)

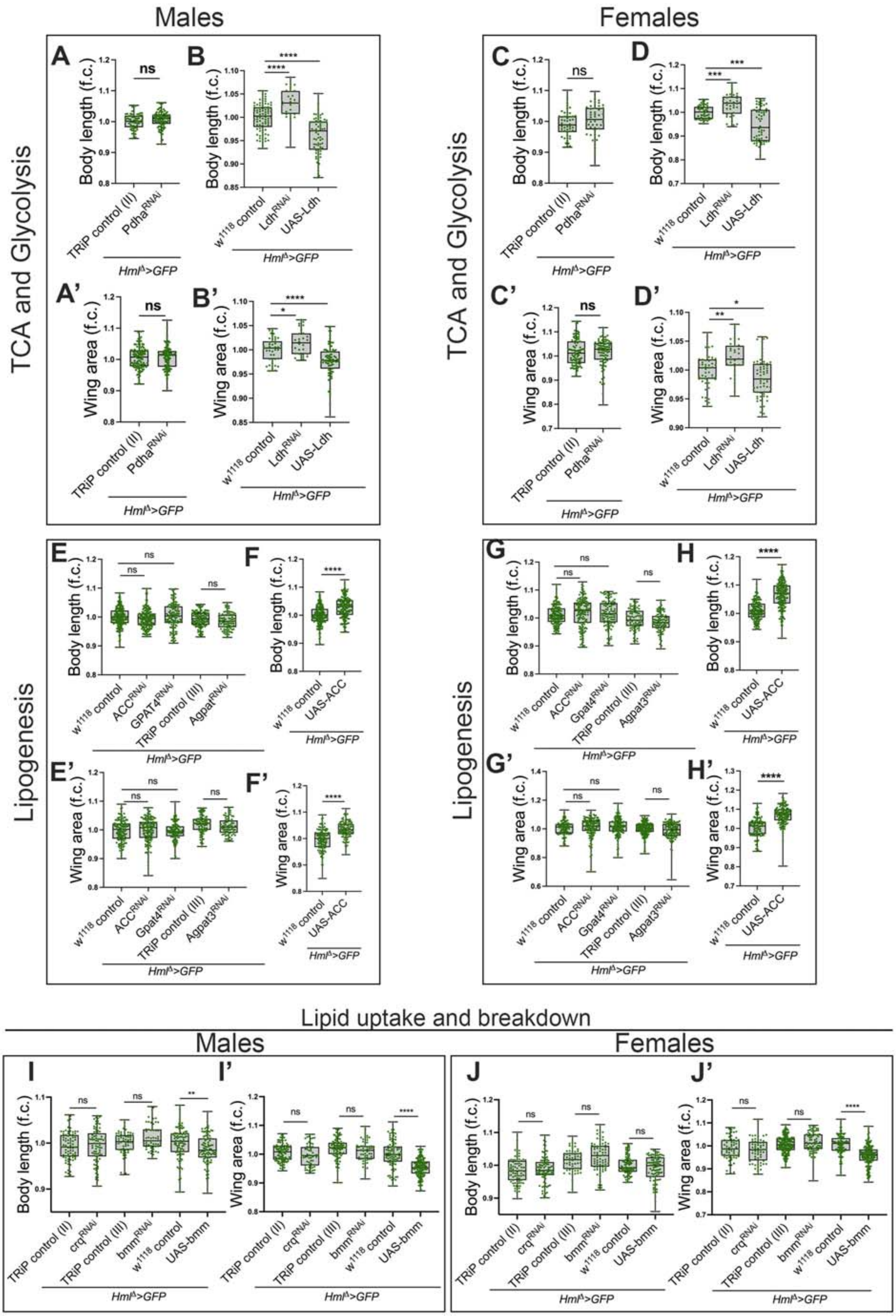
Males
Females
TCA and Glycolysis
A
B
C
D
A'
B'
C'
D'
Body length (f.c.)
Wing area (f.c.)
ns
TRiP control (II)
PdhaRNAi
w1118 control
LdhRNAi
UAS-Ldh
HmlΔ>GFP
Lipogenesis
E
F
G
H
E'
F'
G'
H'
ACCRNAi
GPAT4RNAi
Gpat4RNAi
TRiP control (III)
AgpatRNAi
Agpat3RNAi
UAS-ACC
Lipid uptake and breakdown
I
I'
J
J'
crqRNAi
bmmRNAi
UAS-bmm

Figure EV6. Control of homeostatic growth by macrophage metabolic state changes.

(**A**) Quantification of body length (RF, males) in *Hml*$^{\Delta}$*>GFP/Pdha*^{RNAi} ($N = 3$, $n = 87$, $P = 0.0957$) in comparison to *Hml*$^{\Delta}$*>GFP/TRiP (II) control* ($N = 3$, $n = 62$). (**A′**) Quantification of wing area (RF, males) in *Hml*$^{\Delta}$*>GFP/Pdha*^{RNAi} ($N = 3$, $n = 108$, $P = 0.8986$) in comparison to *Hml*$^{\Delta}$*>GFP/TRiP (II) control* ($N = 3$, $n = 108$). (**B**) Quantification of body length (RF, males) in *Hml*$^{\Delta}$*>GFP/Ldh*^{RNAi} ($N = 3$, $n = 29$, $P < 0.0001$) and *Hml*$^{\Delta}$*>GFP/UAS-Ldh* ($N = 3$, $n = 73$, $P < 0.0001$) in comparison to *Hml*$^{\Delta}$*>GFP/w*^{1118} *control* ($N = 3$, $n = 81$). (**B′**) Quantification of wing area (RF, males) in *Hml*$^{\Delta}$*>GFP/Ldh*^{RNAi} ($N = 3$, $n = 28$, $P = 0.0413$) and *Hml*$^{\Delta}$*>GFP/UAS-Ldh* ($N = 3$, $n = 81$, $P < 0.0001$) in comparison to *Hml*$^{\Delta}$*>GFP/w*^{1118} *control* ($N = 3$, $n = 47$). (**C**) Quantification of body length (RF, females) in *Hml*$^{\Delta}$*>GFP/Pdha*^{RNAi} ($N = 3$, $n = 45$, $P = 0.0895$) in comparison to *Hml*$^{\Delta}$*>GFP/TRiP (II) control* ($N = 3$, $n = 52$). (**C′**) Quantification of wing area (RF, females) in *Hml*$^{\Delta}$*>GFP/Pdha*^{RNAi} ($N = 3$, $n = 82$, $P = 0.4170$) in comparison to *Hml*$^{\Delta}$*>GFP/TRiP (II) control* ($N = 3$, $n = 92$). (**D**) Quantification of body length (RF, females) in *Hml*$^{\Delta}$*>GFP/Ldh*^{RNAi} ($N = 3$, $n = 38$, $P = 0.0004$) and *Hml*$^{\Delta}$*>GFP/UAS-Ldh* ($N = 3$, $n = 58$, $P = 0.0005$) in comparison to *Hml*$^{\Delta}$*>GFP/w*^{1118} *control* ($N = 3$, $n = 51$). (**D′**) Quantification of wing area (RF, females) in *Hml*$^{\Delta}$*>GFP/Ldh*^{RNAi} ($N = 3$, $n = 34$, $P = 0.0014$) *Hml*$^{\Delta}$*>GFP/UAS-Ldh* ($N = 3$, $n = 64$, $P = 0.0188$) in comparison to *Hml*$^{\Delta}$*>GFP/w*^{1118} *control* ($N = 3$, $n = 52$). (**E**) Quantification of body length (RF, males) in *Hml*$^{\Delta}$*>GFP/ACC*^{RNAi} ($N = 3$, $n = 165$, $P = 0.0681$), *Hml*$^{\Delta}$*>GFP/Gpat4*^{RNAi} ($N = 3$, $n = 119$, $P = 0.4515$) in comparison to RF control, *Hml*$^{\Delta}$*>GFP/w*^{1118} ($N = 3$, $n = 188$). *Hml*$^{\Delta}$*>GFP/Agpat3*^{RNAi} ($N = 3$, $n = 88$, $P = 0.0535$ in comparison to *Hml*$^{\Delta}$*>GFP/TRiP (III) control* ($N = 3$, $n = 116$). (**E′**) Quantification of wing area (RF, males) in *Hml*$^{\Delta}$*>GFP/ACC*^{RNAi} ($N = 3$, $n = 131$, $P = 0.4355$), *Hml*$^{\Delta}$*>GFP/Gpat4*^{RNAi} ($N = 3$, $n = 114$, $P = 0.4514$) in comparison to RF control, *Hml*$^{\Delta}$*>GFP/w*^{1118} ($N = 3$, $n = 126$). *Hml*$^{\Delta}$*>GFP/Agpat3*^{RNAi} ($N = 3$, $n = 73$, $P = 0.053$) in comparison to *Hml*$^{\Delta}$*>GFP/TRiP (III) control* ($N = 3$, $n = 91$). (**F**) Quantification of body length (RF, males) in *Hml*$^{\Delta}$*>GFP/UAS-ACC* ($N = 3$, $n = 176$, $P < 0.0001$) in comparison to RF *control*, *Hml*$^{\Delta}$*>GFP/w*^{1118} ($N = 3$, $n = 189$). (**F′**) Quantification of wing area (RF, males) in *Hml*$^{\Delta}$*>GFP/UAS-ACC* ($N = 3$, $n = 109$, $P < 0.0001$) in comparison to RF control, *Hml*$^{\Delta}$*>GFP/w*^{1118} ($N = 3$, $n = 127$). (**G**) Quantification of body length (RF, females) in *Hml*$^{\Delta}$*>GFP/ACC*^{RNAi} ($N = 3$, $n = 141$, $P = 0.0549$) *Hml*$^{\Delta}$*>GFP/Gpat4*^{RNAi} ($N = 3$, $n = 108$, $P = 0.1340$) in comparison to RF control, *Hml*$^{\Delta}$*>GFP/w*^{1118} ($N = 3$, $n = 152$). *Hml*$^{\Delta}$*>GFP/Agpat3*^{RNAi} ($N = 3$, $n = 90$, $P = 0.0555$ in comparison to *Hml*$^{\Delta}$*>GFP/TRiP (III) control* ($N = 3$, $n = 88$). (**G′**) Quantification of wing area (RF, females) in *Hml*$^{\Delta}$*>GFP/ACC*^{RNAi} ($N = 3$, $n = 119$, $P = 0.0536$), *Hml*$^{\Delta}$*>GFP/Gpat4*^{RNAi} ($N = 3$, $n = 132$, $P = 0.2863$) in comparison to RF control, *Hml*$^{\Delta}$*>GFP/w*^{1118} ($N = 3$, $n = 98$). *Hml*$^{\Delta}$*>GFP/Agpat3*^{RNAi} ($N = 3$, $n = 102$, $P = 0.1034$) in comparison to *Hml*$^{\Delta}$*>GFP/TRiP (III) control* ($N = 3$, $n = 174$). (**H**) Quantification of body length (RF, females) in *Hml*$^{\Delta}$*>GFP/UAS-ACC* ($N = 3$, $n = 189$, $P < 0.0001$) in comparison to RF control, *Hml*$^{\Delta}$*>GFP/w*^{1118} ($N = 3$, $n = 152$). (**H′**) Quantification of wing area (RF, females) in *Hml*$^{\Delta}$*>GFP/UAS-ACC* ($N = 3$, $n = 126$, $P < 0.0001$) in comparison to RF *control*, *Hml*$^{\Delta}$*>GFP/w*^{1118} ($N = 3$, $n = 98$). (**I**) Quantification of body length (RF, males) in *Hml*$^{\Delta}$*>GFP/crq*^{RNAi} ($N = 3$, $n = 79$, $P = 0.6827$) in comparison to *Hml*$^{\Delta}$*>GFP/TRiP (II) control* (Ct.HSD, $N = 3$, $n = 78$). *Hml*$^{\Delta}$*>GFP/bmm*^{RNAi} ($N = 3$, $n = 54$, $P = 0.1471$) in comparison to *Hml*$^{\Delta}$*>GFP/TRiP (III) control* (Ct.HSD, $N = 3$, $n = 60$). *Hml*$^{\Delta}$*>GFP/UAS-bmm* ($N = 3$, $n = 90$, $P = 0.0039$) in comparison to *Hml*$^{\Delta}$*>GFP/w*^{1118} control (Ct.HSD, $N = 3$, $n = 90$). (**I′**) Quantification of wing area (RF, males) in *Hml*$^{\Delta}$*>GFP/crq*^{RNAi} ($N = 3$, $n = 56$, $P = 0.0852$) in comparison to *Hml*$^{\Delta}$*>GFP/TRiP (II) control* (Ct.HSD, $N = 3$, $n = 101$). *Hml*$^{\Delta}$*>GFP/bmm*^{RNAi} ($N = 3$, $n = 58$, $P = 0.092$) in comparison to *Hml*$^{\Delta}$*>GFP/TRiP (III) control* (Ct.HSD, $N = 3$, $n = 97$). *Hml*$^{\Delta}$*>GFP/UAS-bmm* ($N = 3$, $n = 144$, $P < 0.0001$) in comparison to *Hml*$^{\Delta}$*>GFP/w*^{1118} *control* (Ct.HSD, $N = 3$, $n = 90$). (**J**) Quantification of body length (RF, females) in *Hml*$^{\Delta}$*>GFP/crq*^{RNAi} ($N = 3$, $n = 87$, $P = 0.6118$) in comparison to *Hml*$^{\Delta}$*>GFP/TRiP (II) control* (Ct.HSD, N = 3, n = 82). *Hml*$^{\Delta}$*>GFP/bmm*^{RNAi} ($N = 3$, $n = 74$, $P = 0.0723$) in comparison to *Hml*$^{\Delta}$*>GFP/TRiP (III) control* (Ct.HSD, $N = 3$, $n = 59$). *Hml*$^{\Delta}$*>GFP/UAS-bmm* ($N = 3$, $n = 90$, $P = 0.2505$) in comparison to *Hml*$^{\Delta}$*>GFP/w*^{1118} *control* (Ct.HSD, $N = 3$, $n = 90$). (**J′**) Quantification of wing area (RF, females) in *Hml*$^{\Delta}$*>GFP/crq*^{RNAi} ($N = 3$, $n = 60$, $P = 0.3552$) in comparison to *Hml*$^{\Delta}$*>GFP/TRiP (II) control* (Ct.HSD, $N = 3$, $n = 66$). *Hml*$^{\Delta}$*>GFP/bmm*^{RNAi} ($N = 3$, $n = 77$, $P = 0.0938$) in comparison to *Hml*$^{\Delta}$*>GFP/TRiP (III) control* (Ct.HSD, $N = 3$, $n = 169$). *Hml*$^{\Delta}$*>GFP/UAS-bmm* ($N = 3$, $n = 155$, $P < 0.0001$) in comparison to *Hml*$^{\Delta}$*>GFP/w*^{1118} *control* (Ct.HSD, $N = 3$, $n = 112$). Data information: In quantification graphs each dot represents an animal. Comparison for significance is with respective background controls. Asterisks mark statistically significant differences (*$P < 0.05$; **$P < 0.01$; ***$P < 0.001$; ****$P < 0.0001$). The statistical analysis applied for all panels (**A–J′**) is Mann–Whitney test. N indicates the number of independent biological replicates, and *n* refers to the total number of animals analyzed. RF is regular food. Box plots show the median (center line), 25th–75th percentiles (bounds of box), and whiskers extending to the minimum and maximum values; all individual data points are shown. Source data are available online for this figure.

 © The Author(s)
